# Glycosylation of Methylflavonoids in the Cultures of Entomopathogenic Filamentous Fungi as a Tool for Obtaining New Biologically Active Compounds

**DOI:** 10.3390/ijms23105558

**Published:** 2022-05-16

**Authors:** Agnieszka Krawczyk-Łebek, Monika Dymarska, Tomasz Janeczko, Edyta Kostrzewa-Susłow

**Affiliations:** Department of Food Chemistry and Biocatalysis, Faculty of Biotechnology and Food Science, Wrocław University of Environmental and Life Sciences, 50-375 Wrocław, Poland; monika.dymarska@upwr.edu.pl (M.D.); tomasz.janeczko@upwr.edu.pl (T.J.)

**Keywords:** biotransformations, methylflavonoids, glycosylation, 4-*O*-methylglucopyranoside, *Beauveria bassiana*, *Isaria fumosorosea*, antimicrobial, anticarcinogenic, hepatoprotective, cardioprotective

## Abstract

Flavonoid compounds are secondary plant metabolites with numerous biological activities; they naturally occur mainly in the form of glycosides. The glucosyl moiety attached to the flavonoid core makes them more stable and water-soluble. The methyl derivatives of flavonoids also show increased stability and intestinal absorption. Our study showed that such flavonoids can be obtained by combined chemical and biotechnological methods with entomopathogenic filamentous fungi as glycosylation biocatalysts. In the current paper, two flavonoids, i.e., 2′-hydroxy-4-methylchalcone and 4′-methylflavone, have been synthesized and biotransformed in the cultures of two strains of entomopathogenic filamentous fungi *Isaria fumosorosea* KCH J2 and *Beauveria bassiana* KCH J1.5. Biotransformation of 2′-hydroxy-4-methylchalcone resulted in the formation of two dihydrochalcone glucopyranoside derivatives in the culture of *I. fumosorosea* KCH J2 and chalcone glucopyranoside derivative in the case of *B. bassiana* KCH J1.5. 4′-Methylflavone was transformed in the culture of *I. fumosorosea* KCH J2 into four products, i.e., 4′-hydroxymethylflavone, flavone 4′-methylene-*O*-*β*-d-(4″-*O*-methyl)-glucopyranoside, flavone 4′-carboxylic acid, and 4′-methylflavone 3-*O*-*β*-d-(4″-*O*-methyl)-glucopyranoside. 4′-Methylflavone was not efficiently biotransformed in the culture of *B. bassiana* KCH J1.5. The computer-aided simulations based on the chemical structures of the obtained compounds showed their improved physicochemical properties and antimicrobial, anticarcinogenic, hepatoprotective, and cardioprotective potential.

## 1. Introduction

Flavonoids are a large group of polyphenolic secondary metabolites of plants that exhibit extensive bioactive benefits, such as antiviral, antibacterial, anti-inflammatory, cardioprotective, hepatoprotective, skin senescence preventive and treatment, and anticarcinogenic [1,2,3,4,5,6,7,8,9] properties. With regard to their antiviral activity, flavonoids act at different stages of viral infection, including entrance, replication, and translation of viral proteins. They may be effective against many viruses, including influenza, human immunodeficiency virus (HIV), severe acute respiratory syndrome (SARS), and severe acute respiratory syndrome coronavirus-2 (SARS-CoV-2) [8,9]. A comparison of the inhibitory concentration of flavonoids and standard antiviral compounds showed that flavonoids, such as baicalein and apigenin (flavones), and quercetin and fisetin (flavonols), exhibited similar or even greater activity than currently used drugs [8,9]. Concerning the cardioprotective activity of flavonoids, this is supported by both mechanistic and epidemiologic evidence, which suggests that a diet rich in flavonoids may be beneficial in reducing the risk of cardiovascular disease [7,10].

**Scheme 1:**
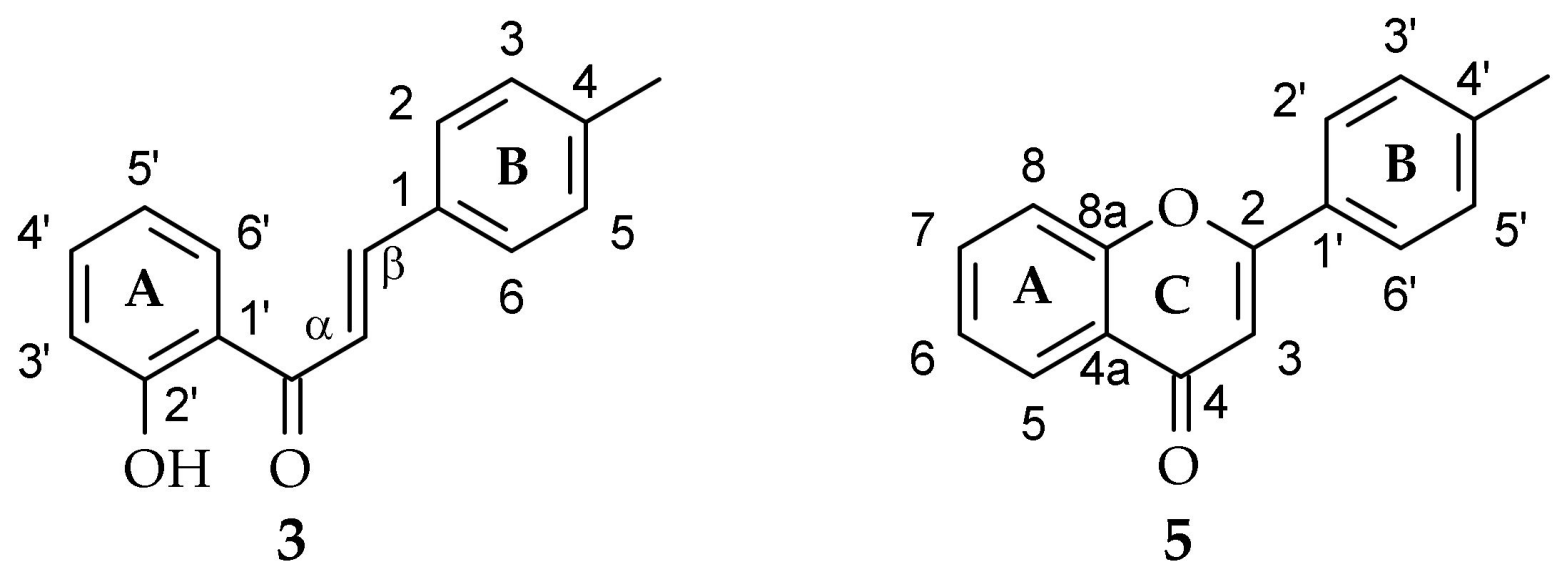
The chemical structure of 2′-hydroxy-4-methylchalcone (**3**) and 4′-methylflavone (**5**).

Flavonoids are usually found in plants in glycosylated, methylated, prenylated, acetylated, and polymerized forms, but glycosides are most common [11,12,13]. The occurrence of flavonoids in glycosylated or methylated forms in plants affects their properties by improving stability, water-solubility, bioavailability, and some types of bioactivity [13,14,15]. *O*-glycosylation can increase anti-virus, anti-obesity, anti-adipogenic, and anti-allergic activity. Furthermore, orally administered flavonoid glycosides keep higher plasma levels and exhibit a longer mean residence time compared to their aglycone forms [15]. Flavonoids are categorized into subclasses depending on their chemical structure. The main subclasses of most common dietary flavonoids are flavonols, flavones, flavan-3-ols, anthocyanidins, flavanones, and isoflavones. Regarding the general chemical structure, flavonoids consist of three C-6-C-3-C-6 rings, namely A (C-6) and B (C-6), linked by a heterocyclic C-ring (C-3) [12,16]. However, chalcones (another subclass of flavonoids) are composed of two aromatic rings joined by a three-carbon α, β-unsaturated carbonyl system [16,17]. The structures of two flavonoids being the subject of the presented paper, i.e., 2′-hydroxy-4-methylflavone and 4′-methylflavone, are shown in Scheme 1.

Dihydrochalcones, which have a saturated bond between carbons α and β, also occur quite abundantly in some plant species; for example, phloretin 4-*O*-*β*-d-glucopyranoside isolated from the stems of *Homalium stenophyllum* [18], 2′,5′-dimethyl-3′-methoxy-4′,6′-dihydroxydihydrochalcone from *Empetrum nigrum* L. var. *japonicum* K. Koch (*E. nigrum*) [19], and glycoside derivatives of phloretin with one or more glycosidic moieties attached at different positions of the flavonoid core from subaerial parts of *Thonningia sanguinea*. The latter moderately inhibited the protein tyrosine phosphatase-1B activity, the increased activity of which is related to diseases such as obesity, type 2 diabetes, and carcinoma [20]. Another dihydrochalcone, 3′-formyl-2′,4′,6′-trihydroxy-5′-methyldihydrochalcone also exhibited antimicrobial and anticancer potential [21].

The extraction of flavonoids from plants does not meet the current quantitative requirements. The application of chemical synthesis is also not sufficient for the production of flavonoid glycosides because of low stereo and regioselectivity and drastic reaction conditions resulting in the decomposition of flavonoid aglycones. To achieve better results, enzymatic reactions using glycosyltransferases and also glucosyltransferase-methyltransferase functional modules have been applied [22,23,24]. Such reactions can be carried out as whole-cell biotransformation, in vivo total biosynthesis, and in vitro enzymatic reaction [22,23,24,25,26,27]. Among these methods, the whole-cell biotransformation of flavonoid aglycones is promising because of its simplicity and low cost.

The primary goal of the presented study was to investigate the ability of two entomopathogenic filamentous fungi strains, i.e., *Isaria fumosorosea* KCH J2 and *Beauveria bassiana* KCH J1.5, to glycosylate 2′-hydroxy-4-methylchalcone and 4′-methyflavone, and to evaluate the potential biological activity of the obtained derivatives using in silico methods. The used strains had been employed in the glycosylation reactions described in our previous studies [25,28,29,30,31,32,33]. The biotransformation substrates were obtained by our team in a two-step synthesis described in our earlier work [34].

## 2. Results and Discussion

The application of entomopathogenic filamentous fungi as biocatalysts in the transformation of 2′-hydroxy-4-methylchalcone and 4′-methylflavone in the cultures of *I. fumosorosea* KCH J2 and *B. bassiana* KCH J1.5 resulted in the formation of five new flavonoid glycosides. The products of the biotransformations were isolated and purified using the preparative thin-layer chromatography (TLC). The chemical structures of the biotransformation products were established based on the Nuclear Magnetic Resonance (NMR) spectroscopy and Liquid Chromatography-Mass Spectrometry (LC-MS).

### 2.1. Biotransformation of 2′-Hydroxy-4-Methylchalcone (***3***) in the Culture of I. fumosorosea KCH J2

**Scheme 2:**
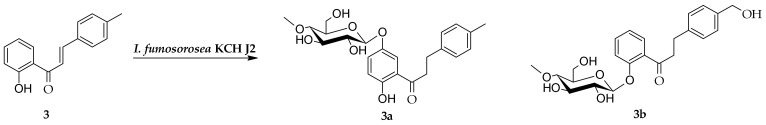
Microbial transformation of 2′-hydroxy-4-methylchalcone (**3**) in *I. fumosorosea* KCH J2 culture.

2′-Hydroxy-4-methylchalcone (**3**) was biotransformed in the culture of *I. fumosorosea* KCH J2 into 2′-hydroxy-4-methyldihydrochalcone 5′-*O*-*β*-d-(4″-*O*-methyl)-glucopyranoside (**3a**) with a 4.9% yield (4.4 mg) and 4-hydroxymethyldihydrochalcone 2′-*O*-*β*-d-(4″-*O*-methyl)-glucopyranoside (**3b**) with a 7.3% yield (6.6 mg) (Scheme 2).

**Scheme 3:**
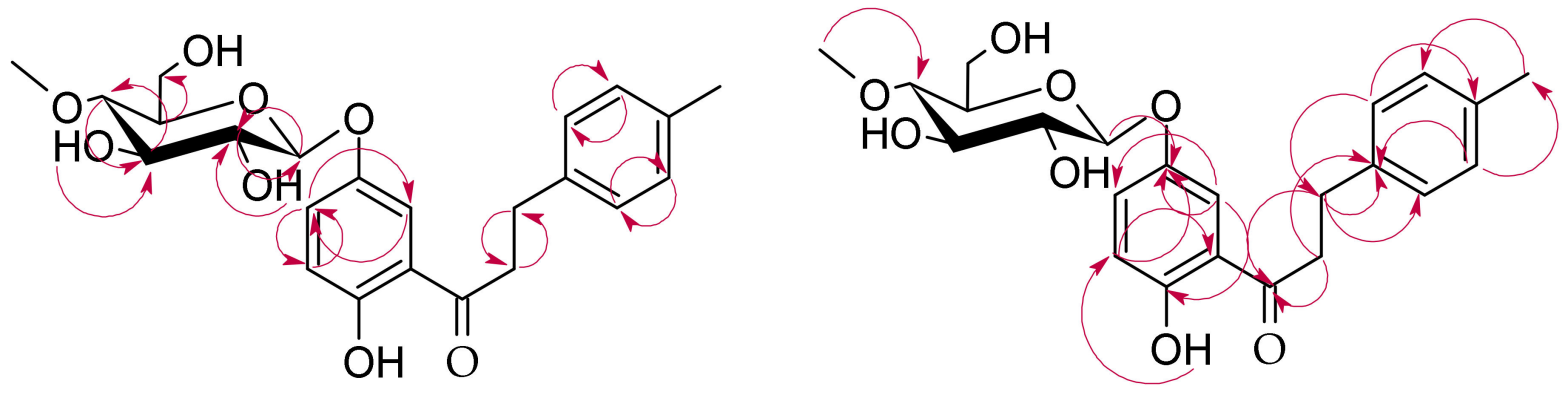
Key COSY (on the **left**) and HMBC (on the **right**) correlations for the structure elucidation of product **3a**.

**Scheme 4:**
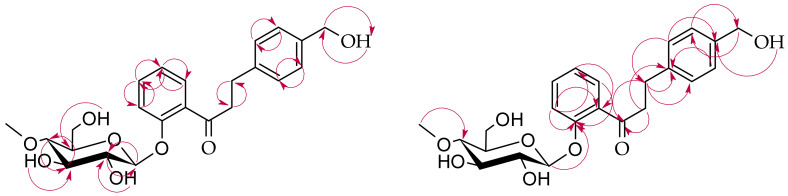
Key COSY (on the **left**) and HMBC (on the **right**) correlations for the structure elucidation of product **3b**.

The structures of products **3a**–**3b** were determined based on NMR spectroscopy (Table 1 and Table 2, Scheme 3 and Scheme 4 showing key COSY (Correlation Spectroscopy) and HMBC (Heteronuclear Multiple Bond Coherence) correlations). In product **3a**, the presence of a glucose moiety was confirmed by five characteristic carbon signals observed in the region from δ = 80.4 to δ = 62.3 ppm in the ^13^C-NMR spectrum (Carbon-13 Nuclear Magnetic Resonance) (Supplementary Materials: Figure S27), as well as proton signals of δ_H_ ranging from δ = 3.84 to δ = 3.15 ppm in the ^1^H-NMR spectrum (Proton Nuclear Magnetic Resonance) (Supplementary Materials: Figure S24). In the ^1^H-NMR spectrum, a one-proton doublet from the proton at the anomeric carbon atom was present at δ = 4.83 ppm with the coupling constant (*J* = 7.8 Hz), evidencing *β*-configuration of the glucose (Supplementary Materials: Figure S24). The glucose molecule was also *O*-methylated at C-4″ because in the ^1^H-NMR spectrum, a three-proton singlet at δ = 3.55 ppm, with the corresponding signal at δ = 60.6 ppm in the ^13^C-NMR spectra, was observed (Supplementary Materials: Figures S24 and S27). Moreover, this moiety was correlated with the signal of C-4″ (δ = 80.4 ppm) in the HMBC experiment, which evidences the position of the substitution with the -*O*-CH_3_ group in the glucose unit (Supplementary Materials: Figure S38). In product **3a**, the reduction of a double bond between C-α and C-β occurred and caused the shift of the protons at C-α from 7.93 (in **3**) to 3.42 ppm (in **3a**) and the protons at C-β from 8.02 (in **3**) to 3.00 ppm (in **3a**) (Supplementary Materials: Figures S3 and S24). Naturally, shifted protons at C-α and C-β were correlated with the carbonyl group and the C-1 signal in the HMBC experiment (Supplementary Materials: Figure S40). The signals from one proton of the hydroxyl group at C-2′ and three protons of the methyl group at C-4 remained intact. The 4″-*O*-methylglucopyranoside was attached at C-5′ because in the HMBC experiment, signals from shifted protons at C-3′ (δ = 6.87 ppm) and C-6′ (δ = 7.64 ppm) correlated with the shifted signal from C-5′ (δ = 150.8 ppm). Moreover, the proton at the anomeric carbon atom (δ = 4.83 ppm) also correlated with the shifted signal from C-5′ (δ = 150.8 ppm) (Supplementary Materials: Figures S37 and S39).

In the case of product **3b**, the presence of a glucose moiety was also confirmed by five characteristic carbon signals observed in the region from δ = 80.1 to δ = 62.1 ppm in the ^13^C-NMR spectrum (Supplementary Materials: Figure S52), as well as proton signals of δ_H_ ranging from δ = 3.84 to δ = 3.21 ppm in the ^1^H-NMR spectrum (Supplementary Materials: Figure S49). The presence of a proton at the anomeric carbon atom at δ = 5.06 ppm with the coupling constant (*J* = 7.8 Hz) confirmed the *β*-configuration of the glucose unit in the ^1^H NMR spectrum (Supplementary Materials: Figure S49). *O*-methylation of the glucose unit at C-4″ was established in the HMBC experiment by correlation of the signal from a three-proton singlet at δ = 3.56 ppm, with the corresponding signal at δ = 60.6 ppm in the ^13^C-NMR spectrum and the signal of C-4″ (δ = 80.1 ppm) (Supplementary Materials: Figure S61). Similar to product **3a**, the reduction of a double bond between C-α and C-β also occurred and caused the shift of the protons at C-α from 7.93 (in **3**) to 3.41 ppm (in **3b**) and the protons at C-β from 8.02 (in **3**) to 2.97 ppm (in **3b**) (Supplementary Materials: Figures S3 and S49). In the HMBC experiment, the shifted protons at C-α and C-β correlated with the carbonyl group and with C-1 (Supplementary Materials: Figures S63 and S64). In this case, the signal from three protons of the methyl group at C-4 disappeared because the C-4-CH_3_ group was hydroxylated. Two new signals appeared in the ^1^H NMR spectrum, one from the methylene group at C-4 at δ = 4.58 ppm (*J* = 5.7 Hz) and the second one from the hydroxyl group attached to this methylene group at δ = 4.08 ppm (*J* = 5.8 Hz) (Supplementary Materials: Figure S49). In the HMBC experiment, the correlation between the protons at C-4-CH_2_- (δ = 4.58 ppm) and the signal from C-2 and C-6 (δ = 127.6 ppm) along with the correlation between the proton of the hydroxyl group at C-4-CH_2_-OH (δ = 4.08 ppm) and C-4 (δ = 141.2 ppm) confirmed the hydroxylation of the methyl group at C-4 (Supplementary Materials: Figure S63). The absence of the signal from one proton of the hydroxyl group at C-2′ pointed out that glycosylation occurred at this position. Moreover, in the HMBC experiment, signals from shifted protons at C-3′ (δ = 7.29 ppm) and C-6′ (δ = 7.57 ppm) correlated with the shifted signal from C-2′ (δ = 157.1 ppm) and the proton at the anomeric carbon atom (δ = 5.06 ppm) also correlated with the shifted signal from C-2′ (δ = 157.1 ppm) (Supplementary Materials: Figures S51, S62 and S63).

### 2.2. Biotransformation of 2′-Hydroxy-4-Methylchalcone (***3***) in the Culture of B. bassiana KCH J1.5

**Scheme 5:**
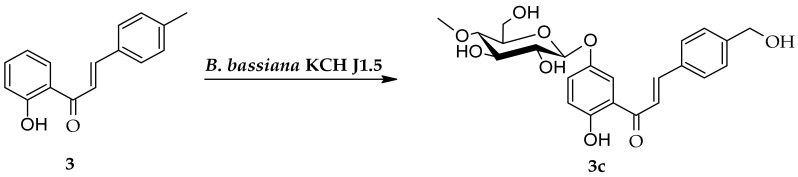
Microbial transformation of 2′-hydroxy-4-methylchalcone (**3**) in *B. bassiana* KCH J1.5 culture.

The biotransformation of 2′-hydroxy-4-methylchalcone (**3**) in the culture of *B. bassiana* KCH J1.5 resulted in the formation of one product, 2′-hydroxy-4-hydroxymethylchalcone 5′-*O*-*β*-d-(4″-*O*-methyl)-glucopyranoside (**3c**) with a 4.9% yield (4.6 mg) (Scheme 5).

**Scheme 6:**
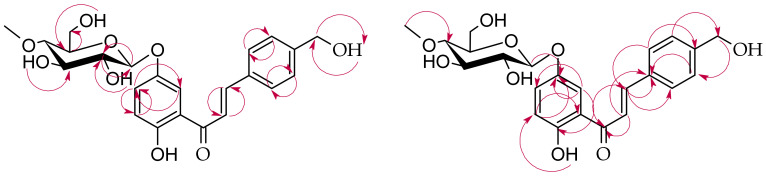
Key COSY (on the **left**) and HMBC (on the **right**) correlations for the structure elucidation of product **3c**.

The structure of product **3c** was also determined based on NMR spectroscopy (Table 1 and Table 2, Scheme 6 showing key COSY and HMBC correlations). Like in the cases of the products **3a** and **3b**, in the ^1^H NMR and ^13^C NMR, characteristic signals from the protons and carbons of the glucose unit were observed (Supplementary Materials: Figures S73 and S76). Furthermore, the attachment of this glucose unit to substrate **3** was confirmed by the presence of a one-proton doublet from the proton at the anomeric carbon atom at δ = 4.92 ppm in the ^1^H-NMR spectrum with the characteristic coupling constant (*J* = 7.8 Hz) typical for its *β*-configuration (Supplementary Materials: Figure S73). The *O*-methylation of the glucose unit at C-4″ was confirmed in the HMBC experiment by correlation of the signal from a three-proton singlet at δ = 3.57 ppm (with the corresponding signal at δ = 60.6 ppm in the ^13^C-NMR spectrum) and the signal of C-4″ (δ = 80.6 ppm) (Supplementary Materials: Figure S86). Unlike products **3a** and **3b**, the double bond between C-α and C-β was not reduced in the case of product **3c**. The signal from one proton of the hydroxyl group at C-2′ remained intact. However, the signal from three protons of the methyl group at C-4 disappeared because the C-4-CH_3_ group was hydroxylated, the same as in the case of product **3b**. Two new signals appeared in the ^1^H NMR spectrum, one from the methylene group at C-4 at δ = 4.70 ppm and the second one from the hydroxyl group attached to this methylene group at δ = 4.39 ppm (Supplementary Materials: Figure S73. The correlation between the protons at C-4-CH_2_- (δ = 4.70 ppm) and the signal from C-3 and C-5 (δ = 127.8 ppm) confirmed the hydroxylation of the methyl group at C-4 (Supplementary Materials: Figure S87). The 4″-*O*-methyl-glucopyranoside was attached at C-5′, the same as in the case of the product **3a**, because in the HMBC experiment, the signals from shifted protons at C-3′ (δ = 6.92 ppm) and C-6′ (δ = 7.91 ppm) correlated with the shifted signal from C-5′ (δ = 150.9 ppm). Furthermore, the proton at the anomeric carbon atom (δ = 4.92 ppm) also correlated with the shifted signal from C-5′ (δ = 150.9 ppm) (Supplementary Materials: Figures S85 and S87).

Our previous and other teams’ studies showed that entomopathogenic filamentous fungi can be a valuable tool in the biotransformation of phenolic compounds, especially flavonoids, including 4″-*O*-methylglucosylation and hydroxylation [23,25,28,29,30,31,35,36,37].

Microbial glycosylation of chalcones in the cultures of filamentous fungi has been described in the literature only a few times. Some previous reports showed that xanthohumol can be biotransformed in the cultures of *Penicillium chrysogenum* 6933 [38], *Absidia coerulea* AM93, and *Rhizopus nigricans* UPF701 [39] to form 4′-*O*-*β*-d-glucopyranoside derivative and in the cultures of *B. bassiana* AM278 [40] and *B. bassiana* AM446 [39] to form 4′-*O*-*β*-d-(4″-*O*-methyl)-glucopyranoside derivative.

Our previous studies on 2′-hydroxy-2-methylchalcone and 2′-hydroxy-5′-methylchalcone biotransformations revealed that the strains *B. bassiana* KCH J1.5 and *I. fumosorosea* KCH J2 were capable of forming 2′-hydroxy-5′-methylchalcone 3-*O*-*β*-d-(4″-*O*-methyl)-glucopyranoside from 2′-hydroxy-5′-methylchalcone [28]. Furthermore, *B. bassiana* KCH J1.5 was able to biotransform 2′-hydroxy-2-methylchalcone into four derivatives with the glucose moiety attached at C-3′: 2′-hydroxy-2-methyldihydrochalcone 3′-*O*-*β*-d-(4″-*O*-methyl)-glucopyranoside, 2′,3-dihydroxy-2-methyldihydrochalcone 3′-*O*-*β*-d-(4″-*O*-methyl)-glucopyranoside, 2′-hydroxy-2-hydroxymethyldihydrochalcone 3′-*O*-*β*-d-(4″-*O*-methyl)-glucopyranoside, and 2′,4-dihydroxy-2-methyldihydrochalcone 3′-*O*-*β*-d-(4″-*O*-methyl)-glucopyranoside. On the other hand, *I. fumosorosea* KCH J2 strain biotransformed 2′-hydroxy-2-methylchalcone into another product, i.e., 3-hydroxy-2-methyldihydrochalcone 2′-*O*-*β*-d-(4″-*O*-methyl)-glucopyranoside.

The results of the present study also showed different regioselectivity of glycosyltransferase-methyltransferase functional modules of both strains. Moreover, in the case of *B. bassiana* KCH J1,5 microbial transformation, the only biotransformation product, 2′-hydroxy-4-hydroxymethylchalcone 5′-*O*-*β*-d-(4″-*O*-methyl)-glucopyranoside (**3c**), was glycosylated at C-5′, not at C-2′ like in the case o 2′-hydroxy-2-methylchalcone. Surprisingly, the reduction of the double bond of chalcone did not occur, and a relatively low yield characterized this biotransformation. On the other hand, *I. fumosorosea* KCH J2 strain catalyzed glycosylation at the two positions: 2′ (**3b**) and 5′ (**3a**). Interestingly, product **3b** was also hydroxylated at C-4-CH_3_. We have not observed such a reaction in the culture of this strain in our previous works.

The reduction of the double bond of chalcones has been observed in the cultures of many microorganisms, as we highlighted in our previous work [29], the same as glycosylation of dihydrochalcones [41]. Our innovation is to perform these reactions simultaneously in one pot using extensive enzymatic systems of the selected strains of entomopathogenic filamentous fungi [29]. These fungi naturally produce enzymes to penetrate the insect’s cuticle. The best known are their cuticular degrading enzymes: proteases, chitinases, chitosanase, and lipases [42,43]. However, our previous studies revealed that both strains accept flavonoid substrates and catalyze reactions of hydroxylation, glycosylation, and reduction [25,28,29,30,31,32,33,37]. Xie and coworkers identified a glycosyltransferase–methyltransferase gene pair that encodes the methyglucosylation functional module in *B. bassiana* [44]. Whereas the enzymatic oxidations are probably catalyzed by cytochrome P450 monooxygenases [45]. However, most of the enzymes responsible for these reactions have not been fully recognized.

### 2.3. Biotransformation of 4′-Methylflavone (***5***) in the Culture of I. fumosorosea KCH J2

**Scheme 7:**
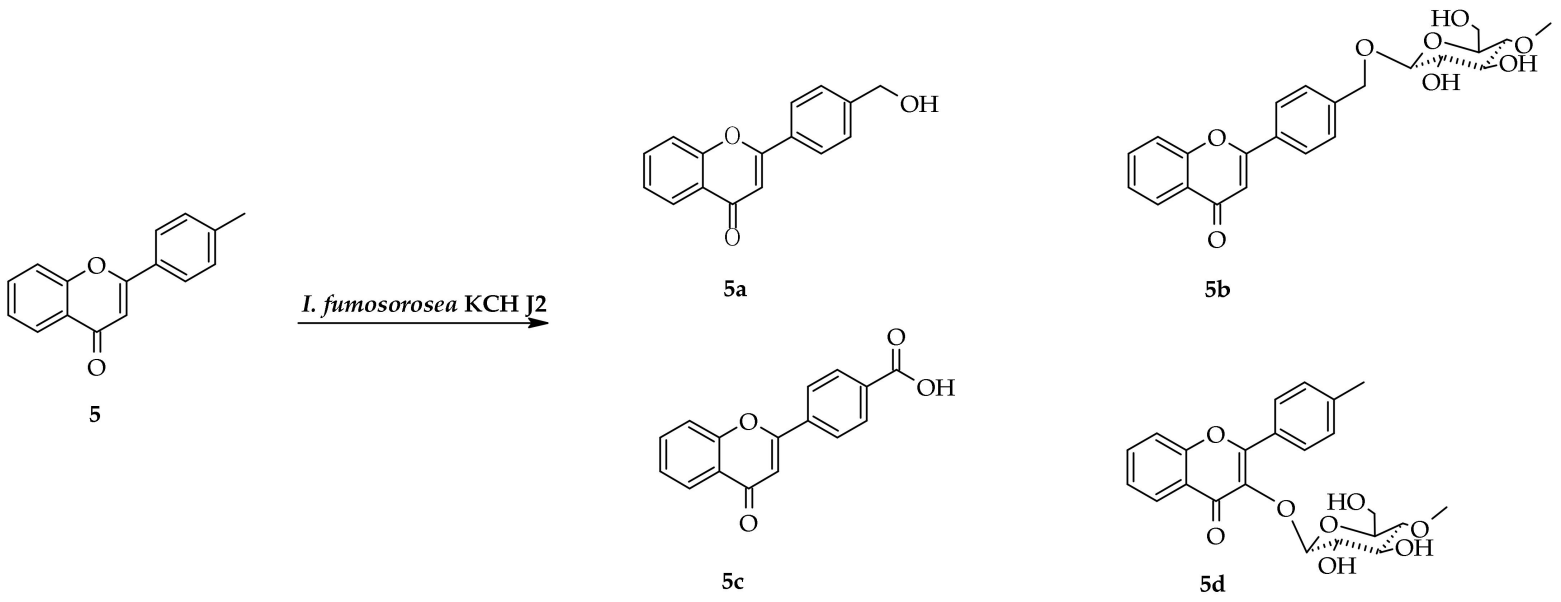
Microbial transformation of 4′-methylflavone (**5**) in *I. fumosorosea* KCH J2 culture.

The biotransformation of 4′-methylflavone in the culture of *I. fumosorosea* KCH J2 resulted in the formation of four products, i.e., 4′-hydroxymethylflavone (**5a**) with a 10.1% yield (5.4 mg), flavone 4′-methylene-*O*-*β*-d-(4″-*O*-methyl)-glucopyranoside (**5b**) with an 8.1% yield (7.3 mg), flavone 4′-carboxylic acid (**5c**) with a 32.7% yield (18.4 mg), and 4′-methylflavone 3-*O*-*β*-d-(4″-*O*-methyl)-glucopyranoside (**5d**) with a 3.9% yield (3.5 mg) (Scheme 7).

**Scheme 8:**
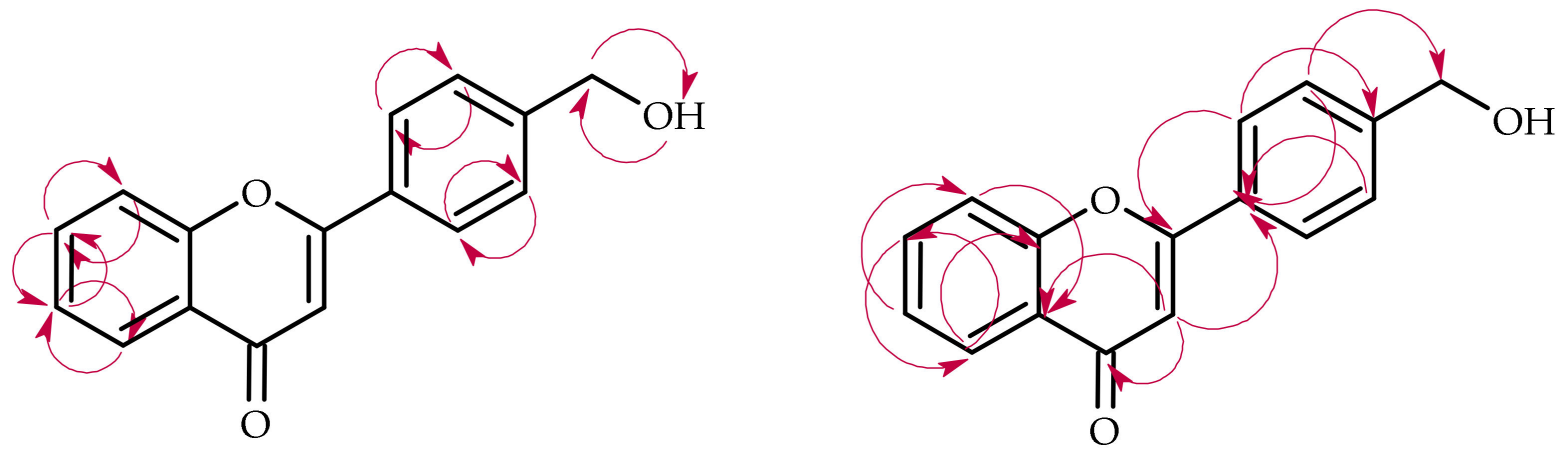
Key COSY (on the **left**) and HMBC (on the **right**) correlations for the structure elucidation of product **5a**.

The first product (**5a**) was 4′-hydroxymethylflavone with the chemical structure determined based on the NMR spectroscopy (Table 3 and Table 4, Scheme 8 with key COSY and HMBC correlations). In the ^1^H NMR spectrum of product **5a**, the signal from the 4′-methyl group at δ = 2.43 ppm disappeared, and the methylene group signal appeared at δ = 4.76 ppm (*J* = 5.8 Hz) along with the signal of the attached hydroxyl group at δ = 4.47 ppm (*J* = 5.8 Hz), which proved the substitution at 4′-CH_3_ of the substrate **5** (Supplementary Materials: Figure S114). In addition, the signal of two protons at C-3′ and C-5′ became shifted towards the lower field from δ = 7.41 (in **5**) to δ = 7.59 ppm (in **5a**) (Supplementary Materials: Figures S95 and S114). The signal of C-4′ was also shifted in the ^13^C NMR spectrum from δ = 143.1 (in **5**) to δ = 147.6 ppm (in **5a**) (Supplementary Materials: Figures S96 and S116). Likewise, in the HMBC experiment, the correlation between the protons at C-4′-CH_2_- (δ = 4.76 ppm) and the signal from C-3′, C-5′ (δ = 127.8), and the correlation between the protons at C-3′, C-5′ (δ = 7.59 ppm) and the carbon signal of 4′-CH_2_ (δ = 64.1 ppm), confirmed substitution with the hydroxyl moiety at C-4′-CH_3_ (Supplementary Materials: Figure S123).

**Scheme 9:**
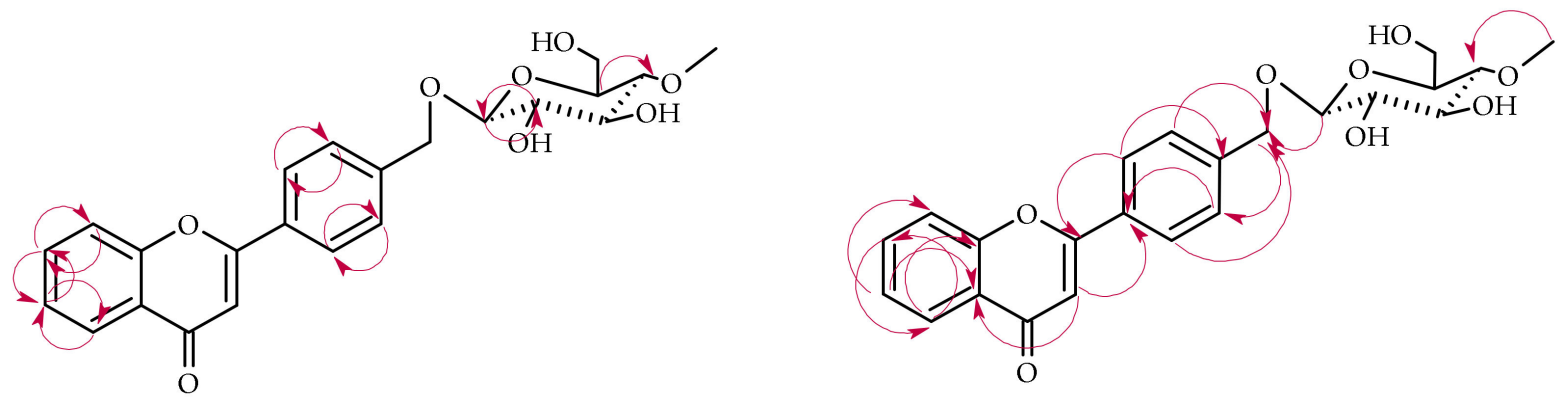
Key COSY (on the **left**) and HMBC (on the **right**) correlations for the structure elucidation of product **5b**.

The second biotransformation product, **5b**, was the *O*-methylglycosylated 4′-methylflavone derivative (Table 3 and Table 4, Scheme 9 with key COSY and HMBC correlations). Like in the cases of the products **3a**–**3c**, characteristic signals from the protons and carbons of the glucose moiety were observed in the ^1^H NMR and ^13^C NMR spectra (Supplementary Materials: Figures S133 and S136). The attachment of a glucose unit to substrate **5** was confirmed by the presence of a one-proton doublet from the proton at the anomeric carbon atom at δ = 4.42 ppm in the ^1^H-NMR spectrum with the characteristic coupling constant (*J* = 7.8 Hz) for its *β*-configuration (Supplementary Materials: Figure S133). A three-proton singlet at δ = 3.54 ppm in the ^1^H NMR spectrum with the corresponding signal at δ = 60.5 ppm in the ^13^C-NMR spectrum evidenced the appearance of a -OCH_3_ moiety (Supplementary Materials: Figures S133 and S136). Moreover, the correlation of this signal with the C-4″ signal at δ 80.5 ppm confirmed that *O*-methylation occurred at the C-4″ hydroxyl group of the sugar moiety (Supplementary Materials: Figure S146). The appearance in the ^1^H NMR spectrum of two one-proton signals from the methylene moiety at δ = 5.00 and δ = 4.76 ppm instead of a three-proton signal from the methyl group at 2.43 ppm of substrate **5** evidenced the substitution at C-4′-CH_3_ (Supplementary Materials: Figure S133). The presence of the characteristic AA’BB’ coupling system with the signals from protons at C-2′ and C-6′ and signals from protons at C-3′ and C-5′ only slightly shifted confirmed substitution at C-4′-CH_3_ (Supplementary Materials: Figure S132). In the HMBC experiment, a proton at the anomeric carbon atom (δ = 4.42 ppm) was visibly coupled with the C-4′-CH_2_- signal (δ = 70.3 ppm), proving substitution with the glucose unit at this position (Supplementary Materials: Figure S146).

**Scheme 10:**
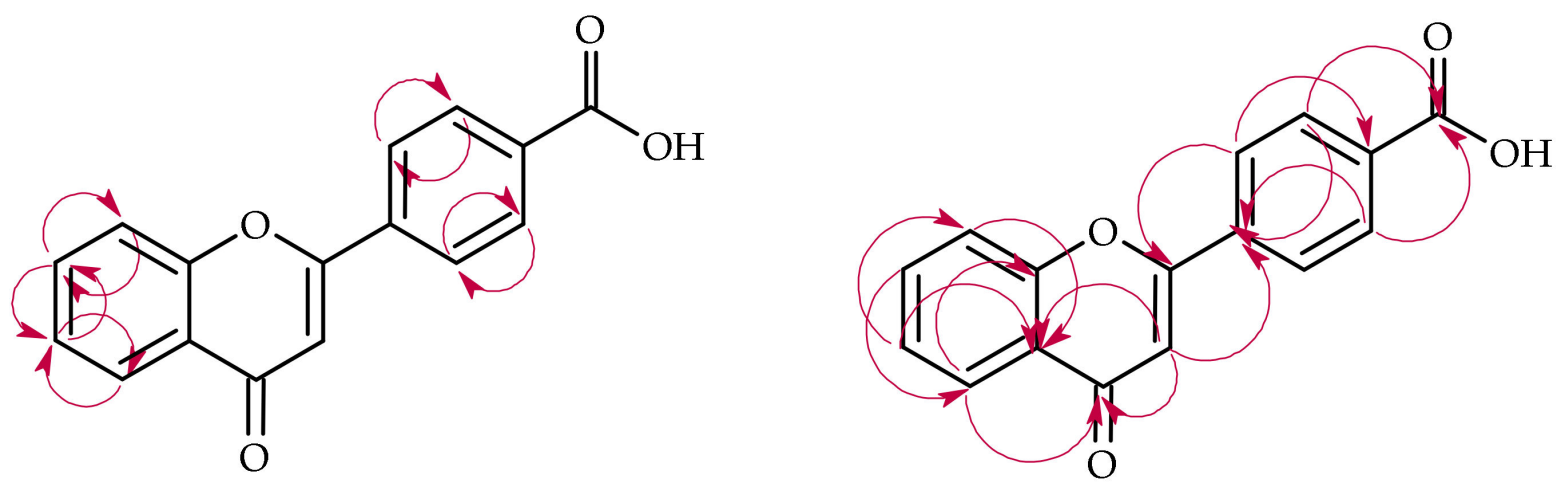
Key COSY (on the **left**) and HMBC (on the **right**) correlations for the structure elucidation of product **5c**.

The product **5c** was flavone 4′-carboxylic acid that probably arose as a product of further oxidation of the hydroxyl group attached at C-4′-CH_3_ (Table 3 and Table 4, Scheme 10 with key COSY and HMBC correlations). In the ^1^H NMR spectrum, the signal from the 4′-methyl group at δ = 2.43 ppm was absent (Supplementary Materials: Figures S94 and S153). In addition, the signal of two protons at C-3′ and C-5′ was shifted strongly towards the lower field from δ = 7.41 (in **5**) to δ = 8.23 ppm (in **5c**) (Supplementary Materials: Figures S95 and S154). Likewise, the signal of two protons at C-2′ and C-6′ was also shifted strongly towards the lower field from δ = 7.99 (in **5**) to δ = 8.26 ppm (in **5c**) (Supplementary Materials: Figures S95 and S154). In the ^13^C NMR spectrum, the signal of C-4′ was shifted from δ = 143.1 (in **5**) to δ = 134.0 ppm (in **5a**), and the signal of C-4′-CH_3_ disappeared (Supplementary Materials: Figures S96 and S156). In the ^1^H NMR spectrum, the new signal appeared at δ = 166.9 ppm and correlated with the protons at C-3′ and C-5′ in the HMBC experiment. This signal was from the carboxylic group at C-4′ (Supplementary Materials: Figure S162). This unique structure of the biotransformation product was confirmed by MS analysis (Supplementary Materials: Figure S152).

**Scheme 11:**
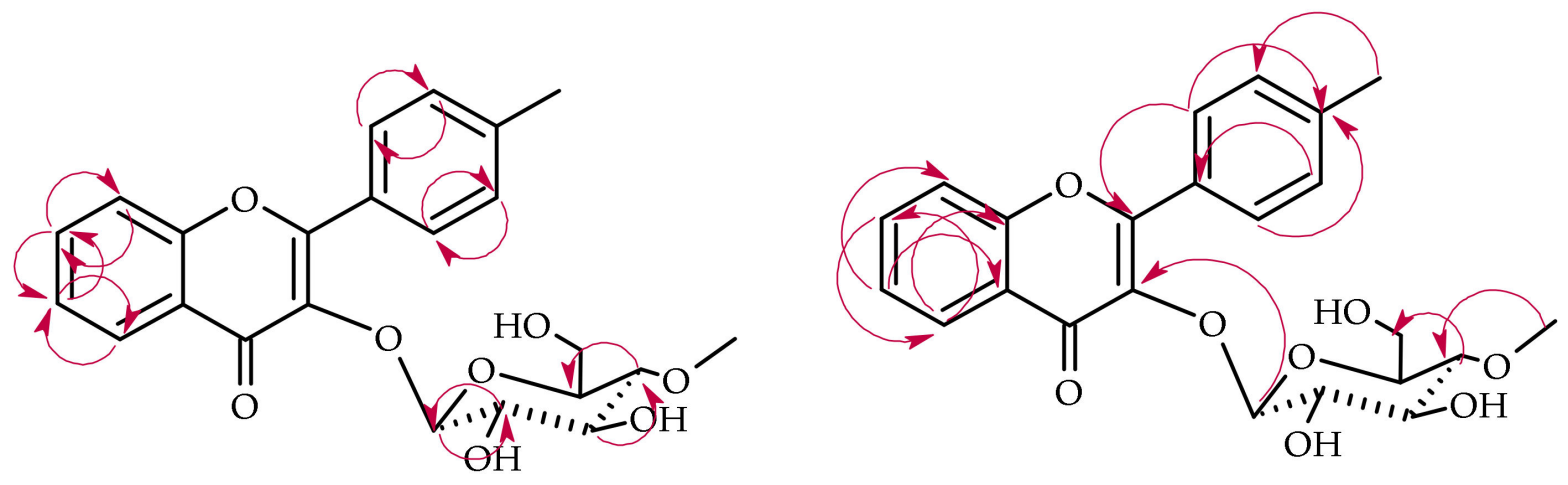
Key COSY (on the **left**) and HMBC (on the **right**) correlations for the structure elucidation of product **5d**.

The last product of 4′-methylflavone biotransformation was 4′-methylflavone 3-*O*-*β*-d-(4″-*O*-methyl)-glucopyranoside (**5d**) (Table 3 and Table 4, Scheme 11 with key COSY and HMBC correlations). In this case, the methyl group at C-4′ remained intact, and the substitution with the glycosidic moiety occurred at C-3 because, in the ^1^H NMR spectrum, the signal from the proton at C-3 disappeared (Supplementary Materials: Figures S94 and S169). The characteristic signals from the protons H2″–H6″ appeared in the ^1^H NMR spectrum (from about δ = 3.6 to about δ = 3.1 ppm, Supplementary Materials: Figure S171), as well as the corresponding carbon signals in the ^13^C-NMR spectrum (from about δ = 80 ppm to about δ = 62 ppm, Supplementary Materials: Figure S174). The *O*-methylation, which occurred at the C-4″ hydroxyl group of the sugar moiety, was confirmed because a three-proton singlet at δ = 3.51 ppm in the ^1^H NMR with the corresponding signal at δ = 60.5 ppm in the ^13^C-NMR spectra correlated with the signal of C-4″ (about δ = 80 ppm) in the HMBC experiment (Supplementary Materials: Figure S183). The signal from H-1″ at the anomeric carbon atom (at δ = 5.22 ppm in the ^1^H-NMR spectrum) coupled with the shifted carbon signal from C-3 (at δ = 138.3 ppm in the ^13^C-NMR spectrum) in the HMBC experiment, which confirms the attachment of a glucose unit to substrate **5** at C-3 (Supplementary Materials: Figure S183). The coupling constant of a one-proton doublet from H-1″ (*J* = 7.9 Hz) evidenced the glucose *β*-configuration (Supplementary Materials: Figure S171).

### 2.4. Biotransformation of 4′-Methylflavone (***5***) in the Culture of B. bassiana KCH J1.5

The biotransformation of 4′-methylflavone (**5**) in the culture of *B. bassiana* KCH J1.5 was not performed in the semi-preparative scale. The reason for this was the low conversion of the substrate during the screening procedure.

**Scheme 12:**
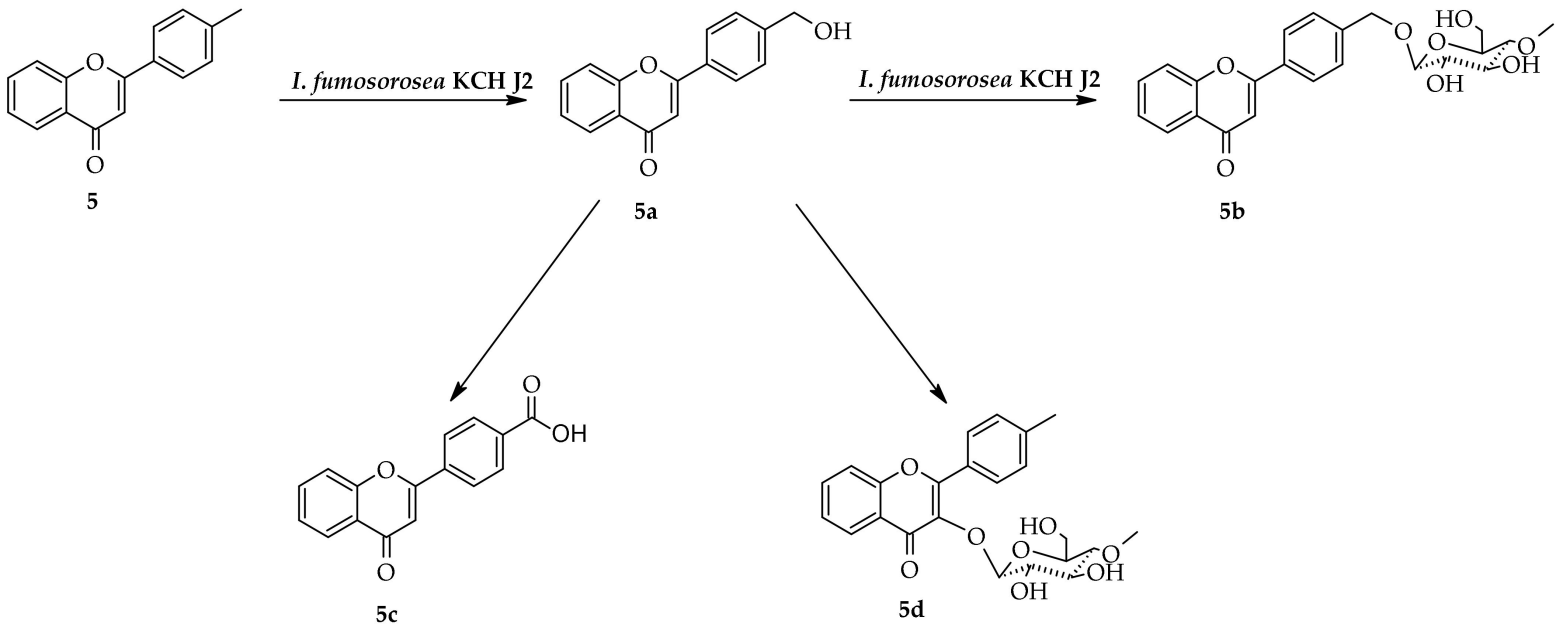
The probable course of 4′-methylflavone biotransformation in the culture of *I. fumosorosea* KCH J2.

As shown above, the biotransformation of 4′-methylflavone in the culture of *I. fumosorosea* KCH J2 resulted in the formation of four products. It can be assumed that, at first, the reaction occurred of the hydroxylation at C-4′-CH_3_ that led to 4′-hydroxymethylflavone (**5a**). Subsequently, three different reaction pathways occurred. The first one led to the creation of flavone 4′-methylene-*O*-*β*-d-(4″-*O*-methyl)-glucopyranoside (**5b**) after glycosylation of the newly formed hydroxyl group at C-4′-CH_3_, the second one led to the formation of flavone 4′-carboxylic acid (**5c**)—a product of further oxidation of the hydroxyl group at C-4′-CH_3_, and the third one ended in the formation of 4′-methylflavone 3-*O*-*β*-d-(4″-*O*-methyl)-glucopyranoside (**5d**), in which glycosylation occurred at C-3 similar to the case of 2′-methoxybiotransformation by the same fungal strain [37]. The biotransformation of 4′-methylflavanone in the cultures of *I. fumosorosea* KCH J2 resulted in the formation of similar products [46]. Scheme 12 shows the probable course of 4′-methylflavone biotransformation in the culture of *I. fumosorosea* KCH J2.

### 2.5. Pharmacokinetics, Drug-Likeness, and Biological Activity Prediction

#### 2.5.1. 2′-Hydroxy-4-methylchalcone SwissADME

Pharmacokinetics, water solubility, and drug-likeness of 2′-hydroxy-4-methylchalcone and its biotransformation derivatives have been predicted using the Swiss-ADME website (Supplementary Materials: Figures S16, S41, S65 and S88). The Brain Or IntestinaL EstimateD permeation method (BOILED-Egg), as a predictive model that computes the lipophilicity and polarity of small molecules, showed that the gastrointestinal absorption of all tested molecules except 2′-hydroxy-4-hydroxymethylchalcone 5′-*O*-*β*-d-(4″-*O*-methyl)-glucopyranoside (**3c**) was high. The reaction of glycosylation of 2′-hydroxy-4-methylchalcone observed in products **3a**, **3b**, and **3c** resulted in increased water solubility. Biotransformation products lost the ability to permeate through the blood–brain barrier passively. However, they gained the ability to be actively transported, unlike aglycones, by the P-glycoprotein. The simulations showed a lack of inhibition of glycosylated derivatives **3a**, **3b**, and **3c** on cytochrome P450 enzymes (CYP1A2, CYP2C9, CYP2C19, CYP2D6, and CYP3A4), which are involved in drug metabolism in human hepatocytes. These results are encouraging since the impact on CYP450 isozymes activity is responsible for adverse drug effects [47]. Molecules **3**, **3a**, and **3b** passed Swiss-ADME platform drug-likeness estimators with zero violations. The Abbott bioavailability score (ABS), which is formulated as the probability that a compound will have >10% bioavailability in rat or measurable Caco-2 permeability, for all tested compounds was 0.55.

#### 2.5.2. 2′-Hydroxy-4-methylchalcone Way2Drug Pass Online

Another online tool, Way2Drug Pass Online, was used to predict biological activity, including pharmacological effects, mechanisms of action, and interaction with metabolic enzymes, of 2′-hydroxy-4-methylchalcone and its biotransformation derivatives (Supplementary Materials: Figures S17–S20, S42–S45, S66–S69 and S89–S92).

2′-Hydroxy-4-methylchalcone (**3**) showed great potential as an inter alia mucomembranous protector (Pa = 0.944), membrane integrity agonist (Pa = 0.901), monophenol monooxygenase inhibitor (Pa = 0.845), antihypoxic agent (Pa = 0.843), JAK2 expression inhibitor (Pa = 0.836), and carminative agent (Pa = 0.828).

The potential biological activity of 2′-hydroxy-4-methyldihydrochalcone 5′-*O*-*β*-d-(4″-*O*-methyl)-glucopyranoside (**3a**) can be highlighted as antimicrobial activity, CDP-glycerol glycerophosphotransferase inhibitor (P = 0.935) and replicase polyprotein 1ab from SARS-CoV-2 inhibitor (P = 0.812), monophenol monooxygenase inhibitor (Pa = 0.931), membrane integrity agonist (P = 0.883), anticarcinogenic (P = 0.851) hepatoprotectant (P = 0.836).

The biological activity of 4-hydroxymethyldihydrochalcone 2′-*O*-*β*-d-(4″-*O*-methyl)-glucopyranoside (**3b**) was predicted as antimicrobial activity, CDP-glycerol glycerophosphotransferase inhibitor (P = 0.943) and replicase polyprotein 1ab from SARS-CoV-2 inhibitor (P = 0.683), monophenol monooxygenase inhibitor (Pa = 0.897), anaphylatoxin receptor antagonist (P = 0.860), and anticarcinogenic (P = 0.836).

The Pass Online simulation for 2′-hydroxy-4-hydroxymethylchalcone 5′-*O*-*β*-d-(4″-*O*-methyl)-glucopyranoside (**3c**) showed activity of monophenol monooxygenase inhibitor (Pa = 0.985), antimicrobial activity, CDP-glycerol glycerophosphotransferase inhibitor (P = 0.936) and replicase polyprotein 1ab from SARS-CoV-2 inhibitor (P = 0.781), antiprotozoal activity (leishmania) (P = 0.930), and chemopreventive activity (P = 0.921).

The mucomembranous protection is related to the protection of the stomach mucosa from acid gastric juices. A reported experiment with the benzimidazole derivatives linked with the pyrimidine nucleus as mucomembranous protector showed that the observed pharmacological properties confirmed computer predictions and justified their utility [48]. As demonstrated by the simulation results, the potential effect of 2′-hydroxy-4-methylchalcone (**3**) on the gastrointestinal tract may also be related to the carminative activity in preventing the gas formation and facilitating their excretion. Flavonoids exhibiting such activity have been used in traditional medicine [49].

Plasma membrane integrity is a key factor for cellular homeostasis [50]. Therefore, the activity of 2′-hydroxy-4-methylchalcone and 2′-hydroxy-4-methyldihydrochalcone 5′-*O*-*β*-d-(4″-*O*-methyl)-glucopyranoside (**3a**) as membrane integrity agonists should be further investigated. Our previous studies also revealed that 2′-hydroxy-4-methylchalcone (**3**) had a great impact on the hydrophobic area and the low impact on the hydrophilic area of the biological membrane [34].

The simulations showed that all tested compounds could exhibit anticarcinogenic activity as a monophenol monooxygenase inhibitor. A monophenol monooxygenase (tyrosinase) increased activity is associated with the development of malignant melanoma. Hence, inhibition of this enzyme may be one of the possible treatments [51,52,53].

The predicted antihypoxic effect can be used to reduce oxidative stress in the prevention and treatment of chronic diseases [54,55,56,57]. In contrast, JAK2 expression inhibition could be used in the treatment of inter alia, diabetic kidney disease [58], polycythemia vera, essential thrombocythemia, idiopathic myelofibrosis [59], and cancer [60,61,62].

CDP-glycerol glycerophosphotransferase is responsible for the polymerization of teichoic acid chains, which play a key role in shaping the bacterial cell, integrating its envelope, creating a bacterial biofilm, and, consequently, the pathogenesis of gram-positive bacteria [63].

4-Hydroxymethyldihydrochalcone 2′-*O*-*β*-d-(4″-*O*-methyl)-glucopyranoside (**3b**) could limit the hyperactivity of anaphylatoxin as an anaphylatoxin receptor antagonist. Anaphylatoxin plays an important role in response to bacterial infections and inflammatory processes but also in sepsis, ischemia-reperfusion injuries, complex immunological diseases, and asthma [64].

The reported pharmacokinetic results and activity predictions seem encouraging as these compounds can probably be considered safe and biologically active but require further research.

#### 2.5.3. 4′-Methylflavone SwissADME

The Swiss-ADME website has been used to assess the pharmacokinetics, water solubility, and drug-likeness of 4′-methylflavone and its derivatives (Supplementary Materials: Figures S107, S125, S147, S163, and S185). The gastrointestinal absorption of 4′-methylflavone (**5**), 4′-hydroxymethylflavone (**5a**), flavone 4′-methylene-*O*-*β*-d-(4″-*O*-methyl)-glucopyranoside (**5b**), flavone 4′-carboxylic acid (**5c**), and 4′-methylflavone 3-*O*-*β*-d-(4″-*O*-methyl)-glucopyranoside (**5d**) was high. The water solubility of biotransformation products **5a**, **5b**, **5c**, and **5d** increased compared to substrate **5**. The highest increase in an aqueous solubility was observed for glycosylated products **5b** and **5d**. Both glycosylated products gained the ability to be actively transported by the P-glycoprotein. In contrast, compounds **5**, **5a**, and **5c** were predicted to permeate through the blood–brain barrier passively. All molecules tested passed Swiss-ADME platform drug-likeness estimators with zero violations. The ABS score for all tested compounds was 0.55, except molecule **5c** showing an ABS score of 0.85.

#### 2.5.4. 4′-Methylflavone Way2Drug Pass Online

The evaluation of the biological activity of 4′-methylflavone and its biotransformation products was also made (Supplementary Materials: Figures S108–S111, S126–S129, S148–S151, S164–S167, and S186–S189).

4′-Methylflavone (**5**) biological activity includes 4-nitrophenol 2-monooxygenase inhibition (Pa = 0.952), HIF-1α expression inhibition (Pa = 0.952), agonistic effect on membrane integrity (Pa = 0.947), kinase inhibition (Pa = 0.938), and as an anaphylatoxin receptor antagonist (Pa = 0.929).

4′-Hydroxymethylflavone (**5a**) was predicted as a potential membrane integrity agonist (Pa = 0.935), 4-nitrophenol 2-monooxygenase inhibitor (Pa = 0.915), HIF-1α expression inhibitor (Pa = 0.911), anaphylatoxin receptor antagonist (Pa = 0.879), and kinase inhibitor (Pa = 0.871).

Flavone 4′-methylene-*O*-*β*-d-(4″-*O*-methyl)-glucopyranoside (**5b**) was expected to act as a membrane permeability inhibitor (Pa = 0.957), vasoprotector (Pa = 0.942), CDP-glycerol glycerophosphotransferase inhibitor (P = 0.913), anaphylatoxin receptor antagonist (Pa = 0.894), and SARS-CoV-2 replicase polyprotein 1ab antagonist (Pa = 0.786).

Flavone 4′-carboxylic acid (**5c**) predicted activity was inter alia 4-nitrophenol 2-monooxygenase inhibition (Pa = 0.972), chlordecone reductase inhibition (Pa = 0.959), cholestanetriol 26-monooxygenase inhibition (Pa = 0.957), agonistic effect on membrane integrity (Pa = 0.929), and against HIV-2 integrase (Pa = 0.853).

The Pass Online simulation for 4′-methylflavone 3-*O*-*β*-d-(4″-*O*-methyl)-glucopyranoside (**5d**) showed activity as a membrane integrity agonist (Pa = 0.971), cardioprotectant (Pa = 0.939), CDP-glycerol glycerophosphotransferase inhibitor (P = 0.937), SARS-CoV-2 replicase polyprotein 1ab antagonist (Pa = 0.942), and vasoprotector (Pa = 0.914).

4′-Methylflavone (**5**) and 4′-hydroxymethylflavone (**5a**) may serve as potential hypoxia-inducible factor 1 (HIF-1) inhibitors. HIF-1 takes a central position in oxygen-sensing machinery. It regulates vascular development and oxygen transport in the blood system. In normal oxygen conditions in the cell, subunit HIF-1α is hydroxylated by prolyl hydroxylases (PHDs) and factor-inhibiting HIF-1 (FIH), and, consequently, degraded in the proteasome. However, under hypoxia conditions, the lack of oxygen inhibits the activity of PHDs and FIH. The unhydroxylated HIF-1α forms a complex with HIF-1β. As a result, the HIF transcription of target genes responsible for normal processes such as angiogenesis and erythropoiesis but also for carcinogenesis are activated. Hypoxia with increased HIFs activity is characteristic of malignant tumors. Therefore, inhibition of HIF-1 is one of the therapeutic targets [65,66]. Similarly, the inhibition of kinases, which take part in cellular functions, such as metabolism, cell cycle regulation, and differentiation, is used in cancer treatment because dysregulation of protein kinases is involved in carcinogenesis [67].

All tested flavones showed potential as membrane integrity agonists and membrane permeability inhibitors. The result of our previous research showed that 4′-methyflavone had an impact on the hydrophilic area of red blood cell membranes (RBCMs) and phosphatidylcholine model membranes (PC) [34]. Another team’s research results with the 1,2-dipalmitoyl-sn-glycero-3-phosphocholine (DPPC) model membrane were quite similar and showed that 4′-methylflavone and 4′-methyl-7-hydroxyflavone were localized in between the hydrophilic and hydrophobic core. The interaction with the membrane followed the order flavone > 4′-methylflavone > 4′-methyl-7-hydroxyflavone > 7-hydroxyflavone [68].

Likewise, the high potential of flavone 4′-methylene-*O*-*β*-d-(4″-*O*-methyl)-glucopyranoside (**5b**) and 4′-methylflavone 3-*O*-*β*-d-(4″-*O*-methyl)-glucopyranoside (**5d**) as cardioprotective and vasoprotective agents correlates with many previous studies, which showed that flavonoids affect the cardiovascular system in many ways, such as reduction of the cellular oxidative stress, alteration of proinflammatory cytokines, and modulating many pathways of intracellular signaling [6,10,69,70,71,72,73].

## 3. Materials and Methods

### 3.1. Substrates

Both biotransformation substrates 2′-hydroxy-4-methylchalcone (**3**) and 4′-methylflavone (**5**) were obtained by the previously described method of chemical synthesis [34]. 2′-Hydroxy-4-methylchalcone (**3**) was obtained with a 65.5% yield, and 4′-methylflavone (**5**) was obtained with a 70.0% yield.

The physical data, including color and form, melting point (°C), molecular ion mass, molecular formula, retention time t_R_ (min), retardation factor Rf, and NMR spectral data, of the resulting compounds **3** and **5** are presented below, in Table 1, Table 2, Table 3 and Table 4, and in the Supplementary Materials.

#### 3.1.1. 2′-Hydroxy-4-methylchalcone (**3**)

Yellow crystals, mp = 118 °C, ESISMS m/z 239.1 ([M + H]^+^, C_16_H_14_O_2_), t_R_ = 18.53, Rf = 0.98; ^1^H-NMR, see Table 1, ^13^C-NMR, see Table 2; Supplementary Materials: Figures S1–S15.

#### 3.1.2. 4′-Methylflavone (**5**)

Light-yellow crystals, mp = 110–111 °C, ESIMS m/z 237.1 ([M + H]^+^, C_16_H_12_O_2_), t_R_ = 16.81, Rf = 0.95, ^1^H-NMR, see Table 3, ^13^C-NMR, see Table 4, Supplementary Materials: Figures S93–S106.

### 3.2. Microorganisms

The biotransformations were performed using two entomopathogenic filamentous fungi strains, i.e., *I. fumosorosea* KCH J2 and *B. bassiana* KCH J1.5, which belong to the collection of the Department of Food Chemistry and Biocatalysis of the Wrocław University of Environmental and Life Sciences in Poland. A detailed description of the collection and reproduction of entomopathogenic filamentous fungi, as well as their genetic identification, were described in our previous papers [25,32].

### 3.3. Analysis

Thin-layer chromatography (TLC) and high-performance liquid chromatography (HPLC) were used to evaluate the course of the biotransformation. TLC analyses were carried out using TLC Silica gel 60/Kieselguhr F254 (0.2 mm thick) plates (Merck, Darmstadt, Germany) with a mixture of chloroform (Stanlab, Lublin, Poland) and methanol (Chempur, Piekary Śląskie, Poland) (9:1 *v*/*v*) as eluent. The products were observed without additional visualization under the ultraviolet lamp at λ = 254 and λ = 365 nm [29,30].

HPLC analyses were performed with the use of a Dionex Ultimate 3000 instrument (Thermo Fisher Scientific, Waltham, MA, USA) with a DAD-3000 diode array detector and an analytical octadecylsilica (ODS) 2 column (4.6 × 250 mm, 5 µm particle size, Waters, Milford, MA, USA) and pre-column. The mobile phase was a mixture of 0.1% aqueous acid, formic acid *v*/*v* (A), and acetonitrile (B). The gradient program was as follows: initial conditions—32.5% B in A, 4 min—40% B in A, 8 min—40% B in A, 10 min—45% B in A, 15 min—95% B in A, 18 min—95% B in A, 19 min—32.5% B in A, 23 min—32.5% B in A. The flow rate was 1 mL min^−1^, the injection volume was 5 µL, and the detection wavelengths were 254 and 280 nm. The column was operated at 30 °C. [29,30]. Data were collected with Chromeleon version 7.2 (Thermo Fisher Scientific, Waltham, MA, USA) software.

The separation of the semipreparative biotransformation products was attained with the use of 500 and 1000 µm preparative TLC silica gel plates with 10–12 μm particle size and 60 Å size of the pores (Analtech, Gehrden, Germany) with a mixture of chloroform and methanol (9:1 *v*/*v*) as the eluent. The compounds were extracted from the selected gel fractions using 20 mL ethyl acetate (Stanlab, Lublin, Poland) three times and afterward evaporated using a rotary evaporator [29,30].

NMR analyses (^1^H-NMR, ^13^C-NMR, COSY, Heteronuclear Single Quantum Correlation (HSQC), HMBC) were performed using a DRX Avance^TM^ 600 MHz NMR spectrometer (Bruker, Billerica, MA, USA). All samples were dissolved in deuterated acetone to perform analyses.

Optical rotation was measured using a digital polarimeter P-2000-Na (ABL&E-JASCO, Kraków, Poland).

The molecular formulas of all products were confirmed by analyses performed on the LC-MS 8045 SHIMADZU Triple Quadrupole Liquid Chromatograph Mass Spectrometer with an electrospray ionization (ESI) source (Shimadzu, Kyoto, Japan), as described previously with minor modifications [29,30]. The separation was achieved on the Kinetex column (2.6 µm C18 100 Å, 100 × 3 mm, Phenomenex, Torrance, CA, USA) operated at 30 °C. The mobile phase was a mixture of 0.1% aqueous formic acid *v*/*v* (A) and acetonitrile (B). The flow rate was 0.4 mL min^−1^, and the injection volume was 10 µL. The gradient program was as follows: initial conditions—80% B in A, 6.5 min—100% B, 7 min—80% B in A. The principal operating parameters for the LC-MS were set as follows: nebulizing gas flow: 3 L min^−1^, heating gas flow: 10 L min^−1^, interface temperature: 300 °C, drying gas flow: 10 L min^−1^, data acquisition range, *m*/*z* 100–500 Da; ionization mode, negative and positive. Data were collected with LabSolutions version 5.97 (Shimadzu, Kyoto, Japan) software [29,30].

### 3.4. Screening Procedure

The screening procedure evaluated the time needed for the complete conversion of substrates **3** and **5**. The modified Sabouraud medium was utilized as a growth medium for entomopathogenic filamentous fungi (10 g aminobac (purchased from BTL, Warsaw, Poland), 30 g saccharose (purchased from Chempur, Piekary Śląskie, Poland), 1 L distilled water). In the first step, the cultures of fungi strains were transferred from potato slants to 300 mL Erlenmeyer flasks with a 100 mL modified Sabouraud liquid medium. The cultures were bred on a rotary shaker (DHN, Warsaw, Poland) (140 rpm) at 25 °C for 72 h. In the second step, 0.5 mL of the pre-grown cultures were transferred to another 300 mL Erlenmeyer flasks with 100 mL modified Sabouraud liquid medium and also incubated at 25 °C for 72 h. Afterward, 10 mg of substrate **3** or **5** (dissolved in 0.5 mL of dimethyl sulfoxide (Chempur, Piekary Śląskie, Poland)) was added to each flask with the microorganism. The molar concentration of substrates **3** and **5** was 0.42 mM. The samples were collected after 3, 6, and 9 days of substrate incubation and extracted with 30 mL of ethyl acetate. Subsequently, the extracts were dried for 5 min with anhydrous magnesium sulfate (Chempur, Piekary Śląskie, Poland) and concentrated using a rotary evaporator (Heidolph, Schwabach, Germany) at 55 °C. All biotransformations were terminated after the confirmation of complete substrate conversion or lack of further substrate conversion, i.e., 9 days from the start. The collected samples were analyzed by chromatographic methods (TLC and HPLC described in 3.3) to check the composition of the post-reaction mixture. At the same time, the stability of the substrates under biotransformation conditions and the cultivation of the microorganisms without substrate were investigated as controls [29,30].

### 3.5. The Semipreparative Biotransformations

For each biotransformation in the semipreparative scale, a 2 L flask with 500 mL of the modified Sabouraud medium was used. The scale was adequate to obtain high enough amounts of product to perform NMR analyses and determine chemical structures.

In the first step, 1 mL of the preincubation culture of the filamentous fungi was transferred to the flask, which was subsequently incubated for 72 h under the same conditions as during the screening procedure. In the next step, 50 mg of substrate **3** or **5** (dissolved in 2.0 mL of dimethyl sulfoxide) was added to the flask. The molar concentration of substrates **3** and **5** was 0.42 mM. The incubation of the reaction mixture on the rotary shaker was continued for 9 days. The experiment was ended after the confirmation of complete substrate conversion (or lack of further substrate conversion). The flavonoid products were extracted from the post-reaction mixture 3 times with 300 mL of ethyl acetate. In the next step, the joined extracts were dried for 5 min with anhydrous magnesium sulfate, filtered, and finally dried on a rotary evaporator. The separation and purification of the microbial transformation products were achieved using preparative TLC plates. The fractions of products were marked under an ultraviolet lamp, separated, extracted (3 times with portions of 20 mL of ethyl acetate), and then analyzed by spectroscopic methods. The yields of performed biotransformations were determined based on the masses of the isolated products [29,30].

The physical data, including color and form, melting point (°C), molecular ion mass, molecular formula, retention time t_R_ (min), retardation factor Rf, and NMR spectral data of the resulting compounds **3a**–**3c** and **5a**–**5d** are presented below, in Table 1, Table 2, Table 3 and Table 4, and in the Supplementary Materials.

#### 3.5.1. 2′-Hydroxy-4-methyldihydrochalcone 5′-*O*-β-d-(4″-*O*-methyl)-glucopyranoside (**3a**)

Light-yellow crystals, mp = 124–125 °C, ESIMS m/z 431.1 ([M − H]^−^, C_23_H_28_O_8_), t_R_ = 12.45, Rf = 0.67; ^1^H-NMR, see Table 1, ^13^C-NMR, see Table 2, Supplementary Materials: Figures S21–S40.

#### 3.5.2. 4-Hydroxymethyldihydrochalcone 2′-*O*-β-d-(4″-*O*-methyl)-glucopyranoside (**3b**)

Light-yellow crystals, mp = 116–117 °C, ESIMS m/z 431.1 ([M − H]^−^, C_23_H_28_O_8_), t_R_ = 4.26, Rf = 0.57; ^1^H-NMR, see Table 1, ^13^C-NMR, see Table 2, Supplementary Materials: Figures S46–S64.

#### 3.5.3. 2′-Hydroxy-4-hydroxymethylchalcone 5′-*O*-β-d-(4″-*O*-methyl)-glucopyranoside (**3c**)

Light-yellow crystals, mp = 213–215 °C, ESIMS m/z 447.2 ([M + H]^+^, C_23_H_26_O_9_), t_R_ = 5.06, Rf = 0.45; ^1^H-NMR, see Table 1, ^13^C-NMR, see Table 2, Supplementary Materials: Figures S70–S87.

#### 3.5.4. 4′-Hydroxymethylflavone (**5a**)

Light-yellow crystals, mp = 61–63 °C ESIMS m/z 253.1 ([M + H]^+^, C_16_H_12_O_3_), t_R_ = 7.88, Rf = 0.82, ^1^H-NMR, see Table 3, ^13^C-NMR, see Table 4, Supplementary Materials: Figures S112–S124.

#### 3.5.5. Flavone 4′-methylene-*O*-β-d-(4″-*O*-methyl)-glucopyranoside (**5b**)

Light-yellow crystals, mp = 213–214 °C ESIMS m/z 429.1 ([M + H]^+^, C_23_H_24_O_8_), t_R_ = 4.60, Rf = 0.55, ^1^H-NMR, see Table 3, ^13^C-NMR, see Table 4, Supplementary Materials: Figures S130–S146.

#### 3.5.6. Flavone 4′-carboxylic acid (**5c**)

Light-yellow crystals, mp = 260–262 °C ESIMS m/z 267.0 ([M + H]^+^, C_16_H_10_O_4_), t_R_ = 9.28, Rf = 0.41, ^1^H-NMR, see Table 3, ^13^C-NMR, see Table 4, Supplementary Materials: Figures S152–S162.

#### 3.5.7. 4′-Methylflavone 3-*O*-β-d-(4″-*O*-methyl)-glucopyranoside (**5d**)

Light-yellow crystals, mp = 158–160 °C ESIMS m/z 429.2 ([M + H]^+^, C_23_H_24_O_8_), t_R_ = 9.17, Rf = 0.73, ^1^H-NMR, see Table 3, ^13^C-NMR, see Table 4, Supplementary Materials: Figures S168–S184.

### 3.6. Pharmacokinetics, Drug Nature, Biological Activity Prediction

The predictions of pharmacokinetic and physicochemical properties, medicinal chemistry friendliness, and potential biological activity of flavonoid derivatives on the basis of their structural formulae were computed using the SwissADME and Way2Drug Pass Online with accompanying services (accessed date: 20 April 2022). The structures of the molecules were built by ACD Chemsketch 2021.2.0 and saved in a .mol format and in this form, imported into both programs. The biological activity types in Pass Online are shown as the probability to be revealed (Pa) and not to be revealed (Pi) and are independent values in the range from 0 to 1. The results of predictions are shown in the Supplementary Materials: Figures S16–S20, S41–S45, S65–S69, S88–S92, S125–S129, S147–S151, S163–S167, and S185–S189.

## 4. Conclusions

In the presented work, two flavonoid compounds, i.e., 2′-hydroxy-4-methylchalcone and 4′-methylflavone, were synthesized and biotransformed in the cultures of *I. fumosorosea* KCH J2 and *B. bassiana* KCH J1.5, which are two strains of entomopathogenic filamentous fungi. 2′-Hydroxy-4-methylchalcone was biotransformed in the culture of *I. fumosorosea* KCH J2 into two products of its glycosylation and reduction of the double bond, i.e., 2′-hydroxy-4-methyldihydrochalcone 5′-*O*-*β*-d-(4″-*O*-methyl)-glucopyranoside and 4-hydroxymethyldihydrochalcone 2′-*O*-*β*-d-(4″-*O*-methyl)-glucopyranoside. On the other hand, *B. bassiana* KCH J1.5 microbial transformation resulted in the formation of only one glycosylated product, i.e., 2′-hydroxy-4-hydroxymethylchalcone 5′-*O*-*β*-d-(4″-*O*-methyl)-glucopyranoside. The simulations carried out with the use of the SwissADME platform showed that these glycoside derivatives were obtained for the first time and had an increased water solubility compared to the biotransformation substrate. Their potential biological activity predicted by the Way2Drug Pass Online tool includes antimicrobial, anticarcinogenic, chemopreventive, hepatoprotective, and membrane integrity factors.

The second tested compound, 4′-methylflavone, was successfully biotransformed only in the culture of *I. fumosorosea* KCH J2. The fungal enzyme system catalyzed the hydroxylation of the methyl moiety at C-4′ of the flavonoid B ring to form the 4′-hydroxymethylflavone intermediate. Subsequently, the methylglucosyl moiety was attached to this hydroxyl group resulting in flavone 4′-methylene-*O*-*β*-d-(4″-*O*-methyl)-glucopyranoside. This strain was also able to catalyze further oxidation of 4′-hydroxymethylflavone to yield flavone 4′-carboxylic acid and to glycosylate 4′-hydroxymethylflavone at C-3 of the C ring to form 4′-methylflavone 3-*O*-*β*-d-(4″-*O*-methyl)-glucopyranoside. The two glycosylated products have not been previously described in the literature. The Swiss-ADME simulations confirmed the increased water solubility and high gastrointestinal absorption of these flavonoid derivatives. The predictions made using the Way2Drug Pass Online tool demonstrated that these compounds are, for example, potential membrane permeability inhibitors and membrane integrity agonists, vasoprotectors, and cardioprotectants. They also exhibit great potential as antiviral and antibacterial agents. Nonetheless, the actual biological activity potential and bioavailability of the obtained flavonoids need further evaluation in in vitro and in vivo tests. Our next aim is to run in vitro studies of the obtained compounds focused on their interaction with biological membranes, liposomes, and human albumin. These studies will continue our previous studies with 2′-hydroxy-4-methylchalcone, 4′-methylflavanone, and 4′-methylflavone, and will allow us to compare results for flavonoid aglycones and glycosides [34].

## Figures and Tables

**Table 1:** ^1^H-NMR chemical shifts δ (ppm) and coupling constants *J* (Hz) of 2′-hydroxy-4-methylchalcone (**3**) and products of its biotransformation **3a**–**3c** in Acetone-d6, 600 MHz (Supplementary Materials: Figures S2, S3, S22–S24, S47–4S9 and S71–S73).

Proton	Compound			
	3	3a	3b	3c
H-α	7.93 (d) *J* = 15.4	3.42 (m)	3.41 (m)	7.97 (d) *J* = 15.4
H-β	8.02 (d) *J* = 15.4	3.00 (t) *J* = 7.5	2.97 (t) *J* = 7.5	7.93 (d) *J* = 15.4
H-2	7.79 (d) *J* = 8.0	7.19 (d) *J* = 8.0	7.26 (d) *J* = 8.3	7.89 (d) *J* = 8.2
H-3	7.31 (d) *J* = 7.9	7.09 (d) *J* = 7.8	7.23 (d) *J* = 8.3	7.48 (d) *J* = 8.2
H-5	7.31 (d) *J* = 7.9	7.09 (d) *J* = 7.8	7.23 (d) *J* = 8.3	7.48 (d) *J* = 8.2
H-6	7.79 (d) *J* = 8.0	7.19 (d) *J* = 8.0	7.26 (d) *J* = 8.3	7.89 (d) *J* = 8.2
H-3′	6.99 (t) *J* = 8.4	6.87 (d) *J* = 9.0	7.29 (dd) *J* = 8.4, *J* = 0.7	6.92 (d) *J* = 9.0
H-4′	7.57 (m)	7.28 (dd) *J* = 9.0 *J* = 3.0	7.47 (ddd) *J* = 8.4, *J* = 7.3, *J* = 1.8	7.33 (dd) *J* = 9.0 *J* = 2.9
H-5′	6.99 (t) *J* = 8.4	-	7.10 (td) *J* = 7.6 *J* = 1.0	-
H-6′	8.27 (d) *J* = 8.2	7.64 (d) *J* = 2.9	7.57 (dd) *J* = 7.7 *J* = 1.8	7.91 (d) *J* = 3.0
H-1″	-	4.83 (d) *J* = 7.8	5.06 (d) *J* = 7.8	4.92 (d) *J* = 7.8
H-2″	-	3.42 (m)	3.50 (m)	3.47 (m)
H-3″	-	3.59 (m)	3.63 (td) *J* = 9.0, *J* = 4.3	3.64 (m)
H-4″	-	3.15 (dd) *J* = 9.7, *J* = 8.9	3.21 (dd) *J* = 9.6, *J* = 9.1	3.15 (m)
H-5″	-	3.47 (m)	3.50 (m)	3.60 (ddd) *J* = 9.6, *J* = 6.1, *J* = 2.1
H-6”	-	3.84 (m) 3.67 (m)	3.84 (ddd) *J* = 11.6 *J* = 5.3 *J* = 2.2 3.68 (m)	3.93 (ddd) *J* = 11.5, *J* = 5.3, *J* = 1.5 3.73 (dt) *J* = 11.9 *J* = 6.2
C4″-OCH_3_	-	3.55 (s)	3.56 (s)	3.57 (s)
C2′-OH	12.94 (s)	11.95 (s)	-	12.60 (s)
C4-CH_3_	2.39 (s)	2.27 (s)	-	-
2″-OH	-	4.62 (d) *J* = 2.6	4.60 (d) *J* = 4.2	4.65 (d) *J* = 3.8
3″-OH	-	4.39 (d) *J* = 3.5	4.50 (d) *J* = 4.4	4.45 (d) *J* = 3.6
6″-OH	-	3.84 (m)	3.74 (dd) *J* = 6.8 *J* = 5.4	4.05 (m)
4-CH_2_-	-	-	4.58 (d) *J* = 5.7	4.70 (m)
4-CH_2_-OH	-	-	4.08 (t) *J* = 5.8	4.39 (m)

**Table 2:** ^13^C-NMR chemical shifts δ (ppm) and coupling constants *J* (Hz) of 2′-hydroxy-4-methylchalcone (**3**) and products of its biotransformation **3a**–**3c** in Acetone-d6, 151 MHz (Supplementary Materials: Figures S4, S5, S25–S27, S50–S52 and S74–S76).

Carbon	Compound			
	3	3a	3b	3c
C-α	120.4	40.4	45.7	120.8
C-β	146.4	30.4	30.6	146.5
C-1	142.4	138.8	140.9	134.2
C-2	130.0	129.3	127.6	130.1
C-3	130.6	129.8	129.1	127.8
C-4	133.0	136.1	141.2	147.0
C-5	130.6	129.8	129.1	127.8
C-6	130.0	129.3	127.6	130.1
C-1′	120.9	119.9	130.7	120.4
C-2′	164.5	158.5	157.1	159.9
C-3′	118.9	119.4	117.1	119.5
C-4′	137.4	127.5	133.9	127.2
C-5′	119.8	150.8	123.1	150.9
C-6′	131.3	118.2	130.3	117.9
C-1″	-	102.8	102.2	102.9
C-2″	-	74.9	75.0	75.0
C-3″	-	78.0	78.1	78.1
C-4″	-	80.4	80.1	80.6
C-5″	-	77.2	77.2	77.2
C-6″	-	62.3	62.1	62.5
4″-OCH_3_	-	60.6	60.6	60.6
C=O	195.1	206.9	202.4	194.7
4-CH_3_	21.5	21.0	-	-
4-CH_2_-	-	-	64.6	64.3

**Table 3:** ^1^H-NMR chemical shifts δ (ppm) and coupling constants *J* (Hz) of 4′-methylflavone (**5**) and products of its biotransformation **5a**–**5d** in Acetone-d6, 600 MHz (Supplementary Materials: Figures S94, S95, S113, S114, S131–S133, S153, S154 and S169–S171).

Proton	Compound				
	5	5a	5b	5c	5d
H-3	6.82 (s)	6.86 (s)	6.88 (s)	6.99 (s)	-
H-5	8.12 (dd) *J* = 7.9, *J* = 1.6	8.13 (dd) *J* = 7.9, *J* = 1.6	8.13 (dd) *J* = 7.8, *J* = 1.7	8.14 (dd) *J* = 7.9, *J* = 1.6	8.19 (dd) *J* = 8.0, *J* = 1.7
H-6	7.48 (m)	7.50 (m)	7.50 (m)	7.52 (m)	7.53 (ddd) *J* = 8.0, *J* = 7.1, *J* = 0.9
H-7	7.81 (ddd) *J* = 8.7, *J* = 7.1 *J* = 1.7	7.82 (ddd) *J* = 8.6, *J* = 7.1 *J* = 1.7	7.83 (ddd) *J* = 8.7, *J* = 7.1, *J* = 1.7	7.85 (ddd) *J* = 8.6, *J* = 7.1 *J* = 1.7	7.85 (ddd) *J* = 8.7, *J* = 7.1 *J* = 1.7
H-8	7.73 (d) *J* = 8.4	7.75 (d) *J* = 8.4	7.75 (dd) *J* = 8.4, *J* = 0.8	7.79 (dd) *J* = 8.4, *J* = 0.6	7.74 (d) *J* = 8.4
H-2′	7.99 (d) *J* = 8.3	8.08 (d) *J* = 8.4	8.09 (d) *J* = 8.4	8.26 (m)	8.17 (d) *J* = 8.3
H-3′	7.41 (d) *J* = 8.0	7.59 (d) *J* = 8.6	7.65 (d) *J* = 8.4	8.23 (m)	7.37 (d) *J* = 8.1
H-5′	7.41 (d) *J* = 8.0	7.59 (d) *J* = 8.6	7.65 (d) *J* = 8.4	8.23 (m)	7.37 (d) *J* = 8.1
H-6′	7.99 (d) *J* = 8.3	8.08 (d) *J* = 8.4	8.09 (d) *J* = 8.4	8.26 (m)	8.17 (d) *J* = 8.3
H-1″	-	-	4.42 (d) *J* = 7.8	-	5.22 (d) *J* = 7.9
H-2″	-	-	3.29 (m)	-	3.40 (m)
H-3″	-	-	3.52 (m)	-	3.58 (m)
H-4″	-	-	3.13 (dd) *J* = 9.5, *J* = 9.0	-	3.11 (m)
H-5″	-	-	3.29 (m)	-	3.20 (ddd) *J* = 9.7, *J* = 5.0, *J* = 2.4
H-6″	-	-	3.83 (d) *J* = 10.5 3.69 (m)	-	3.55 (m) 3.48 (dd) *J* = 7.0, *J* = 4.9
C4″-OCH_3_	-	-	3.54 (s)	-	3.51 (s)
C4′-CH_3_	2.43 (s)	-	-	-	2.43 (s)
2″-OH	-	-	-	-	5.33 (d) *J* = 2.3
3″-OH	-	-	-	-	4.36 (d) *J* = 3.5
6″-OH	-	-	3.66 (d) *J* = 4.6	-	3.55 (m)
4′-CH_2_-	-	4.76 (d) *J* = 5.8	5.00 (d) *J* = 13.0 4.76 (d) *J* = 13.0	-	-
4′-CH_2_-OH	-	4.47 (t) *J* = 5.8	-	-	-

**Table 4:** ^13^C-NMR chemical shifts δ (ppm) and coupling constants *J* (Hz) of 4′-methylflavone (**5**) and products of its biotransformations **5a**–**5d** in Acetone-d6, 151 MHz (Supplementary Materials: Figures S96, S97, S115, S116, S134–S136, S155, S156 and S172–S174).

Carbon	Compound				
	5	5a	5b	5c	5d
C-2	164.1	164.0	163.8	162.8	158.5
C-3	107.4	107.6	107.8	109.2	138.3
C-4	177.9	177.9	177.9	177.9	175.6
C-4a	124.9	124.9	124.9	124.9	124.5
C-5	126.0	126.0	126.0	126.0	126.2
C-6	126.1	126.1	126.2	126.4	126.0
C-7	134.7	134.8	134.8	135.1	135.1
C-8	119.2	119.3	119.3	119.4	119.2
C-8a	157.2	157.2	157.1	157.2	156.3
C-1′	123.0	131.2	131.7	136.8	129.1
C-2′	127.2	127.1	127.1	127.4	130.3
C-3′	130.6	127.8	128.9	131.1	129.6
C-4′	143.1	147.6	143.4	134.0	142.2
C-5′	130.6	127.8	128.9	131.1	129.6
C-6′	127.2	127.1	127.1	127.4	130.3
C-1″	-	-	103.3	-	104.7
C-2″	-	-	75.2	-	75.6
C-3″	-	-	78.1	-	78.3
C-4″	-	-	80.5	-	79.9
C-5″	-	-	77.0	-	77.2
C-6″	-	-	62.4	-	62.2
4″-OCH_3_	-	-	60.5	-	60.5
4′-CH_3_	21.4	-	-	-	21.5
4′-CH_2_-	-	64.1	70.3	-	-
4′-COOH	-	-	-	166.9	-

## Data Availability

Samples of the compounds **3**, **3a**–**3c** and **5**, **5a**–**5d** are available from the authors.

## Supplementary Materials

The following supporting information can be downloaded at: https://www.mdpi.com/article/10.3390/ijms23105558/s1.
