# Supplementary material for: Glycosylation of Methylflavonoids in the Cultures of Entomopathogenic Filamentous Fungi as a Tool for Obtaining New Biologically Active Compounds
Source: Int J Mol Sci. 2022 May 16;23(10):5558. doi: 10.3390/ijms23105558 (PMC9146141; doi:10.3390/ijms23105558)
Supplement: Supplementary file 1 [file ijms-23-05558-s001.zip › ijms-1728304-supplementary.pdf]

Supplementary materials

Glycosylation of methylflavonoids in the cultures of entomopathogenic filamentous fungi as a tool for obtaining new biologically active compounds

Agnieszka Krawczyk-Łebek*, Monika Dymarska, Tomasz Janeczko and Edyta Kostrzewa-Susłow*

Department of Food Chemistry and Biocatalysis, Faculty of Biotechnology and Food Science, Wrocław University of Environmental and Life Sciences, Wrocław, Poland
*Correspondence: agnieszka.krawczyk-lebek@upwr.edu.pl, edyta.kostrzewa-suslow@upwr.edu.pl

Content

Figure S1. MS analysis of 2′-hydroxy-4-methylchalcone (**3**)
Figure S2. ^{1}H NMR spectrum (*δ,* acetone-d6, 600 MHz) of 2′-hydroxy-4-methylchalcone (**3**)
Figure S3 ^{1}H NMR spectrum expansion (*δ,* acetone-d6, 600 MHz) of 2′-hydroxy-4-methylchalcone (**3**)
Figure S4. ^{13}C NMR spectrum (*δ,* acetone-d6, 151 MHz) of 2′-hydroxy-4-methylchalcone (**3**)
Figure S5. ^{13}C NMR spectrum expansion (*δ,* acetone-d6, 151 MHz) of 2′-hydroxy-4-methylchalcone (**3**)
Figure S6. COSY contour map – ^{1}H x ^{1}H of 2′-hydroxy-4-methylchalcone (**3**)
Figure S7. COSY contour map – ^{1}H x ^{1}H expansion of 2′-hydroxy-4-methylchalcone (**3**)
Figure S8. COSY contour map – ^{1}H x ^{1}H expansion of 2′-hydroxy-4-methylchalcone (**3**)
Figure S9. HSQC contour map – ^{1}H x ^{13}C of 2′-hydroxy-4-methylchalcone (**3**)
Figure S10. HSQC contour map – ^{1}H x ^{13}C expansion of 2′-hydroxy-4-methylchalcone (**3**)
Figure S11. HSQC contour map – ^{1}H x ^{13}C expansion of 2′-hydroxy-4-methylchalcone (**3**)
Figure S12. HMBC contour map – ^{1}H x ^{13}C of 2′-hydroxy-4-methylchalcone (**3**)
Figure S13. HMBC contour map – ^{1}H x ^{13}C expansion of 2′-hydroxy-4-methylchalcone (**3**)
Figure S14. HMBC contour map – ^{1}H x ^{13}C expansion of 2′-hydroxy-4-methylchalcone (**3**)
Figure S15. HMBC contour map – ^{1}H x ^{13}C expansion of 2′-hydroxy-4-methylchalcone (**3**)
Figure S16. 2′-Hydroxy-4-methylchalcone (**3**) physicochemical and ADME parameters prediction using the SwissADME modelling
Figure S17. 2′-Hydroxy-4-methylchalcone (**3**) physicochemical biological activity prediction using the Way2Drug Pass online modelling
Figure S18. 2′-Hydroxy-4-methylchalcone (**3**) antibacterial activity prediction using the Way2Drug AntiBac-Pred modelling
Figure S19. 2′-Hydroxy-4-methylchalcone (**3**) antifungal activity prediction using the Way2Drug AntiFun-Pred modelling
Figure S20. 2′-Hydroxy-4-methylchalcone (**3**) antiviral activity prediction using the Way2Drug AntiVir-Pred modelling
Figure S21. MS analysis 2′-hydroxy-4-methyldihydrochalcone 5′-*O*-*β*-D-(4″-*O*-methyl)-glucopyranoside (**3a**)

Figure S22. ^{1}H NMR spectrum (*δ,* acetone-d6, 600 MHz) of 2′-hydroxy-4-methyldihydrochalcone 5′-*O*-*β*-D-(4″-*O*-methyl)-glucopyranoside (**3a**)
Figure S23. ^{1}H NMR spectrum expansion (*δ,* acetone-d6, 600 MHz) of 2′-hydroxy-4-methyldihydrochalcone 5′-*O*-*β*-D-(4″-*O*-methyl)-glucopyranoside (**3a**)
Figure S24. ^{1}H NMR spectrum expansion (*δ,* acetone-d6, 600 MHz) of 2′-hydroxy-4-methyldihydrochalcone 5′-*O*-*β*-D-(4″-*O*-methyl)-glucopyranoside (**3a**)
Figure S25. ^{13}C NMR spectrum expansion (*δ,* acetone-d6, 151 MHz) of 2′-hydroxy-4-methyldihydrochalcone 5′-*O*-*β*-D-(4″-*O*-methyl)-glucopyranoside (**3a**)
Figure S26. ^{13}C NMR spectrum expansion (*δ,* acetone-d6, 151 MHz) of 2′-hydroxy-4-methyldihydrochalcone 5′-*O*-*β*-D-(4″-*O*-methyl)-glucopyranoside (**3a**)
Figure S27. ^{13}C NMR spectrum expansion (*δ,* acetone-d6, 151 MHz) of 2′-hydroxy-4-methyldihydrochalcone 5′-*O*-*β*-D-(4″-*O*-methyl)-glucopyranoside (**3a**)
Figure S28. COSY contour map – ^{1}H x ^{1}H of 2′-hydroxy-4-methyldihydrochalcone 5′-*O*-*β*-D-(4″-*O*-methyl)-glucopyranoside (**3a**)
Figure S29. COSY contour map – ^{1}H x ^{1}H expansion of 2′-hydroxy-4-methyldihydrochalcone 5′-*O*-*β*-D-(4″-*O*-methyl)-glucopyranoside (**3a**)
Figure S30. COSY contour map – ^{1}H x ^{1}H expansion of 2′-hydroxy-4-methyldihydrochalcone 5′-*O*-*β*-D-(4″-*O*-methyl)-glucopyranoside (**3a**)
Figure S31. HSQC contour map – ^{1}H x ^{13}C of 2′-hydroxy-4-methyldihydrochalcone 5′-*O*-*β*-D-(4″-*O*-methyl)-glucopyranoside (**3a**)
Figure S32. HSQC contour map – ^{1}H x ^{13}C expansion of 2′-hydroxy-4-methyldihydrochalcone 5′-*O*-*β*-D-(4″-*O*-methyl)-glucopyranoside (**3a**)
Figure S33. HSQC contour map – ^{1}H x ^{13}C expansion of 2′-hydroxy-4-methyldihydrochalcone 5′-*O*-*β*-D-(4″-*O*-methyl)-glucopyranoside (**3a**)
Figure S34. HMBC contour map – ^{1}H x ^{13}C of 2′-hydroxy-4-methyldihydrochalcone 5′-*O*-*β*-D-(4″-*O*-methyl)-glucopyranoside (**3a**)
Figure S35. HMBC contour map – ^{1}H x ^{13}C expansion of 2′-hydroxy-4-methyldihydrochalcone 5′-*O*-*β*-D-(4″-*O*-methyl)-glucopyranoside (**3a**)
Figure S36. HMBC contour map – ^{1}H x ^{13}C expansion of 2′-hydroxy-4-methyldihydrochalcone 5′-*O*-*β*-D-(4″-*O*-methyl)-glucopyranoside (**3a**)
Figure S37. HMBC contour map – ^{1}H x ^{13}C expansion of 2′-hydroxy-4-methyldihydrochalcone 5′-*O*-*β*-D-(4″-*O*-methyl)-glucopyranoside (**3a**)
Figure S38. HMBC contour map – ^{1}H x ^{13}C expansion of 2′-hydroxy-4-methyldihydrochalcone 5′-*O*-*β*-D-(4″-*O*-methyl)-glucopyranoside (**3a**)
Figure S39. HMBC contour map – ^{1}H x ^{13}C expansion of 2′-hydroxy-4-methyldihydrochalcone 5′-*O*-*β*-D-(4″-*O*-methyl)-glucopyranoside (**3a**)
Figure S40. HMBC contour map – ^{1}H x ^{13}C expansion of 2′-hydroxy-4-methyldihydrochalcone 5′-*O*-*β*-D-(4″-*O*-methyl)-glucopyranoside (**3a**)
Figure S41. 2′-Hydroxy-4-methyldihydrochalcone 5′-*O*-*β*-D-(4″-*O*-methyl)-glucopyranoside (**3a**) physicochemical and ADME parameters prediction using the SwissADME modelling

Figure S42. 2′-Hydroxy-4-methyldihydrochalcone 5′-*O*-*β*-D-(4″-*O*-methyl)-glucopyranoside (**3a**) biological activity prediction using the Way2Drug Pass online modelling
Figure S43. 2′-Hydroxy-4-methyldihydrochalcone 5′-*O*-*β*-D-(4″-*O*-methyl)-glucopyranoside (**3a**) antibacterial activity prediction using the Way2Drug AntiBac-Pred modelling
Figure S44. 2′-Hydroxy-4-methyldihydrochalcone 5′-*O*-*β*-D-(4″-*O*-methyl)-glucopyranoside (**3a**) antifungal activity prediction using the Way2Drug AntiFun-Pred modelling
Figure S45. 2′-Hydroxy-4-methyldihydrochalcone 5′-*O*-*β*-D-(4″-*O*-methyl)-glucopyranoside (**3a**) antiviral activity prediction using the Way2Drug AntiVir-Pred modelling
Figure S46. MS analysis 4-hydroxymethyldihydrochalcone 2′-*O*-*β*-D-(4″-*O*-methyl)-glucopyranoside (**3b**)
Figure S47. ^{1}H NMR spectrum (*δ,* acetone-d6, 600 MHz) of 4-hydroxymethyldihydrochalcone 2′-*O*-*β*-D-(4″-*O*-methyl)-glucopyranoside (**3b**)
Figure S48. ^{1}H NMR spectrum expansion (*δ,* acetone-d6, 600 MHz) of 4-hydroxymethyldihydrochalcone 2′-*O*-*β*-D-(4″-*O*-methyl)-glucopyranoside (**3b**)
Figure S49. ^{1}H NMR spectrum expansion (*δ,* acetone-d6, 600 MHz) of 4-hydroxymethyldihydrochalcone 2′-*O*-*β*-D-(4″-*O*-methyl)-glucopyranoside (**3b**)
Figure S50. ^{13}C NMR spectrum expansion (*δ,* acetone-d6, 151 MHz) of 4-hydroxymethyldihydrochalcone 2′-*O*-*β*-D-(4″-*O*-methyl)-glucopyranoside (**3b**)
Figure S51. ^{13}C NMR spectrum expansion (*δ,* acetone-d6, 151 MHz) of 4-hydroxymethyldihydrochalcone 2′-*O*-*β*-D-(4″-*O*-methyl)-glucopyranoside (**3b**)
Figure S52. ^{13}C NMR spectrum expansion (*δ,* acetone-d6, 151 MHz) of 4-hydroxymethyldihydrochalcone 2′-*O*-*β*-D-(4″-*O*-methyl)-glucopyranoside (**3b**)
Figure S53. COSY contour map – ^{1}H x ^{1}H of 4-hydroxymethyldihydrochalcone 2′-*O*-*β*-D-(4″-*O*-methyl)-glucopyranoside (**3b**)
Figure S54. COSY contour map – ^{1}H x ^{1}H expansion of 4-hydroxymethyldihydrochalcone 2′-*O*-*β*-D-(4″-*O*-methyl)-glucopyranoside (**3b**)
Figure S55. COSY contour map – ^{1}H x ^{1}H expansion of 4-hydroxymethyldihydrochalcone 2′-*O*-*β*-D-(4″-*O*-methyl)-glucopyranoside (**3b**)
Figure S56. HSQC contour map – ^{1}H x ^{13}C of 4-hydroxymethyldihydrochalcone 2′-*O*-*β*-D-(4″-*O*-methyl)-glucopyranoside (**3b**)
Figure S57. HSQC contour map – ^{1}H x ^{13}C expansion of 4-hydroxymethyldihydrochalcone 2′-*O*-*β*-D-(4″-*O*-methyl)-glucopyranoside (**3b**)
Figure S58. HSQC contour map – ^{1}H x ^{13}C expansion of 4-hydroxymethyldihydrochalcone 2′-*O*-*β*-D-(4″-*O*-methyl)-glucopyranoside (**3b**)
Figure S59. HMBC contour map – ^{1}H x ^{13}C of 4-hydroxymethyldihydrochalcone 2′-*O*-*β*-D-(4″-*O*-methyl)-glucopyranoside (**3b**)
Figure S60. HMBC contour map – ^{1}H x ^{13}C expansion of 4-hydroxymethyldihydrochalcone 2′-*O*-*β*-D-(4″-*O*-methyl)-glucopyranoside (**3b**)
Figure S61. HMBC contour map – ^{1}H x ^{13}C expansion of 4-hydroxymethyldihydrochalcone 2′-*O*-*β*-D-(4″-*O*-methyl)-glucopyranoside (**3b**)
Figure S62. HMBC contour map – ^{1}H x ^{13}C expansion of 4-hydroxymethyldihydrochalcone 2′-*O*-*β*-D-(4″-*O*-methyl)-glucopyranoside (**3b**)

Figure S63. HMBC contour map – ^{1}H x ^{13}C expansion of 4-hydroxymethyldihydrochalcone 2′-*O*-*β*-D-(4″-*O*-methyl)-glucopyranoside (**3b**)
Figure S64. HMBC contour map – ^{1}H x ^{13}C expansion of 4-hydroxymethyldihydrochalcone 2′-*O*-*β*-D-(4″-*O*-methyl)-glucopyranoside (**3b**)
Figure S65. 4-Hydroxymethyldihydrochalcone 2′-*O*-*β*-D-(4″-*O*-methyl)-glucopyranoside (**3b**) physicochemical and ADME parameters prediction using the SwissADME modelling
Figure S66. 4-Hydroxymethyldihydrochalcone 2′-*O*-*β*-D-(4″-*O*-methyl)-glucopyranoside (**3b**) biological activity prediction using the Way2Drug Pass online modelling
Figure S67. 4-Hydroxymethyldihydrochalcone 2′-*O*-*β*-D-(4″-*O*-methyl)-glucopyranoside (**3b**) antibacterial activity prediction using the Way2Drug AntiBac-Pred modelling
Figure S68. 4-Hydroxymethyldihydrochalcone 2′-*O*-*β*-D-(4″-*O*-methyl)-glucopyranoside (**3b**) antifungal activity prediction using the Way2Drug AntiFun-Pred modelling
Figure S69. 4-Hydroxymethyldihydrochalcone 2′-*O*-*β*-D-(4″-*O*-methyl)-glucopyranoside (**3b**) antiviral activity prediction using the Way2Drug AntiVir-Pred modelling
Figure S70. MS analysis 2′-hydroxy-4-hydroxymethylchalcone 5′-*O*-*β*-D-(4″-*O*-methyl)-glucopyranoside (**3c**)
Figure S71. ^{1}H NMR spectrum (*δ,* acetone-d6, 600 MHz) of 2′-hydroxy-4-hydroxymethylchalcone 5′-*O*-*β*-D-(4″-*O*-methyl)-glucopyranoside (**3c**)
Figure S72. ^{1}H NMR spectrum expansion (*δ,* acetone-d6, 600 MHz) of 2′-hydroxy-4-hydroxymethylchalcone 5′-*O*-*β*-D-(4″-*O*-methyl)-glucopyranoside (**3c**)
Figure S73. ^{1}H NMR spectrum expansion (*δ,* acetone-d6, 600 MHz) of 2′-hydroxy-4-hydroxymethylchalcone 5′-*O*-*β*-D-(4″-*O*-methyl)-glucopyranoside (**3c**)
Figure S74. ^{13}C NMR spectrum expansion (*δ,* acetone-d6, 151 MHz) of 2′-hydroxy-4-hydroxymethylchalcone 5′-*O*-*β*-D-(4″-*O*-methyl)-glucopyranoside (**3c**)
Figure S75. ^{13}C NMR spectrum expansion (*δ,* acetone-d6, 151 MHz) of 2′-hydroxy-4-hydroxymethylchalcone 5′-*O*-*β*-D-(4″-*O*-methyl)-glucopyranoside (**3c**)
Figure S76. ^{13}C NMR spectrum expansion (*δ,* acetone-d6, 151 MHz) of 2′-hydroxy-4-hydroxymethylchalcone 5′-*O*-*β*-D-(4″-*O*-methyl)-glucopyranoside (**3c**)
Figure S77. COSY contour map – ^{1}H x ^{1}H of 2′-hydroxy-4-hydroxymethylchalcone 5′-*O*-*β*-D-(4″-*O*-methyl)-glucopyranoside (**3c**)
Figure S78. COSY contour map – ^{1}H x ^{1}H expansion of 2′-hydroxy-4-hydroxymethylchalcone 5′-*O*-*β*-D-(4″-*O*-methyl)-glucopyranoside (**3c**)
Figure S79. COSY contour map – ^{1}H x ^{1}H expansion of 2′-hydroxy-4-hydroxymethylchalcone 5′-*O*-*β*-D-(4″-*O*-methyl)-glucopyranoside (**3c**)
Figure S80. HSQC contour map – ^{1}H x ^{13}C of 2′-hydroxy-4-hydroxymethylchalcone 5′-*O*-*β*-D-(4″-*O*-methyl)-glucopyranoside (**3c**)
Figure S81. HSQC contour map – ^{1}H x ^{13}C expansion of 2′-hydroxy-4-hydroxymethylchalcone 5′-*O*-*β*-D-(4″-*O*-methyl)-glucopyranoside (**3c**)
Figure S82. HSQC contour map – ^{1}H x ^{13}C expansion of 2′-hydroxy-4-hydroxymethylchalcone 5′-*O*-*β*-D-(4″-*O*-methyl)-glucopyranoside (**3c**)
Figure S83. HMBC contour map – ^{1}H x ^{13}C of 2′-hydroxy-4-hydroxymethylchalcone 5′-*O*-*β*-D-(4″-*O*-methyl)-glucopyranoside (**3c**)

Figure S84. HMBC contour map – ^{1}H x ^{13}C expansion of 2′-hydroxy-4-hydroxymethylchalcone 5′-*O*-*β*-D-(4″-*O*-methyl)-glucopyranoside (**3c**)
Figure S85. HMBC contour map – ^{1}H x ^{13}C expansion of 2′-hydroxy-4-hydroxymethylchalcone 5′-*O*-*β*-D-(4″-*O*-methyl)-glucopyranoside (**3c**)
Figure S86. HMBC contour map – ^{1}H x ^{13}C expansion of 2′-hydroxy-4-hydroxymethylchalcone 5′-*O*-*β*-D-(4″-*O*-methyl)-glucopyranoside (**3c**)
Figure S87. HMBC contour map – ^{1}H x ^{13}C expansion of 2′-hydroxy-4-hydroxymethylchalcone 5′-*O*-*β*-D-(4″-*O*-methyl)-glucopyranoside (**3c**)
Figure S88. 2′-Hydroxy-4-hydroxymethylchalcone 5′-*O*-*β*-D-(4″-*O*-methyl)-glucopyranoside (**3c**) physicochemical and ADME parameters prediction using the SwissADME modelling
Figure S89. 2′-Hydroxy-4-hydroxymethylchalcone 5′-*O*-*β*-D-(4″-*O*-methyl)-glucopyranoside (**3c**) biological activity prediction using the Way2Drug Pass online modelling
Figure S90. 2′-Hydroxy-4-hydroxymethylchalcone 5′-*O*-*β*-D-(4″-*O*-methyl)-glucopyranoside (**3c**) antibacterial activity prediction using the Way2Drug AntiBac-Pred modelling
Figure S91. 2′-Hydroxy-4-hydroxymethylchalcone 5′-*O*-*β*-D-(4″-*O*-methyl)-glucopyranoside (**3c**) antifungal activity prediction using the Way2Drug AntiFun-Pred modelling
Figure S92. 2′-Hydroxy-4-hydroxymethylchalcone 5′-*O*-*β*-D-(4″-*O*-methyl)-glucopyranoside (**3c**) antiviral activity prediction using the Way2Drug AntiVir-Pred modelling
Figure S93. MS analysis of 4′-methyflavone (**5**)
Figure S94. ^{1}H NMR spectrum (*δ*, acetone-d6, 600 MHz) of 4′-methyflavone (**5**)
Figure S95. ^{1}H NMR spectrum expansion (*δ*, acetone-d6, 600 MHz) of 4′-methyflavone (**5**)
Figure S96. ^{13}C NMR spectrum (*δ*, acetone-d6, 151 MHz) of 4′-methyflavone (**5**)
Figure S97. ^{13}C NMR spectrum expansion (*δ*, acetone-d6, 151 MHz) of 4′-methyflavone (**5**)
Figure S98. COSY contour map – ^{1}H x ^{1}H of 4′-methyflavone (**5**)
Figure S99. COSY contour map – ^{1}H x ^{1}H expansion of 4′-methyflavone (**5**)
Figure S100. COSY contour map – ^{1}H x ^{1}H expansion of 4′-methyflavone (**5**)
Figure S101. HSQC contour map – ^{1}H x ^{13}C of 4′-methyflavone (**5**)
Figure S102. HSQC contour map – ^{1}H x ^{13}C expansion of 4′-methyflavone (**5**)
Figure S103. HSQC contour map – ^{1}H x ^{13}C expansion of 4′-methyflavone (**5**)
Figure S104. HMBC contour map – ^{1}H x ^{13}C of 4′-methyflavone (**5**)
Figure S105. HMBC contour map – ^{1}H x ^{13}C expansion of 4′-methyflavone (**5**)
Figure S106. HMBC contour map – ^{1}H x ^{13}C expansion of 4′-methyflavone (**5**)
Figure S107. 4′-Methyflavone (**5**) physicochemical and ADME parameters prediction using the SwissADME modelling
Figure S108. 4′-Methyflavone (**5**) physicochemical biological activity prediction using the Way2Drug Pass online modelling
Figure S109. 4′-Methyflavone (**5**) antibacterial activity prediction using the Way2Drug AntiBac-Pred modelling
Figure S110. 4′-Methyflavone (**5**) antifungal activity prediction using the Way2Drug AntiFun-Pred modelling
Figure S111. 4′-Methyflavone (**5**) antiviral activity prediction using the Way2Drug AntiVir-Pred modelling
Figure S112. MS analysis 4′-hydroxymethylflavone (**5a**)
Figure S113. ^{1}H NMR spectrum (*δ*, acetone-d6, 600 MHz) of 4′-hydroxymethylflavone (**5a**)

Figure S114. ^{1}H NMR spectrum expansion (*δ,* acetone-d6, 600 MHz) of 4′-hydroxymethylflavone (**5a**)
Figure S115. ^{13}C NMR spectrum expansion (*δ,* acetone-d6, 151 MHz) of 4′-hydroxymethylflavone (**5a**)
Figure S116. ^{13}C NMR spectrum expansion (*δ,* acetone-d6, 151 MHz) of 4′-hydroxymethylflavone (**5a**)
Figure S117. COSY contour map – ^{1}H x ^{1}H of 4′-hydroxymethylflavone (**5a**)
Figure S118. COSY contour map – ^{1}H x ^{1}H expansion of 4′-hydroxymethylflavone (**5a**)
Figure S119. HSQC contour map – ^{1}H x ^{13}C of 4′-hydroxymethylflavone (**5a**)
Figure S120. HSQC contour map – ^{1}H x ^{13}C expansion of 4′-hydroxymethylflavone (**5a**)
Figure S121. HSQC contour map – ^{1}H x ^{13}C expansion of 4′-hydroxymethylflavone (**5a**)
Figure S122. HMBC contour map – ^{1}H x ^{13}C of 4′-hydroxymethylflavone (**5a**)
Figure S123. HMBC contour map – ^{1}H x ^{13}C expansion of 4′-hydroxymethylflavone (**5a**)
Figure S124. HMBC contour map – ^{1}H x ^{13}C expansion of 4′-hydroxymethylflavone (**5a**)
Figure S125. 4′-Hydroxymethylflavone (**5a**) physicochemical and ADME parameters prediction using the SwissADME modelling
Figure S126. 4′-Hydroxymethylflavone (**5a**) biological activity prediction using the Way2Drug Pass online modelling
Figure S127. 4′-Hydroxymethylflavone (**5a**) antibacterial activity prediction using the Way2Drug AntiBac-Pred modelling
Figure S128. 4′-Hydroxymethylflavone (**5a**) antifungal activity prediction using the Way2Drug AntiFun-Pred modelling
Figure S129. 4′-Hydroxymethylflavone (**5a**) antiviral activity prediction using the Way2Drug AntiVir-Pred modelling
Figure S130. MS analysis flavone 4′-methylene-*O*-*β*-D-(4′′-*O*-methyl)-glucopyranoside (**5b**)
Figure S131. ^{1}H NMR spectrum (*δ,* acetone-d6, 600 MHz) of flavone 4′-methylene-*O*-*β*-D-(4′′-*O*-methyl)-glucopyranoside (**5b**)
Figure S132. ^{1}H NMR spectrum expansion (*δ,* acetone-d6, 600 MHz) of flavone 4′-methylene-*O*-*β*-D-(4′′-*O*-methyl)-glucopyranoside (**5b**)
Figure S133. ^{1}H NMR spectrum expansion (*δ,* acetone-d6, 600 MHz) of flavone 4′-methylene-*O*-*β*-D-(4′′-*O*-methyl)-glucopyranoside (**5b**)
Figure S134. ^{13}C NMR spectrum expansion (*δ,* acetone-d6, 151 MHz) of flavone 4′-methylene-*O*-*β*-D-(4′′-*O*-methyl)-glucopyranoside (**5b**)
Figure S135. ^{13}C NMR spectrum expansion (*δ,* acetone-d6, 151 MHz) of flavone 4′-methylene-*O*-*β*-D-(4′′-*O*-methyl)-glucopyranoside (**5b**)
Figure S136. ^{13}C NMR spectrum expansion (*δ,* acetone-d6, 151 MHz) of flavone 4′-methylene-*O*-*β*-D-(4′′-*O*-methyl)-glucopyranoside (**5b**)
Figure S137. COSY contour map – ^{1}H x ^{1}H of flavone 4′-methylene-*O*-*β*-D-(4′′-*O*-methyl)-glucopyranoside (**5b**)
Figure S138. COSY contour map – ^{1}H x ^{1}H expansion of flavone 4′-methylene-*O*-*β*-D-(4′′-*O*-methyl)-glucopyranoside (**5b**)

Figure S139. COSY contour map – ^{1}H x ^{1}H expansion of flavone 4′-methylene-*O*-*β*-D-(4′′-*O*-methyl)-glucopyranoside (**5b**)
Figure S140. HSQC contour map – ^{1}H x ^{13}C of flavone 4′-methylene-*O*-*β*-D-(4′′-*O*-methyl)-glucopyranoside (**5b**)
Figure S141. HSQC contour map – ^{1}H x ^{13}C expansion of flavone 4′-methylene-*O*-*β*-D-(4′′-*O*-methyl)-glucopyranoside (**5b**)
Figure S142. HSQC contour map – ^{1}H x ^{13}C expansion of flavone 4′-methylene-*O*-*β*-D-(4′′-*O*-methyl)-glucopyranoside (**5b**)
Figure S143. HMBC contour map – ^{1}H x ^{13}C of flavone 4′-methylene-*O*-*β*-D-(4′′-*O*-methyl)-glucopyranoside (**5b**)
Figure S144. HMBC contour map – ^{1}H x ^{13}C expansion of flavone 4′-methylene-*O*-*β*-D-(4′′-*O*-methyl)-glucopyranoside (**5b**)
Figure S145. HMBC contour map – ^{1}H x ^{13}C expansion of flavone 4′-methylene-*O*-*β*-D-(4′′-*O*-methyl)-glucopyranoside (**5b**)
Figure S146. HMBC contour map – ^{1}H x ^{13}C expansion of flavone 4′-methylene-*O*-*β*-D-(4′′-*O*-methyl)-glucopyranoside (**5b**)
Figure S147. Flavone 4′-methylene-*O*-*β*-D-(4′′-*O*-methyl)-glucopyranoside (**5b**) physicochemical and ADME parameters prediction using the SwissADME modelling
Figure S148. Flavone 4′-methylene-*O*-*β*-D-(4′′-*O*-methyl)-glucopyranoside (**5b**) biological activity prediction using the Way2Drug Pass online modelling
Figure S149. Flavone 4′-methylene-*O*-*β*-D-(4′′-*O*-methyl)-glucopyranoside (**5b**) antibacterial activity prediction using the Way2Drug AntiBac-Pred modelling
Figure S150. Flavone 4′-methylene-*O*-*β*-D-(4′′-*O*-methyl)-glucopyranoside (**5b**) antifungal activity prediction using the Way2Drug AntiFun-Pred modelling
Figure S151. Flavone 4′-methylene-*O*-*β*-D-(4′′-*O*-methyl)-glucopyranoside (**5b**) antiviral activity prediction using the Way2Drug AntiVir-Pred modelling
Figure S152. MS analysis flavone 4′-carboxylic acid (**5c**)
Figure S153. ^{1}H NMR spectrum (*δ*, acetone-d6, 600 MHz) of flavone 4′-carboxylic acid (**5c**)
Figure S154. ^{1}H NMR spectrum expansion (*δ*, acetone-d6, 600 MHz) of flavone 4′-carboxylic acid (**5c**)
Figure S155. ^{13}C NMR spectrum expansion (*δ*, acetone-d6, 151 MHz) of flavone 4′-carboxylic acid (**5c**)
Figure S156. ^{13}C NMR spectrum expansion (*δ*, acetone-d6, 151 MHz) of flavone 4′-carboxylic acid (**5c**)
Figure S157. COSY contour map – ^{1}H x ^{1}H of flavone 4′-carboxylic acid (**5c**)
Figure S158. COSY contour map – ^{1}H x ^{1}H expansion of flavone 4′-carboxylic acid (**5c**)
Figure S159. HSQC contour map – ^{1}H x ^{13}C of flavone 4′-carboxylic acid (**5c**)
Figure S160. HSQC contour map – ^{1}H x ^{13}C expansion of flavone 4′-carboxylic acid (**5c**)
Figure S161. HMBC contour map – ^{1}H x ^{13}C of flavone 4′-carboxylic acid (**5c**)
Figure S162. HMBC contour map – ^{1}H x ^{13}C expansion of flavone 4′-carboxylic acid (**5c**)
Figure S163. Flavone 4′-carboxylic acid (**5c**) physicochemical and ADME parameters prediction using the SwissADME modelling

Figure S164. Flavone 4′-carboxylic acid (**5c**) biological activity prediction using the Way2Drug Pass online modelling
Figure S165. Flavone 4′-carboxylic acid (**5c**) antibacterial activity prediction using the Way2Drug AntiBac-Pred modelling
Figure S166. Flavone 4′-carboxylic acid (**5c**) antifungal activity prediction using the Way2Drug AntiFun-Pred modelling
Figure S167. Flavone 4′-carboxylic acid (**5c**) antiviral activity prediction using the Way2Drug AntiVir-Pred modelling
Figure S168. MS analysis 4′-methylflavone 3-*O*-*β*-D-(4′′-*O*-methyl)-glucopyranoside (**5d**)
Figure S169. ^{1}H NMR spectrum (*δ,* acetone-d6, 600 MHz) of 4′-methylflavone 3-*O*-*β*-D-(4′′-*O*-methyl)-glucopyranoside (**5d**)
Figure S170. ^{1}H NMR spectrum expansion (*δ,* acetone-d6, 600 MHz) of 4′-methylflavone 3-*O*-*β*-D-(4′′-*O*-methyl)-glucopyranoside (**5d**)
Figure S171. ^{1}H NMR spectrum expansion (*δ,* acetone-d6, 600 MHz) of 4′-methylflavone 3-*O*-*β*-D-(4′′-*O*-methyl)-glucopyranoside (**5d**)
Figure S172. ^{13}C NMR spectrum expansion (*δ,* acetone-d6, 151 MHz) of 4′-methylflavone 3-*O*-*β*-D-(4′′-*O*-methyl)-glucopyranoside (**5d**)
Figure S173. ^{13}C NMR spectrum expansion (*δ,* acetone-d6, 151 MHz) of 4′-methylflavone 3-*O*-*β*-D-(4′′-*O*-methyl)-glucopyranoside (**5d**)
Figure S174. ^{13}C NMR spectrum expansion (*δ,* acetone-d6, 151 MHz) of 4′-methylflavone 3-*O*-*β*-D-(4′′-*O*-methyl)-glucopyranoside (**5d**)
Figure S175. COSY contour map – ^{1}H x ^{1}H of 4′-methylflavone 3-*O*-*β*-D-(4′′-*O*-methyl)-glucopyranoside (**5d**)
Figure S176. COSY contour map – ^{1}H x ^{1}H expansion of 4′-methylflavone 3-*O*-*β*-D-(4′′-*O*-methyl)-glucopyranoside (**5d**)
Figure S177. COSY contour map – ^{1}H x ^{1}H expansion of 4′-methylflavone 3-*O*-*β*-D-(4′′-*O*-methyl)-glucopyranoside (**5d**)
Figure S178. HSQC contour map – ^{1}H x ^{13}C of 4′-methylflavone 3-*O*-*β*-D-(4′′-*O*-methyl)-glucopyranoside (**5d**)
Figure S179. HSQC contour map – ^{1}H x ^{13}C expansion of 4′-methylflavone 3-*O*-*β*-D-(4′′-*O*-methyl)-glucopyranoside (**5d**)
Figure S180. HSQC contour map – ^{1}H x ^{13}C expansion of 4′-methylflavone 3-*O*-*β*-D-(4′′-*O*-methyl)-glucopyranoside (**5d**)
Figure S181. HMBC contour map – ^{1}H x ^{13}C of 4′-methylflavone 3-*O*-*β*-D-(4′′-*O*-methyl)-glucopyranoside (**5d**)
Figure S182. HMBC contour map – ^{1}H x ^{13}C expansion of 4′-methylflavone 3-*O*-*β*-D-(4′′-*O*-methyl)-glucopyranoside (**5d**)
Figure S183. HMBC contour map – ^{1}H x ^{13}C expansion of 4′-methylflavone 3-*O*-*β*-D-(4′′-*O*-methyl)-glucopyranoside (**5d**)
Figure S184. HMBC contour map – ^{1}H x ^{13}C expansion of 4′-methylflavone 3-*O*-*β*-D-(4′′-*O*-methyl)-glucopyranoside (**5d**)

Figure S185. 4′-Methylflavone 3-*O*-*β*-D-(4′′-*O*-methyl)-glucopyranoside (**5d**) physicochemical and ADME parameters prediction using the SwissADME modelling

Figure S186. 4′-Methylflavone 3-*O*-*β*-D-(4′′-*O*-methyl)-glucopyranoside (**5d**) biological activity prediction using the Way2Drug Pass online modelling

Figure S187. 4′-Methylflavone 3-*O*-*β*-D-(4′′-*O*-methyl)-glucopyranoside (**5d**) antibacterial activity prediction using the Way2Drug AntiBac-Pred modelling

Figure S188. 4′-Methylflavone 3-*O*-*β*-D-(4′′-*O*-methyl)-glucopyranoside (**5d**) antifungal activity prediction using the Way2Drug AntiFun-Pred modelling

Figure S189. 4′-Methylflavone 3-*O*-*β*-D-(4′′-*O*-methyl)-glucopyranoside (**5d**) antiviral activity prediction using the Way2Drug AntiVir-Pred modelling

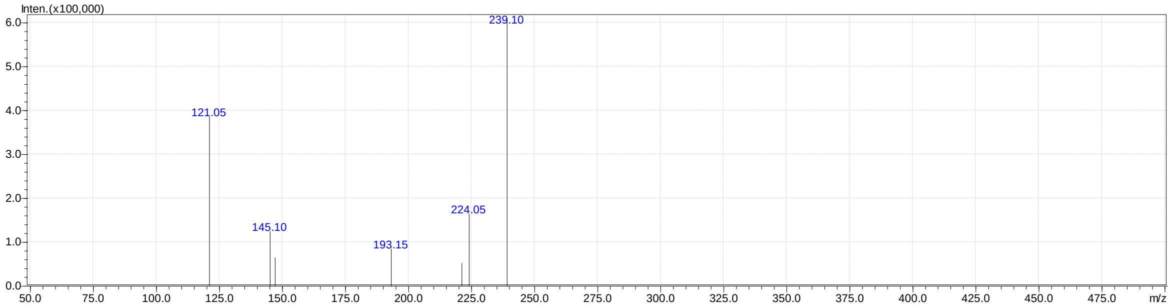

Figure S1. MS analysis of 2′-hydroxy-4-methylchalcone (**3**)

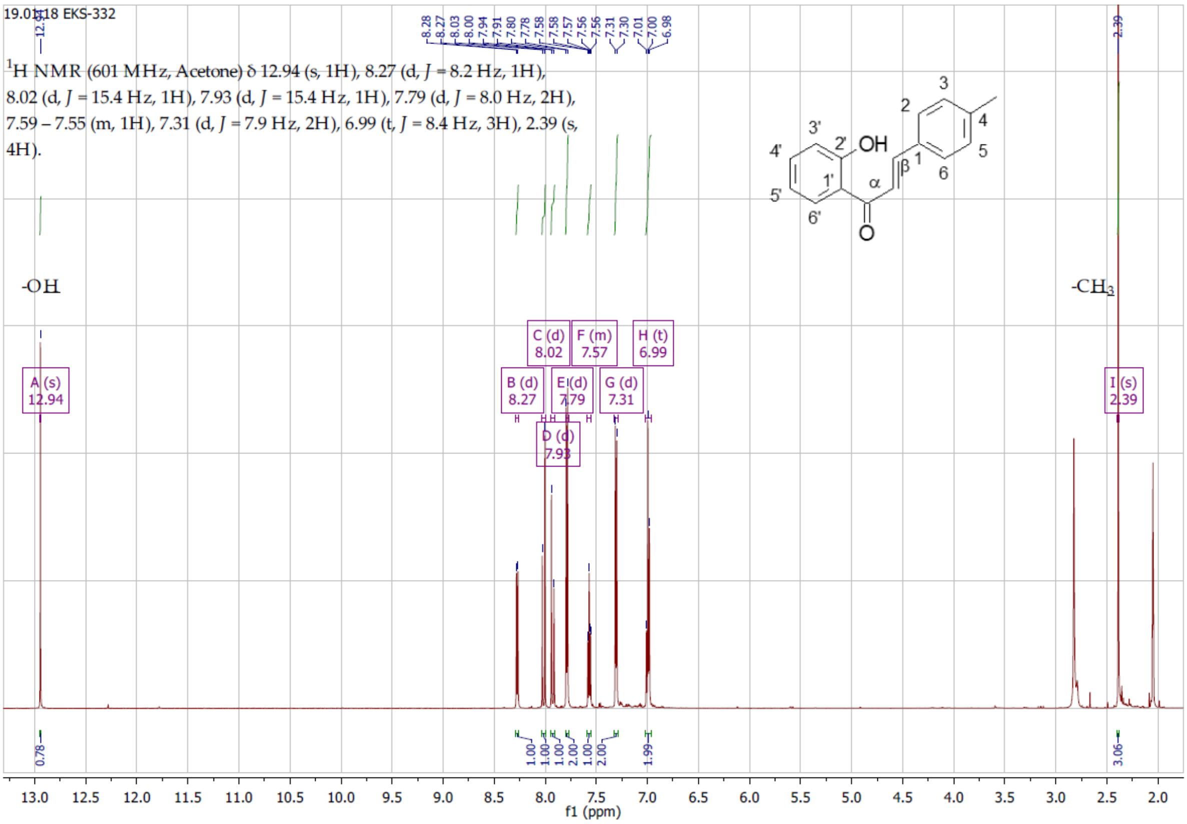

Figure S2. ^{1}H NMR spectrum (*δ*, acetone-d6, 600 MHz) of 2′-hydroxy-4-methylchalcone (**3**)

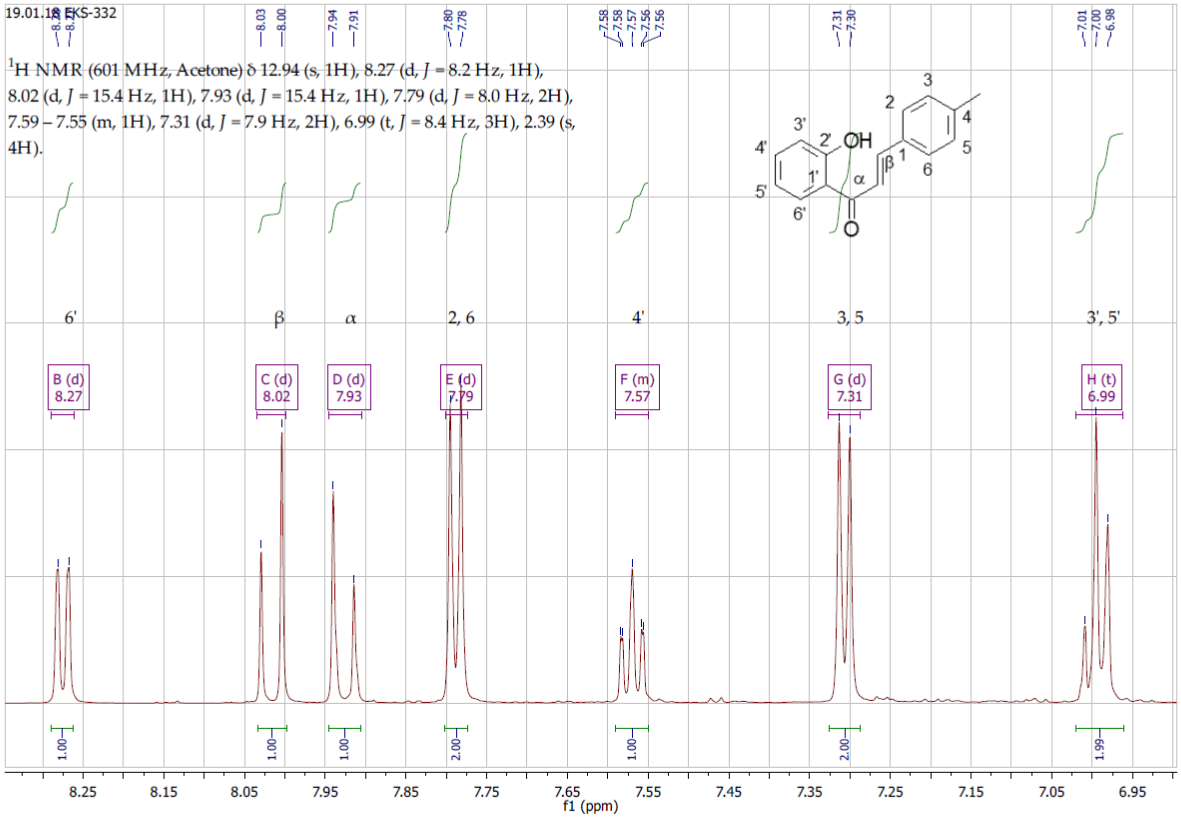

Figure S3 ^{1}H NMR spectrum expansion (δ, acetone-d6, 600 MHz) of 2′-hydroxy-4-methylchalcone (**3**)

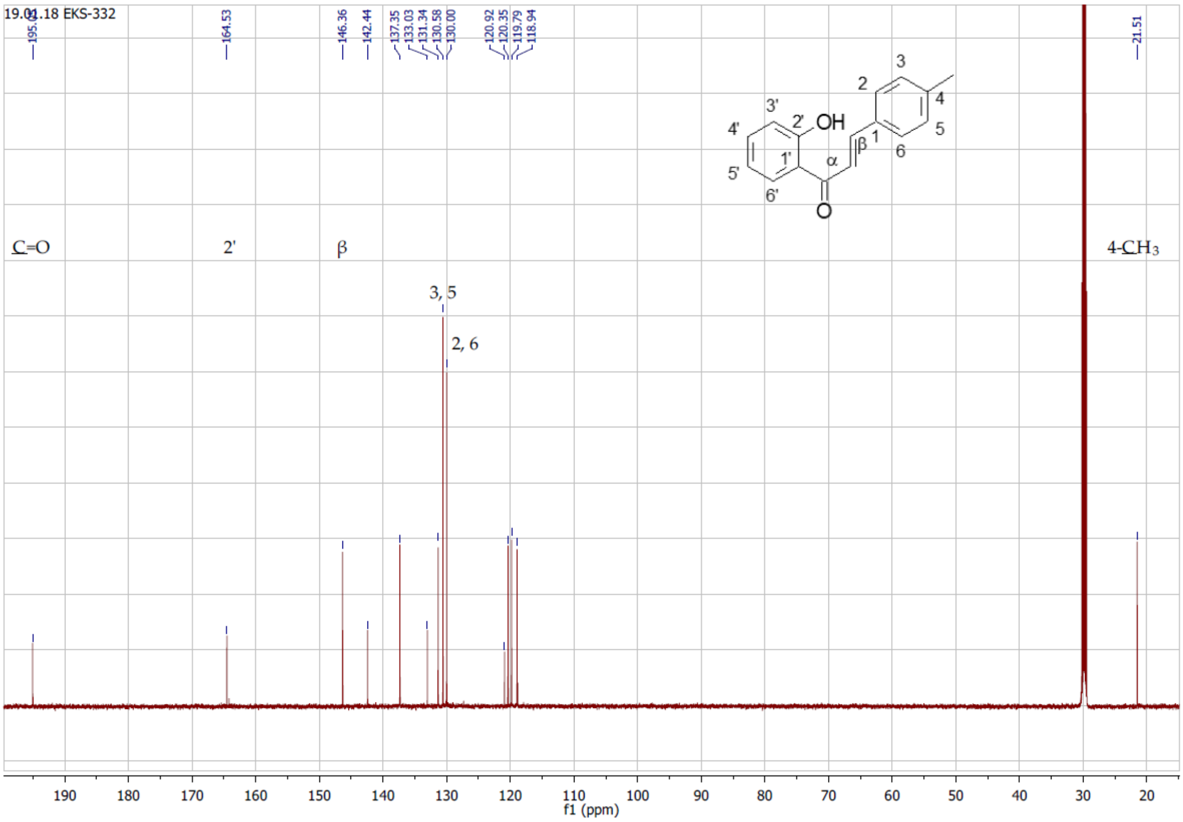

Figure S4. ^{13}C NMR spectrum (δ, acetone-d6, 151 MHz) of 2′-hydroxy-4-methylchalcone (**3**)

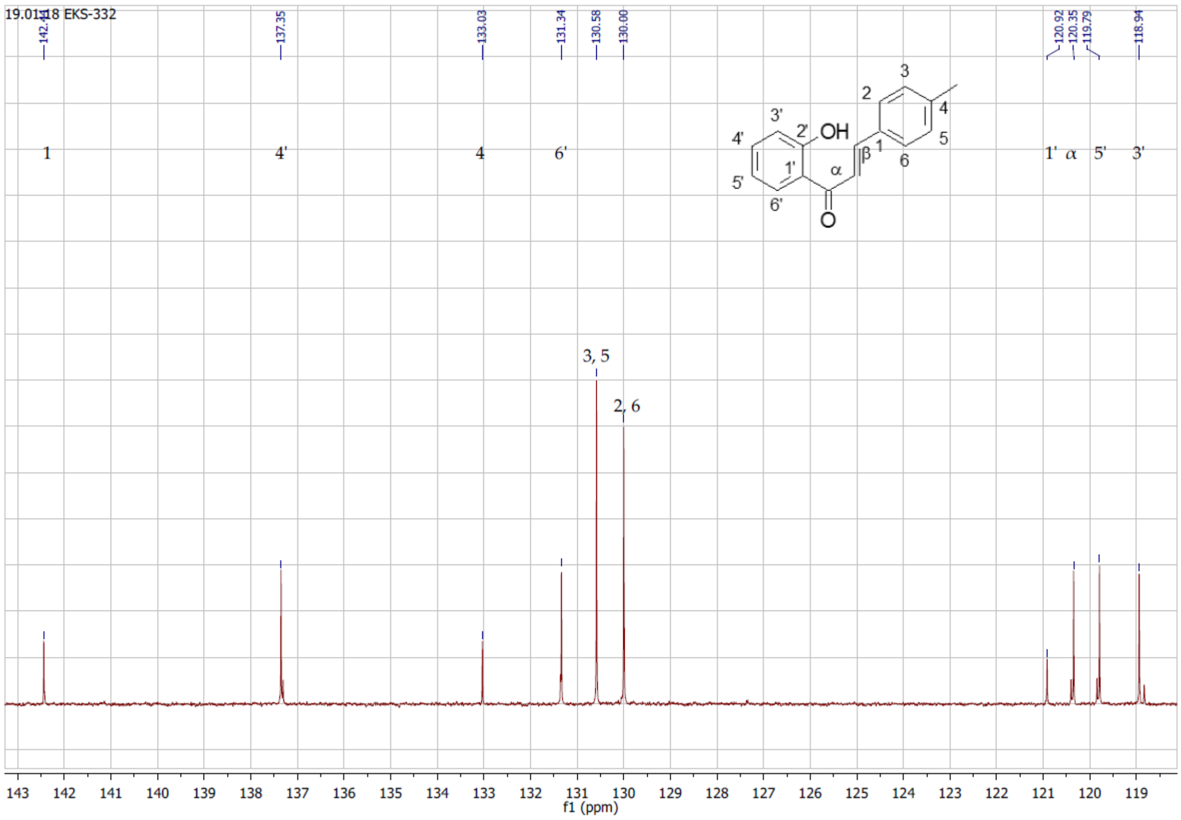

Figure S5. ^{13}C NMR spectrum expansion (δ, acetone-d6, 151 MHz) of 2′-hydroxy-4-methylchalcone (**3**)

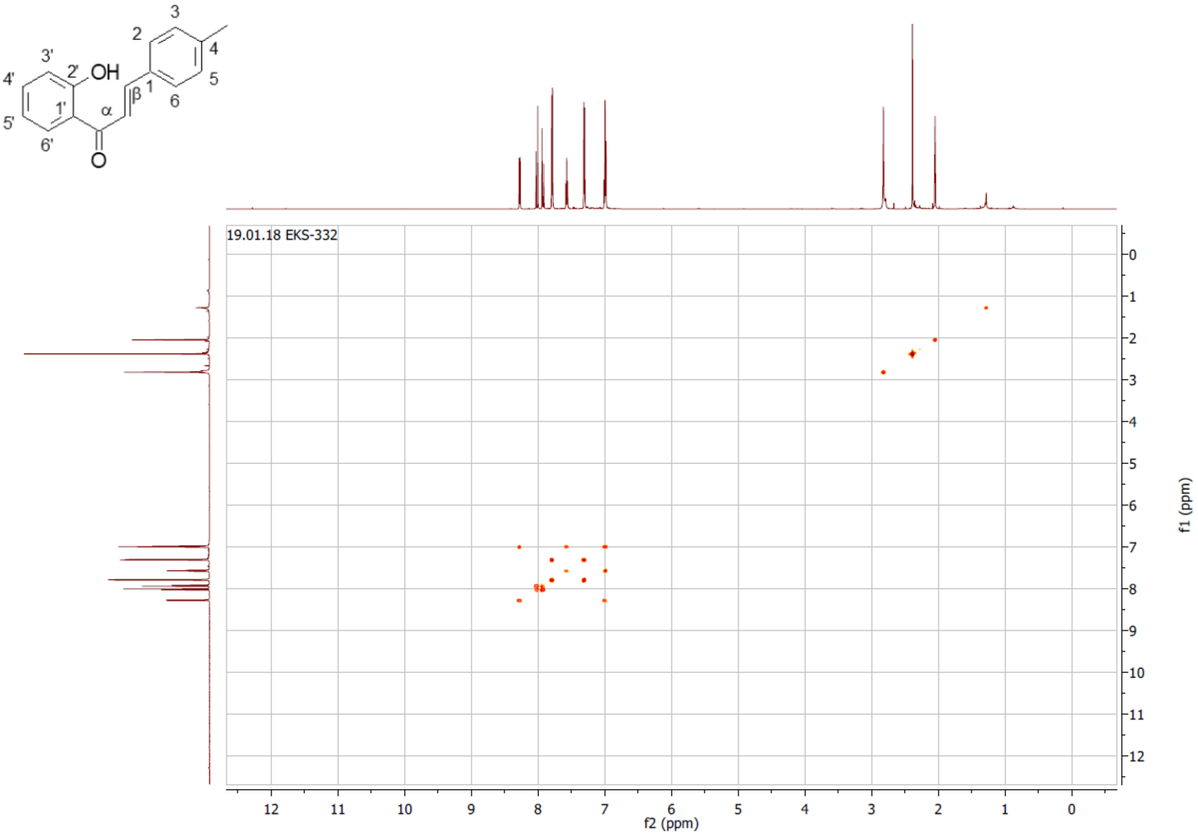

Figure S6. COSY contour map – ^1H x ^1H of 2′-hydroxy-4-methylchalcone (**3**)

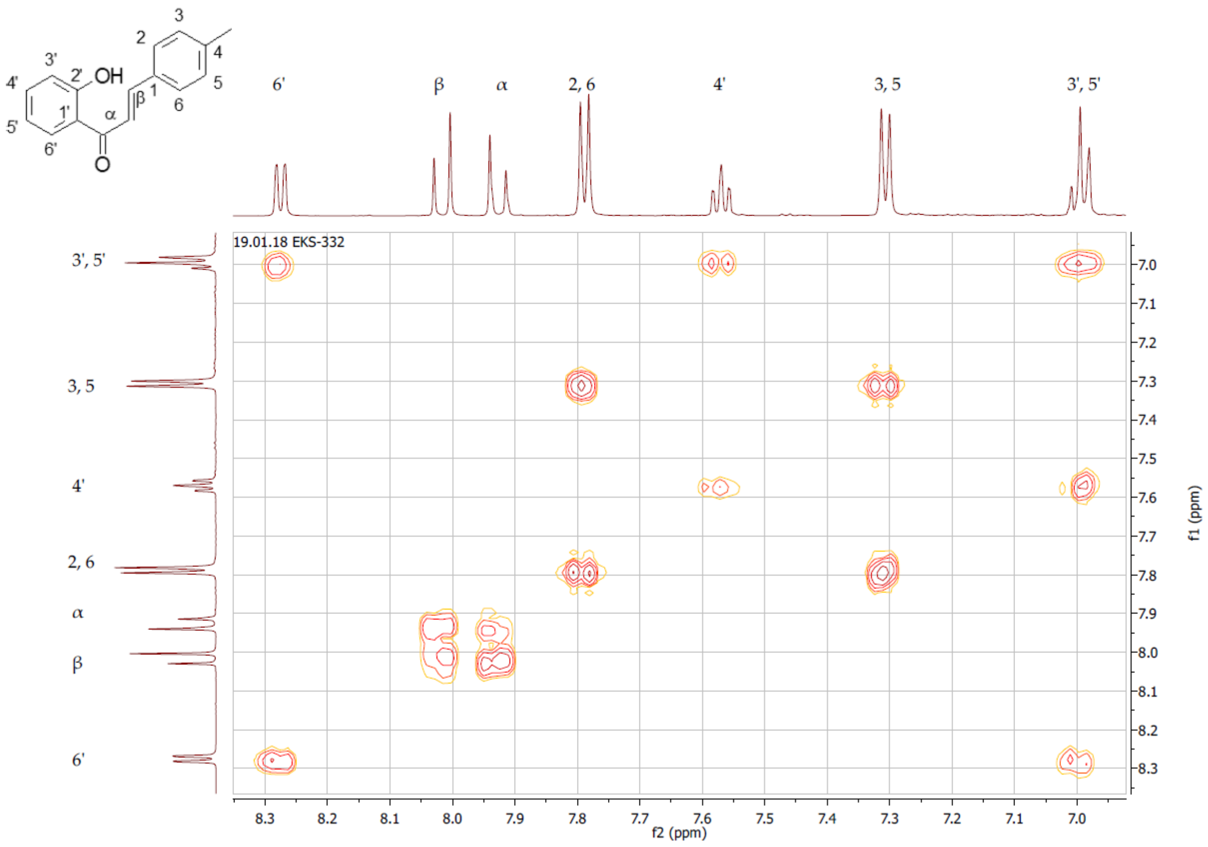

Figure S7. COSY contour map – ^1H x ^1H expansion of 2′-hydroxy-4-methylchalcone (**3**)

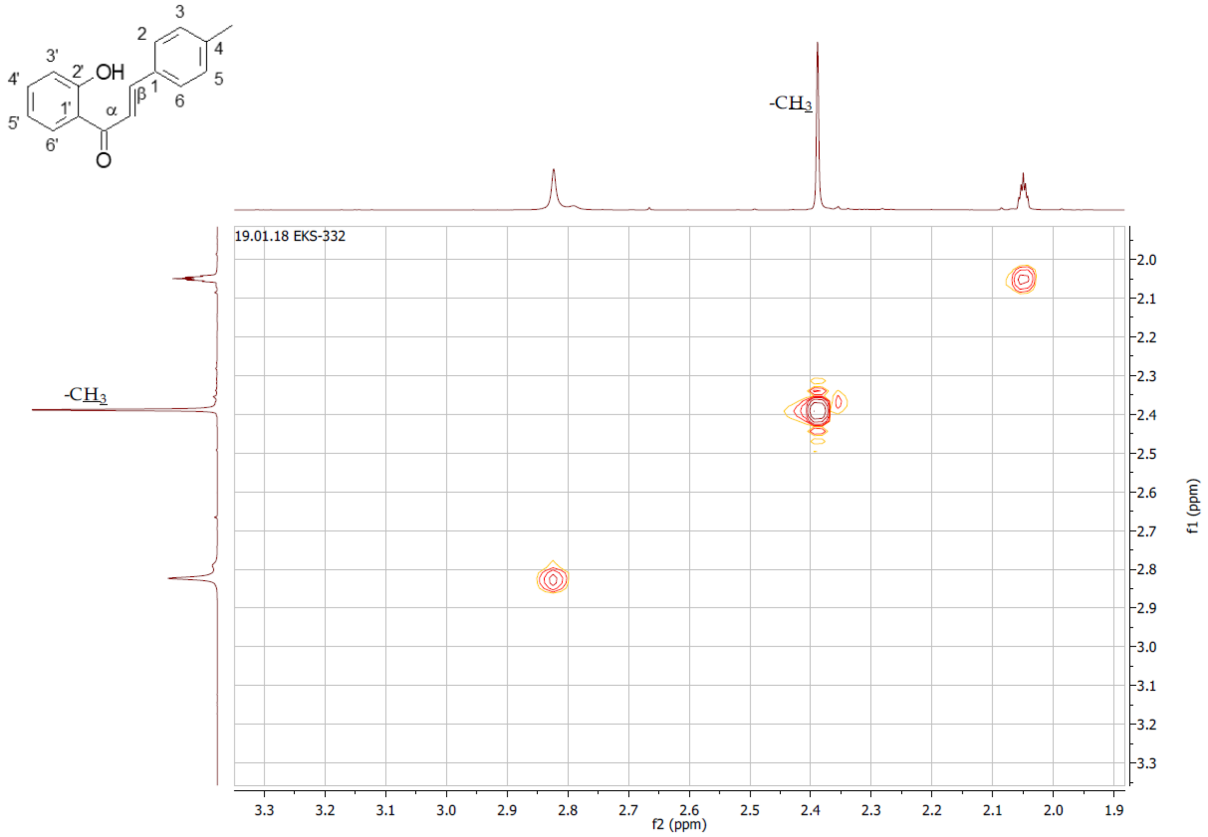

Figure S8. COSY contour map – ^1H x ^1H expansion of 2′-hydroxy-4-methylchalcone (**3**)

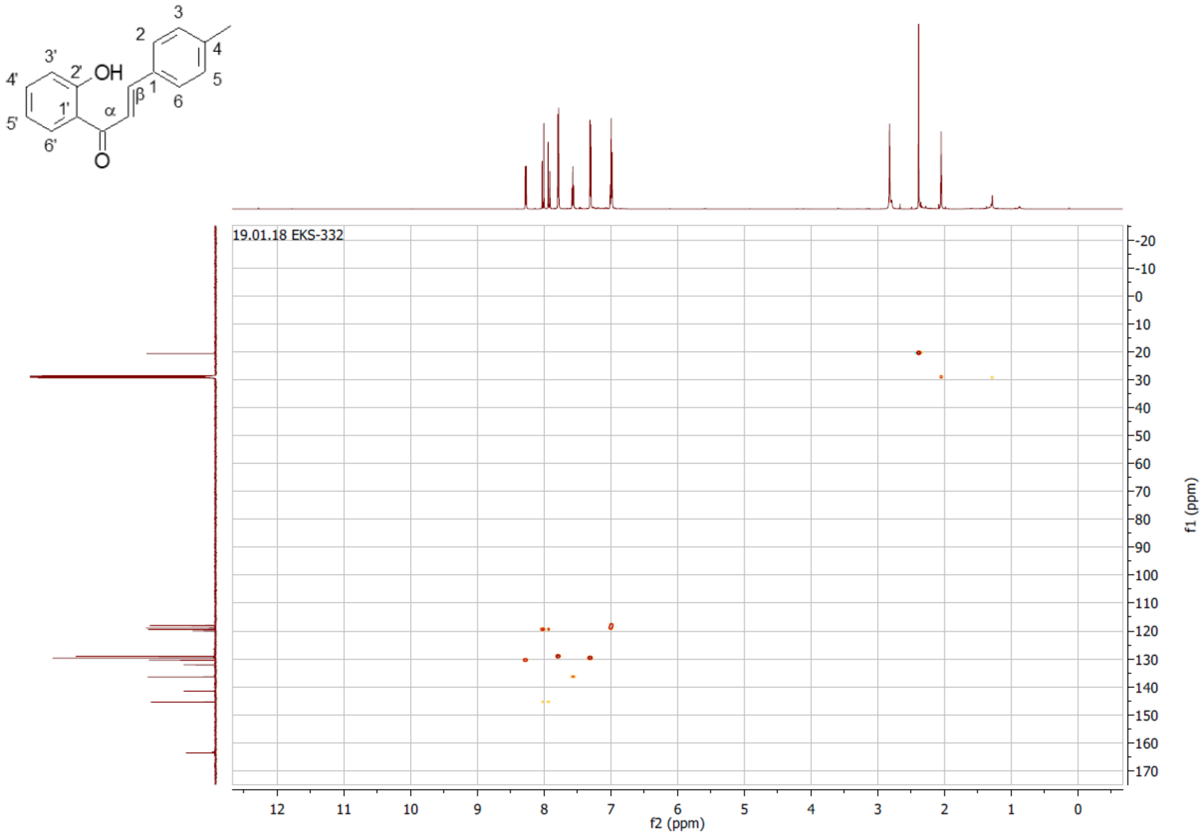

Figure S9. HSQC contour map – ^{1}H x ^{13}C of 2′-hydroxy-4-methylchalcone (**3**)

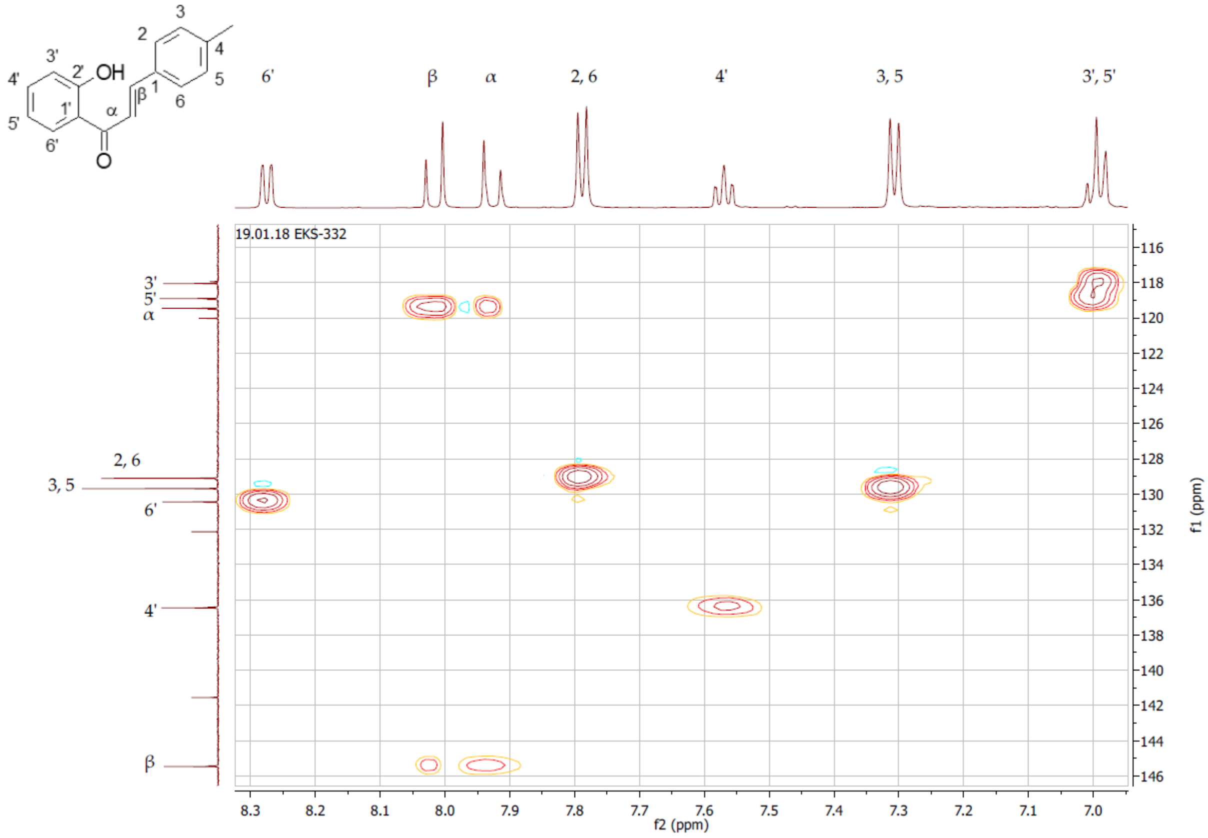

Figure S10. HSQC contour map – ^{1}H x ^{13}C expansion of 2′-hydroxy-4-methylchalcone (**3**)

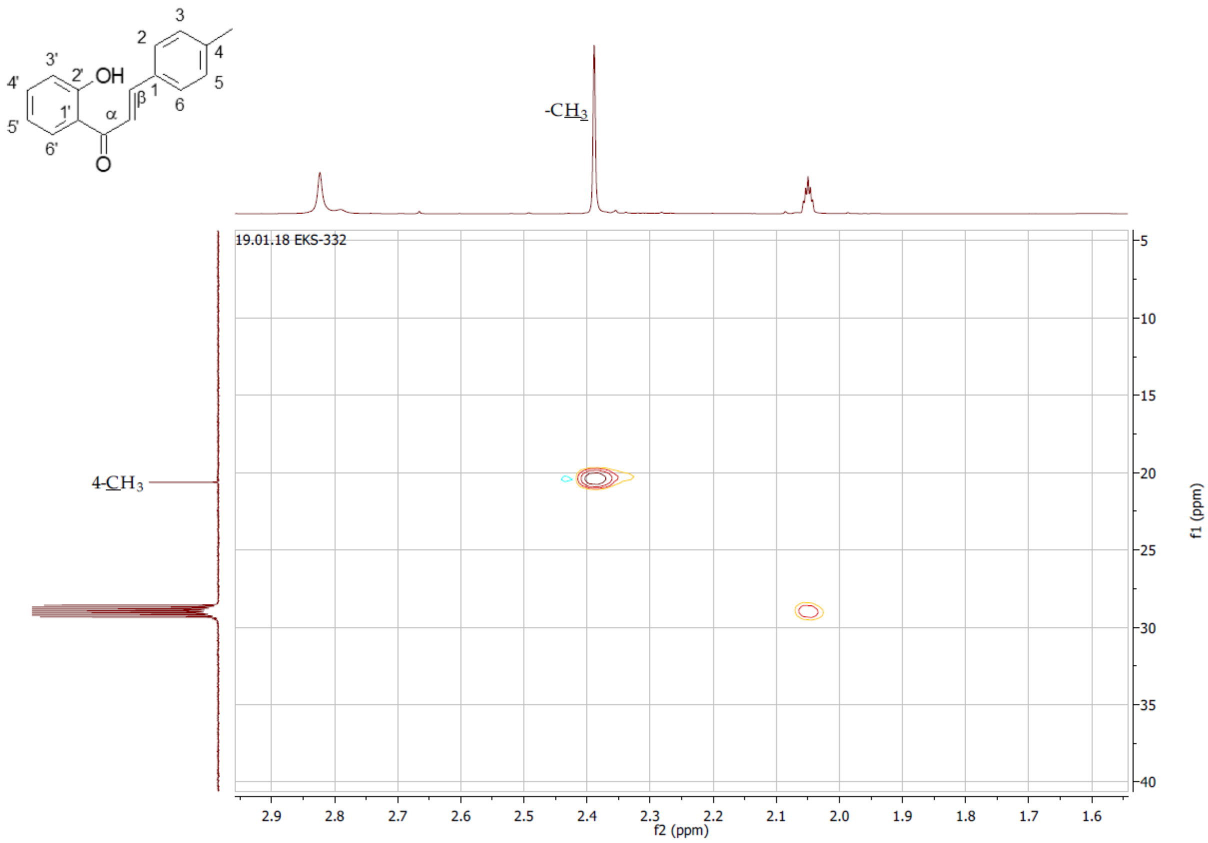

Figure S11. HSQC contour map – ^{1}H x ^{13}C expansion of 2′-hydroxy-4-methylchalcone (**3**)

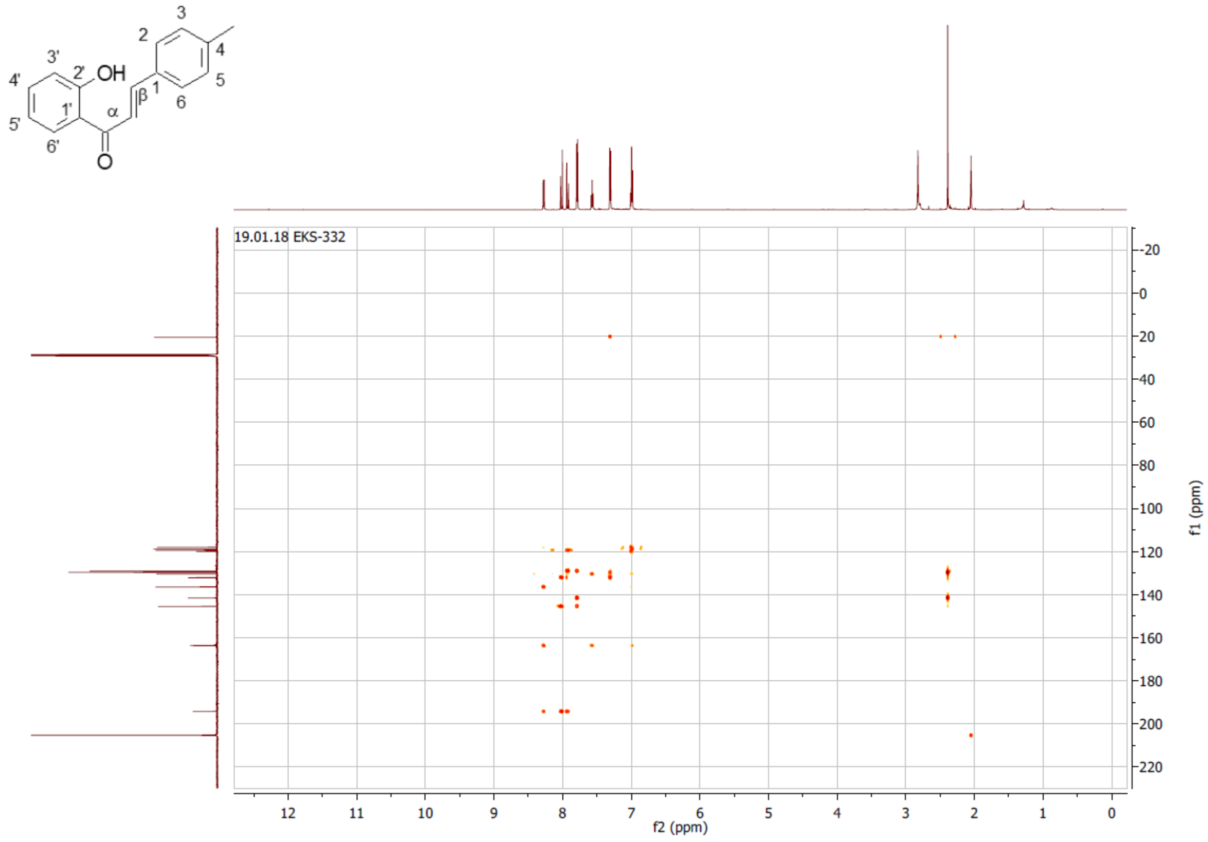

Figure S12. HMBC contour map – ^{1}H x ^{13}C of 2′-hydroxy-4-methylchalcone (**3**)

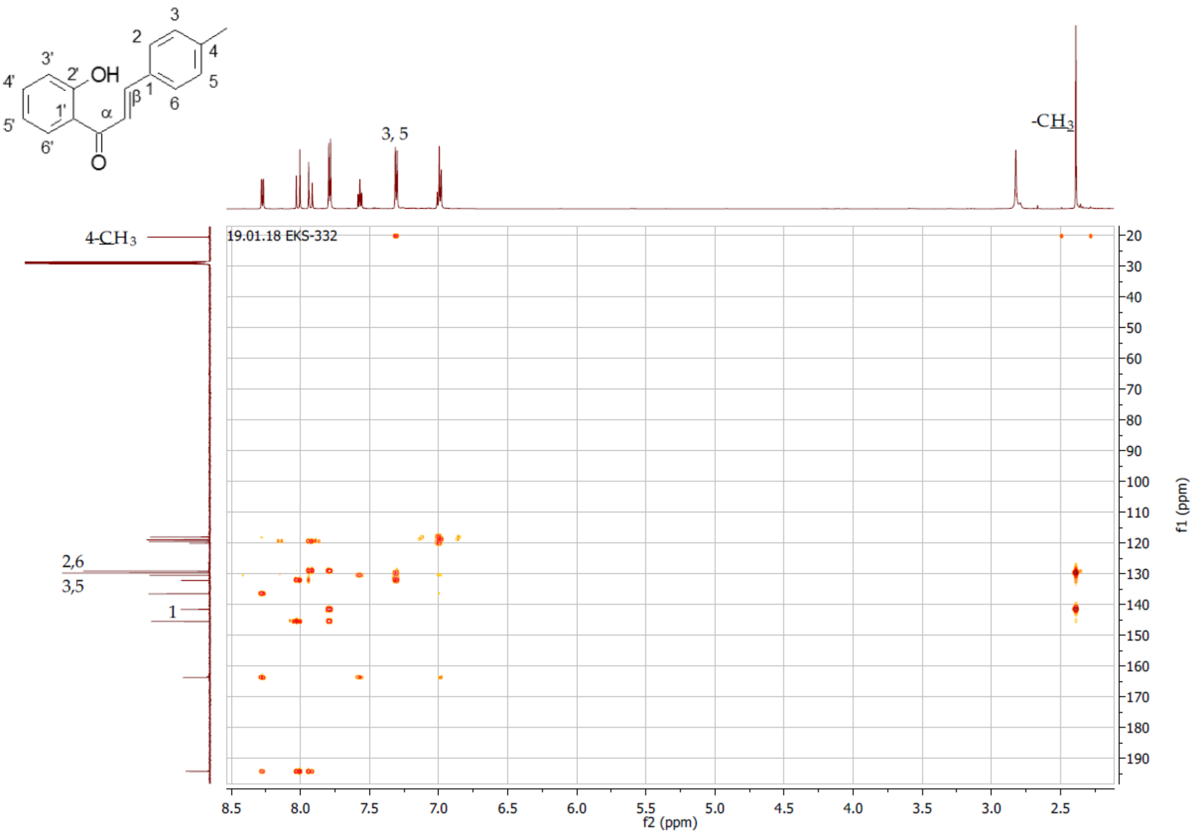

Figure S13. HMBC contour map – ^{1}H x ^{13}C expansion of 2′-hydroxy-4-methylchalcone (**3**)

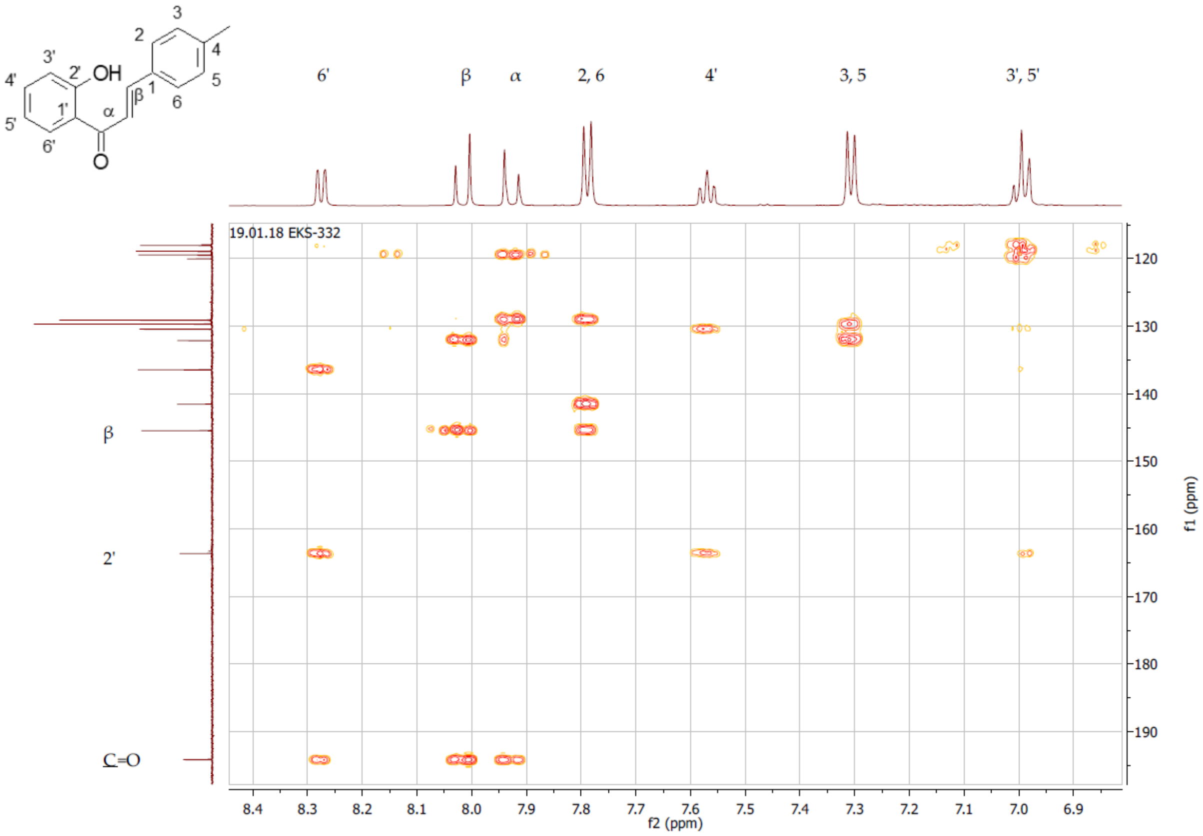

Figure S14. HMBC contour map – ^{1}H x ^{13}C expansion of 2′-hydroxy-4-methylchalcone (**3**)

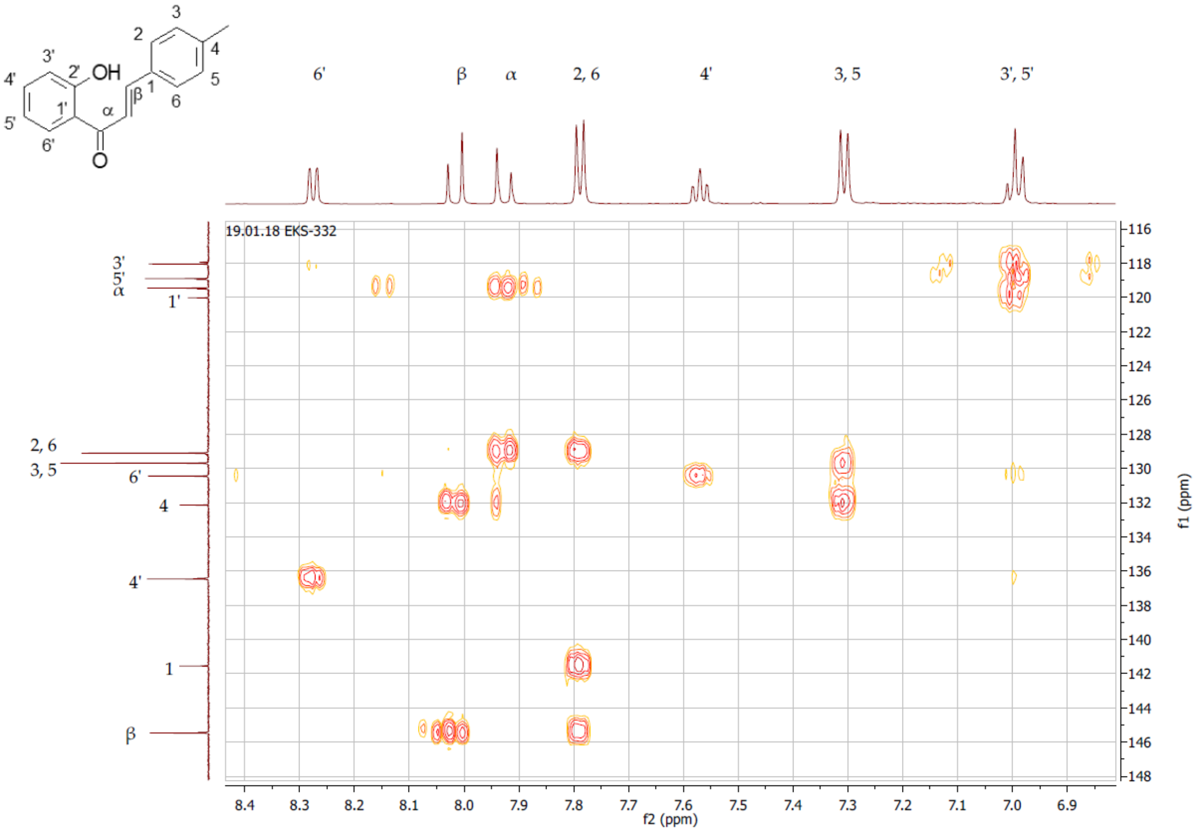

Figure S15. HMBC contour map – ^{1}H x ^{13}C expansion of 2′-hydroxy-4-methylchalcone (**3**)

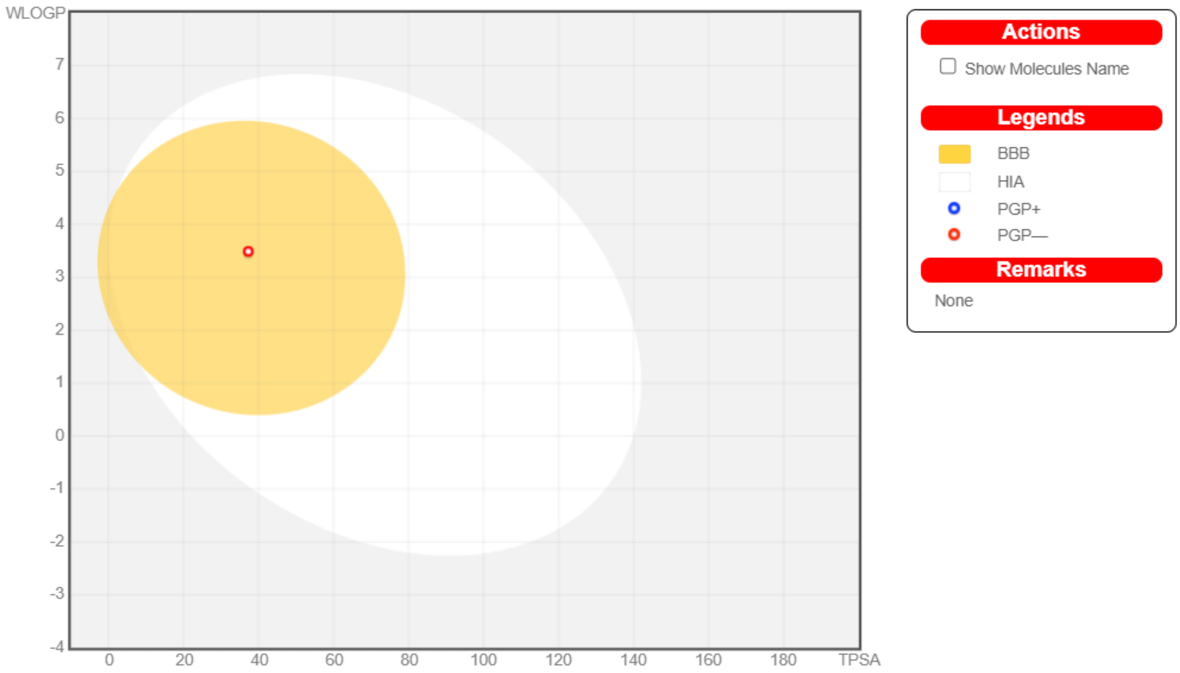

Molecule 1

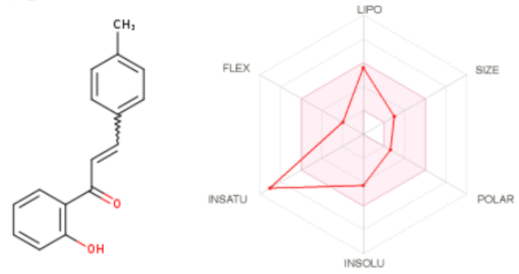

SMILES Cc1ccc(cc1)/C=C/C(=O)c1ccccc1O

Physicochemical Properties	
Formula	C16H14O2
Molecular weight	238.28 g/mol
Num. heavy atoms	18
Num. arom. heavy atoms	12
Fraction Csp3	0.06
Num. rotatable bonds	3
Num. H-bond acceptors	2
Num. H-bond donors	1
Molar Refractivity	73.24
TPSA	37.30 Å²

Lipophilicity	
Log $P_{o/w}$ (iLOGP)	2.78
Log $P_{o/w}$ (XLOGP3)	4.25
Log $P_{o/w}$ (WLOGP)	3.49
Log $P_{o/w}$ (MLOGP)	3.03
Log $P_{o/w}$ (SILICOS-IT)	3.95
Consensus Log $P_{o/w}$	3.50

Water Solubility	
Log *S* (ESOL)	-4.29
Solubility	1.22e-02 mg/ml ; 5.13e-05 mol/l
Class	Moderately soluble
Log *S* (Ali)	-4.74
Solubility	4.29e-03 mg/ml ; 1.80e-05 mol/l
Class	Moderately soluble
Log *S* (SILICOS-IT)	-4.77
Solubility	4.03e-03 mg/ml ; 1.69e-05 mol/l
Class	Moderately soluble

Pharmacokinetics	
GI absorption	High
BBB permeant	Yes
P-gp substrate	No
CYP1A2 inhibitor	Yes
CYP2C19 inhibitor	Yes
CYP2C9 inhibitor	Yes
CYP2D6 inhibitor	No
CYP3A4 inhibitor	No
Log K_p (skin permeation)	-4.74 cm/s

Druglikeness	
Lipinski	Yes; 0 violation
Ghose	Yes
Veber	Yes
Egan	Yes
Muegge	Yes
Bioavailability Score	0.55

Medicinal Chemistry	
PAINS	0 alert
Brenk	1 alert: michael_acceptor_1
Leadlikeness	No; 2 violations: MW<250, XLOGP3>3.5
Synthetic accessibility	2.52

Figure S16. 2′-Hydroxy-4-methylchalcone (**3**) physicochemical and ADME parameters prediction using the SwissADME modelling

Pa	Pi	Activity
0,944	0,004	Mucomembranous protector
0,908	0,004	Feruloyl esterase inhibitor
0,901	0,011	Membrane integrity agonist
0,872	0,002	1-Acylglycerol-3-phosphate O-acyltransferase inhibitor
0,845	0,003	Monophenol monooxygenase inhibitor
0,843	0,003	Antihypoxic
0,834	0,003	Pyruvate decarboxylase inhibitor
0,836	0,005	JAK2 expression inhibitor
0,849	0,020	Aspulvinone dimethylallyltransferase inhibitor
0,828	0,003	Carminative

Figure S17. 2′-Hydroxy-4-methylchalcone (**3**) physicochemical biological activity prediction using the Way2Drug Pass online modelling

Name	Confidence	ChEMBL ID
Prevotella intermedia	0.3405	CHEMBL613261
RESISTANT Staphylococcus simulans	0.3394	CHEMBL612425
Staphylococcus simulans	0.3358	CHEMBL612425
Yersinia pestis	0.3259	CHEMBL614597
Streptococcus mutans	0.2994	CHEMBL612426
Staphylococcus sciuri	0.2866	CHEMBL613150
Bacillus subtilis subsp. subtilis str. 168	0.2656	CHEMBL613315
Mycobacterium marinum	0.2552	CHEMBL614987
Porphyromonas gingivalis	0.2538	CHEMBL614414
RESISTANT Bacteroides thetaiotaomicron	0.2359	CHEMBL614412
Streptococcus sanguinis	0.1991	CHEMBL612314
Pseudomonas fluorescens	0.1910	CHEMBL612500

Figure S18. 2′-Hydroxy-4-methylchalcone (**3**) antibacterial activity prediction using the Way2Drug AntiBac-Pred modelling

Name	Confidence	ChEMBL ID
Epidermophyton floccosum	0.2695	CHEMBL612386
Trichophyton mentagrophytes	0.1523	CHEMBL613162
Trichosporon asahii	0.0529	CHEMBL613164

Figure S19. 2′-Hydroxy-4-methylchalcone (**3**) antifungal activity prediction using the Way2Drug AntiFun-Pred modelling

Virus	Protein target	Confidence
Dengue virus type 2	Genome polyprotein	0.6219
Human immunodeficiency virus 2	Human immunodeficiency virus type 2 integrase	0.5956
Severe acute respiratory syndrome coronavirus 2	Replicase polyprotein 1ab	0.5489
Infectious bronchitis virus	3C-like protease	0.2282
Human immunodeficiency virus 1	Human immunodeficiency virus type 1 integrase	0.1729
Varicella-zoster virus (strain Dumas) (HHV-3) (Human herpesvirus 3)	DNA polymerase	0.1221
Herpes simplex virus (type 1 / strain 17)	Human herpesvirus 1 DNA polymerase	0.1221
Vaccinia virus (strain Western Reserve) (VACV) (Vaccinia virus (strainWR))	DNA polymerase	0.0687

Figure S20. 2′-Hydroxy-4-methylchalcone (**3**) antiviral activity prediction using the Way2Drug AntiVir-Pred modelling

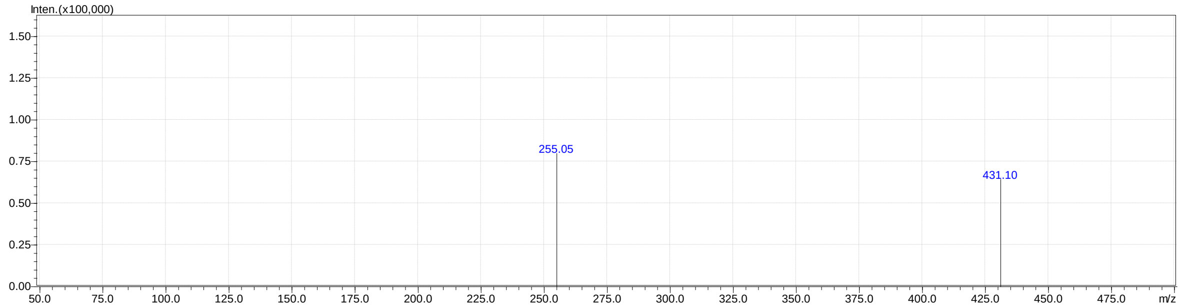

Figure S21. MS analysis 2′-hydroxy-4-methyldihydrochalcone 5′-*O*-*β*-D-(4″-*O*-methyl)-glucopyranoside (**3a**)

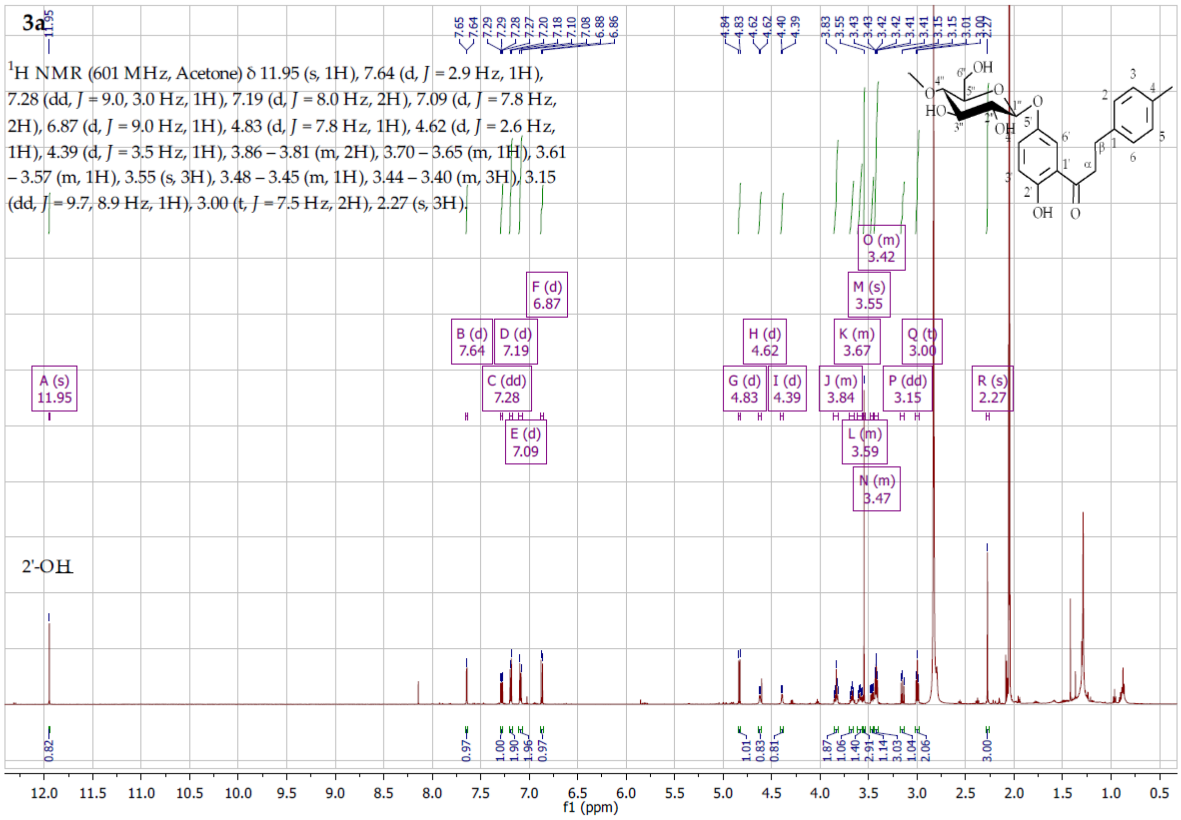

Figure S22. ¹H NMR spectrum (*δ*, acetone-d6, 600 MHz) of 2′-hydroxy-4-methyldihydrochalcone 5′-*O*-*β*-D-(4″-*O*-methyl)-glucopyranoside (**3a**)

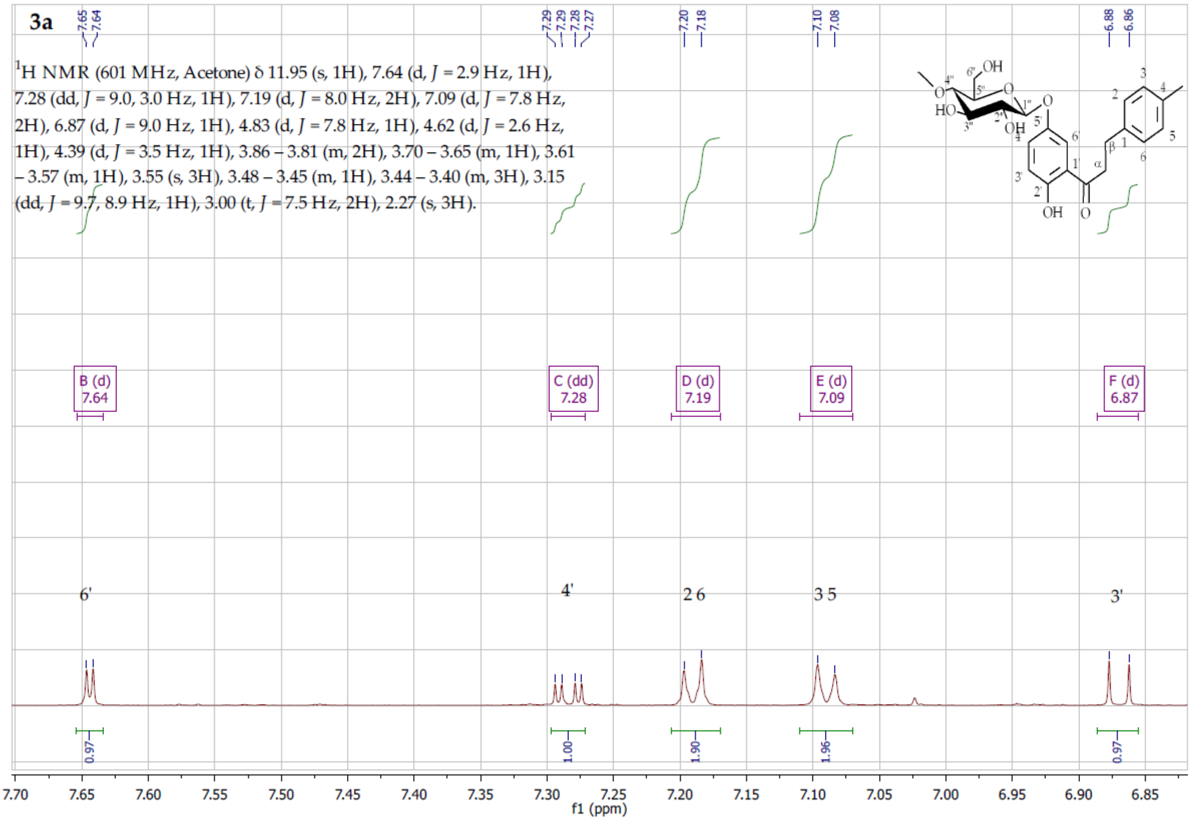

Figure S23. ¹H NMR spectrum expansion (*δ,* acetone-d6, 600 MHz) of 2′-hydroxy-4-methyldihydrochalcone 5′-*O*-*β*-D-(4″-*O*-methyl)-glucopyranoside (**3a**)

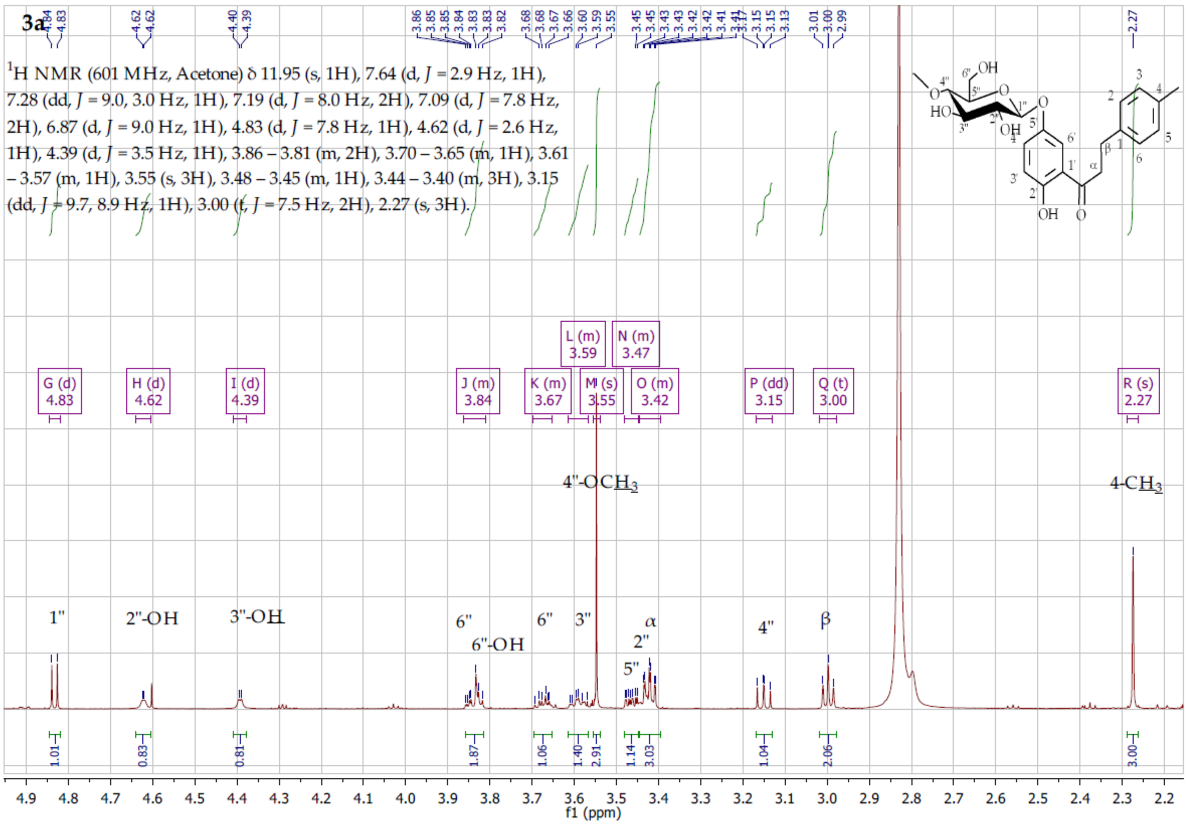

Figure S24. ^{1}H NMR spectrum expansion (*δ*, acetone-d6, 600 MHz) of 2′-hydroxy-4-methyldihydrochalcone 5′-*O*-*β*-D-(4″-*O*-methyl)-glucopyranoside (**3a**)

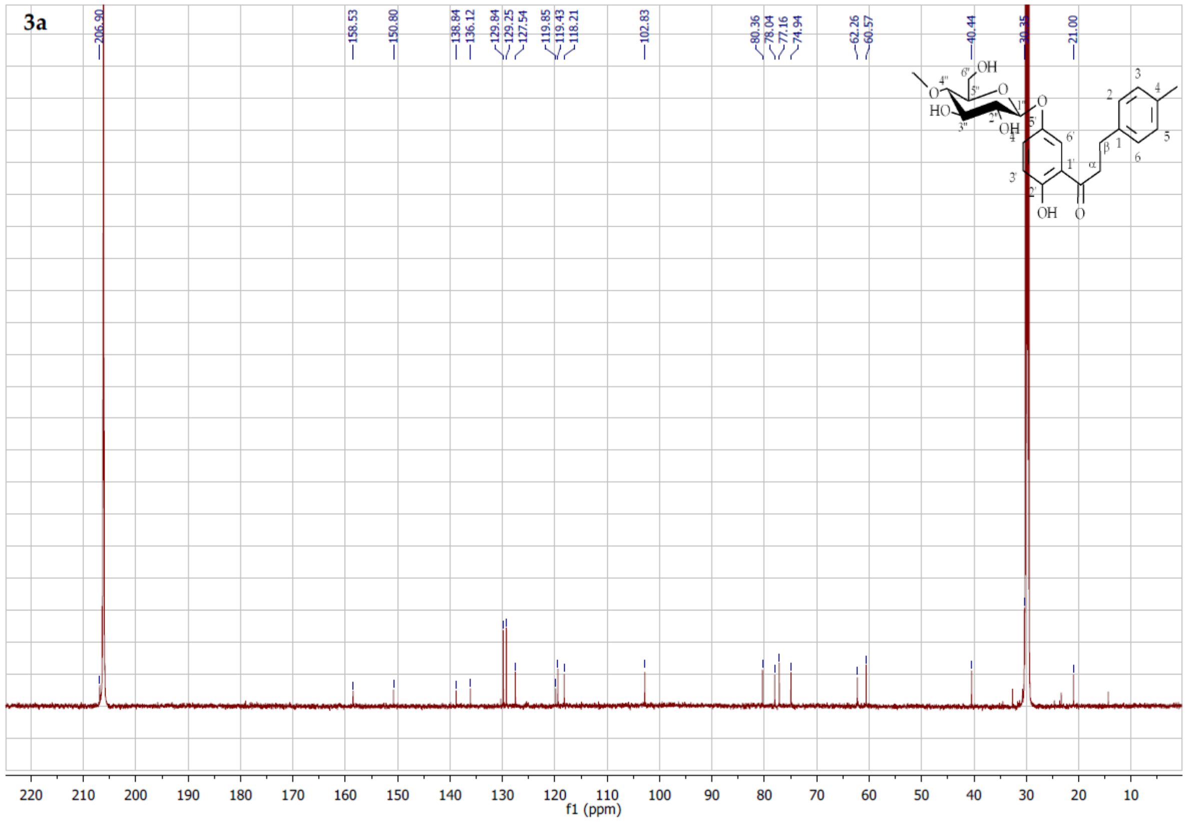

Figure S25. ^{13}C NMR spectrum expansion (*δ*, acetone-d6, 151 MHz) of 2′-hydroxy-4-methyldihydrochalcone 5′-*O*-*β*-D-(4″-*O*-methyl)-glucopyranoside (**3a**)

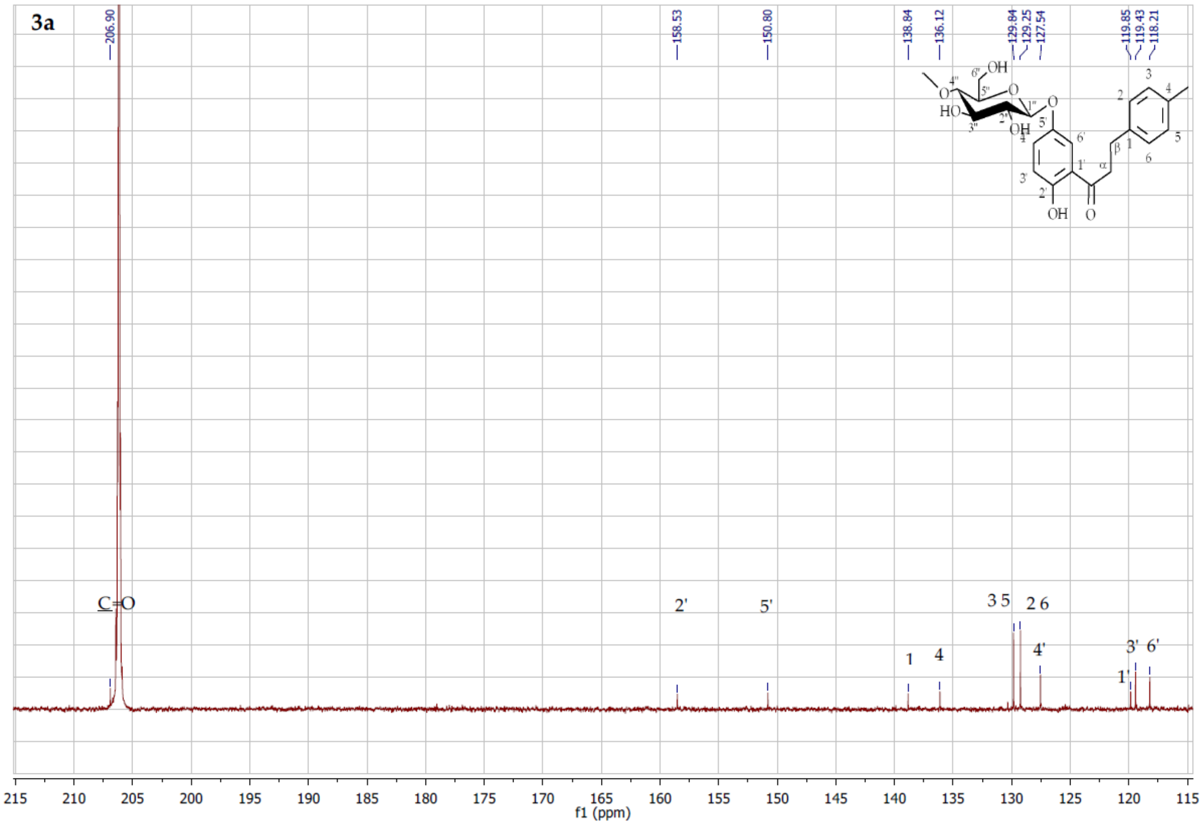

Figure S26. ^{13}C NMR spectrum expansion (*δ*, acetone-d6, 151 MHz) of 2′-hydroxy-4-methyldihydrochalcone 5′-*O*-*β*-D-(4″-*O*-methyl)-glucopyranoside (**3a**)

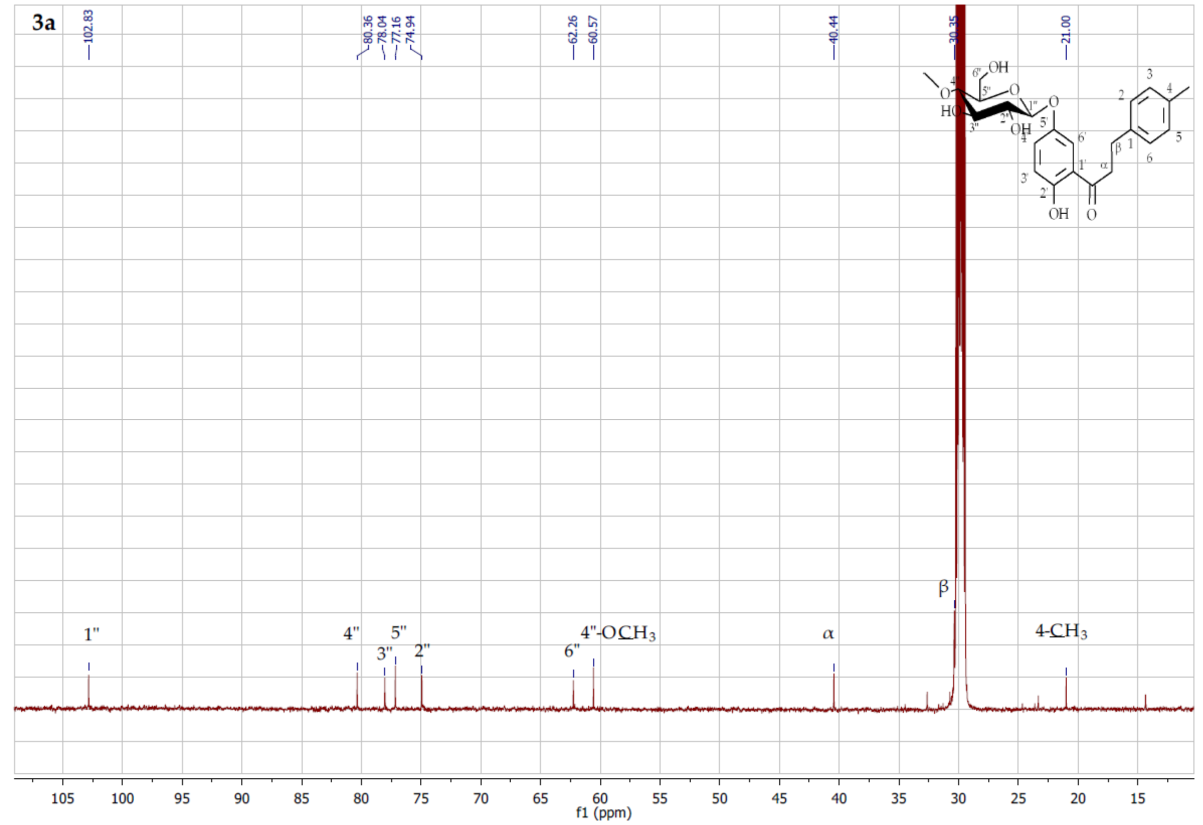

Figure S27. ^{13}C NMR spectrum expansion (*δ*, acetone-d6, 151 MHz) of 2′-hydroxy-4-methyldihydrochalcone 5′-*O*-*β*-D-(4″-*O*-methyl)-glucopyranoside (**3a**)

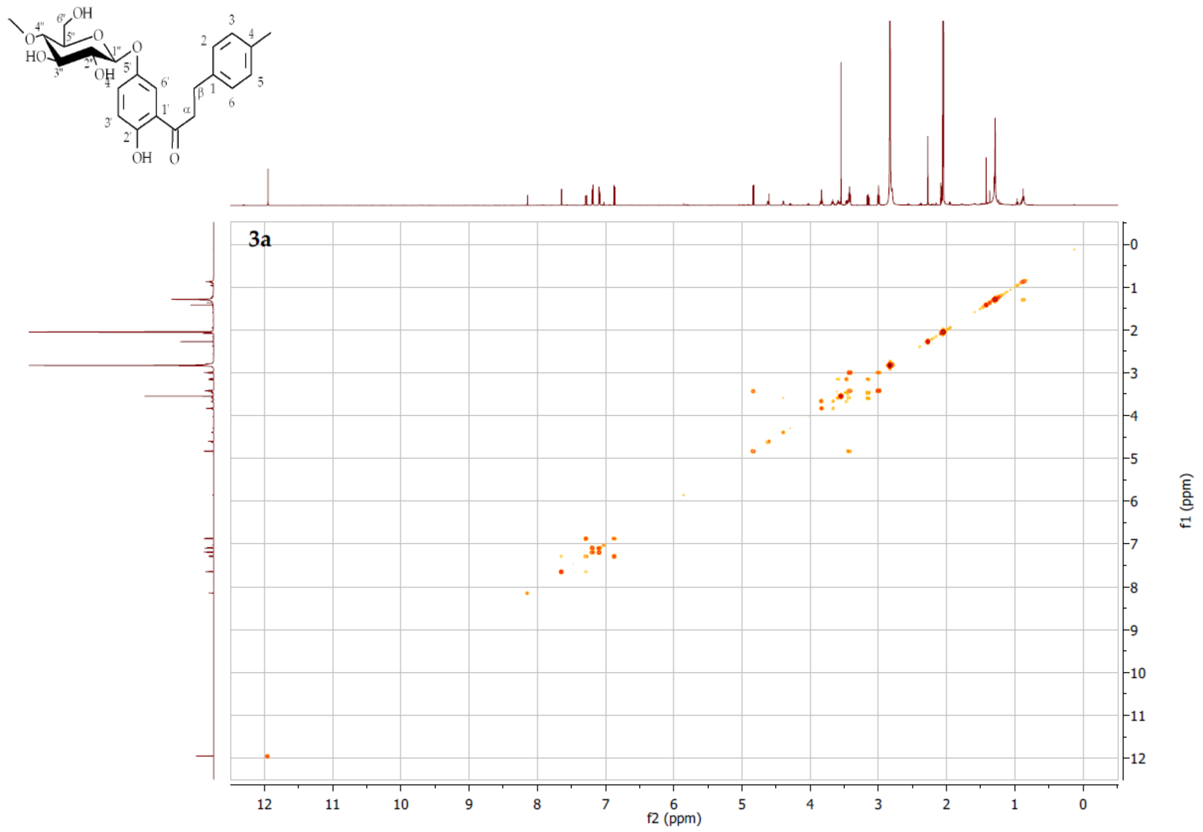

Figure S28. COSY contour map – ^{1}H x ^{1}H of 2′-hydroxy-4-methyldihydrochalcone 5′-*O*-*β*-D-(4″-*O*-methyl)-glucopyranoside (**3a**)

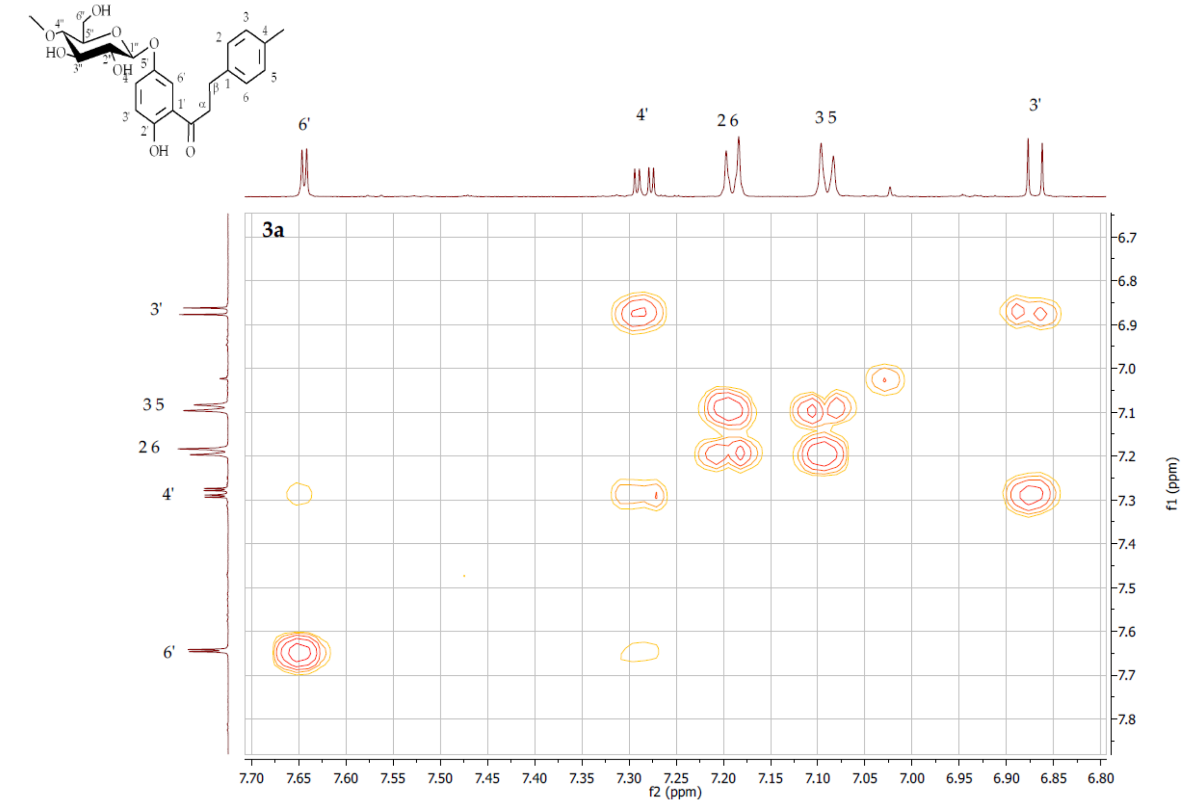

Figure S29. COSY contour map – ^{1}H x ^{1}H expansion of 2′-hydroxy-4-methyldihydrochalcone 5′-*O*-*β*-D-(4″-*O*-methyl)-glucopyranoside (**3a**)

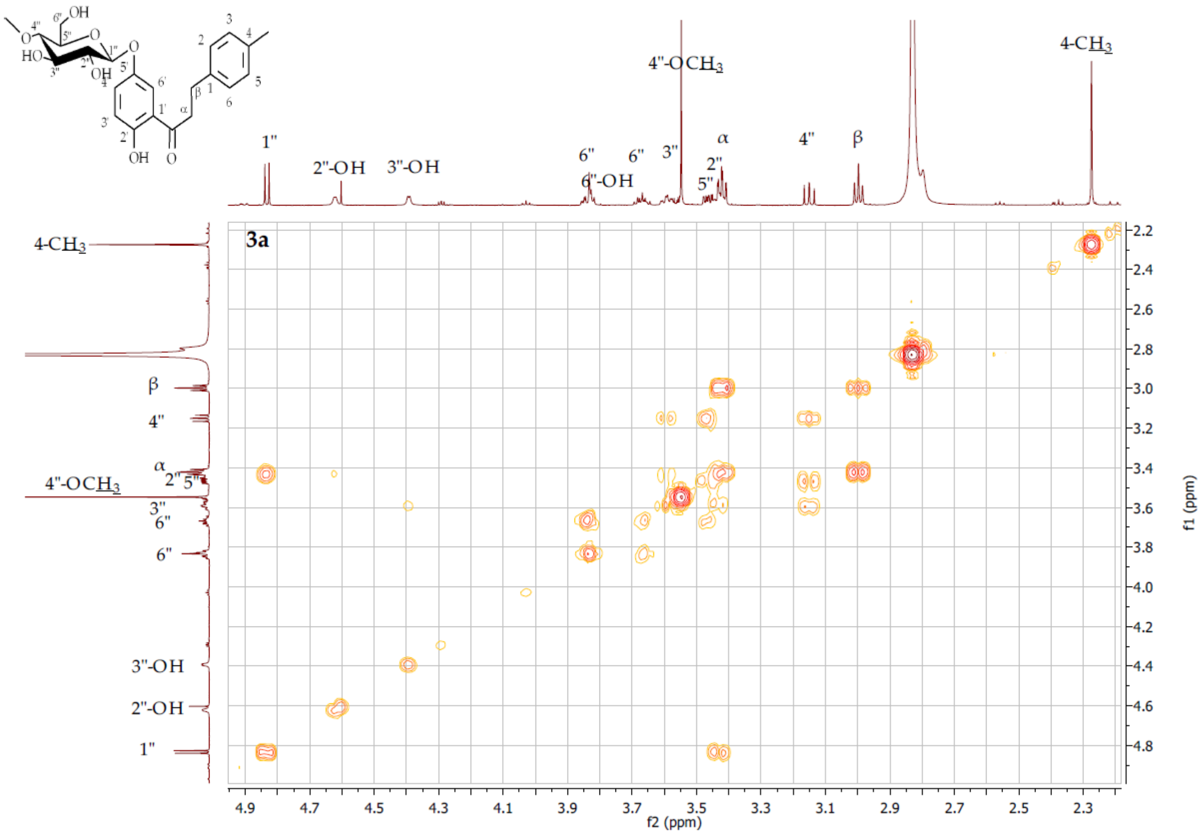

Figure S30. COSY contour map – ^{1}H x ^{1}H expansion of 2′-hydroxy-4-methyldihydrochalcone 5′-*O*-*β*-D-(4″-*O*-methyl)-glucopyranoside (**3a**)

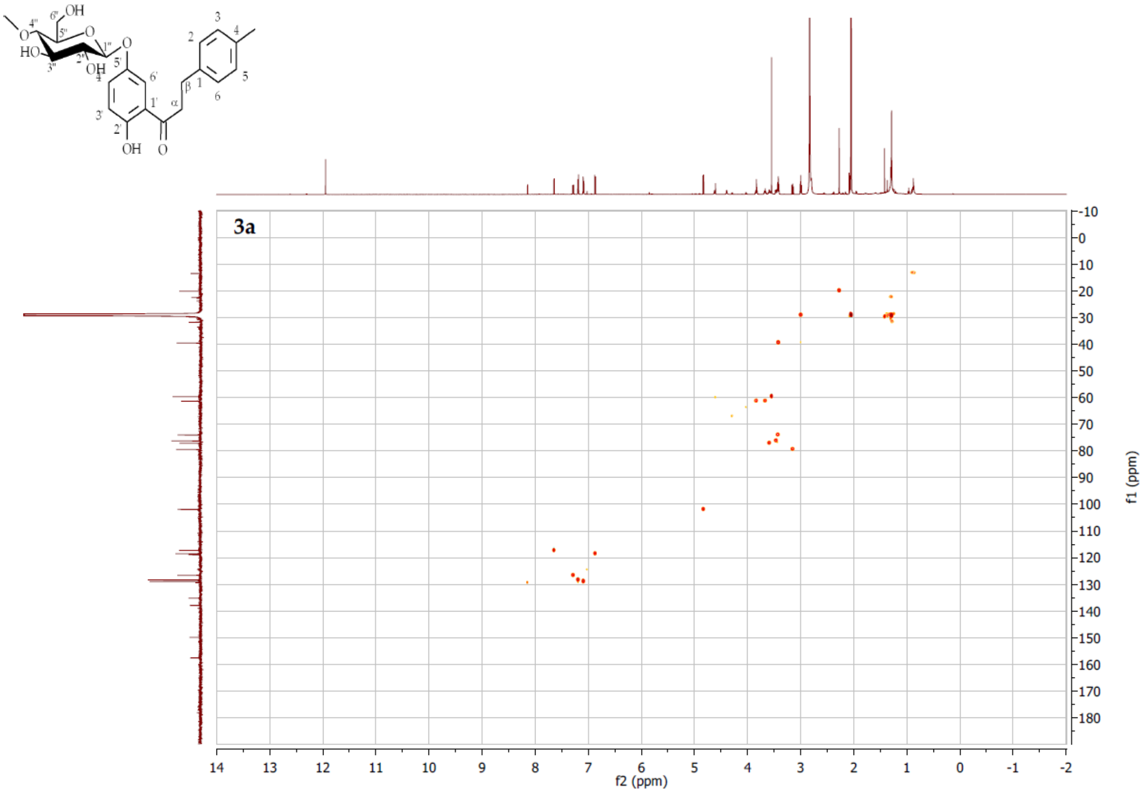

Figure S31. HSQC contour map – ^{1}H x ^{13}C of 2′-hydroxy-4-methyldihydrochalcone 5′-*O*-*β*-D-(4″-*O*-methyl)-glucopyranoside (**3a**)

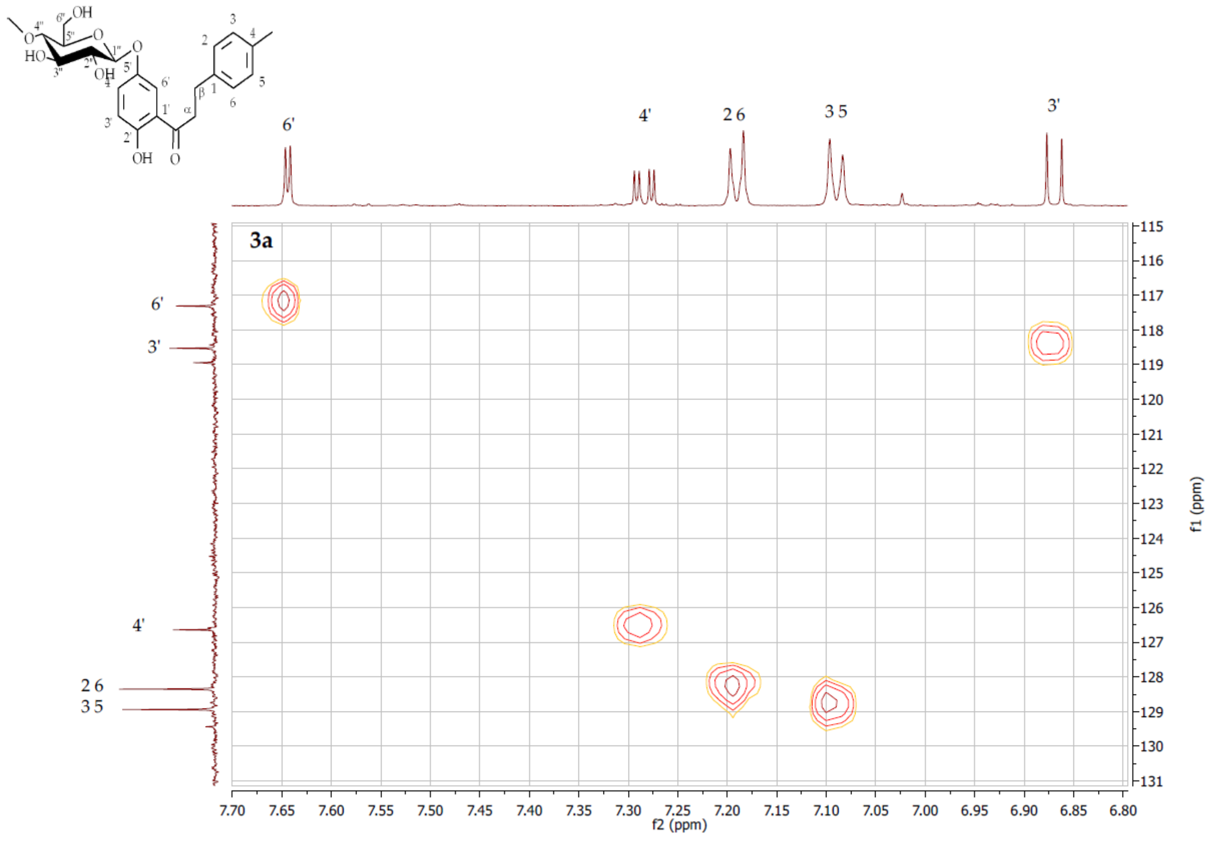

Figure S32. HSQC contour map – ^{1}H x ^{13}C expansion of 2′-hydroxy-4-methyldihydrochalcone 5′-*O*-*β*-D-(4″-*O*-methyl)-glucopyranoside (**3a**)

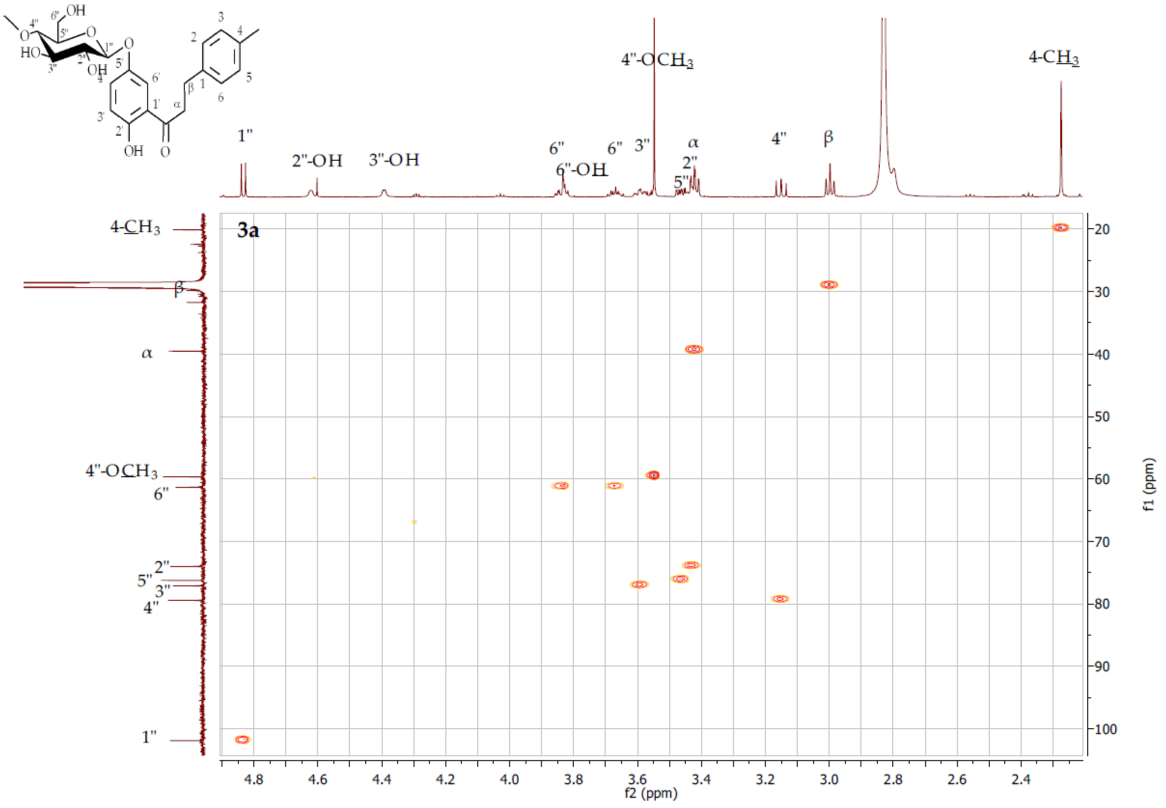

Figure S33. HSQC contour map – ^{1}H x ^{13}C expansion of 2′-hydroxy-4-methyldihydrochalcone 5′-*O*-*β*-D-(4″-*O*-methyl)-glucopyranoside (**3a**)

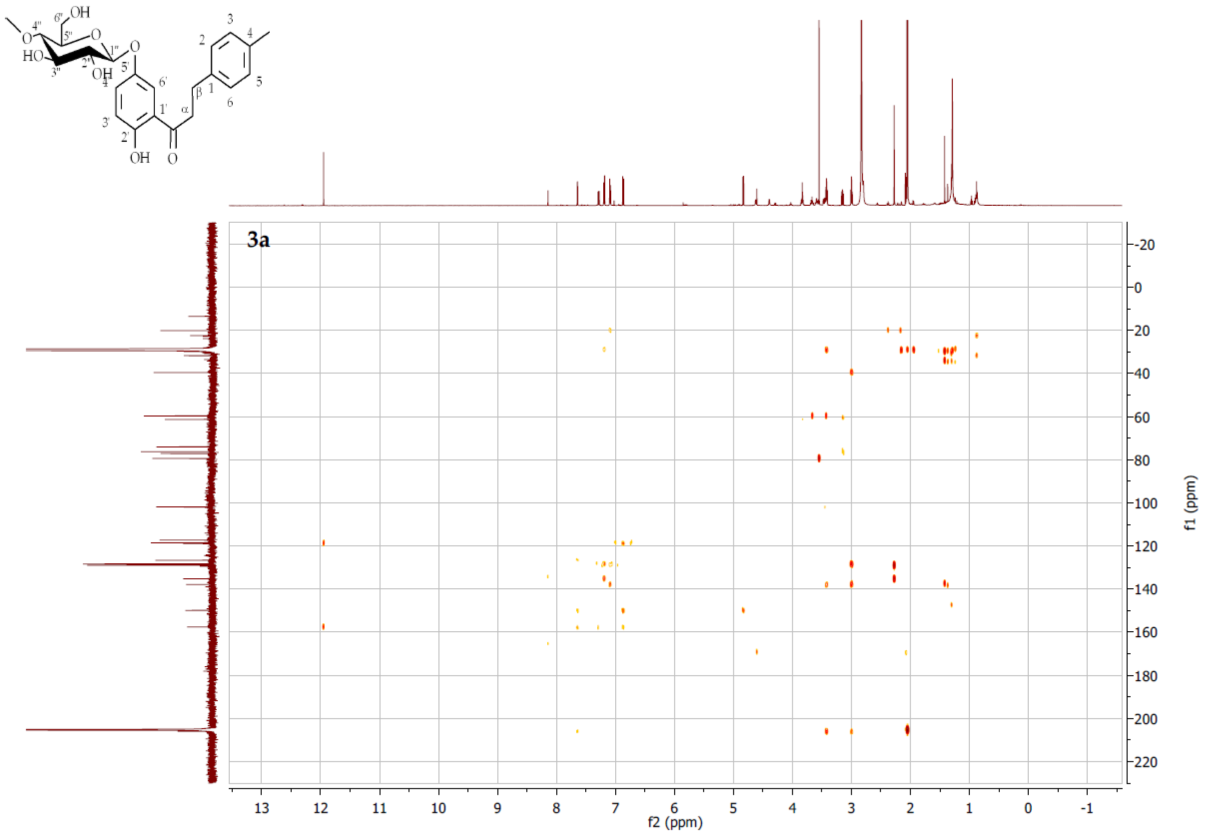

Figure S34. HMBC contour map – ^{1}H x ^{13}C of 2′-hydroxy-4-methyldihydrochalcone 5′-*O*-*β*-D-(4″-*O*-methyl)-glucopyranoside (**3a**)

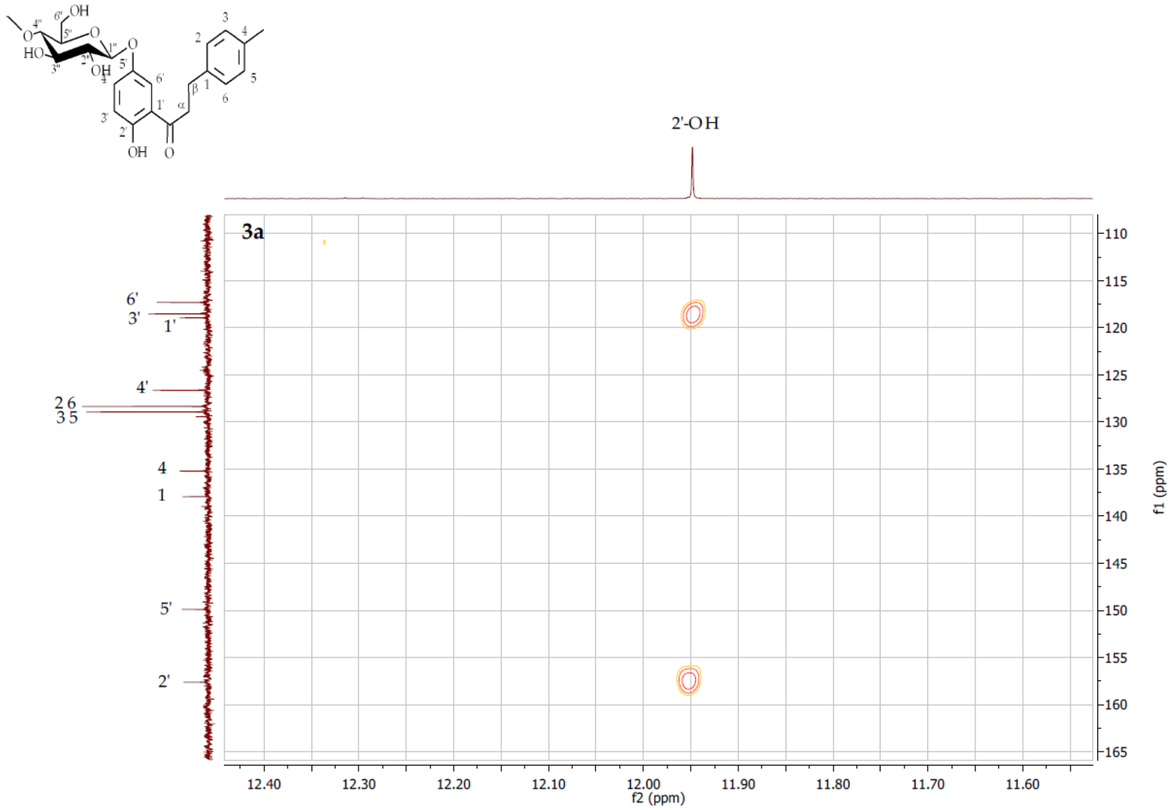

Figure S35. HMBC contour map – ^{1}H x ^{13}C expansion of 2′-hydroxy-4-methyldihydrochalcone 5′-*O*-*β*-D-(4″-*O*-methyl)-glucopyranoside (**3a**)

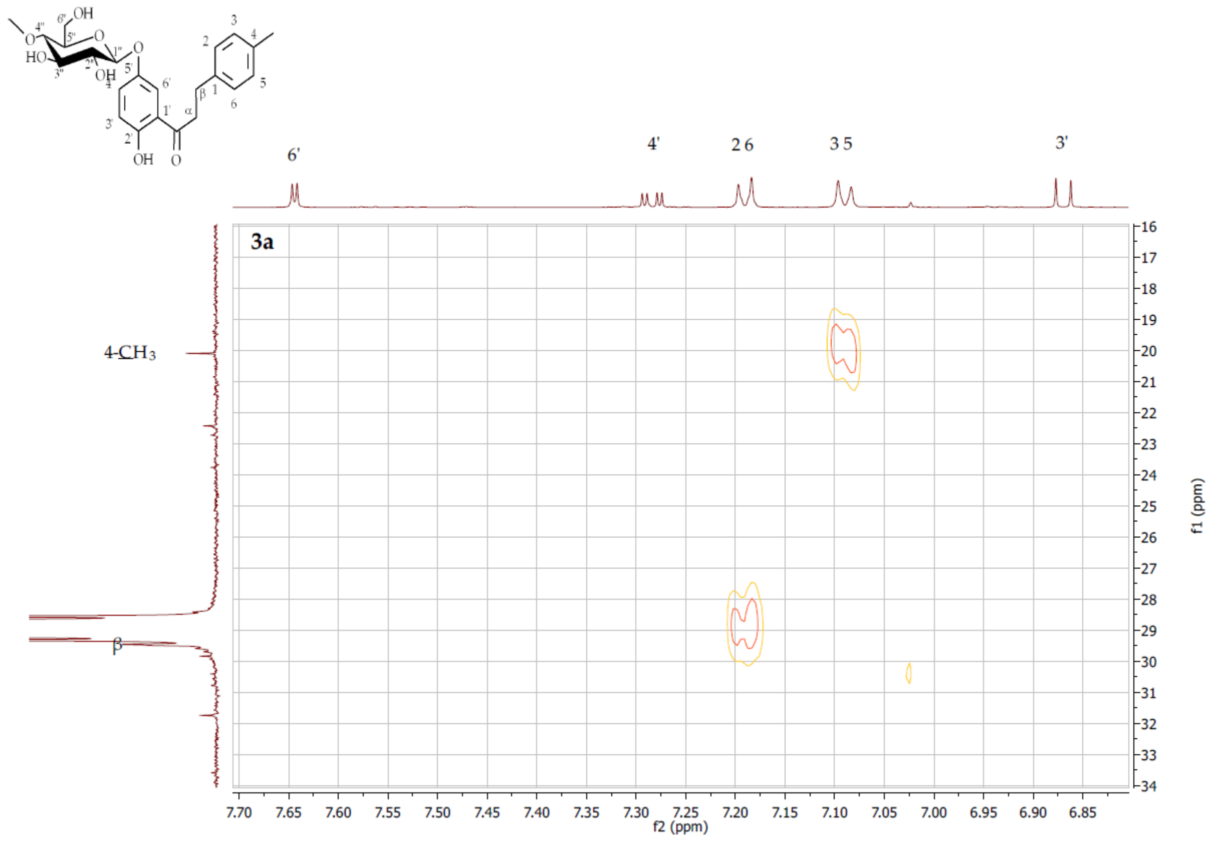

Figure S36. HMBC contour map – ^1H x ^{13}C expansion of 2′-hydroxy-4-methyldihydrochalcone 5′-*O*-*β*-D-(4″-*O*-methyl)-glucopyranoside (**3a**)

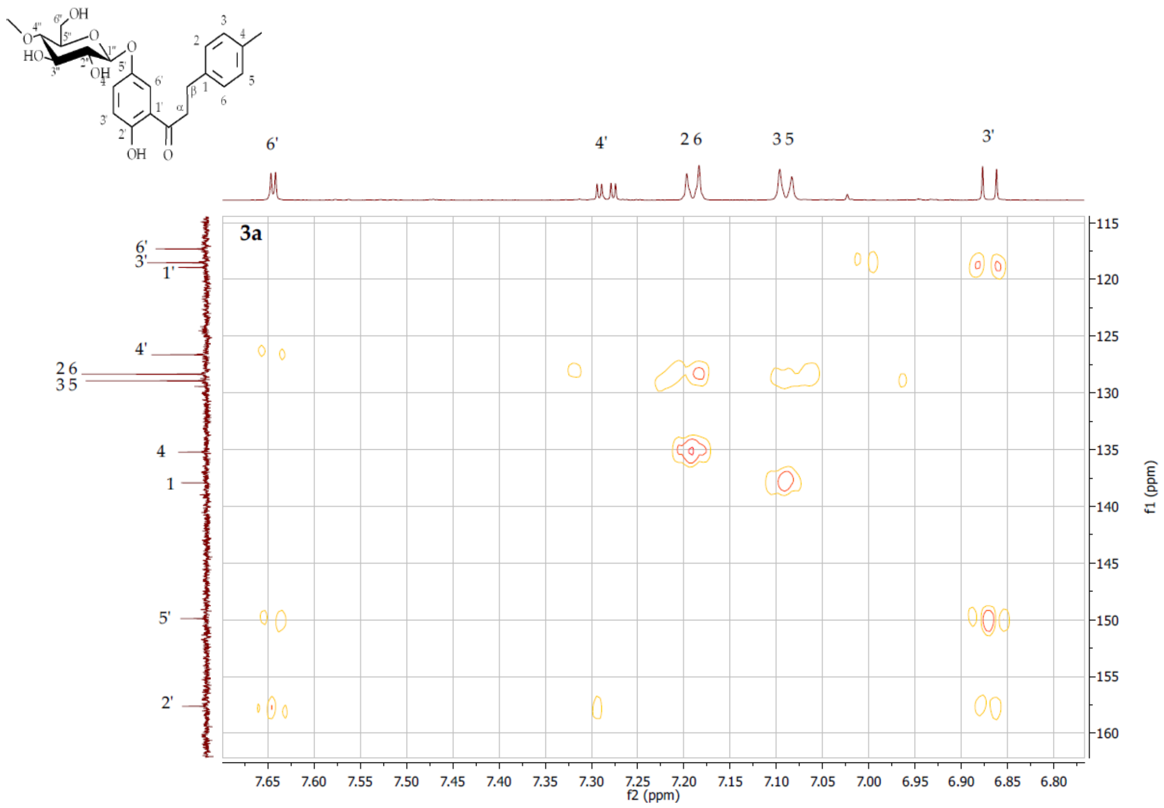

Figure S37. HMBC contour map – ^1H x ^{13}C expansion of 2′-hydroxy-4-methyldihydrochalcone 5′-*O*-*β*-D-(4″-*O*-methyl)-glucopyranoside (**3a**)

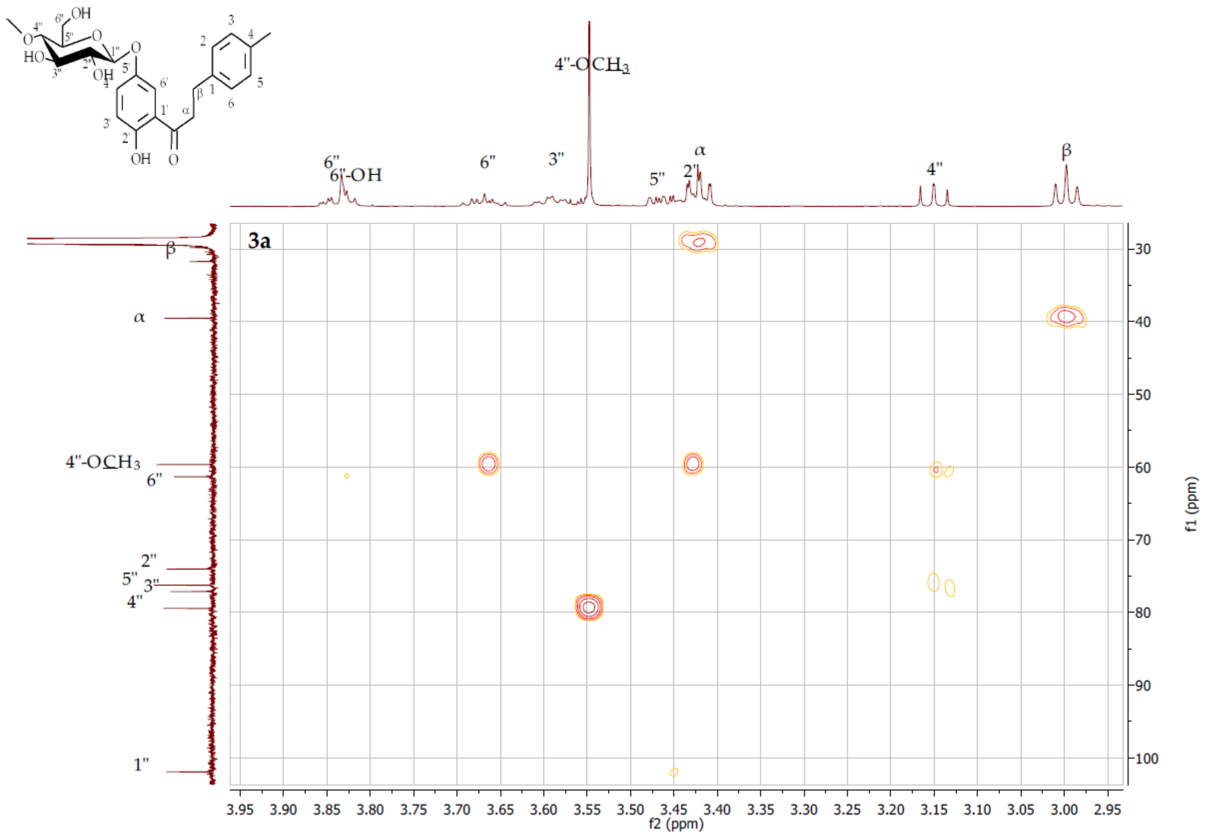

Figure S38. HMBC contour map – ^{1}H x ^{13}C expansion of 2′-hydroxy-4-methyldihydrochalcone 5′-*O*-*β*-D-(4″-*O*-methyl)-glucopyranoside (**3a**)

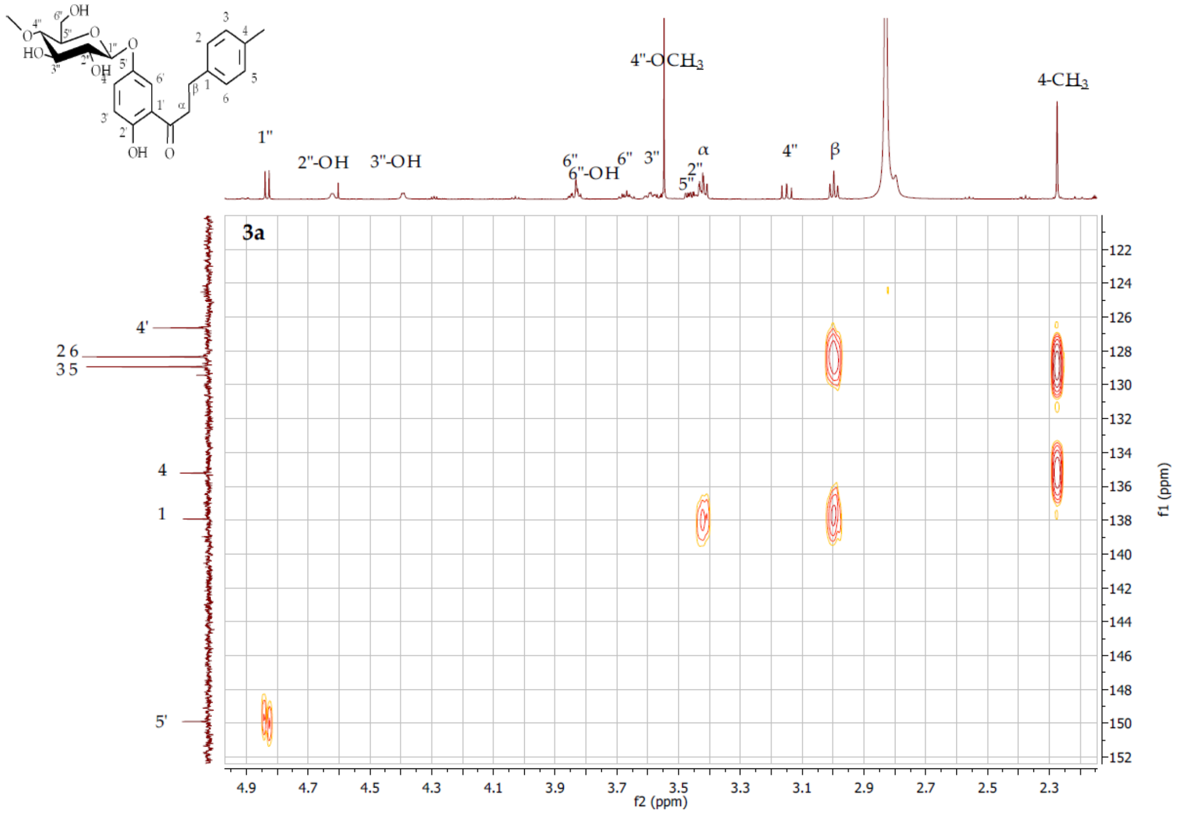

Figure S39. HMBC contour map – ^{1}H x ^{13}C expansion of 2′-hydroxy-4-methyldihydrochalcone 5′-*O*-*β*-D-(4″-*O*-methyl)-glucopyranoside (**3a**)

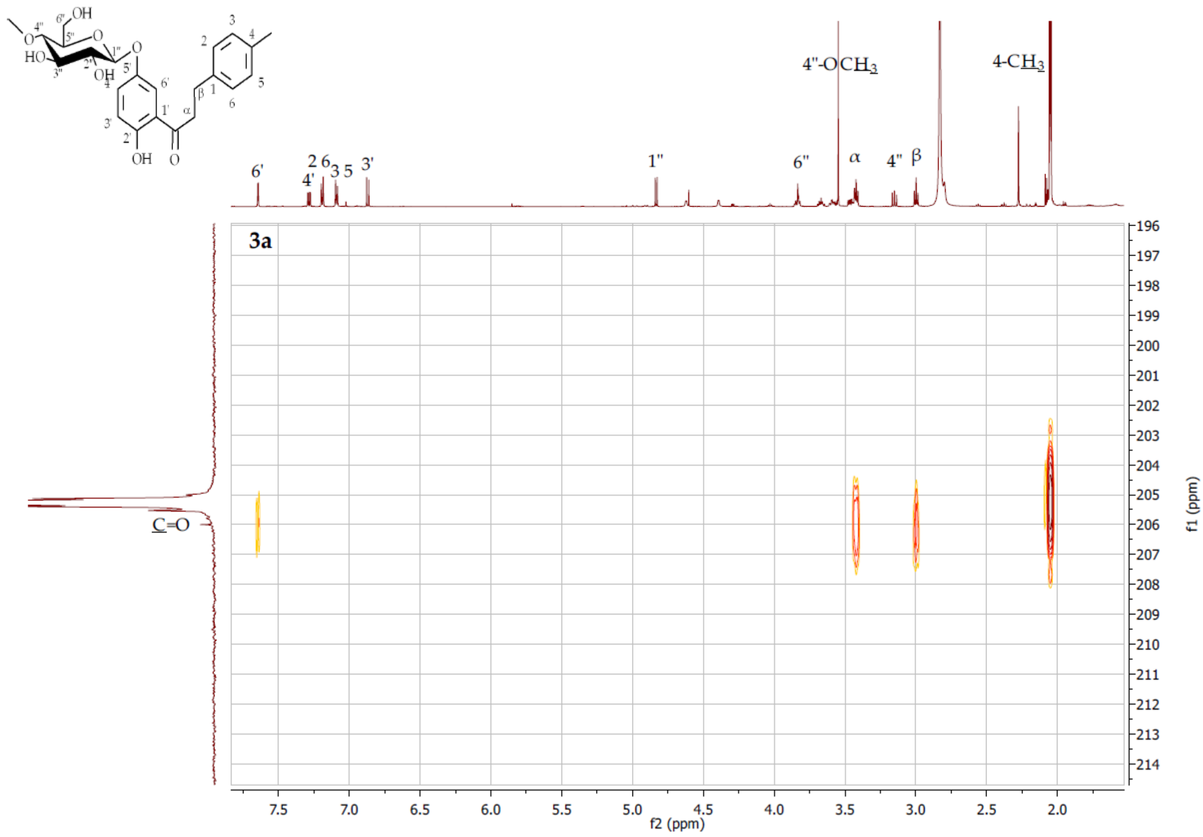

Figure S40. HMBC contour map – ^{1}H x ^{13}C expansion of 2′-hydroxy-4-methyldihydrochalcone 5′-*O*-*β*-D-(4″-*O*-methyl)-glucopyranoside (**3a**)

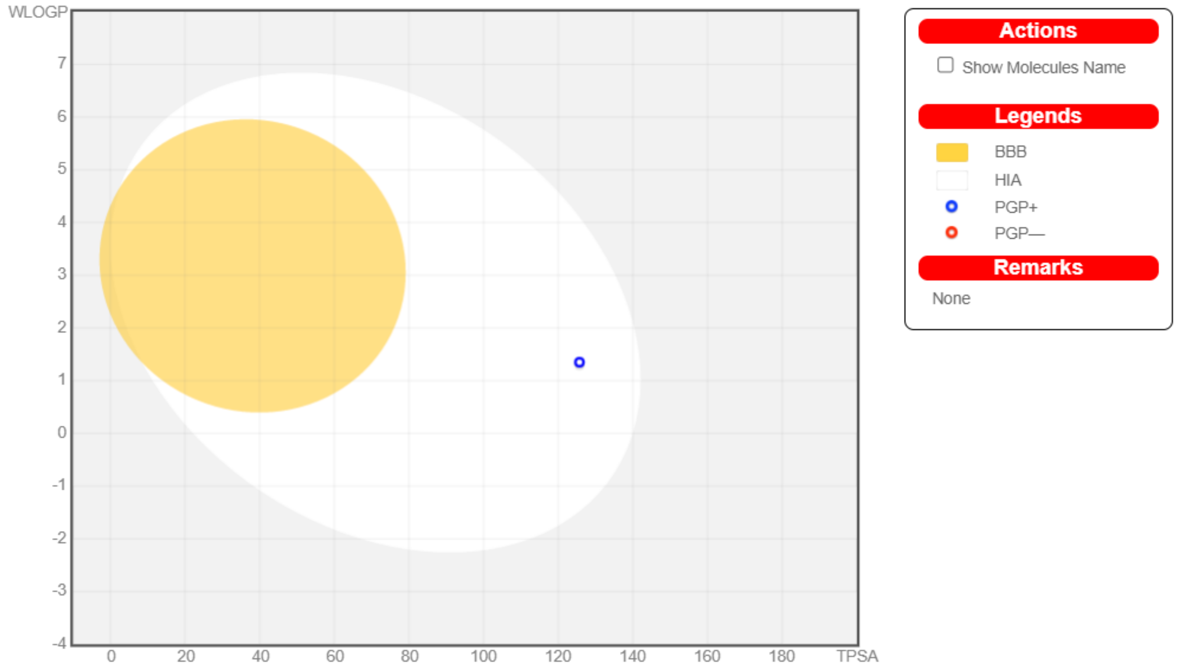

Molecule 1

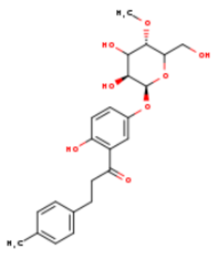

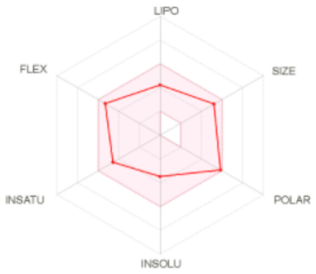

SMILES OCC1O[C@@H](Oc2ccc(c(c2)C(=O)CCc2ccc(cc2)C)O)[C@H](C([C@@H]1OC)O)O

Physicochemical Properties	
Formula	C23H28O8
Molecular weight	432.46 g/mol
Num. heavy atoms	31
Num. arom. heavy atoms	12
Fraction Csp3	0.43
Num. rotatable bonds	8
Num. H-bond acceptors	8
Num. H-bond donors	4
Molar Refractivity	111.79
TPSA	125.68 Å²

Lipophilicity	
Log $P_{o/w}$ (iLOGP)	3.39
Log $P_{o/w}$ (XLOGP3)	1.88
Log $P_{o/w}$ (WLOGP)	1.35
Log $P_{o/w}$ (MLOGP)	0.02
Log $P_{o/w}$ (SILICOS-IT)	2.15
Consensus Log $P_{o/w}$	1.76

Water Solubility	
Log *S* (ESOL)	-3.46
Solubility	1.49e-01 mg/ml ; 3.43e-04 mol/l
Class	Soluble
Log *S* (Ali)	-4.14
Solubility	3.12e-02 mg/ml ; 7.22e-05 mol/l
Class	Moderately soluble
Log *S* (SILICOS-IT)	-3.90
Solubility	5.42e-02 mg/ml ; 1.25e-04 mol/l
Class	Soluble

Pharmacokinetics	
GI absorption	High
BBB permeant	No
P-gp substrate	Yes
CYP1A2 inhibitor	No
CYP2C19 inhibitor	No
CYP2C9 inhibitor	No
CYP2D6 inhibitor	No
CYP3A4 inhibitor	No
Log K_p (skin permeation)	-7.60 cm/s

Druglikeness	
Lipinski	Yes; 0 violation
Ghose	Yes
Veber	Yes
Egan	Yes
Muegge	Yes
Bioavailability Score	0.55

Medicinal Chemistry	
PAINS	0 alert
Brenk	0 alert
Leadlikeness	No; 2 violations: MW>350, Rotors>7
Synthetic accessibility	4.93

Figure S41. 2′-Hydroxy-4-methyldihydrochalcone 5′-*O*-*β*-D-(4″-*O*-methyl)-glucopyranoside (**3a**) physicochemical and ADME parameters prediction using the SwissADME modelling

Pa	Pi	Activity
0,935	0,005	CDP-glycerol glycerophosphotransferase inhibitor
0,931	0,002	Monophenol monooxygenase inhibitor
0,925	0,002	3-Phytase inhibitor
0,901	0,004	Antiinfective
0,896	0,002	Lactase inhibitor
0,883	0,015	Membrane integrity agonist
0,851	0,004	Anticarcinogenic
0,836	0,003	Hepatoprotectant
0,838	0,008	Anaphylatoxin receptor antagonist
0,830	0,002	Neurotrophic factor enhancer

Figure S42. 2′-Hydroxy-4-methyldihydrochalcone 5′-*O*-*β*-D-(4″-*O*-methyl)-glucopyranoside (**3a**) biological activity prediction using the Way2Drug Pass online modelling

Name	Confidence	ChEMBL ID
Clostridium ramosum	0.6506	CHEMBL614971
Actinomyces meyeri	0.6011	CHEMBL612289
RESISTANT Acinetobacter pittii	0.5872	CHEMBL3140321
Clostridium cadaveris	0.5690	CHEMBL614970
RESISTANT Mycobacterium ulcerans	0.5659	CHEMBL612965
Mycobacterium mageritense	0.5658	CHEMBL612959
Yersinia pestis	0.5140	CHEMBL614597
RESISTANT Staphylococcus aureus subsp. aureus RN4220	0.4679	CHEMBL2366906
Staphylococcus lugdunensis	0.4609	CHEMBL613303
Lactobacillus plantarum	0.4426	CHEMBL614973
Nocardia transvalensis	0.4244	CHEMBL613234
Clostridium sordellii	0.4145	CHEMBL613072
Streptococcus sanguinis	0.4078	CHEMBL612314
Streptococcus oralis	0.3993	CHEMBL613305
RESISTANT Chlamydia trachomatis	0.3883	CHEMBL614606

Figure S43. 2′-Hydroxy-4-methyldihydrochalcone 5′-*O*-*β*-D-(4″-*O*-methyl)-glucopyranoside (**3a**) antibacterial activity prediction using the Way2Drug AntiBac-Pred modelling

Name	Confidence	ChEMBL ID
Rhizopus oryzae	0.4467	CHEMBL612306
Trichophyton mentagrophytes	0.3920	CHEMBL613162
Absidia corymbifera	0.3831	CHEMBL612369
Saccharomyces cerevisiae	0.1941	CHEMBL361
Aspergillus niger	0.1738	CHEMBL358
Epidermophyton floccosum	0.1567	CHEMBL612386
Mucor	0.1250	CHEMBL612521
Penicillium marneffei	0.0903	CHEMBL612994
Trichosporon asahii	0.0586	CHEMBL613164
Mucor hiemalis	0.0584	CHEMBL612949
Galactomyces geotrichum	0.0355	CHEMBL613775
Candida rugosa	0.0283	CHEMBL612869

Figure S44. 2′-Hydroxy-4-methyldihydrochalcone 5′-*O*-*β*-D-(4″-*O*-methyl)-glucopyranoside (**3a**) antifungal activity prediction using the Way2Drug AntiFun-Pred modelling

Virus	Protein target	Confidence
Severe acute respiratory syndrome coronavirus 2	Replicase polyprotein 1ab	0.8116
Human immunodeficiency virus 2	Human immunodeficiency virus type 2 integrase	0.0461
Varicella-zoster virus (strain Dumas) (HHV-3) (Human herpesvirus 3)	Thymidine kinase	0.0090

Figure S45. 2′-Hydroxy-4-methyldihydrochalcone 5′-*O*-*β*-D-(4″-*O*-methyl)-glucopyranoside (**3a**) antiviral activity prediction using the Way2Drug AntiVir-Pred modelling

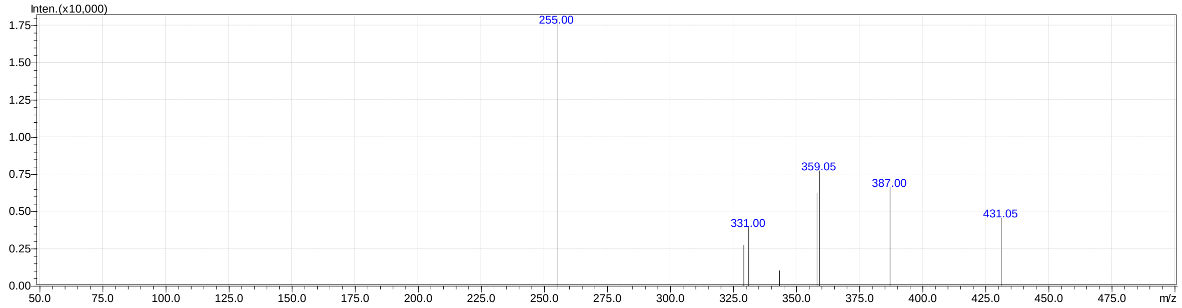

Figure S46. MS analysis 4-hydroxymethyldihydrochalcone 2′-*O*-*β*-D-(4″-*O*-methyl)-glucopyranoside (**3b**)

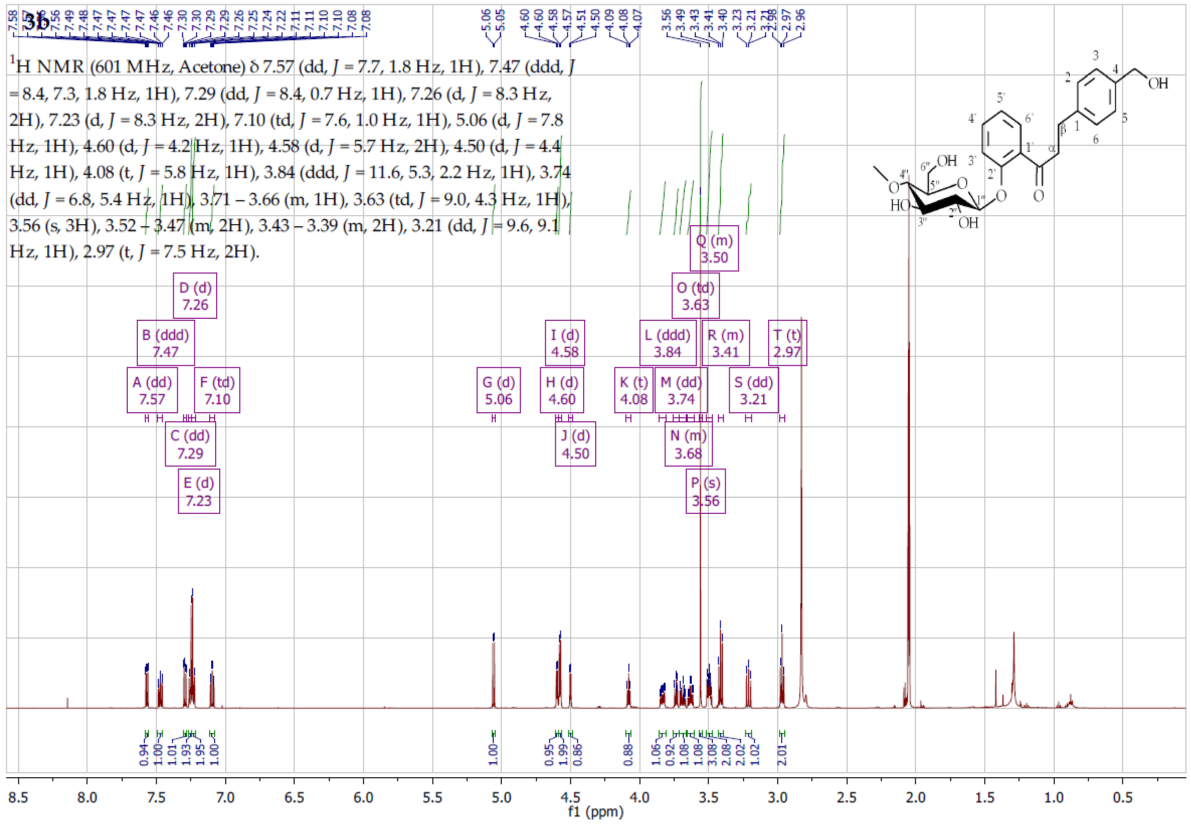

Figure S47. ¹H NMR spectrum (*δ*, acetone-d6, 600 MHz) of 4-hydroxymethyldihydrochalcone 2′-*O*-*β*-D-(4″-*O*-methyl)-glucopyranoside (**3b**)

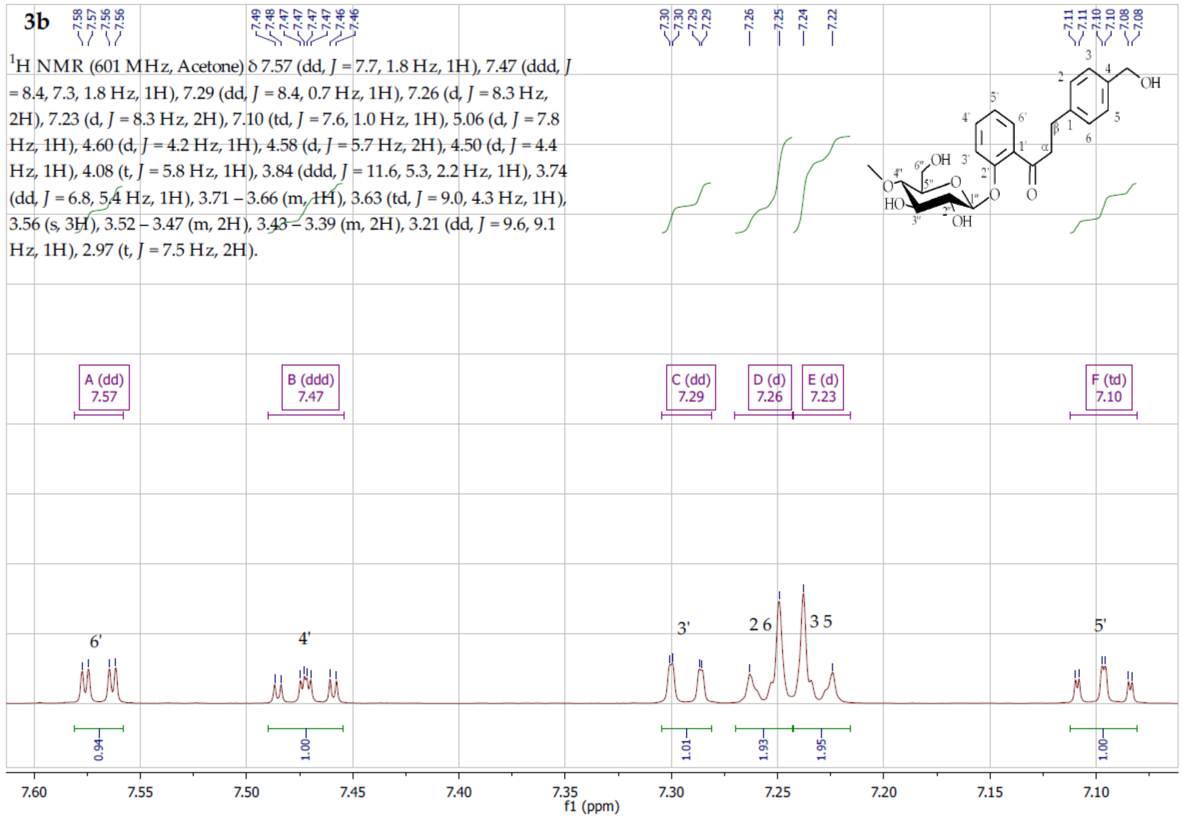

Figure S48. ^{1}H NMR spectrum expansion (*δ*, acetone-d6, 600 MHz) of 4-hydroxymethyldihydrochalcone 2′-*O*-*β*-D-(4″-*O*-methyl)-glucopyranoside (**3b**)

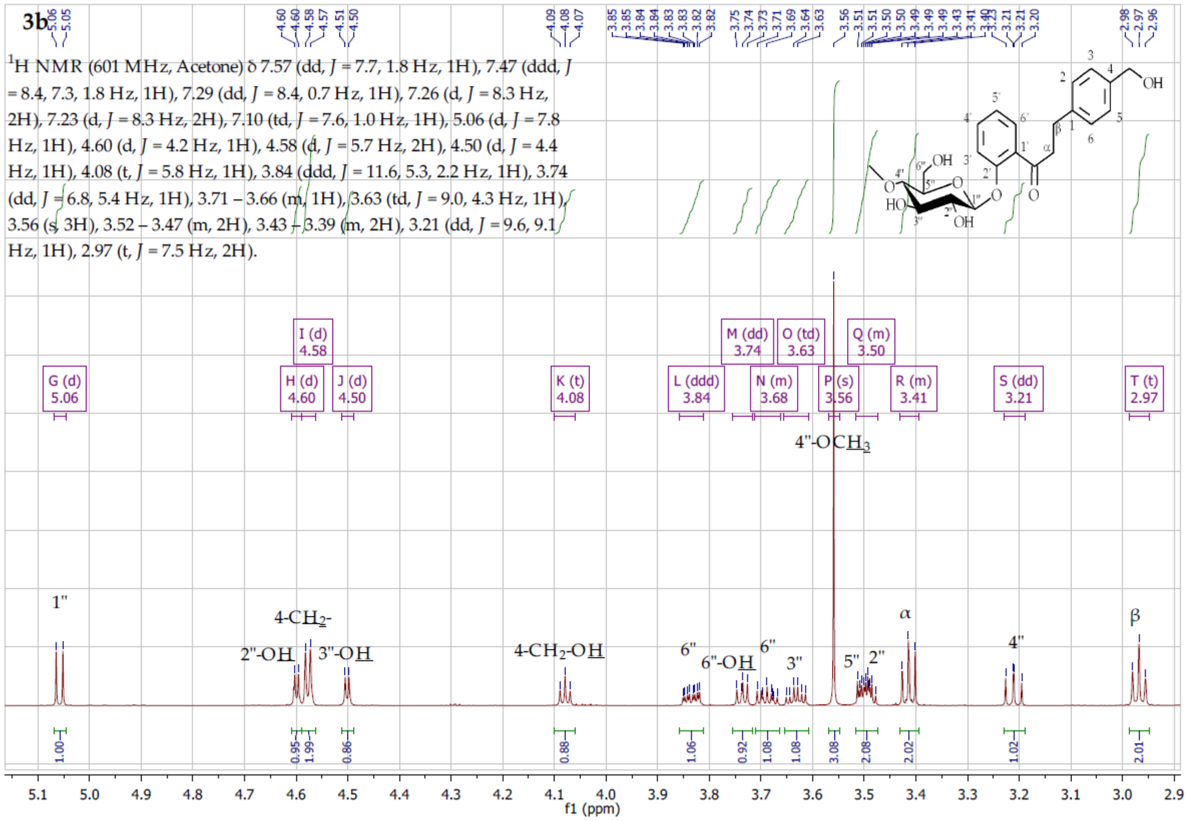

Figure S49. ^{1}H NMR spectrum expansion (*δ*, acetone-d6, 600 MHz) of 4-hydroxymethyldihydrochalcone 2′-*O*-*β*-D-(4″-*O*-methyl)-glucopyranoside (**3b**)

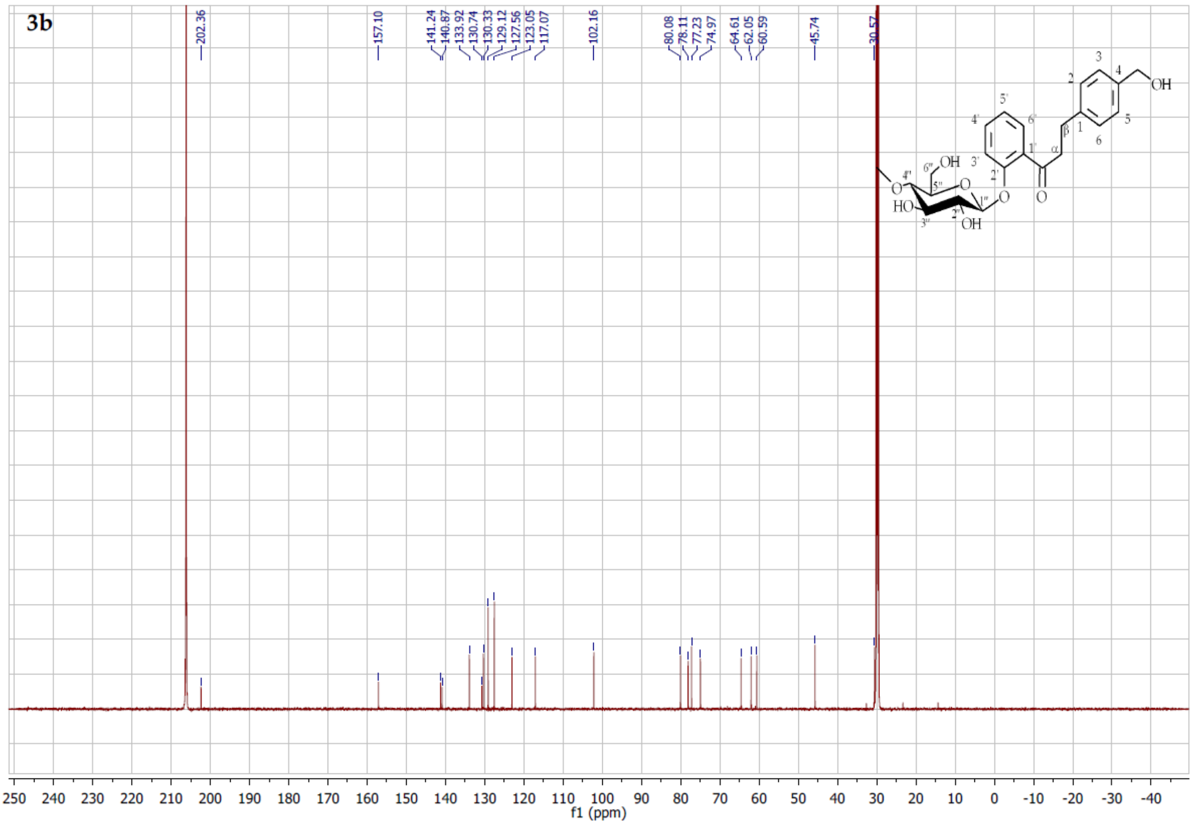

Figure S50. ^{13}C NMR spectrum expansion (*δ*, acetone-d6, 151 MHz) of 4-hydroxymethyldihydrochalcone 2′-*O*-*β*-D-(4″-*O*-methyl)-glucopyranoside (**3b**)

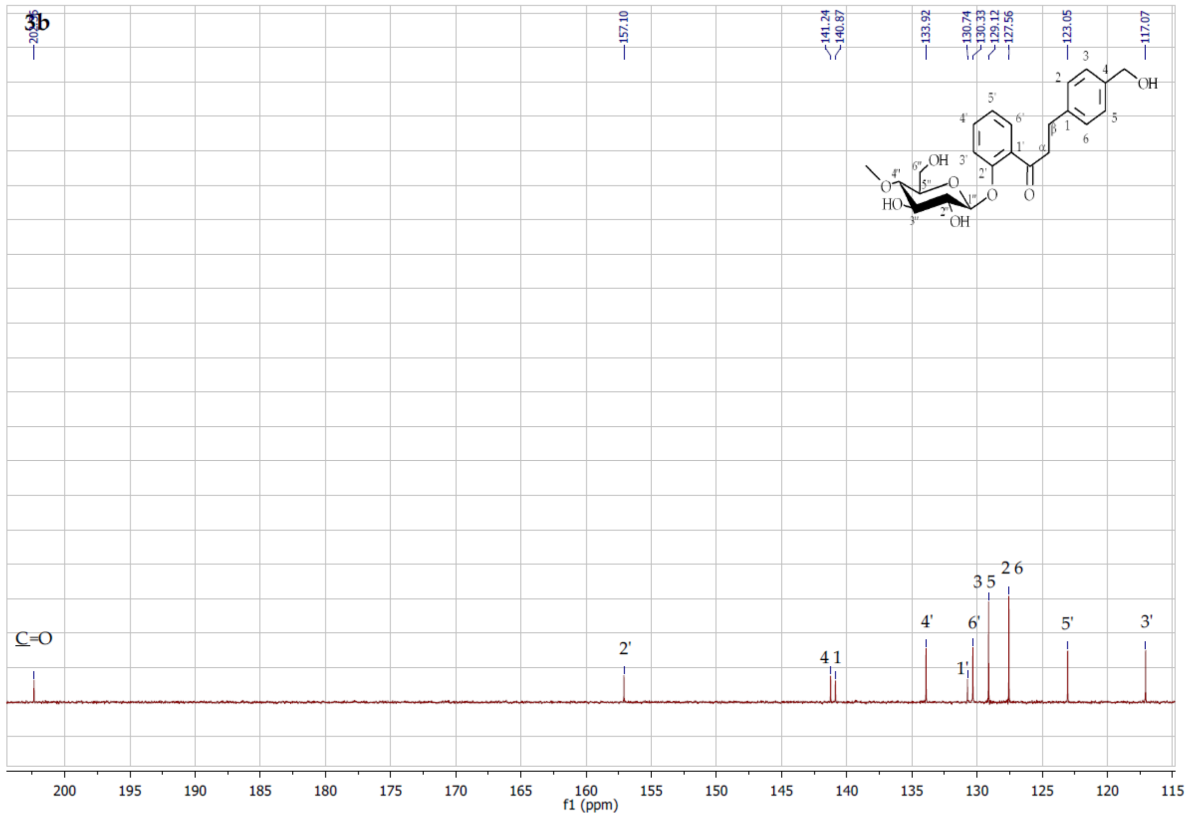

Figure S51. ^{13}C NMR spectrum expansion (*δ*, acetone-d6, 151 MHz) of 4-hydroxymethyldihydrochalcone 2′-*O*-*β*-D-(4″-*O*-methyl)-glucopyranoside (**3b**)

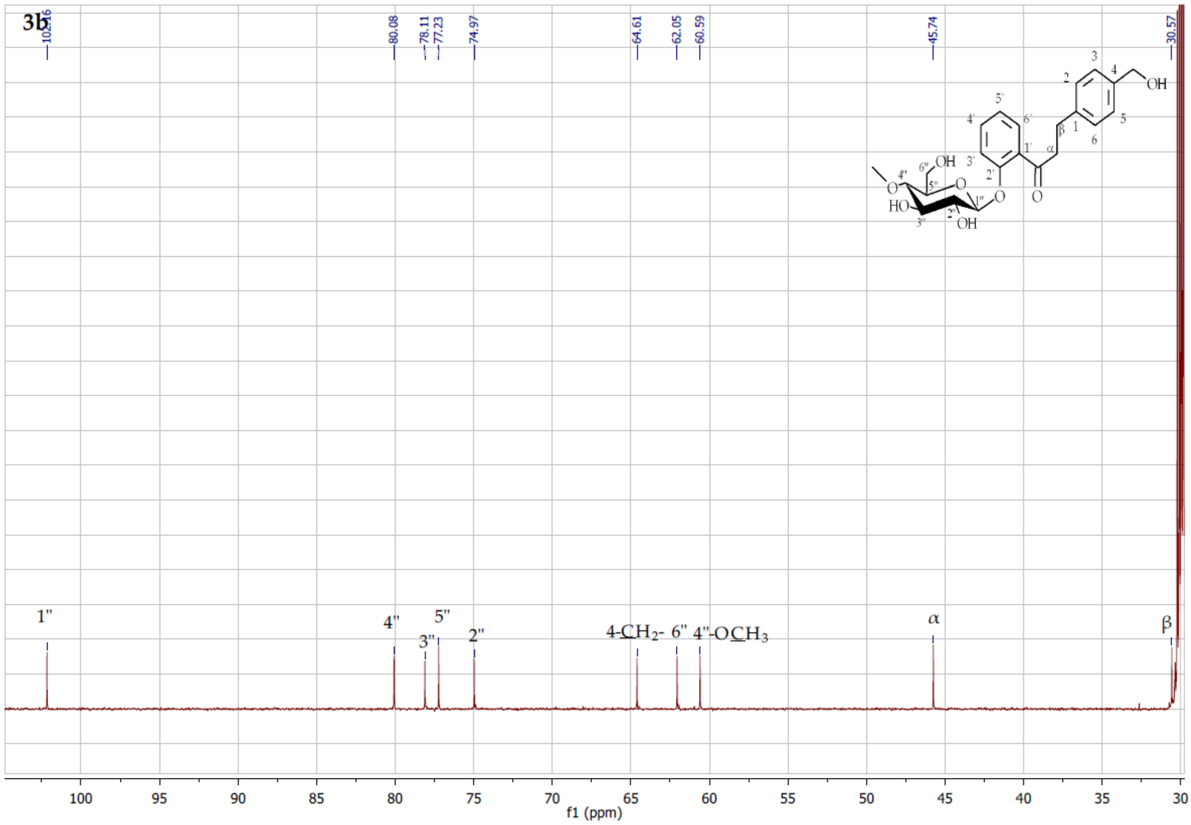

Figure S52. ^{13}C NMR spectrum expansion (δ, acetone-d6, 151 MHz) of 4-hydroxymethyldihydrochalcone 2′-*O*-*β*-D-(4″-*O*-methyl)-glucopyranoside (**3b**)

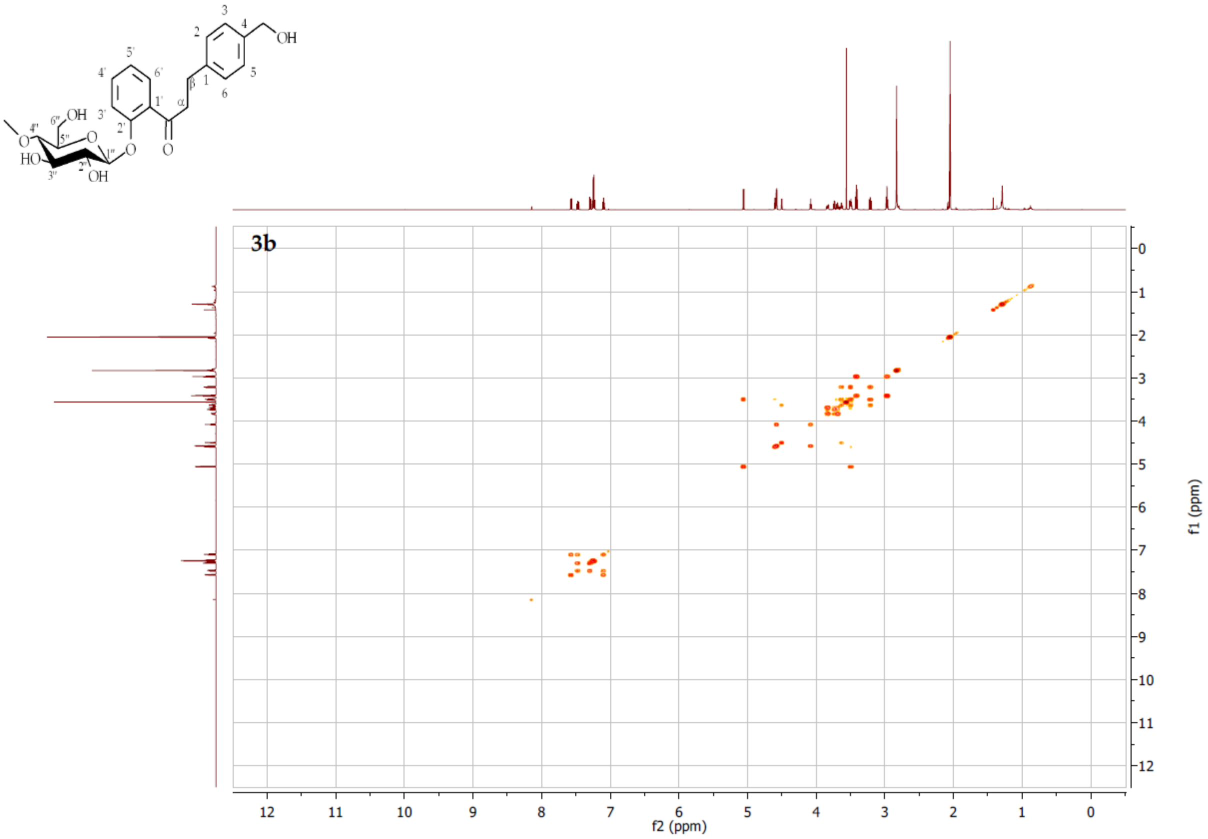

Figure S53. COSY contour map – ^1H x ^1H of 4-hydroxymethyldihydrochalcone 2′-*O*-*β*-D-(4″-*O*-methyl)-glucopyranoside (**3b**)

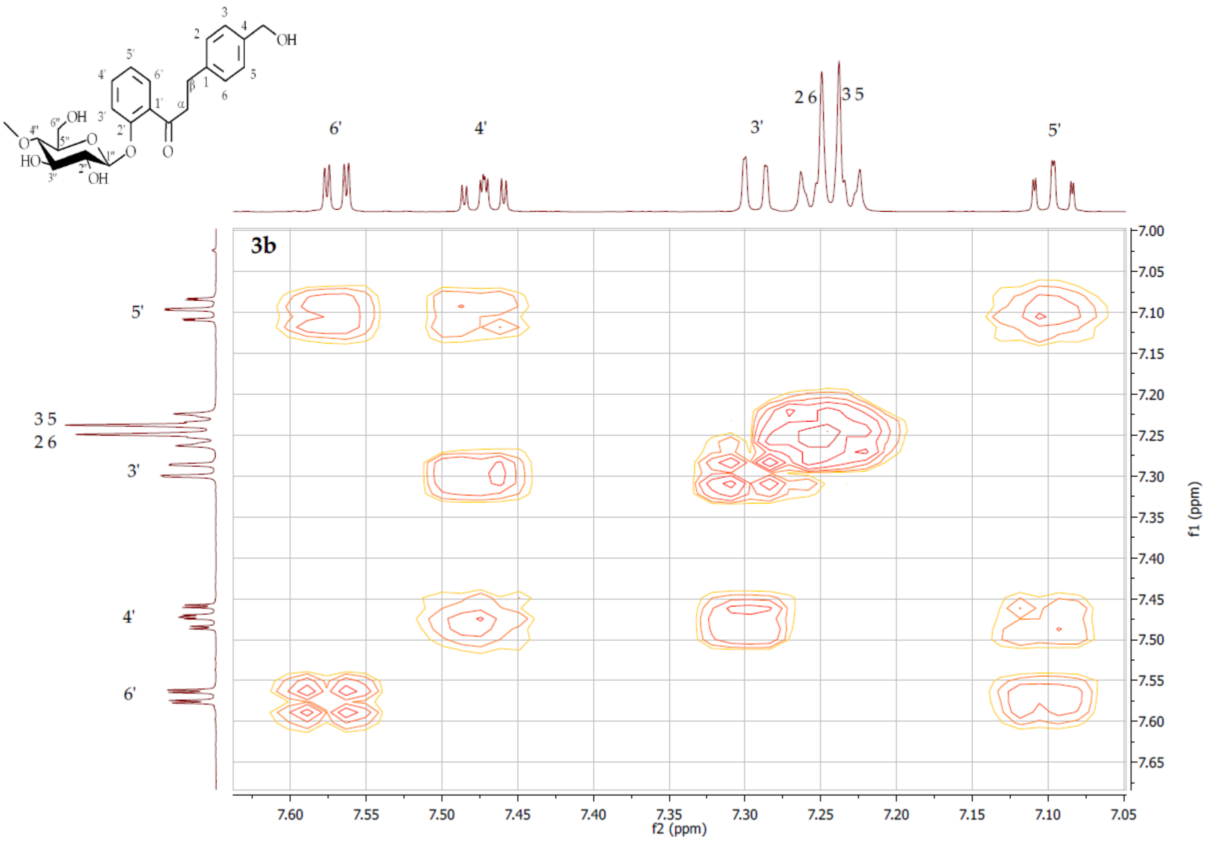

Figure S54. COSY contour map – ^1H x ^1H expansion of 4-hydroxymethyldihydrochalcone 2′-*O*-*β*-D-(4″-*O*-methyl)-glucopyranoside (**3b**)

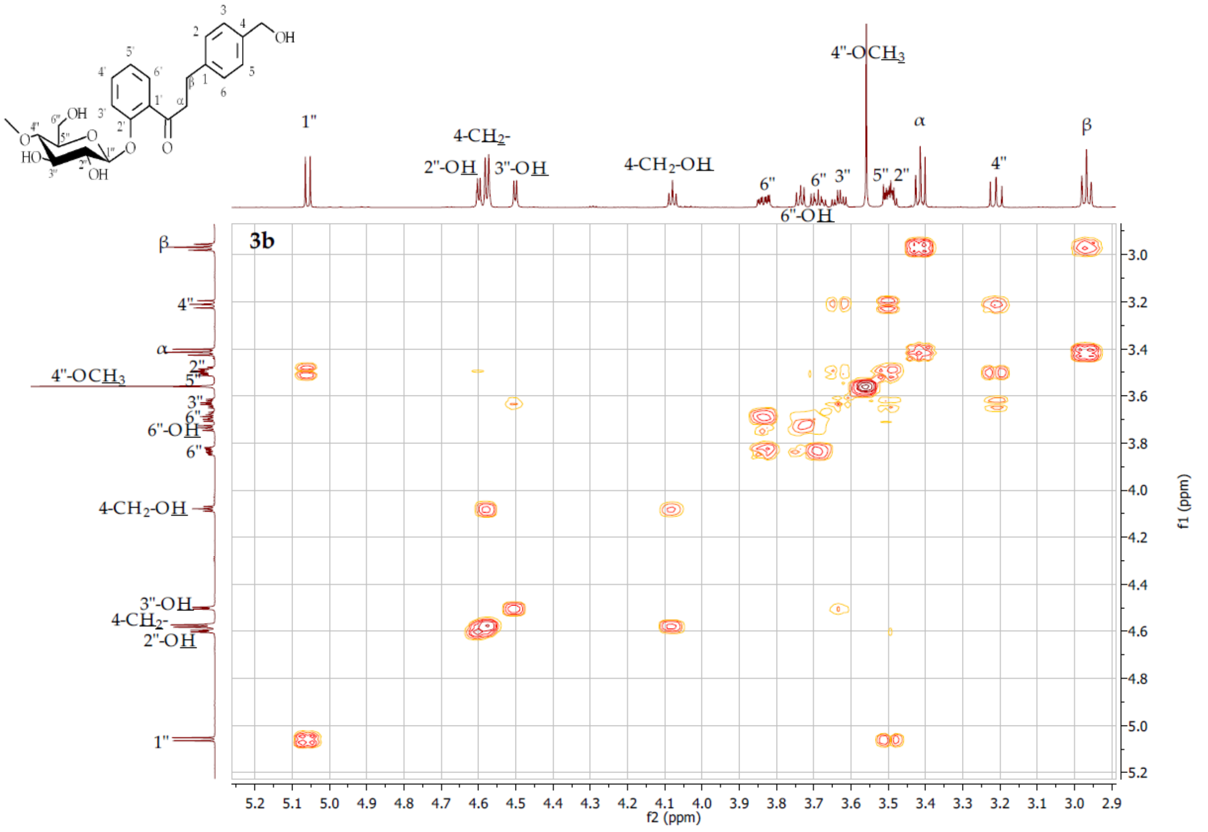

Figure S55. COSY contour map – ^1H x ^1H expansion of 4-hydroxymethyldihydrochalcone 2′-*O*-*β*-D-(4″-*O*-methyl)-glucopyranoside (**3b**)

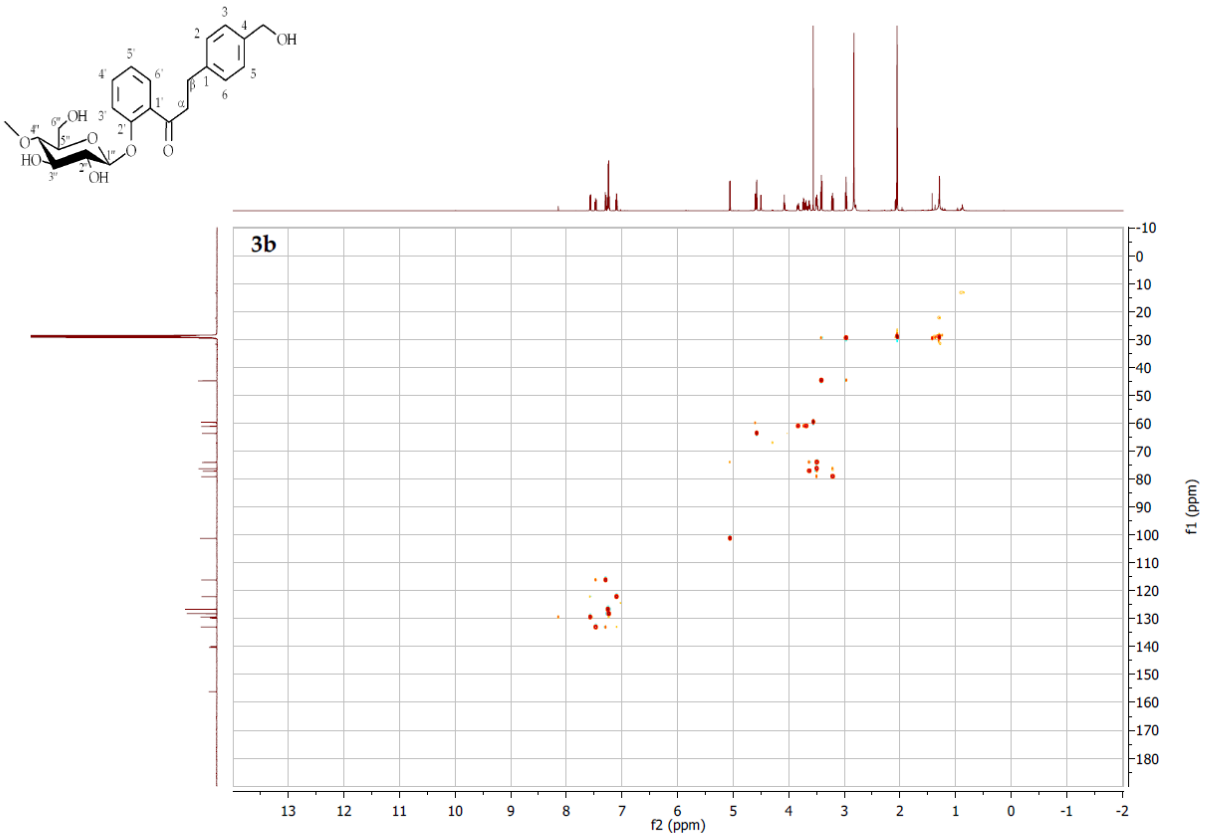

Figure S56. HSQC contour map – ^{1}H x ^{13}C of 4-hydroxymethyldihydrochalcone 2′-*O*-*β*-D-(4″-*O*-methyl)-glucopyranoside (**3b**)

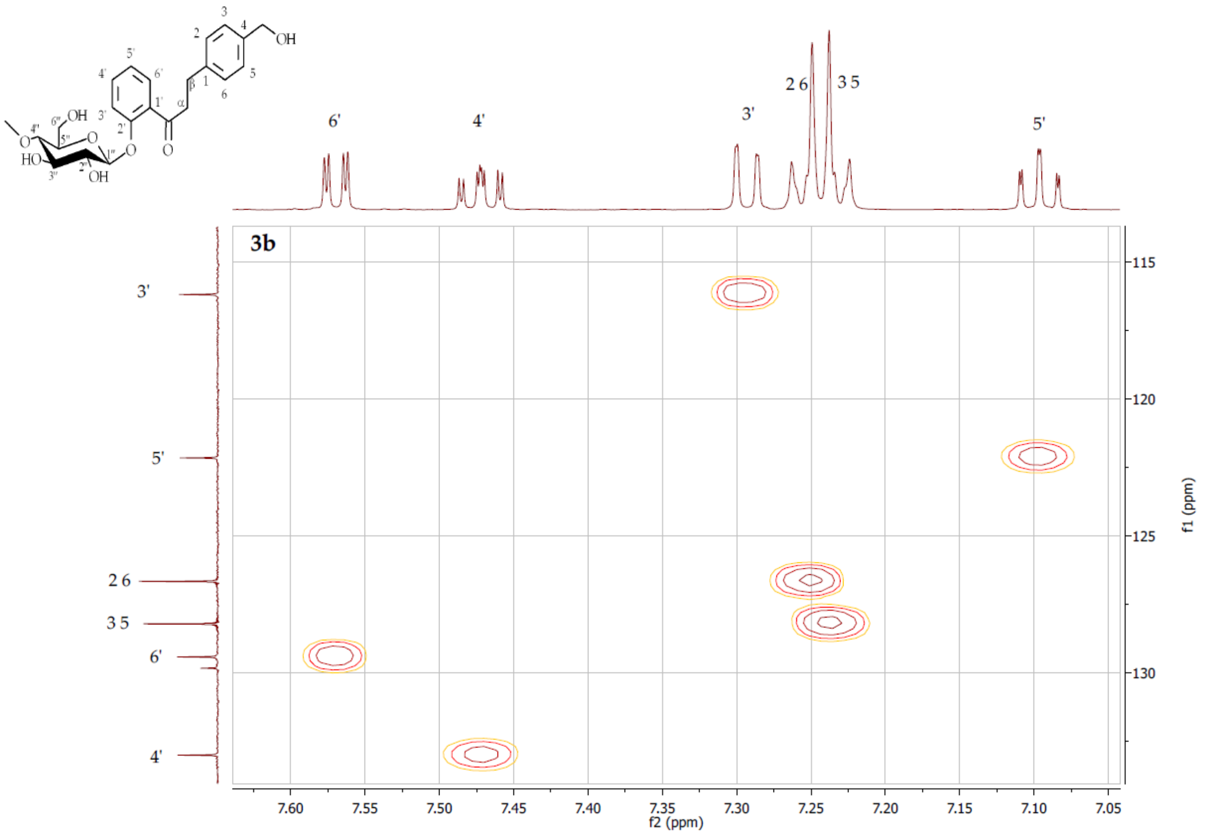

Figure S57. HSQC contour map – ^{1}H x ^{13}C expansion of 4-hydroxymethyldihydrochalcone 2′-*O*-*β*-D-(4″-*O*-methyl)-glucopyranoside (**3b**)

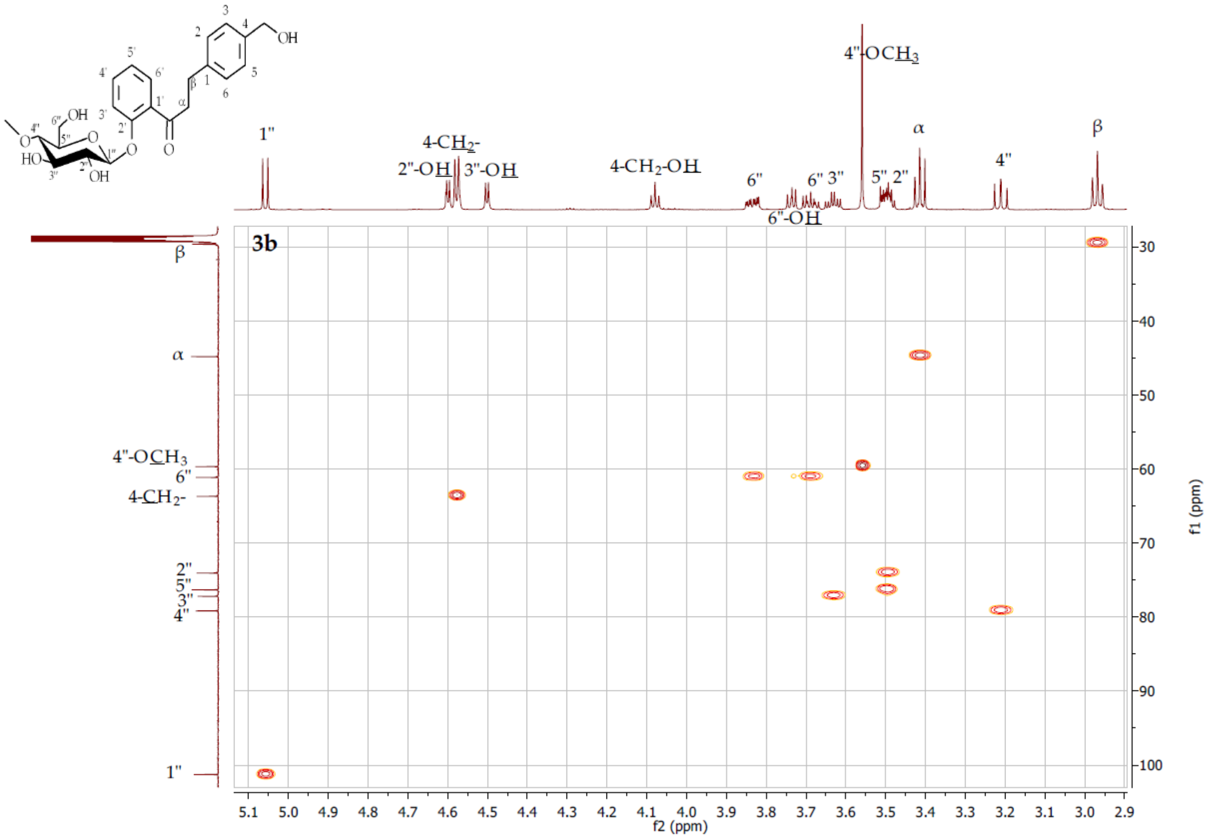

Figure S58. HSQC contour map – ^{1}H x ^{13}C expansion of 4-hydroxymethyldihydrochalcone 2′-*O*-*β*-D-(4″-*O*-methyl)-glucopyranoside (**3b**)

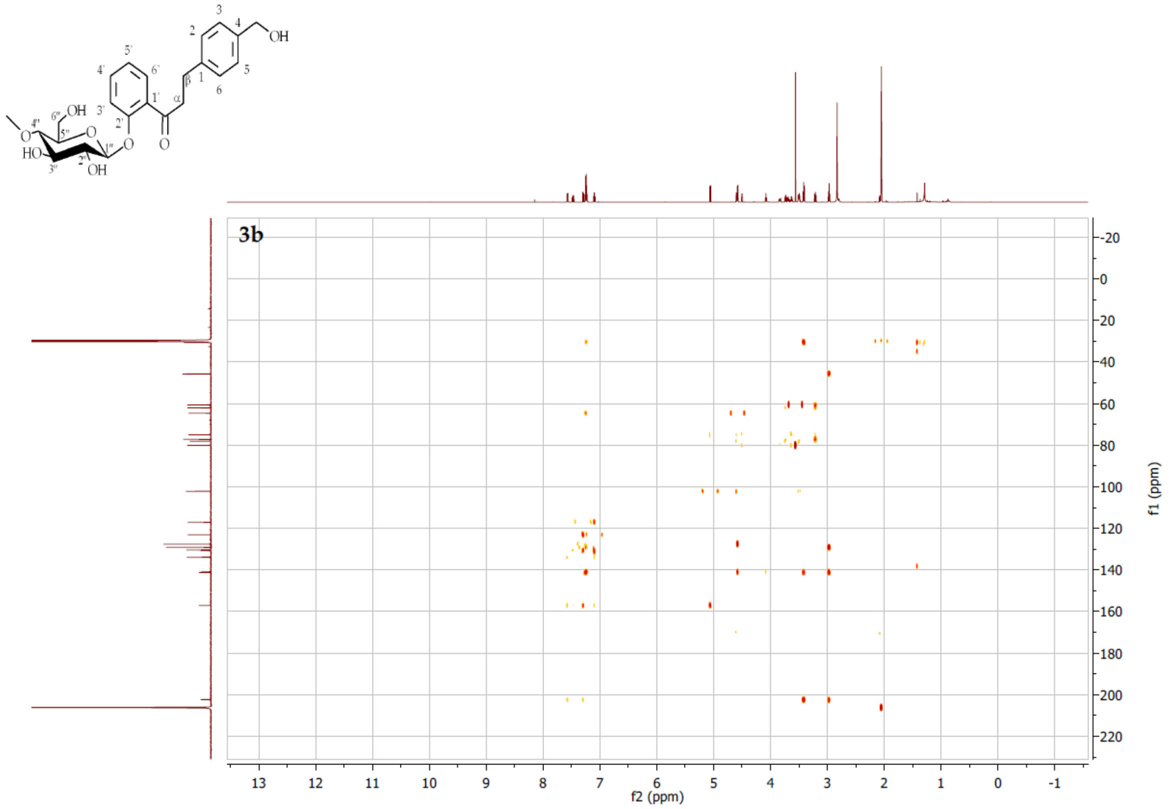

Figure S59. HMBC contour map – ^{1}H x ^{13}C of 4-hydroxymethyldihydrochalcone 2′-*O*-*β*-D-(4″-*O*-methyl)-glucopyranoside (**3b**)

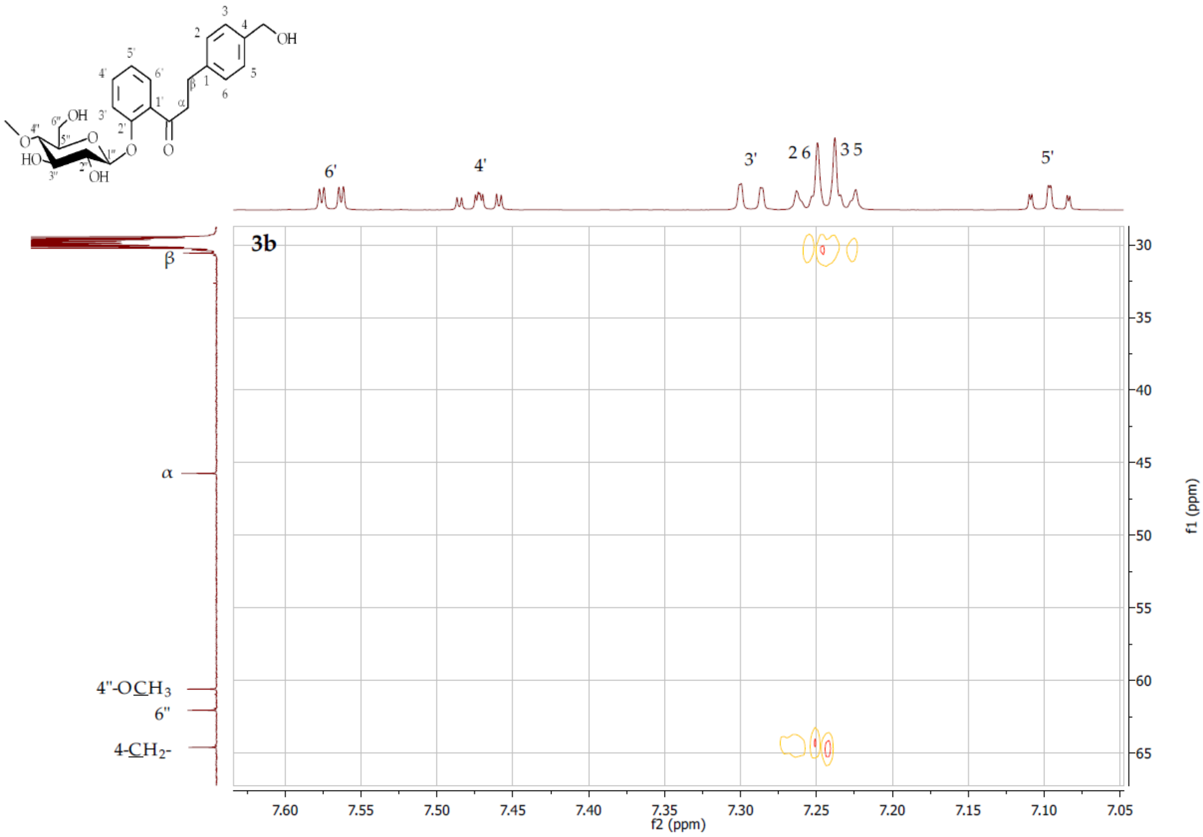

Figure S60. HMBC contour map – ^{1}H x ^{13}C expansion of 4-hydroxymethyldihydrochalcone 2′-*O*-*β*-D-(4″-*O*-methyl)-glucopyranoside (**3b**)

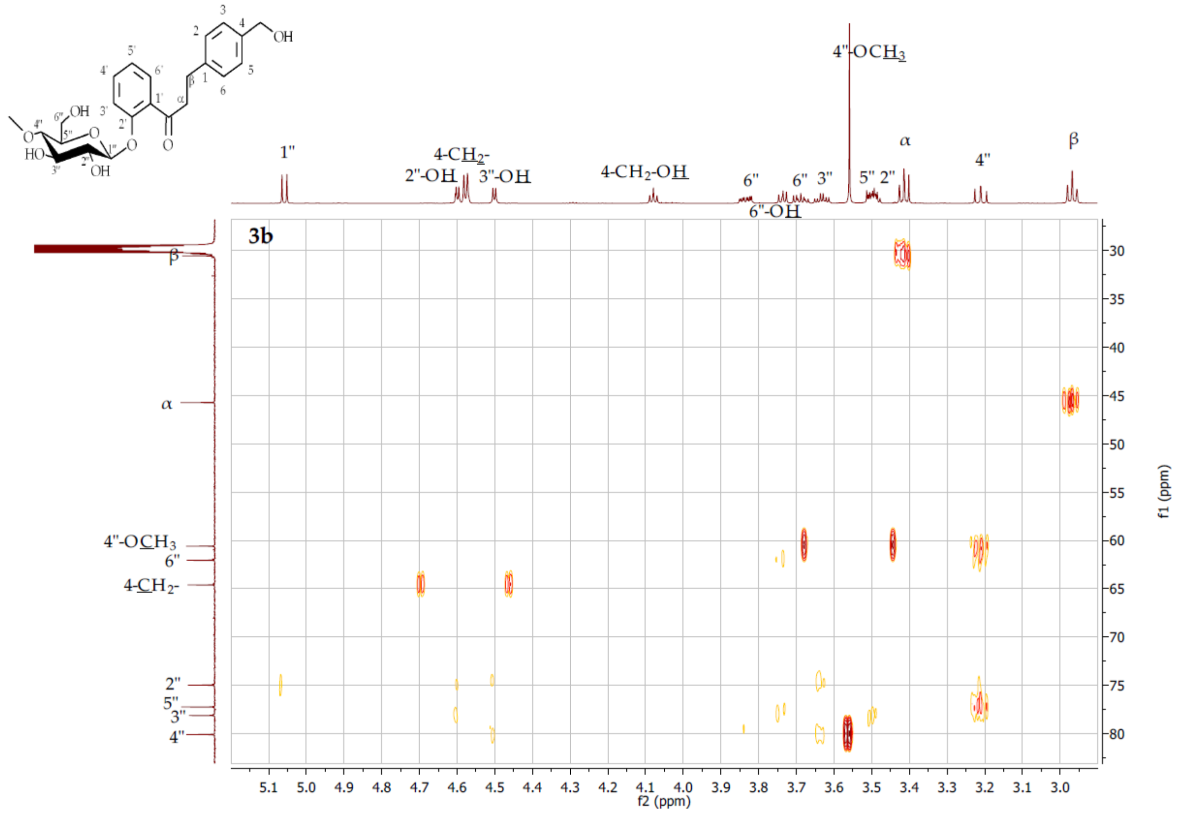

Figure S61. HMBC contour map – ^{1}H x ^{13}C expansion of 4-hydroxymethyldihydrochalcone 2′-*O*-*β*-D-(4″-*O*-methyl)-glucopyranoside (**3b**)

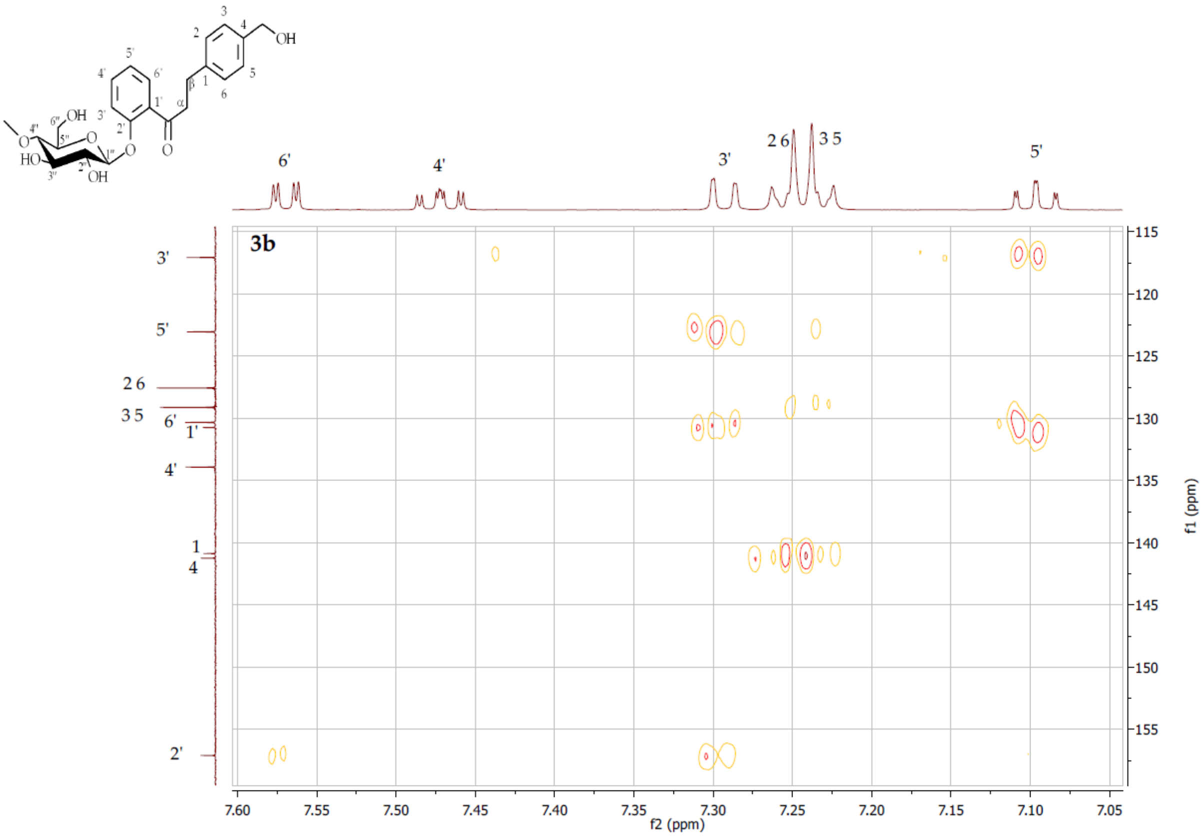

Figure S62. HMBC contour map – ^{1}H x ^{13}C expansion of 4-hydroxymethyldihydrochalcone 2′-*O*-*β*-D-(4″-*O*-methyl)-glucopyranoside (**3b**)

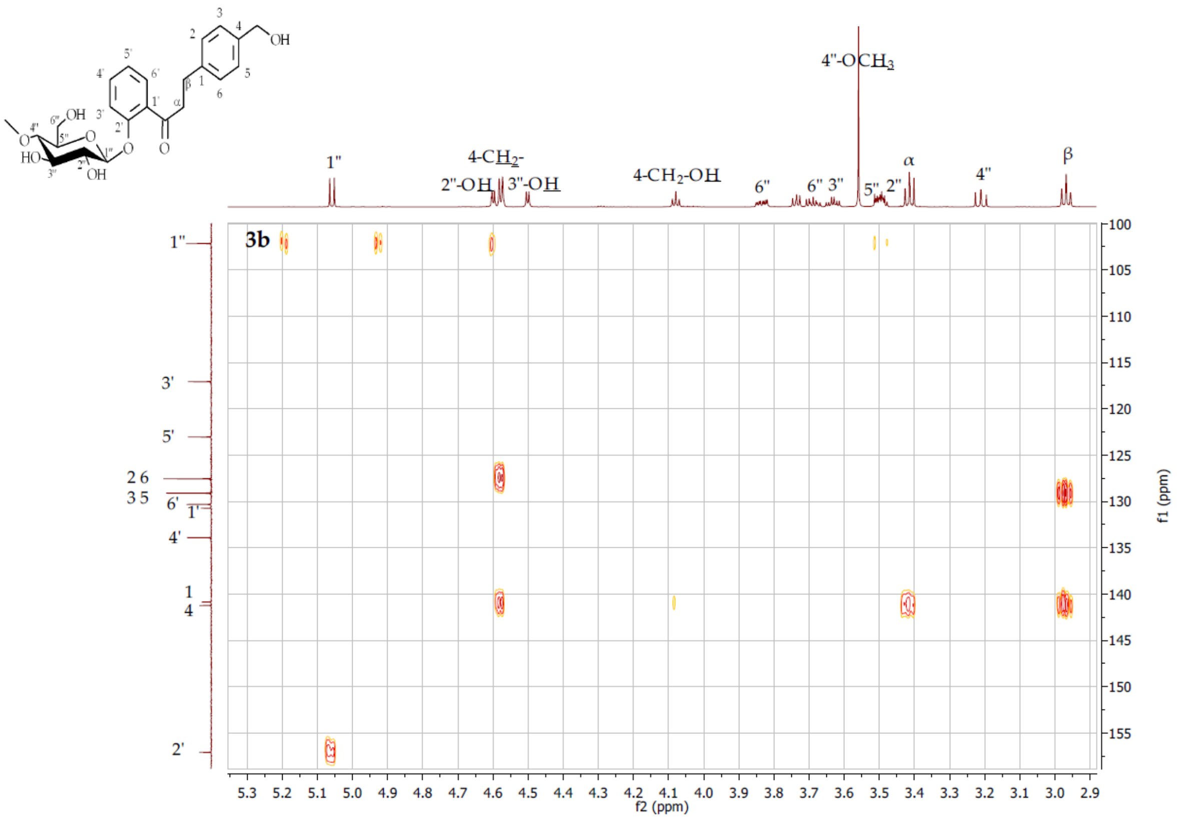

Figure S63. HMBC contour map – ^{1}H x ^{13}C expansion of 4-hydroxymethyldihydrochalcone 2′-*O*-*β*-D-(4″-*O*-methyl)-glucopyranoside (**3b**)

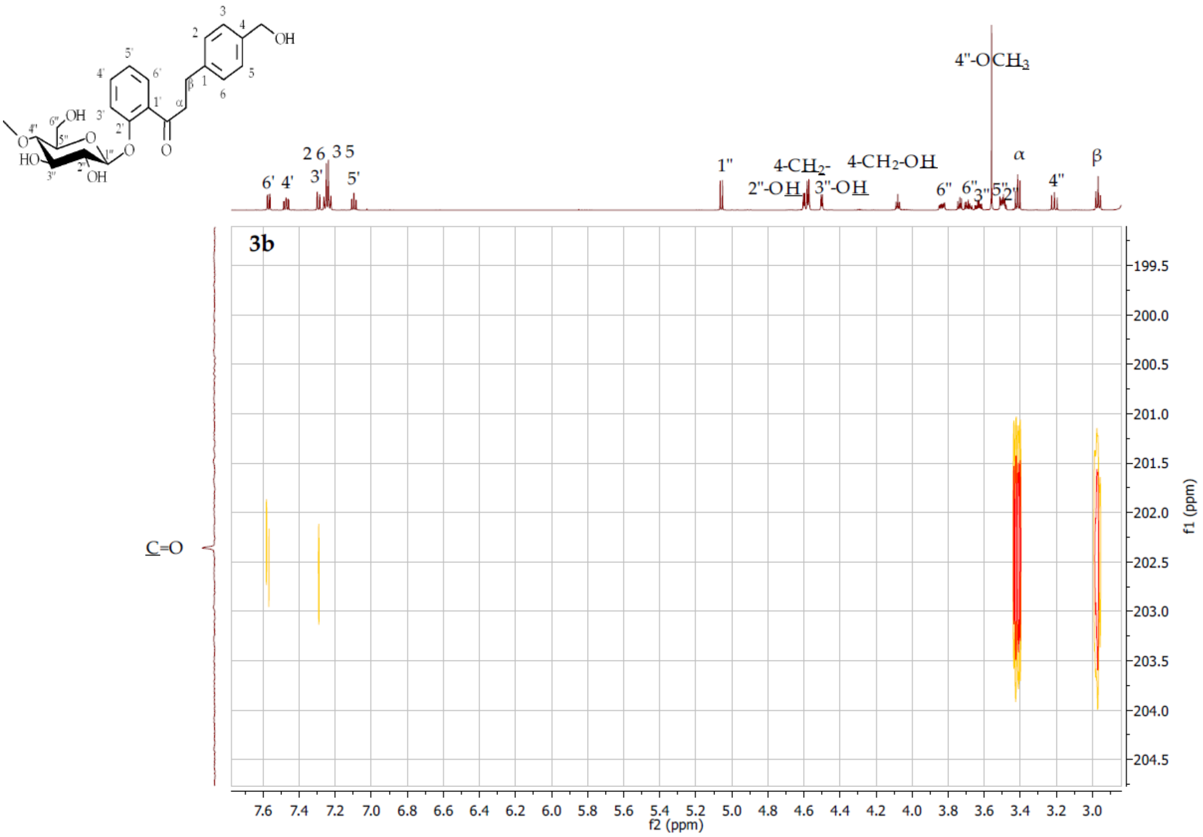

Figure S64. HMBC contour map – ^{1}H x ^{13}C expansion of 4-hydroxymethyldihydrochalcone 2′-*O*-*β*-D-(4″-*O*-methyl)-glucopyranoside (**3b**)

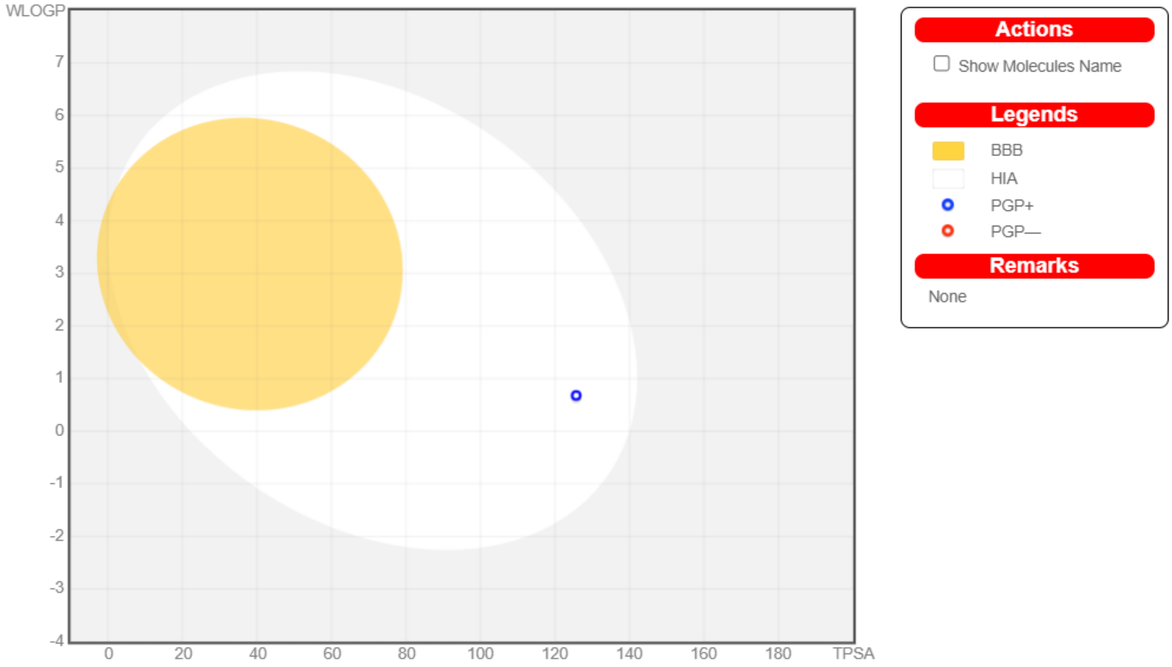

Molecule 1

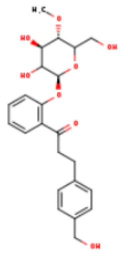

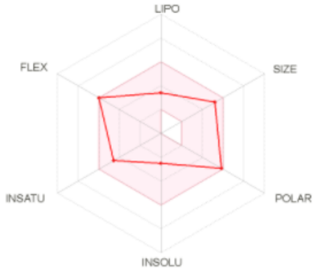

SMILES OCC1O[C@@H](Oc2ccccc2C(=O)CCc2ccc(cc2)CO)C([C@H]([C@@H]1OC)O)O

Physicochemical Properties	
Formula	C23H28O8
Molecular weight	432.46 g/mol
Num. heavy atoms	31
Num. arom. heavy atoms	12
Fraction Csp3	0.43
Num. rotatable bonds	9
Num. H-bond acceptors	8
Num. H-bond donors	4
Molar Refractivity	110.93
TPSA	125.68 Å²

Lipophilicity	
Log $P_{o/w}$ (iLOGP)	2.22
Log $P_{o/w}$ (XLOGP3)	0.44
Log $P_{o/w}$ (WLOGP)	0.68
Log $P_{o/w}$ (MLOGP)	-0.24
Log $P_{o/w}$ (SILICOS-IT)	2.02
Consensus Log $P_{o/w}$	1.02

Water Solubility	
Log *S* (ESOL)	-2.49
Solubility	1.40e+00 mg/ml ; 3.23e-03 mol/l
Class	Soluble
Log *S* (Ali)	-2.65
Solubility	9.75e-01 mg/ml ; 2.25e-03 mol/l
Class	Soluble
Log *S* (SILICOS-IT)	-3.92
Solubility	5.21e-02 mg/ml ; 1.21e-04 mol/l
Class	Soluble

Pharmacokinetics	
GI absorption	High
BBB permeant	No
P-gp substrate	Yes
CYP1A2 inhibitor	No
CYP2C19 inhibitor	No
CYP2C9 inhibitor	No
CYP2D6 inhibitor	No
CYP3A4 inhibitor	No
Log K_p (skin permeation)	-8.63 cm/s

Druglikeness	
Lipinski	Yes; 0 violation
Ghose	Yes
Veber	Yes
Egan	Yes
Muegge	Yes
Bioavailability Score	0.55

Medicinal Chemistry	
PAINS	0 alert
Brenk	0 alert
Leadlikeness	No; 2 violations: MW>350, Rotors>7
Synthetic accessibility	5.00

Figure S65. 4-Hydroxymethyldihydrochalcone 2′-*O*-*β*-D-(4″-*O*-methyl)-glucopyranoside (**3b**) physicochemical and ADME parameters prediction using the SwissADME modelling

All ○ Pa>Pi ○ Pa>0,3 ○ Pa>0,7 ok

Pa	Pi	Activity
0,948	0,001	3-Phytase inhibitor
0,943	0,004	CDP-glycerol glycerophosphotransferase inhibitor
0,914	0,001	Lactase inhibitor
0,897	0,002	Monophenol monooxygenase inhibitor
0,873	0,008	Alkenylglycerophosphocholine hydrolase inhibitor
0,870	0,007	Sugar-phosphatase inhibitor
0,864	0,003	Beta-mannosidase inhibitor
0,860	0,006	Anaphylatoxin receptor antagonist
0,857	0,010	Benzoate-CoA ligase inhibitor
0,836	0,004	Anticarcinogenic

Figure S66. 4-Hydroxymethyldihydrochalcone 2′-*O*-*β*-D-(4″-*O*-methyl)-glucopyranoside (**3b**) biological activity prediction using the Way2Drug Pass online modelling

Name	Confidence	ChEMBL ID
Clostridium ramosum	0.6565	CHEMBL614971
Actinomyces meyeri	0.6090	CHEMBL612289
RESISTANT Acinetobacter pittii	0.6066	CHEMBL3140321
Mycobacterium mageritense	0.5953	CHEMBL612959
Clostridium cadaveris	0.5769	CHEMBL614970
RESISTANT Mycobacterium ulcerans	0.5751	CHEMBL612965
RESISTANT Staphylococcus aureus subsp. aureus RN4220	0.5275	CHEMBL2366906
Streptococcus oralis	0.4533	CHEMBL613305
Lactobacillus plantarum	0.4445	CHEMBL614973
Staphylococcus lugdunensis	0.4419	CHEMBL613303
Nocardia transvalensis	0.4393	CHEMBL613234
Clostridium sordellii	0.4174	CHEMBL613072
RESISTANT Chlamydia trachomatis	0.3922	CHEMBL614606
Streptococcus sanguinis	0.3656	CHEMBL612314
Nocardia otitidiscaviarum	0.3600	CHEMBL613233

Figure S67. 4-Hydroxymethyldihydrochalcone 2′-*O*-*β*-D-(4″-*O*-methyl)-glucopyranoside (**3b**) antibacterial activity prediction using the Way2Drug AntiBac-Pred modelling

Name	Confidence	ChEMBL ID
Rhizopus oryzae	0.4848	CHEMBL612306
Absidia corymbifera	0.4111	CHEMBL612369
Trichophyton mentagrophytes	0.2890	CHEMBL613162
Saccharomyces cerevisiae	0.2196	CHEMBL361
Epidermophyton floccosum	0.2174	CHEMBL612386
Mucor	0.2038	CHEMBL612521
Aspergillus niger	0.1886	CHEMBL358
Yarrowia lipolytica	0.1742	CHEMBL612844
Penicillium marneffei	0.1329	CHEMBL612994
Mucor hiemalis	0.1073	CHEMBL612949
Candida rugosa	0.0422	CHEMBL612869
Cryptococcus bacillisporus	0.0181	CHEMBL615035
Galactomyces geotrichum	0.0056	CHEMBL613775

Figure S68. 4-Hydroxymethyldihydrochalcone 2′-*O*-*β*-D-(4″-*O*-methyl)-glucopyranoside (**3b**) antifungal activity prediction using the Way2Drug AntiFun-Pred modelling

Virus	Protein target	Confidence
Severe acute respiratory syndrome coronavirus 2	Replicase polyprotein 1ab	0.6833
Varicella-zoster virus (strain Dumas) (HHV-3) (Human herpesvirus 3)	Thymidine kinase	0.0144

Figure S69. 4-Hydroxymethyldihydrochalcone 2′-*O*-*β*-D-(4″-*O*-methyl)-glucopyranoside (**3b**) antiviral activity prediction using the Way2Drug AntiVir-Pred modelling

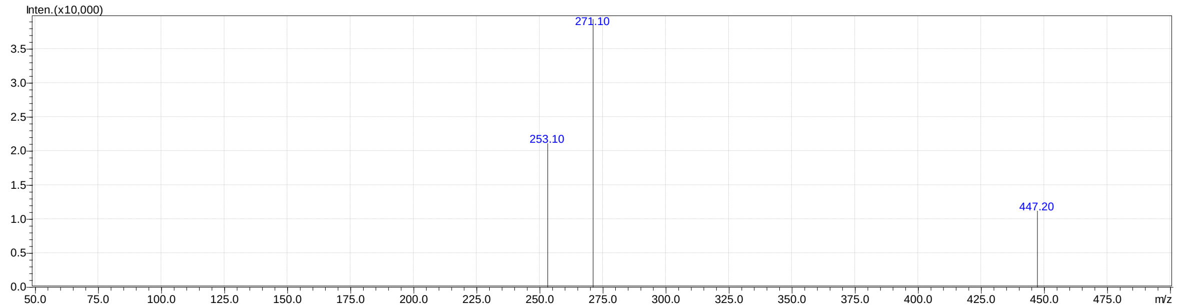

Figure S70. MS analysis 2′-hydroxy-4-hydroxymethylchalcone 5′-*O*-*β*-D-(4″-*O*-methyl)-glucopyranoside (**3c**)

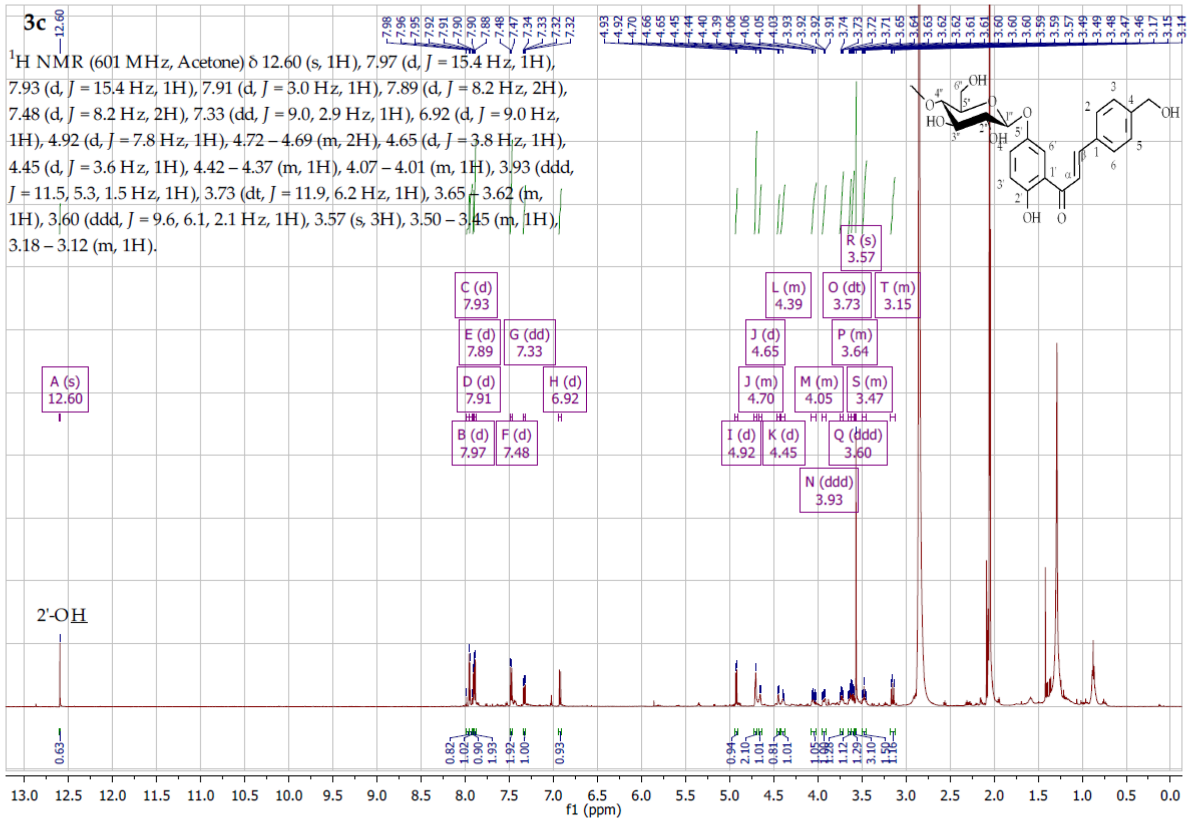

Figure S71. ^{1}H NMR spectrum (δ, acetone-d6, 600 MHz) of 2′-hydroxy-4-hydroxymethylchalcone 5′-*O*-*β*-D-(4″-*O*-methyl)-glucopyranoside (**3c**)

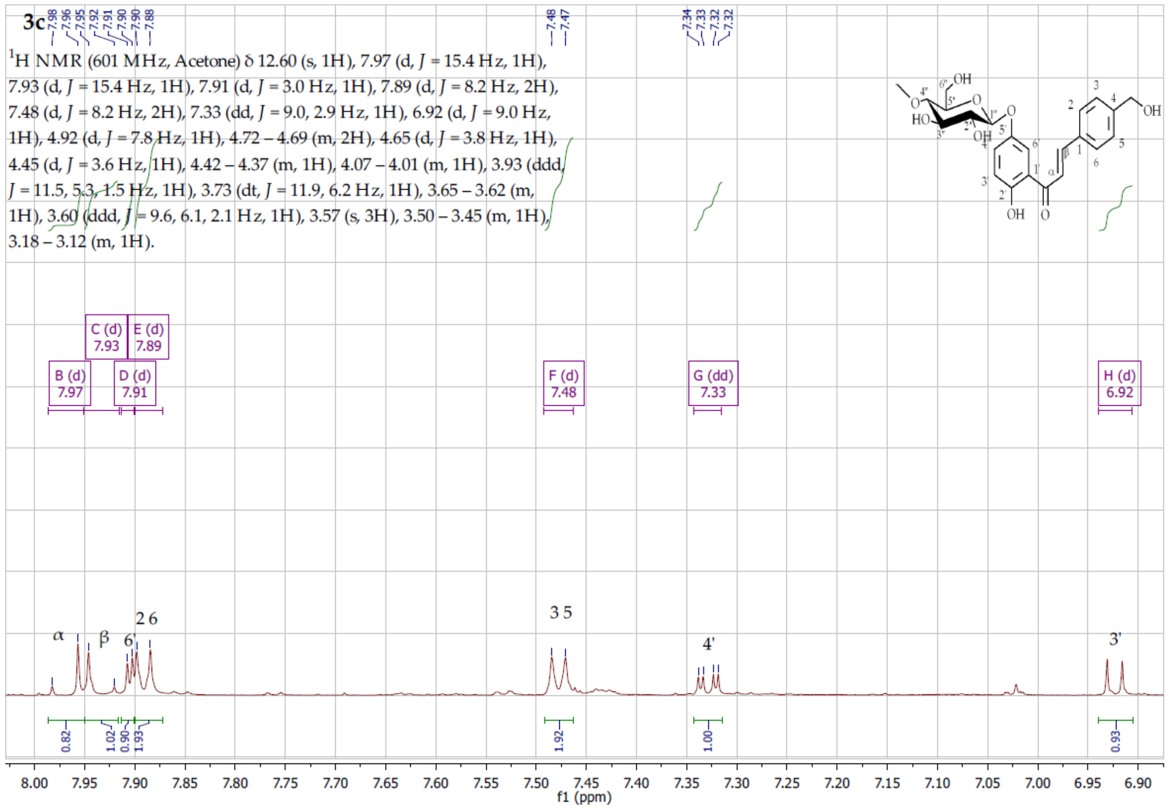

Figure S72. ^{1}H NMR spectrum expansion (δ, acetone-d6, 600 MHz) of 2′-hydroxy-4-hydroxymethylchalcone 5′-*O*-*β*-D-(4″-*O*-methyl)-glucopyranoside (**3c**)

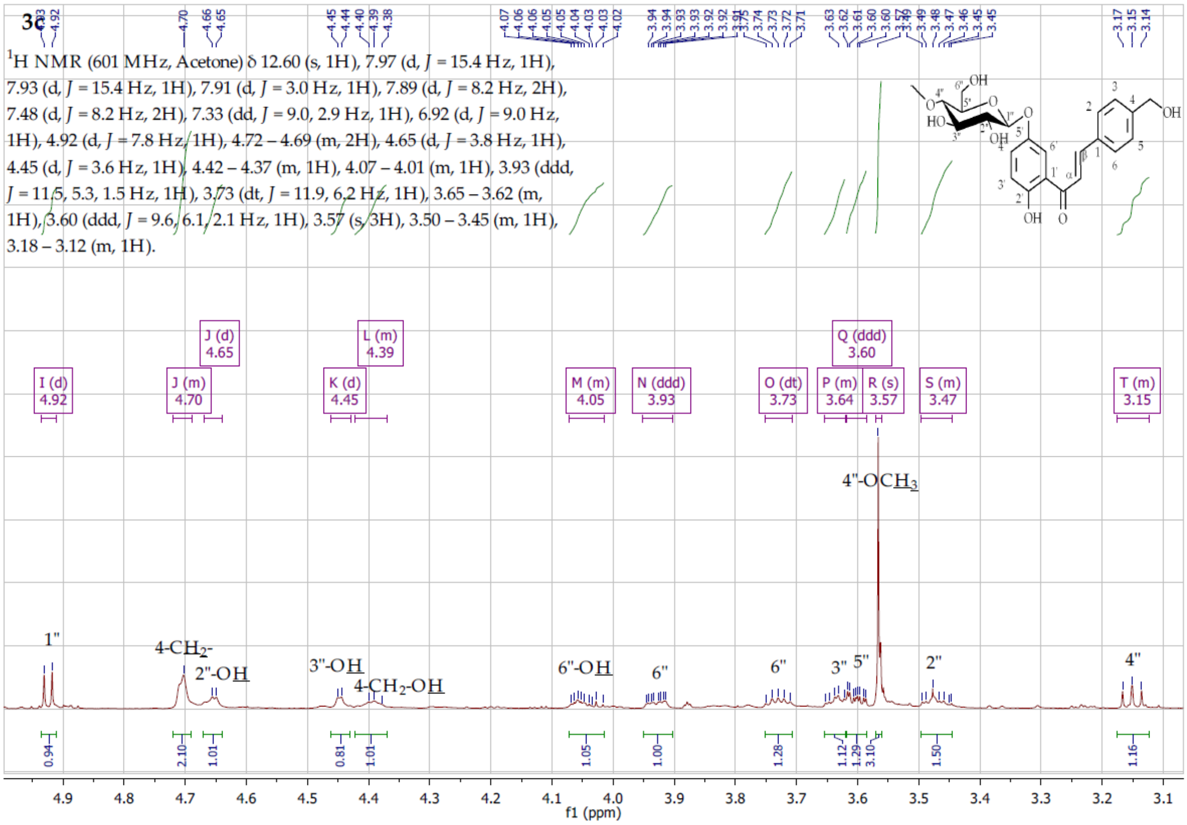

Figure S73. ^{1}H NMR spectrum expansion (*δ*, acetone-d6, 600 MHz) of 2′-hydroxy-4-hydroxymethylchalcone 5′-*O*-*β*-D-(4″-*O*-methyl)-glucopyranoside (**3c**)

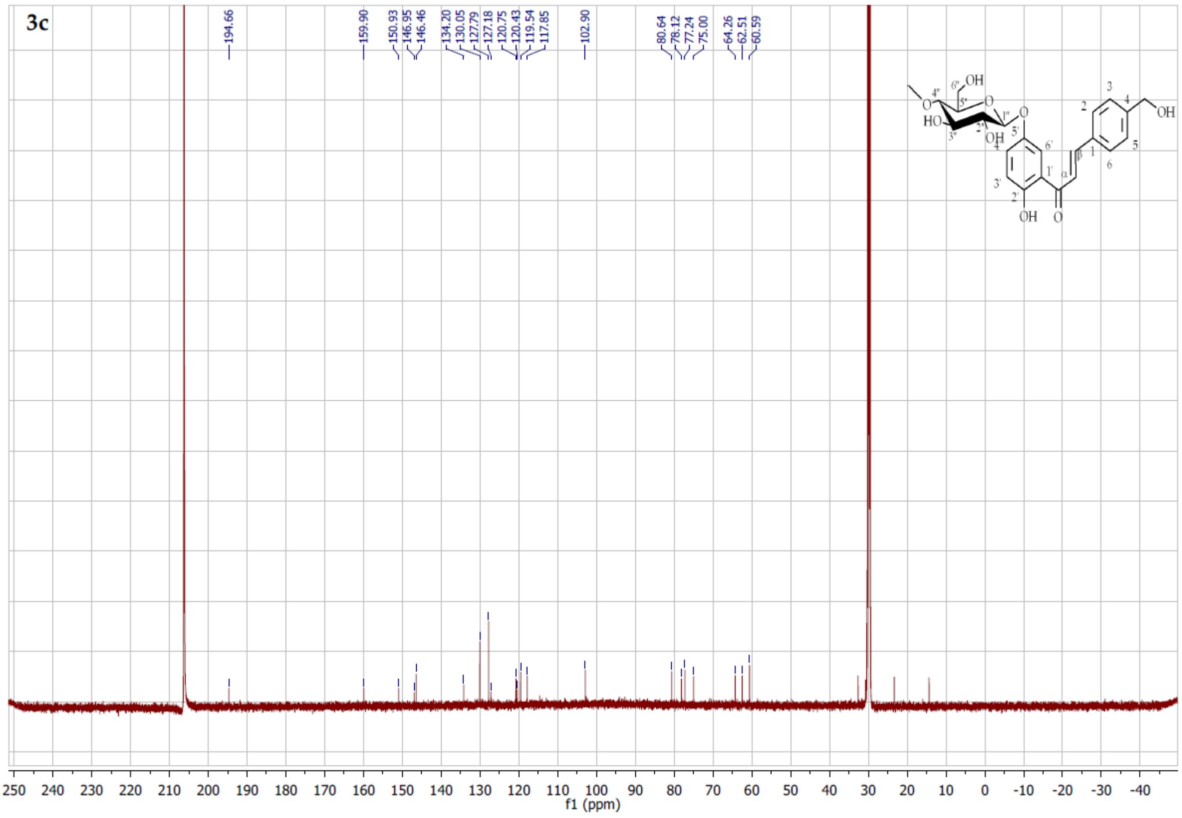

Figure S74. ^{13}C NMR spectrum expansion (*δ*, acetone-d6, 151 MHz) of 2′-hydroxy-4-hydroxymethylchalcone 5′-*O*-*β*-D-(4″-*O*-methyl)-glucopyranoside (**3c**)

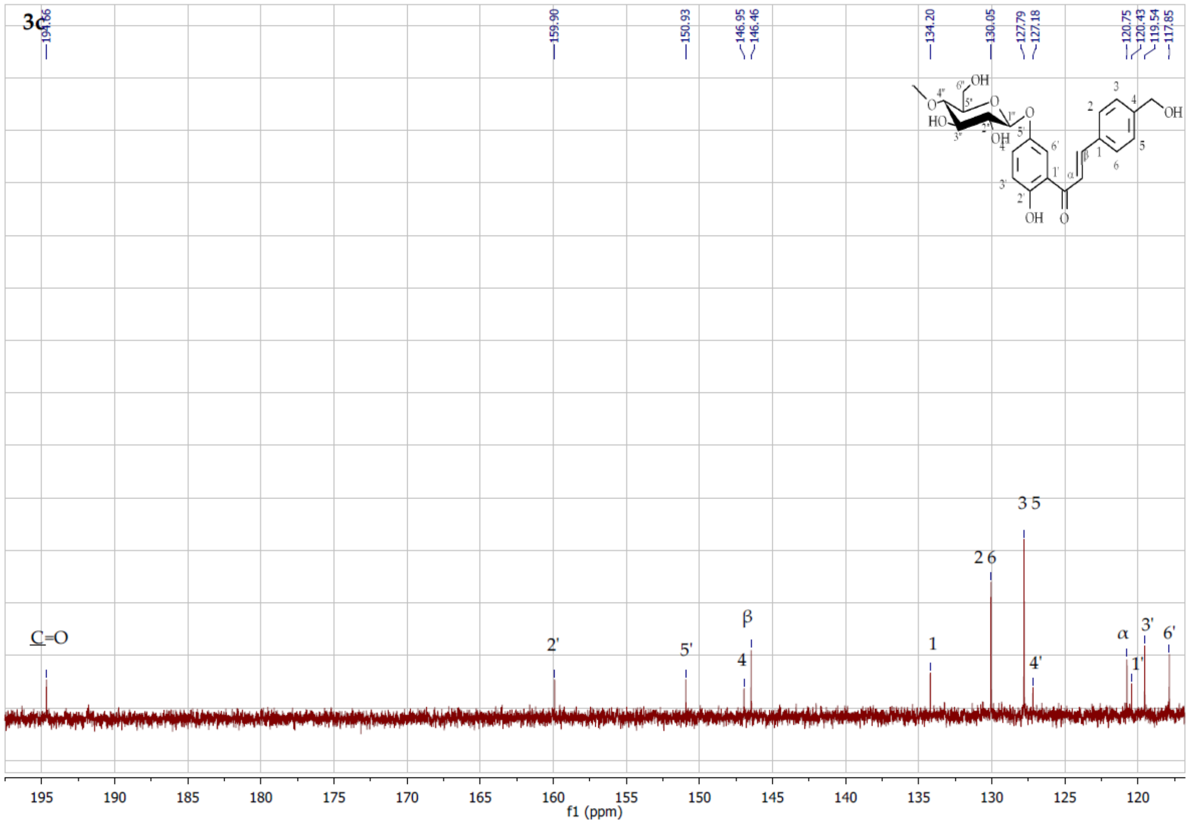

Figure S75. ^{13}C NMR spectrum expansion (*δ*, acetone-d6, 151 MHz) of 2′-hydroxy-4-hydroxymethylchalcone 5′-*O*-*β*-D-(4″-*O*-methyl)-glucopyranoside (**3c**)

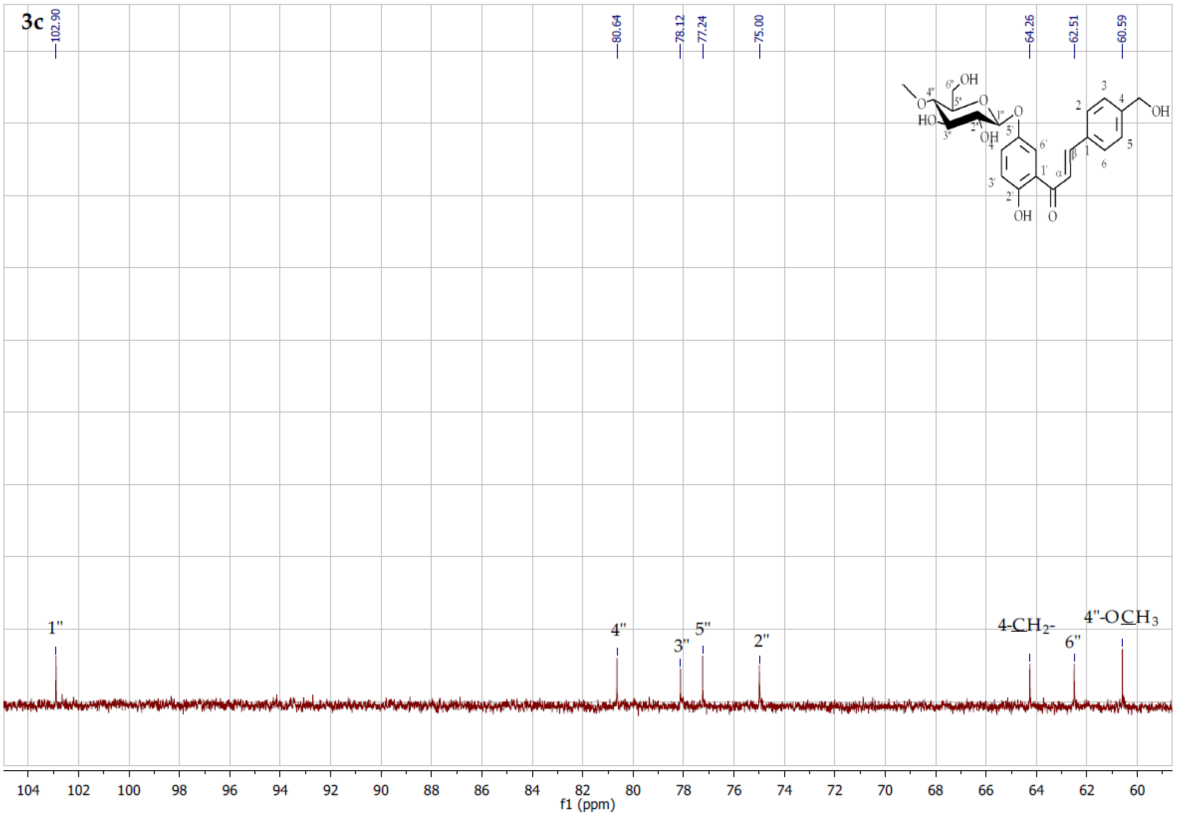

Figure S76. ^{13}C NMR spectrum expansion (*δ*, acetone-d6, 151 MHz) of 2′-hydroxy-4-hydroxymethylchalcone 5′-*O*-*β*-D-(4″-*O*-methyl)-glucopyranoside (**3c**)

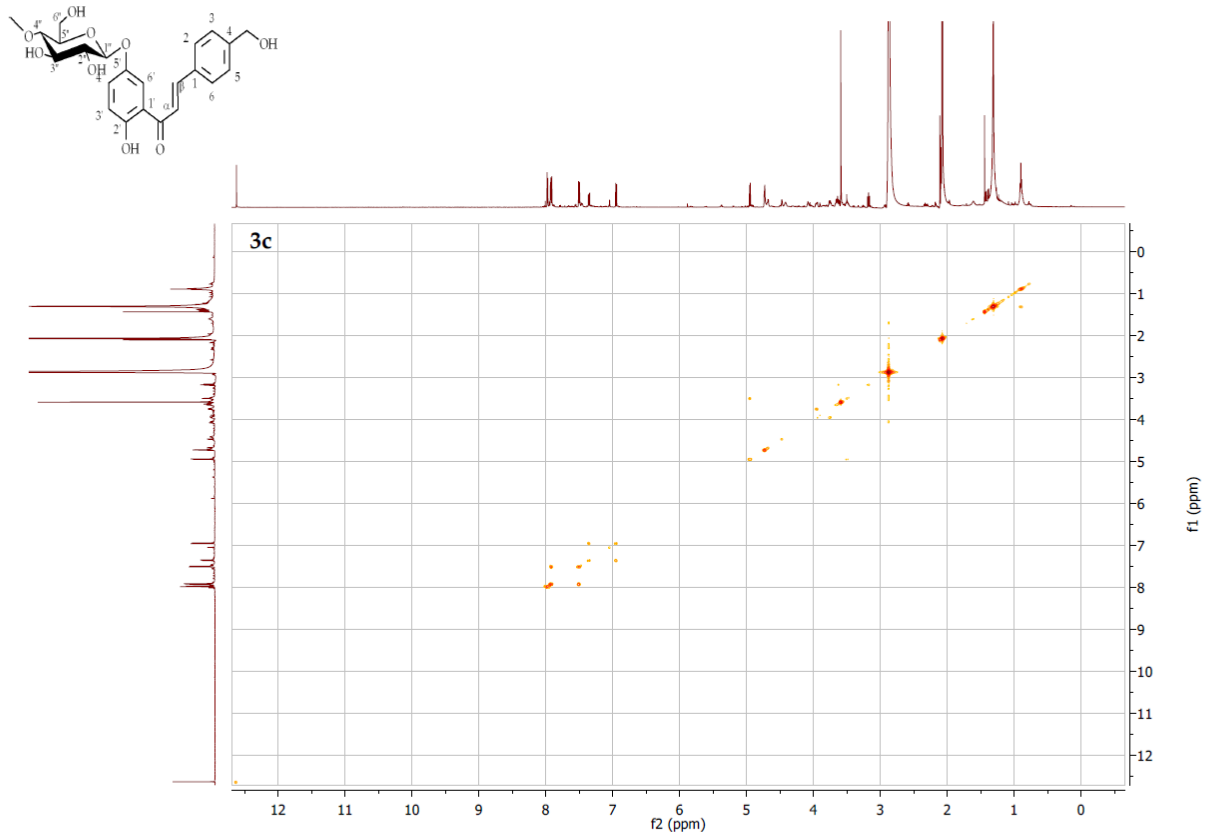

Figure S77. COSY contour map – ^{1}H x ^{1}H of 4-hydroxymethyldihydrochalcone 2′-hydroxy-4-hydroxymethylchalcone 5′-*O*-*β*-D-(4″-*O*-methyl)-glucopyranoside (**3c**)

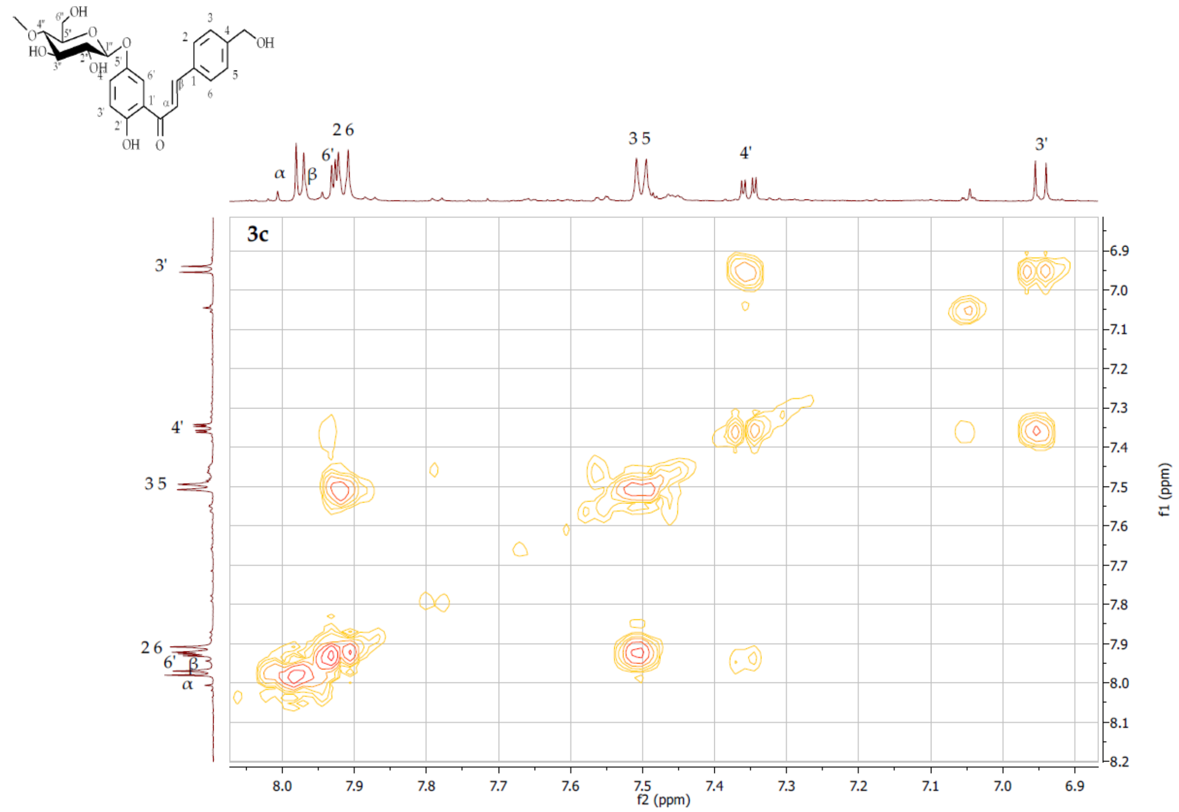

Figure S78. COSY contour map – ^{1}H x ^{1}H expansion of 2′-hydroxy-4-hydroxymethylchalcone 5′-*O*-*β*-D-(4″-*O*-methyl)-glucopyranoside (**3c**)

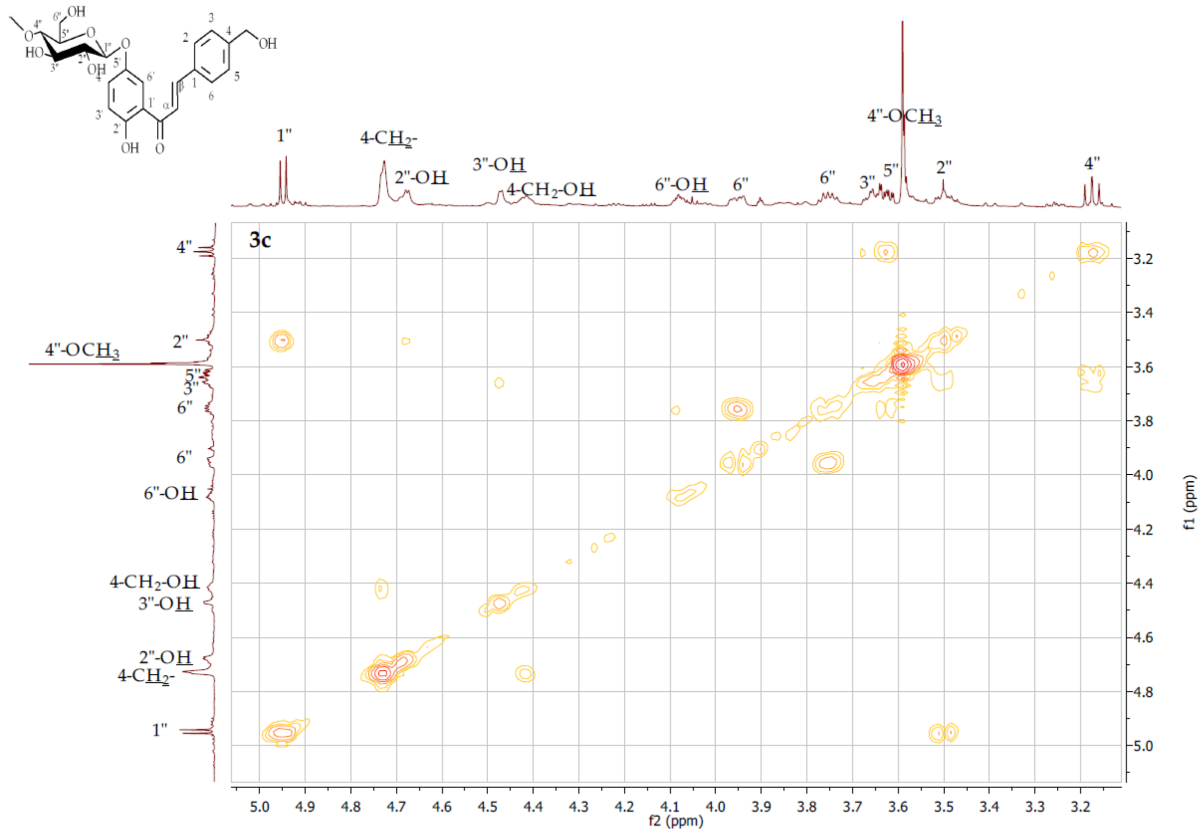

Figure S79. COSY contour map – ^{1}H x ^{1}H expansion of 2′-hydroxy-4-hydroxymethylchalcone 5′-*O*-*β*-D-(4″-*O*-methyl)-glucopyranoside (**3c**)

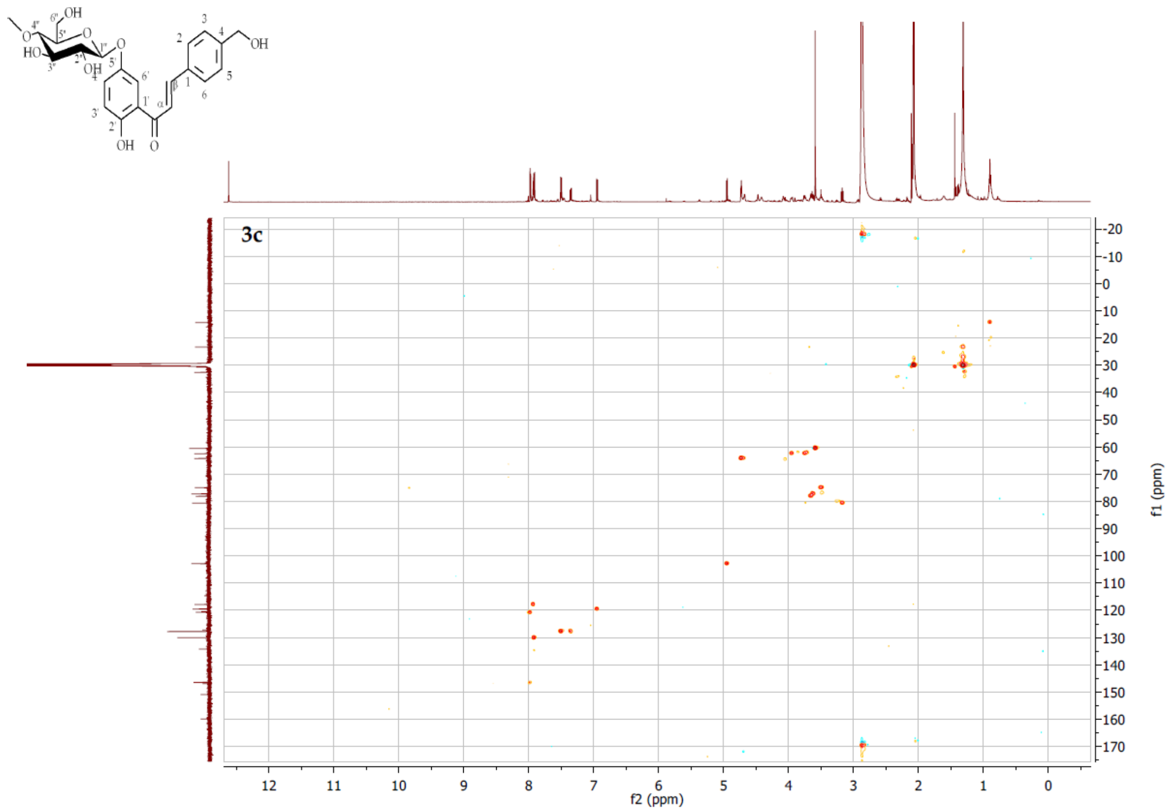

Figure S80. HSQC contour map – ^{1}H x ^{13}C of 2′-hydroxy-4-hydroxymethylchalcone 5′-*O*-*β*-D-(4″-*O*-methyl)-glucopyranoside (**3c**)

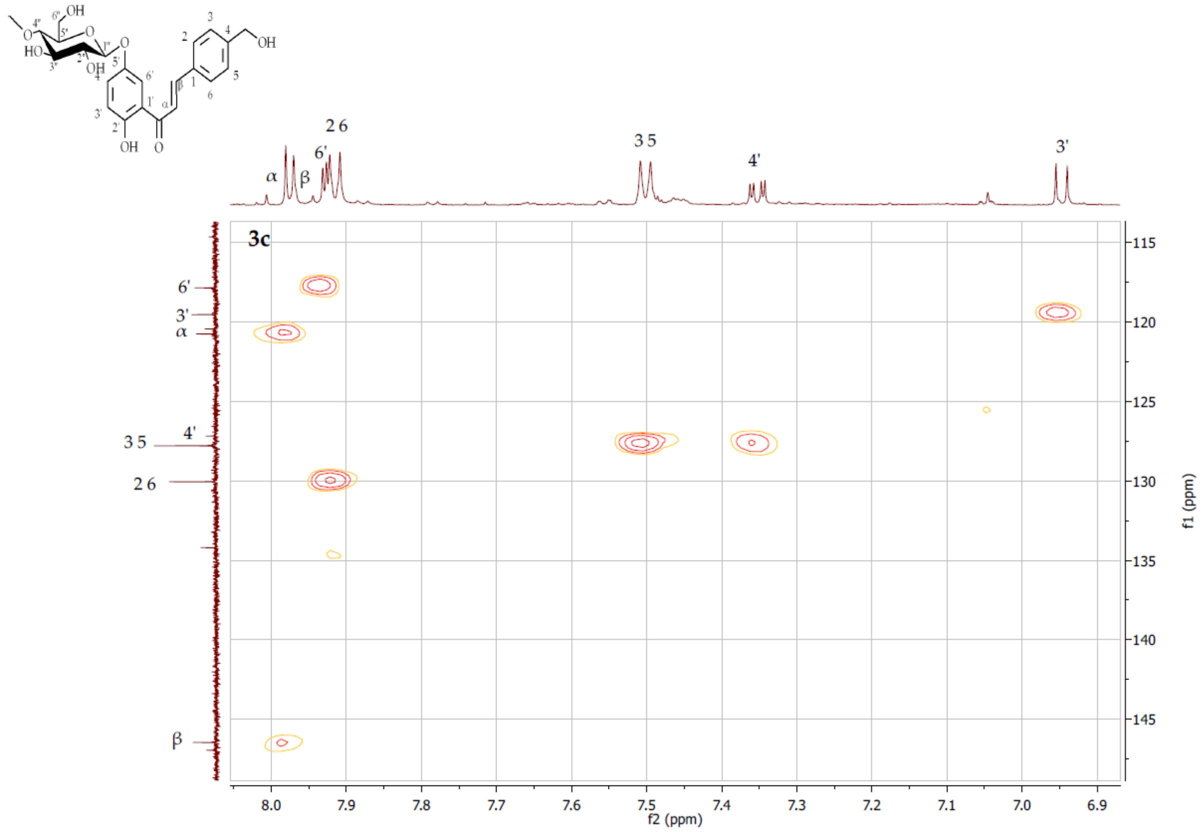

Figure S81. HSQC contour map – ^{1}H x ^{13}C expansion of 2′-hydroxy-4-hydroxymethylchalcone 5′-*O*-*β*-D-(4″-*O*-methyl)-glucopyranoside (**3c**)

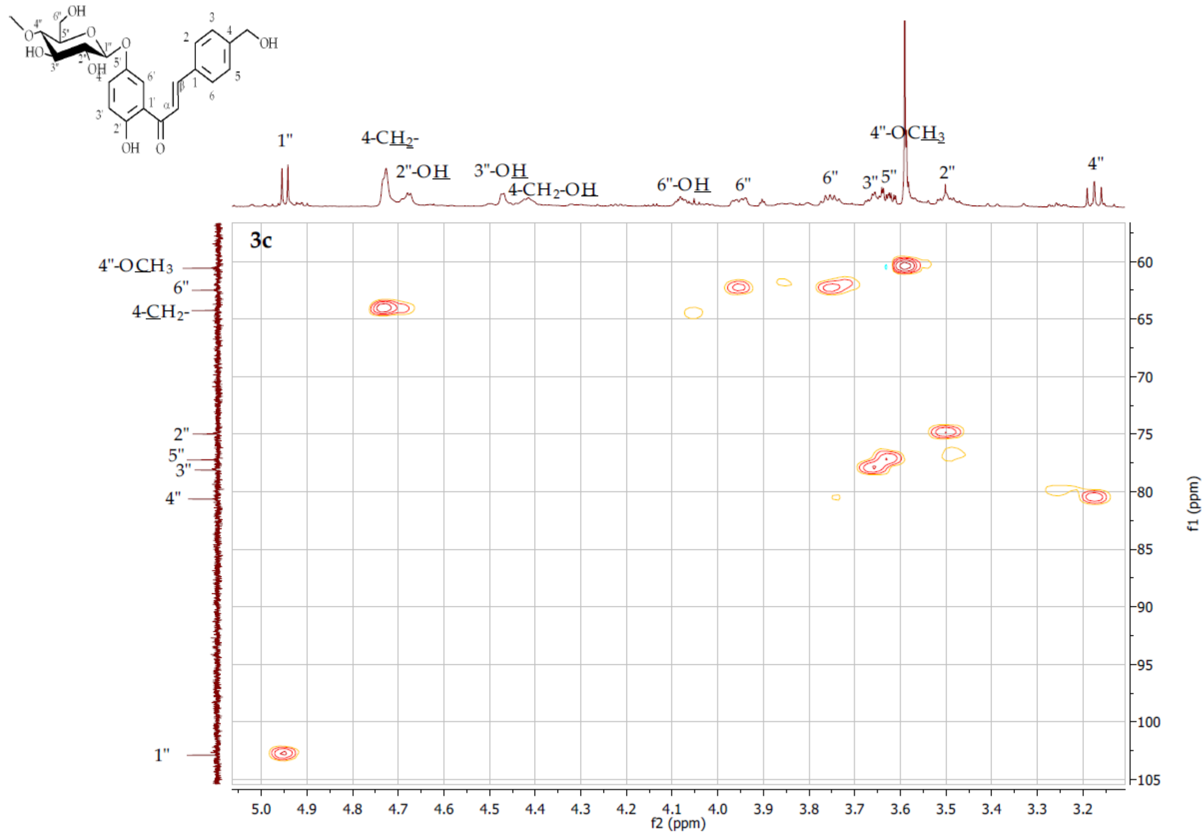

Figure S82. HSQC contour map – ^{1}H x ^{13}C expansion of 2′-hydroxy-4-hydroxymethylchalcone 5′-*O*-*β*-D-(4″-*O*-methyl)-glucopyranoside (**3c**)

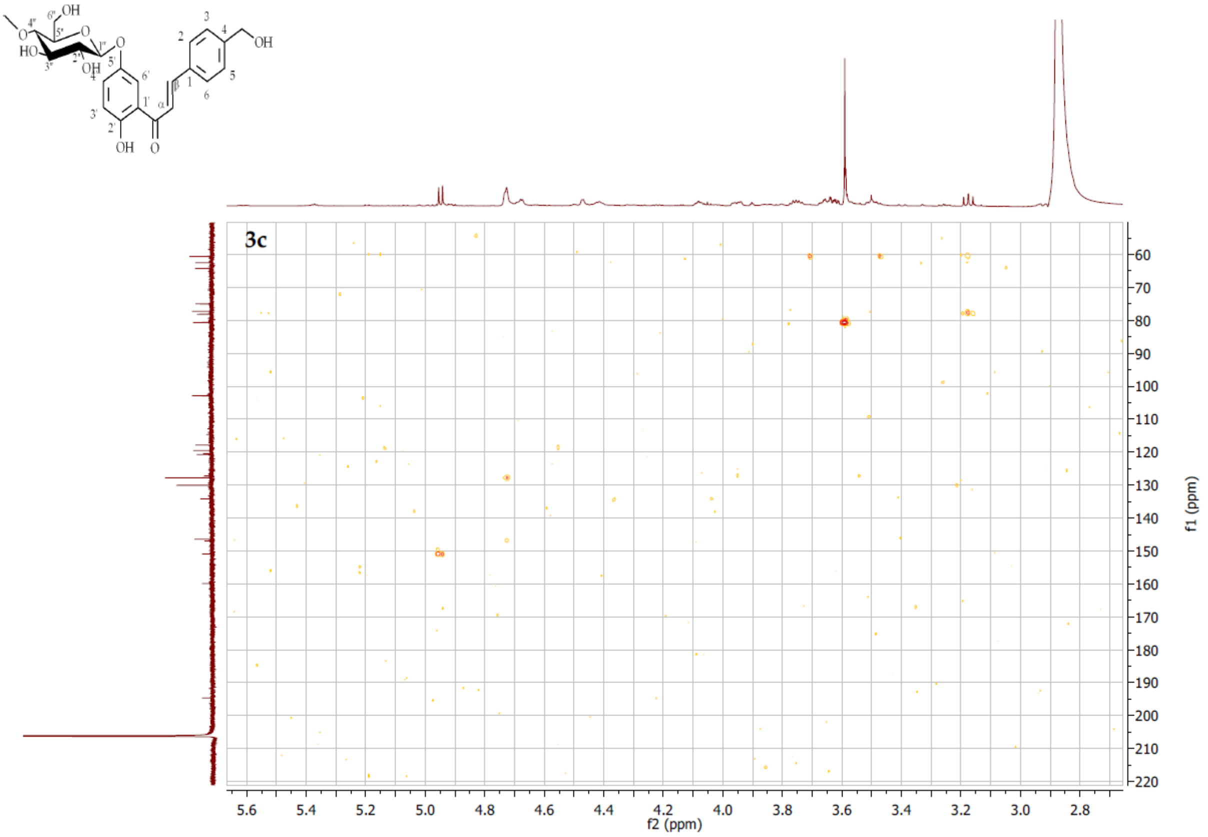

Figure S83. HMBC contour map – ^{1}H x ^{13}C of 2′-hydroxy-4-hydroxymethylchalcone 5′-*O*-*β*-D-(4″-*O*-methyl)-glucopyranoside (**3c**)

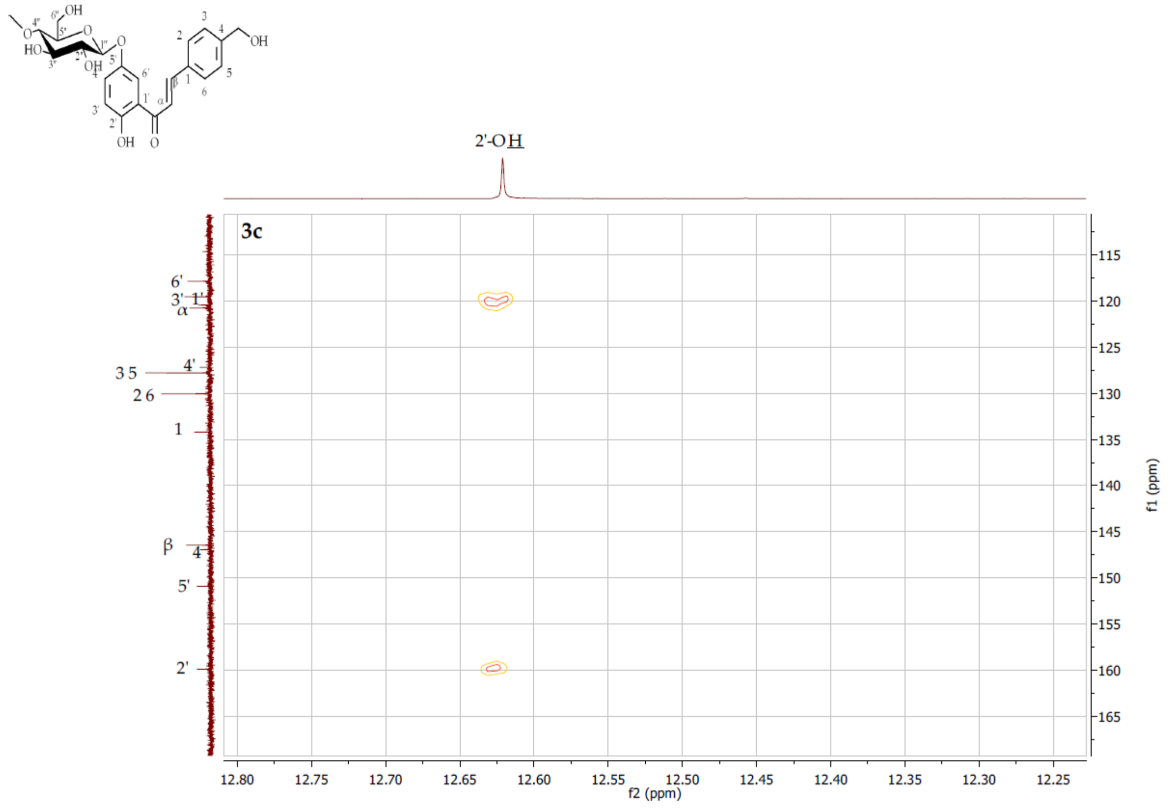

Figure S84. HMBC contour map – ^{1}H x ^{13}C expansion of 2′-hydroxy-4-hydroxymethylchalcone 5′-*O*-*β*-D-(4″-*O*-methyl)-glucopyranoside (**3c**)

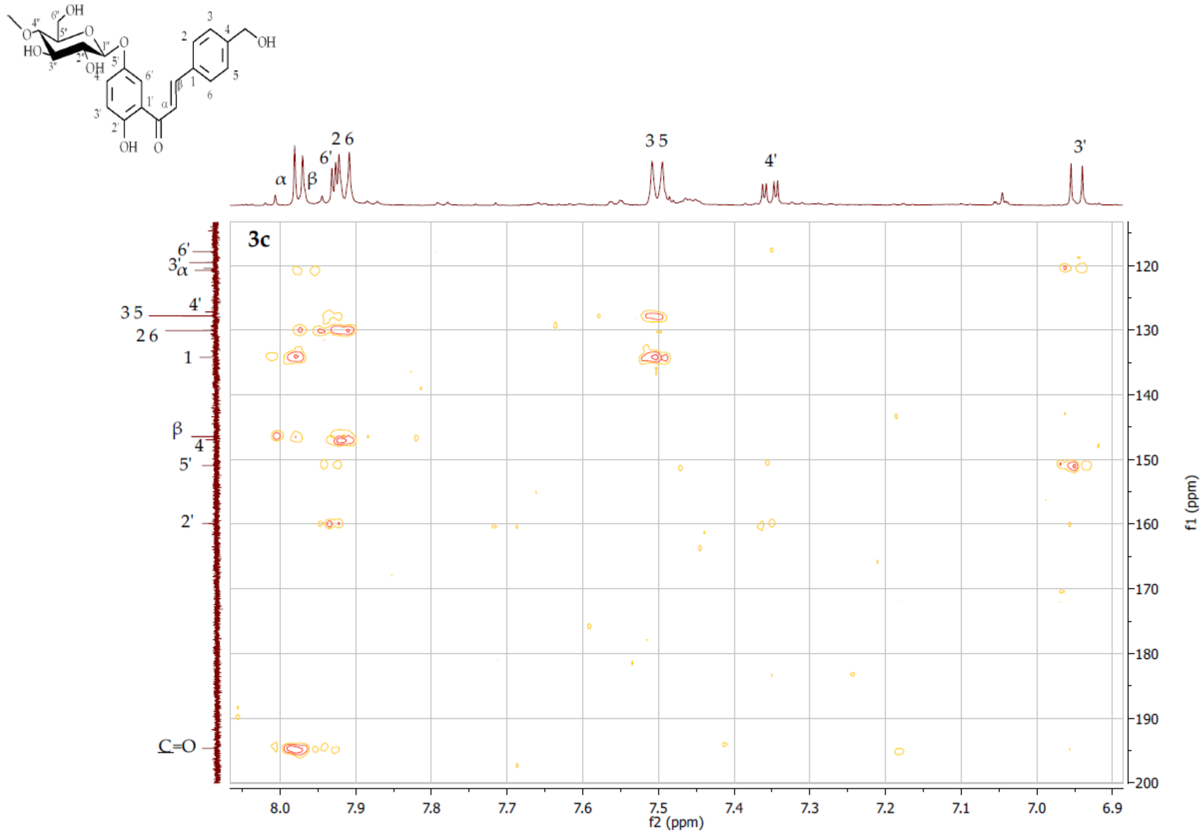

Figure S85. HMBC contour map – ^{1}H x ^{13}C expansion of 2′-hydroxy-4-hydroxymethylchalcone 5′-*O*-*β*-D-(4″-*O*-methyl)-glucopyranoside (**3c**)

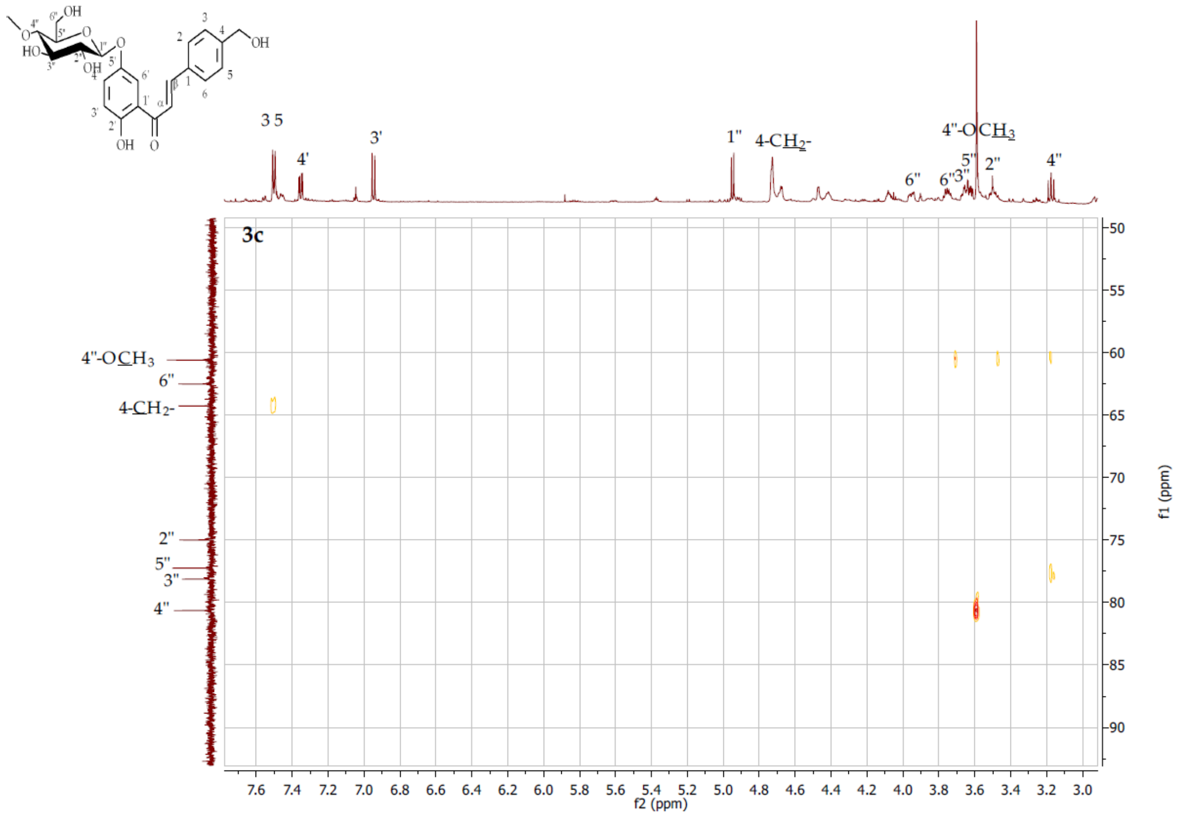

Figure S86. HMBC contour map – ^{1}H x ^{13}C expansion of 2′-hydroxy-4-hydroxymethylchalcone 5′-*O*-*β*-D-(4″-*O*-methyl)-glucopyranoside (**3c**)

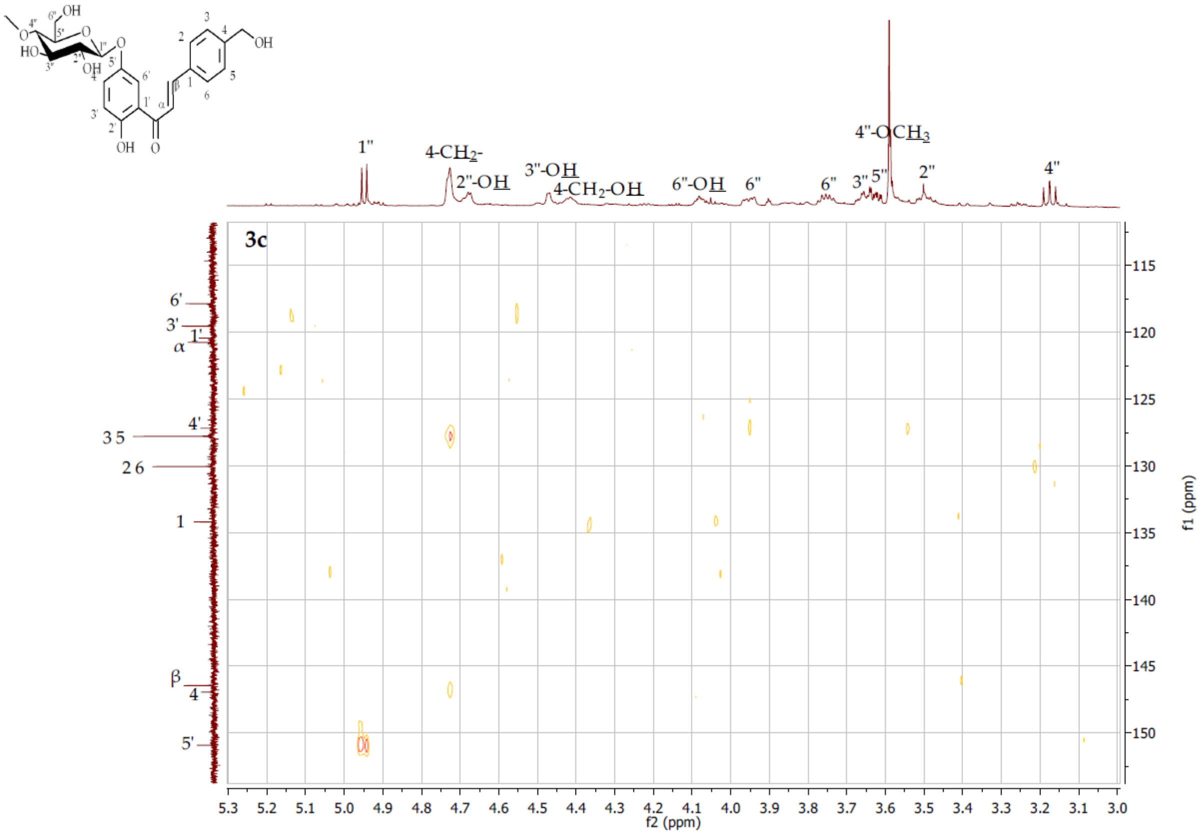

Figure S87. HMBC contour map – ^1H x ^{13}C expansion of 2′-hydroxy-4-hydroxymethylchalcone 5′-*O*-*β*-D-(4″-*O*-methyl)-glucopyranoside (**3c**)

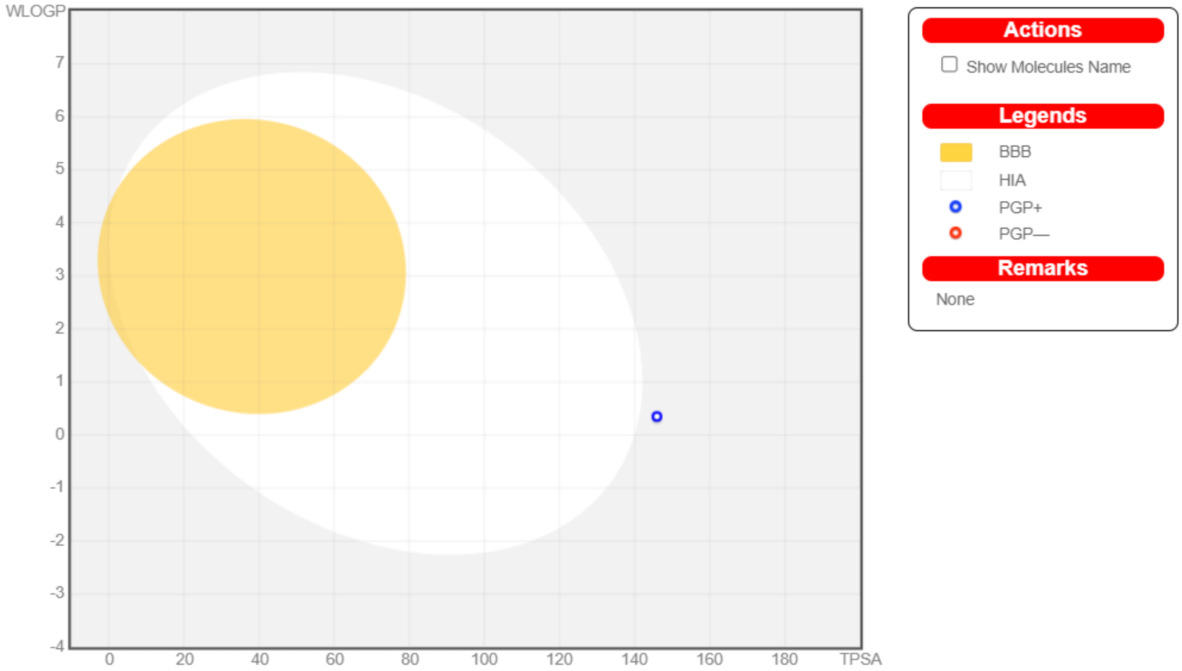

Molecule 1

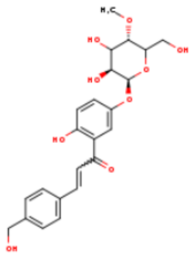

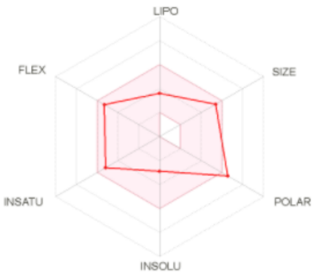

SMILES OCC1O[C@@H](Oc2ccc(c(c2)C(=O)/C=C/c2ccc(cc2)CO)O)[C@H](C([C@@H]1OC)O)O

Physicochemical Properties	
Formula	C23H26O9
Molecular weight	446.45 g/mol
Num. heavy atoms	32
Num. arom. heavy atoms	12
Fraction Csp3	0.35
Num. rotatable bonds	8
Num. H-bond acceptors	9
Num. H-bond donors	5
Molar Refractivity	113.27
TPSA	145.91 Å²

Lipophilicity	
Log $P_{o/w}$ (iLOGP)	2.71
Log $P_{o/w}$ (XLOGP3)	0.83
Log $P_{o/w}$ (WLOGP)	0.35
Log $P_{o/w}$ (MLOGP)	-0.83
Log $P_{o/w}$ (SILICOS-IT)	1.37
Consensus Log $P_{o/w}$	0.89

Water Solubility	
Log *S* (ESOL)	-2.88
Solubility	5.88e-01 mg/ml ; 1.32e-03 mol/l
Class	Soluble
Log *S* (Ali)	-3.48
Solubility	1.49e-01 mg/ml ; 3.34e-04 mol/l
Class	Soluble
Log *S* (SILICOS-IT)	-2.61
Solubility	1.09e+00 mg/ml ; 2.45e-03 mol/l
Class	Soluble

Pharmacokinetics	
GI absorption	Low
BBB permeant	No
P-gp substrate	Yes
CYP1A2 inhibitor	No
CYP2C19 inhibitor	No
CYP2C9 inhibitor	No
CYP2D6 inhibitor	No
CYP3A4 inhibitor	No
Log K_p (skin permeation)	-8.43 cm/s

Druglikeness	
Lipinski	Yes; 0 violation
Ghose	Yes
Veber	No; 1 violation: TPSA>140
Egan	No; 1 violation: TPSA>131.6
Muegge	Yes
Bioavailability Score	0.55

Medicinal Chemistry	
PAINS	0 alert
Brenk	1 alert: michael_acceptor_1
Leadlikeness	No; 2 violations: MW>350, Rotors>7
Synthetic accessibility	5.15

Figure S88. 2′-Hydroxy-4-hydroxymethylchalcone 5′-*O*-*β*-D-(4″-*O*-methyl)-glucopyranoside (**3c**) physicochemical and ADME parameters prediction using the SwissADME modelling

Pa	Pi	Activity
0,985	0,001	Monophenol monooxygenase inhibitor
0,943	0,003	Caspase 3 stimulant
0,936	0,005	CDP-glycerol glycerophosphotransferase inhibitor
0,930	0,002	Antiprotozoal (Leishmania)
0,921	0,006	Membrane integrity agonist
0,915	0,002	Chemopreventive
0,905	0,002	Hepatoprotectant
0,897	0,002	Free radical scavenger
0,875	0,003	Anticarcinogenic
0,875	0,006	Beta-adrenergic receptor kinase inhibitor

Figure S89. 2′-Hydroxy-4-hydroxymethylchalcone 5′-*O*-*β*-D-(4″-*O*-methyl)-glucopyranoside (**3c**) biological activity prediction using the Way2Drug Pass online modelling

Name	Confidence	ChEMBL ID
Clostridium ramosum	0.6112	CHEMBL614971
RESISTANT Acinetobacter pittii	0.5770	CHEMBL3140321
Actinomyces meyeri	0.5592	CHEMBL612289
Mycobacterium mageritense	0.5508	CHEMBL612959
RESISTANT Mycobacterium ulcerans	0.5466	CHEMBL612965
Clostridium cadaveris	0.5462	CHEMBL614970
Staphylococcus lugdunensis	0.4424	CHEMBL613303
RESISTANT Staphylococcus aureus subsp. aureus RN4220	0.4348	CHEMBL2366906
Nocardia transvalensis	0.4267	CHEMBL613234
Lactobacillus plantarum	0.4205	CHEMBL614973
Streptococcus sanguinis	0.4119	CHEMBL612314
Clostridium sordellii	0.3830	CHEMBL613072
Pseudomonas fluorescens	0.3632	CHEMBL612500
Yersinia pestis	0.3527	CHEMBL614597
Nocardia otitidiscaviarum	0.3399	CHEMBL613233

Figure S90. 2′-Hydroxy-4-hydroxymethylchalcone 5′-*O*-*β*-D-(4″-*O*-methyl)-glucopyranoside (**3c**) antibacterial activity prediction using the Way2Drug AntiBac-Pred modelling

Name	Confidence	ChEMBL ID
Rhizopus oryzae	0.3855	CHEMBL612306
Trichophyton mentagrophytes	0.3616	CHEMBL613162
Absidia corymbifera	0.3575	CHEMBL612369
Aspergillus niger	0.1685	CHEMBL358
Epidermophyton floccosum	0.1418	CHEMBL612386
Mucor	0.0948	CHEMBL612521
Yarrowia lipolytica	0.0910	CHEMBL612844
Penicillium marneffei	0.0611	CHEMBL612994
Trichosporon asahii	0.0441	CHEMBL613164
Saccharomyces cerevisiae	0.0263	CHEMBL361
Candida rugosa	0.0211	CHEMBL612869
Galactomyces geotrichum	0.0175	CHEMBL613775

Figure S91. 2′-Hydroxy-4-hydroxymethylchalcone 5′-*O*-*β*-D-(4″-*O*-methyl)-glucopyranoside (**3c**) antifungal activity prediction using the Way2Drug AntiFun-Pred modelling

Virus	Protein target	Confidence
Severe acute respiratory syndrome coronavirus 2	Replicase polyprotein 1ab	0.7810
Human immunodeficiency virus 2	Human immunodeficiency virus type 2 integrase	0.2266
Varicella-zoster virus (strain Dumas) (HHV-3) (Human herpesvirus 3)	Thymidine kinase	0.0086
Influenza A virus	Neuraminidase	0.0031

Figure S92. 2′-Hydroxy-4-hydroxymethylchalcone 5′-*O*-*β*-D-(4″-*O*-methyl)-glucopyranoside (**3c**) antiviral activity prediction using the Way2Drug AntiVir-Pred modelling

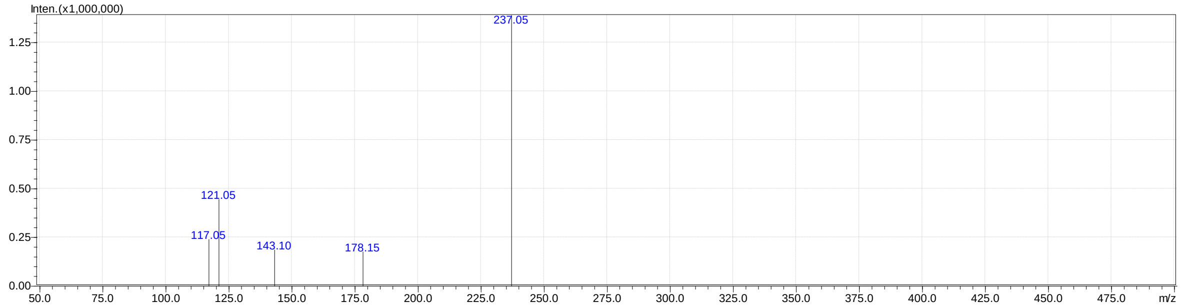

Figure S93. MS analysis of 4′-methyflavone (**5**)

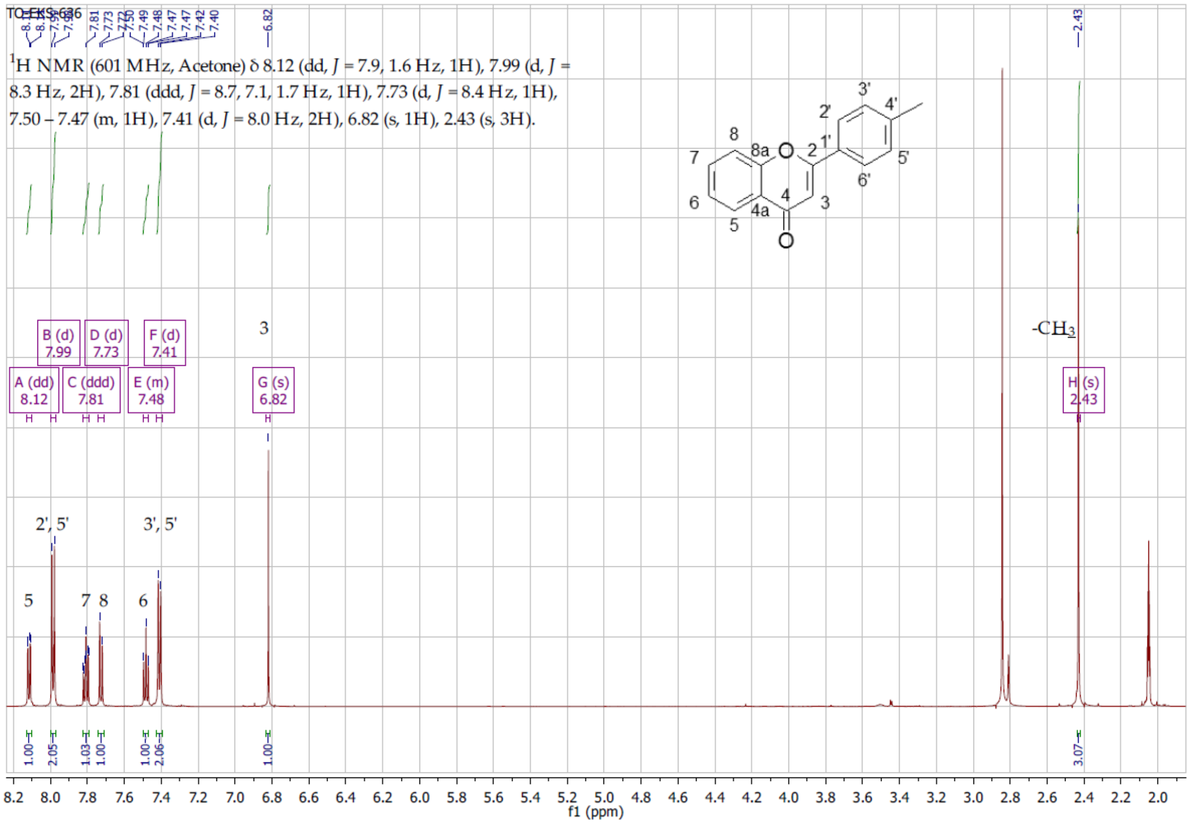

Figure S94. ^{1}H NMR spectrum (*δ,* acetone-d6, 600 MHz) of 4′-methyflavone (**5**)

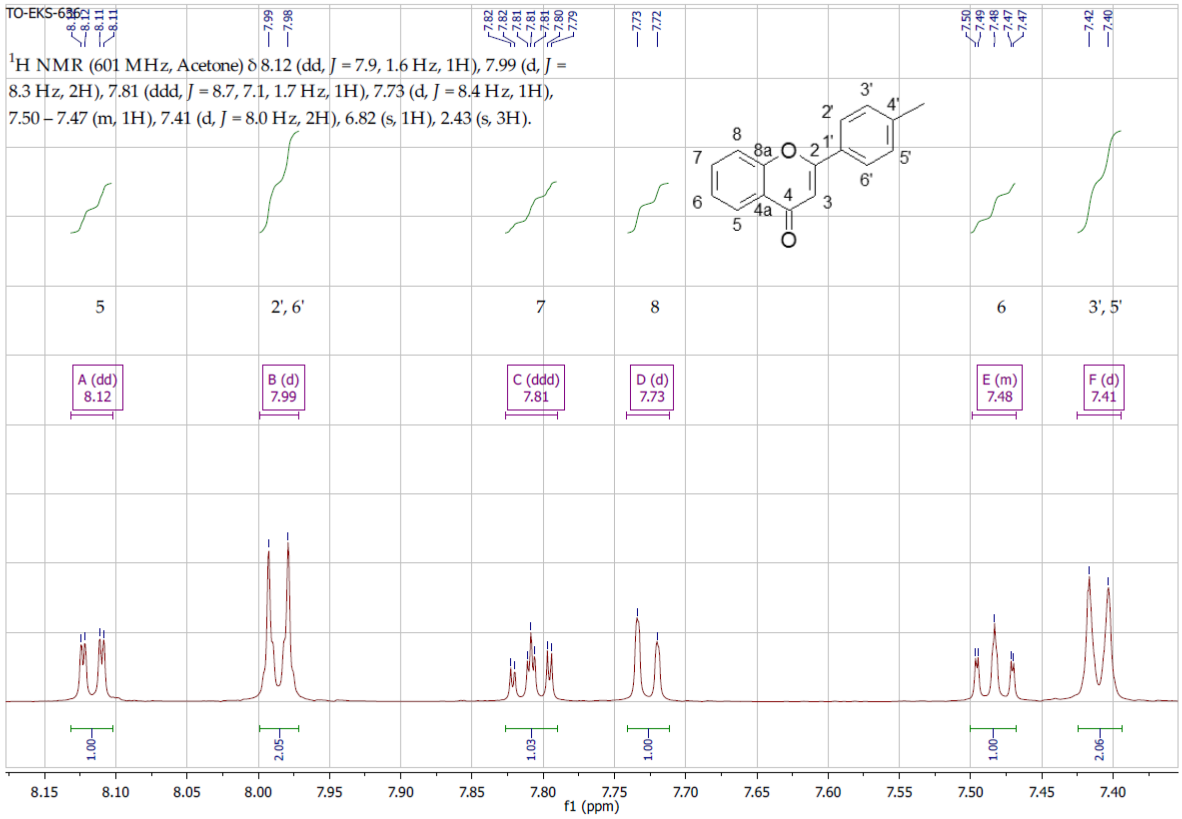

Figure S95. ^{1}H NMR spectrum expansion (*δ*, acetone-d6, 600 MHz) of 4′-methyflavone (**5**)

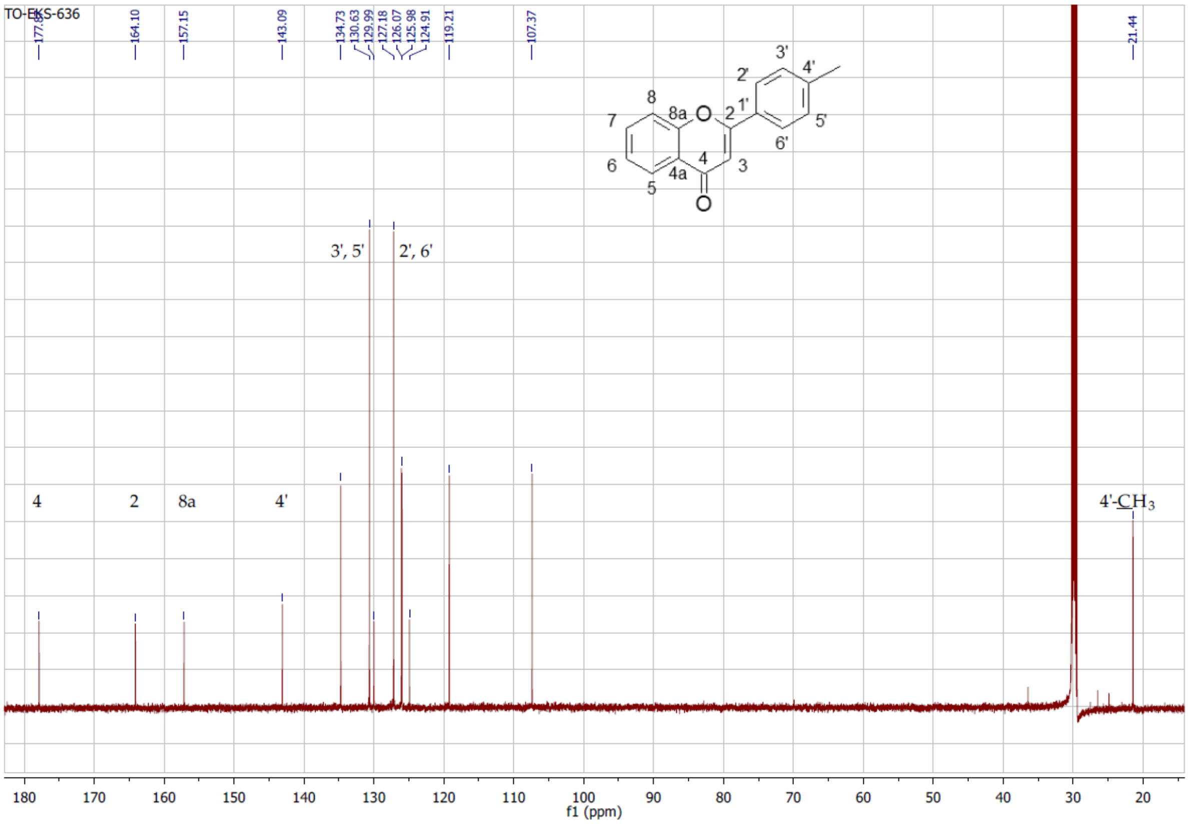

Figure S96. ^{13}C NMR spectrum (*δ*, acetone-d6, 151 MHz) of 4′-methyflavone (**5**)

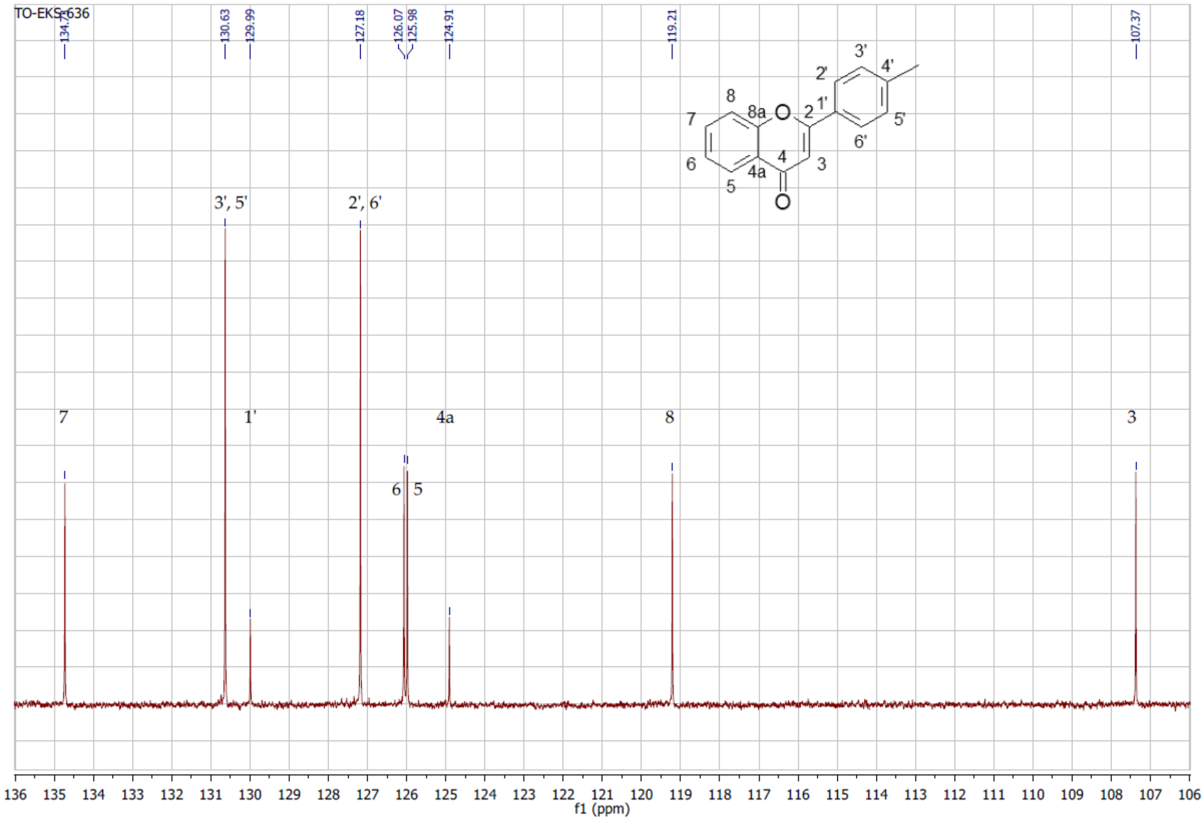

Figure S97. ^{13}C NMR spectrum expansion (δ, acetone-d6, 151 MHz) of 4′-methyflavone (**5**)

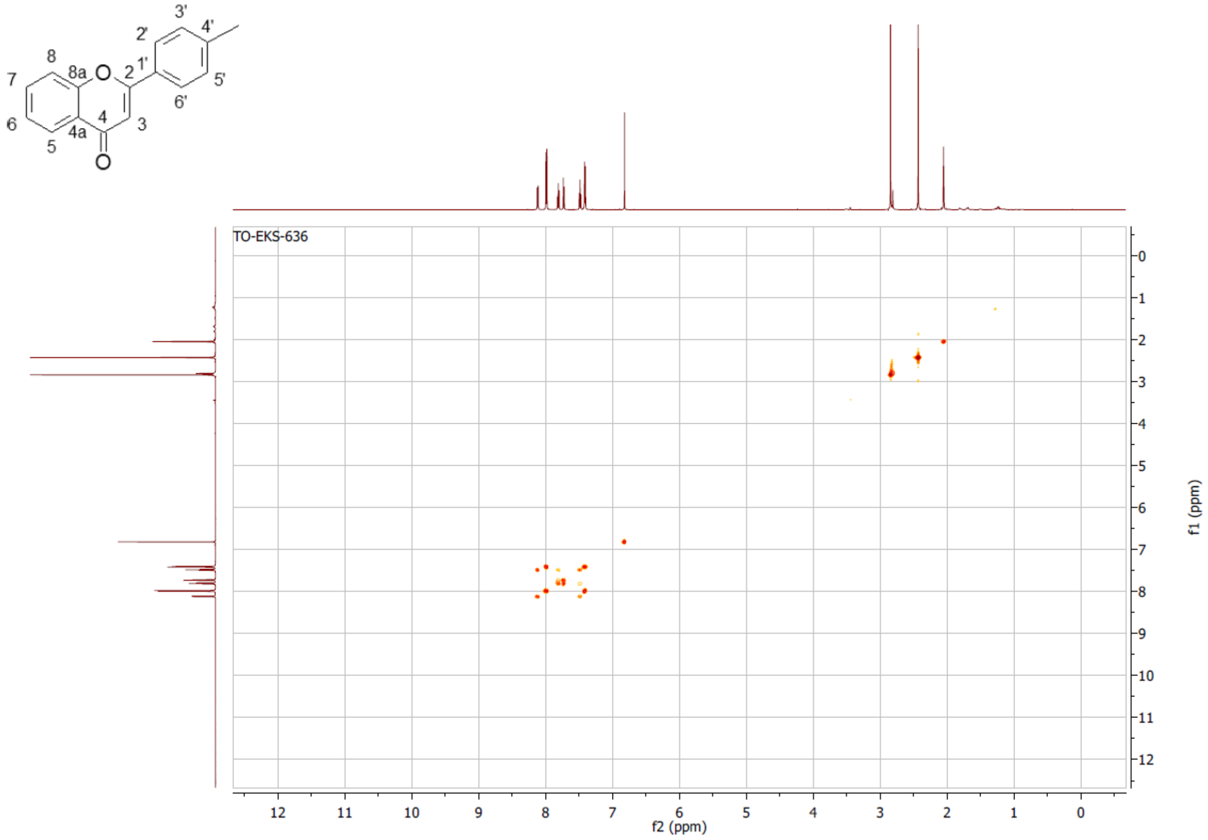

Figure S98. COSY contour map – ^{1}H x ^{1}H of 4′-methyflavone (**5**)

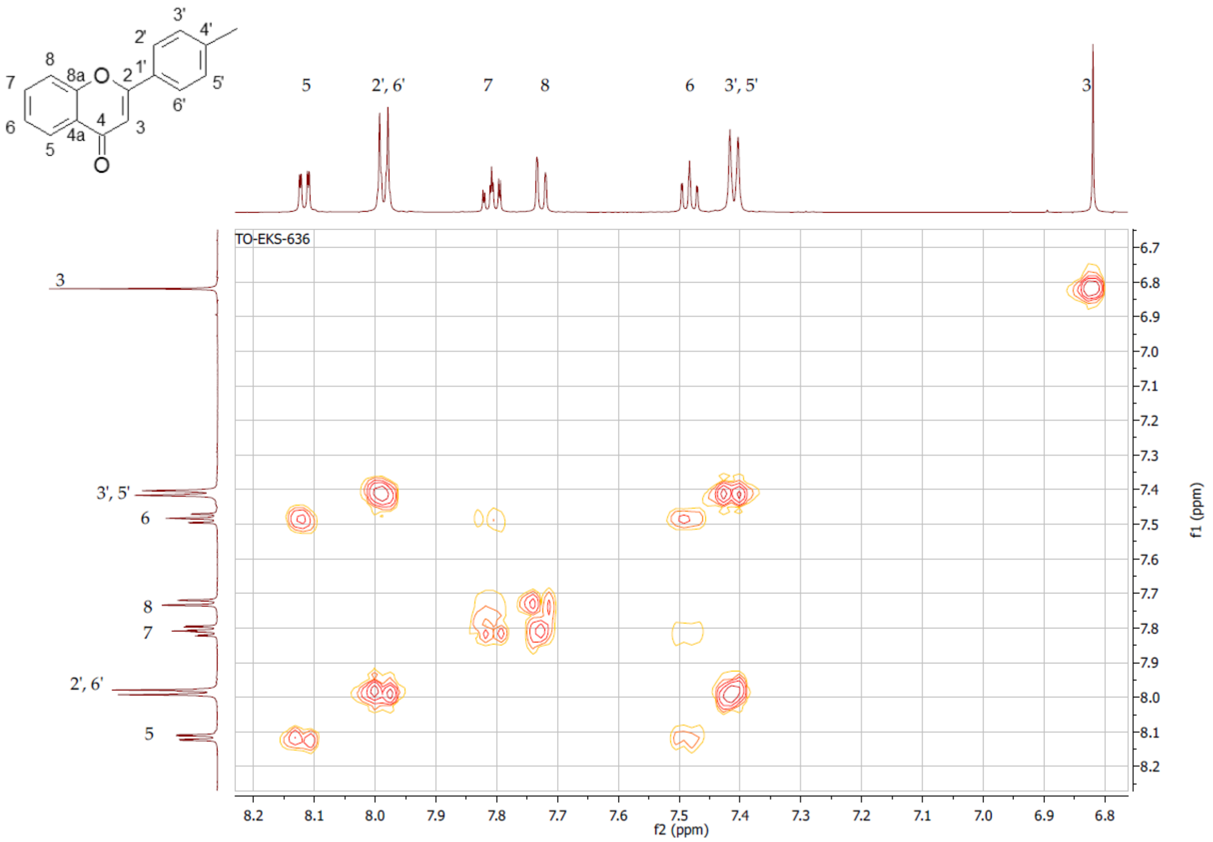

Figure S99. COSY contour map – ^1H x ^1H expansion of 4′-methyflavone (**5**)

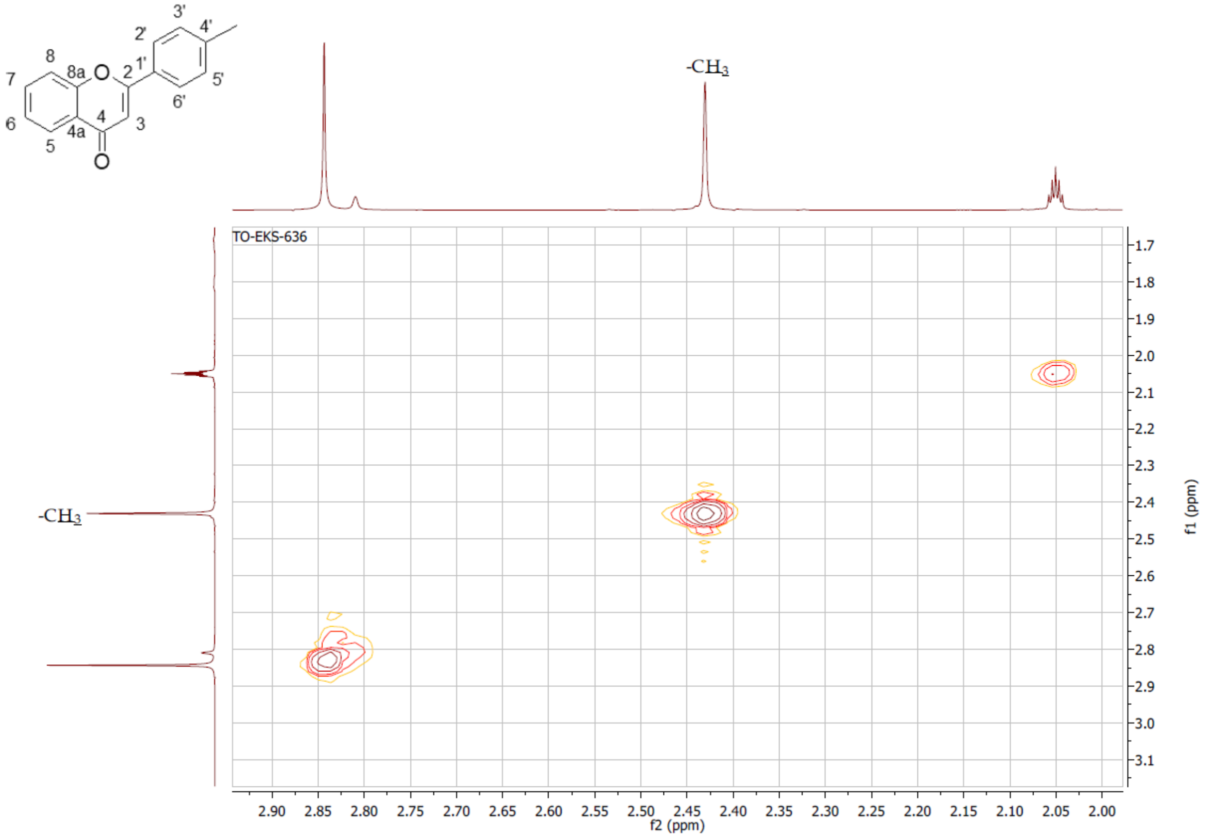

Figure S100. COSY contour map – ^1H x ^1H expansion of 4′-methyflavone (**5**)

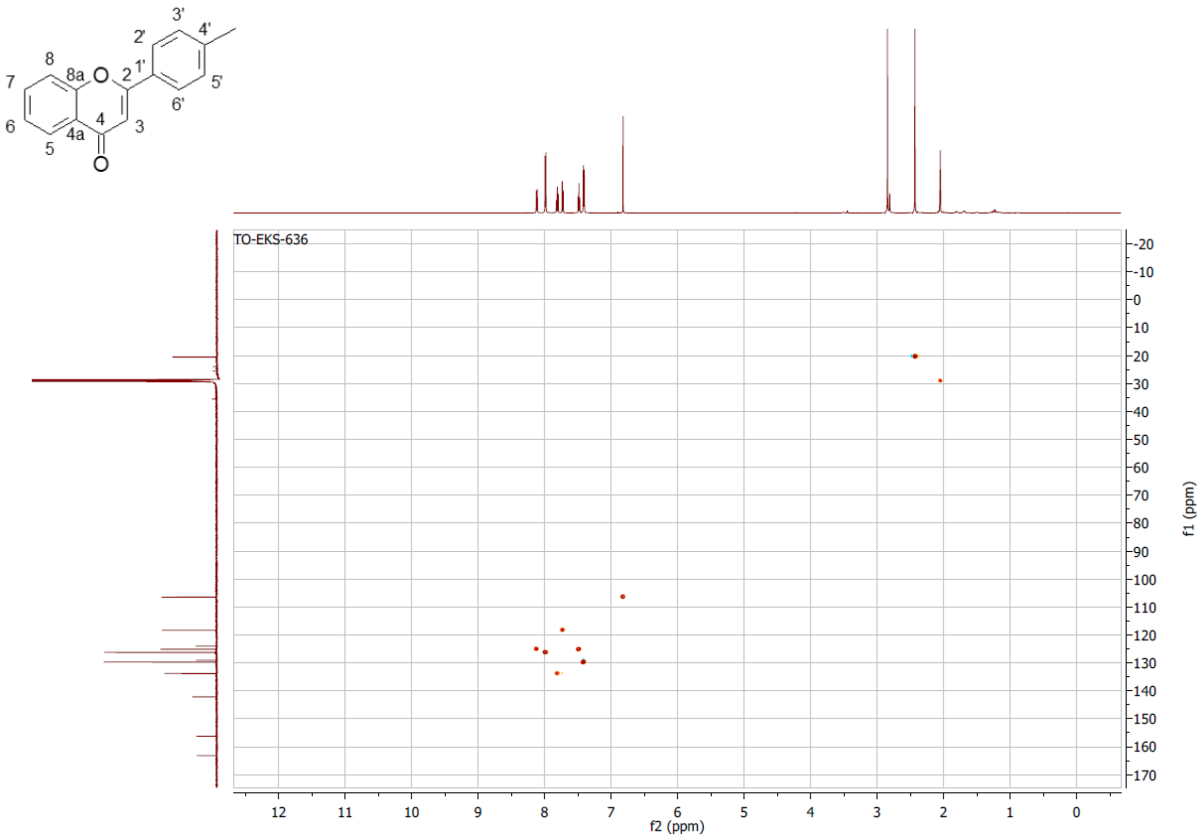

Figure S101. HSQC contour map – ^{1}H x ^{13}C of 4′-methyflavone (**5**)

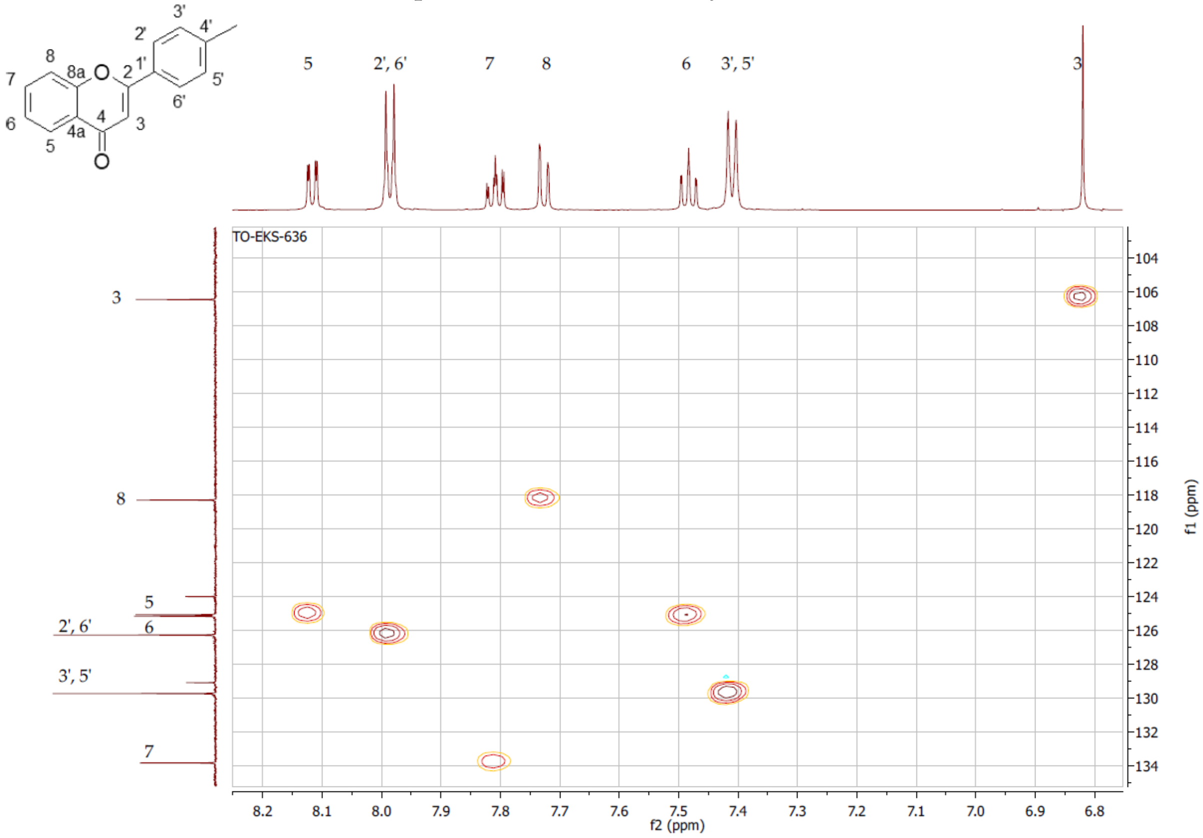

Figure S102. HSQC contour map – ^{1}H x ^{13}C expansion of 4′-methyflavone (**5**)

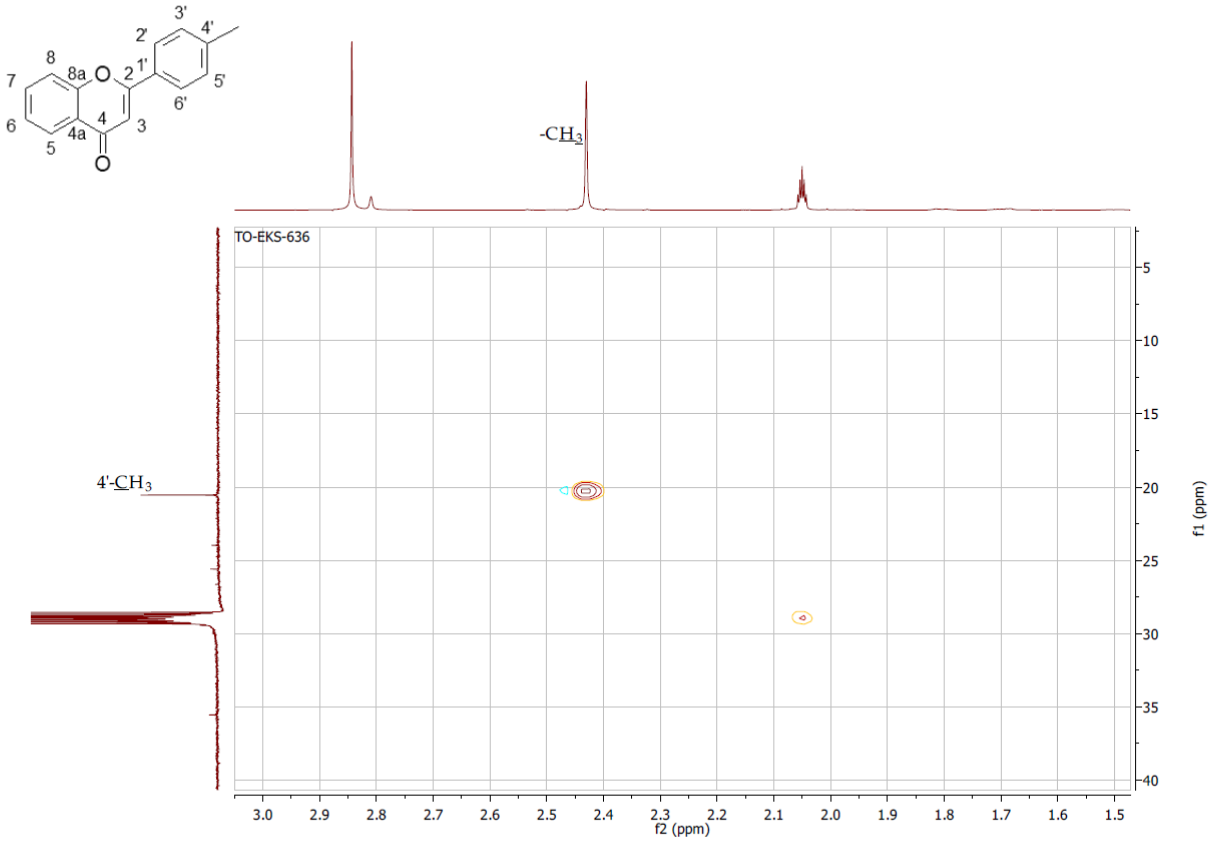

Figure S103. HSQC contour map – ^{1}H x ^{13}C expansion of 4′-methyflavone (**5**)

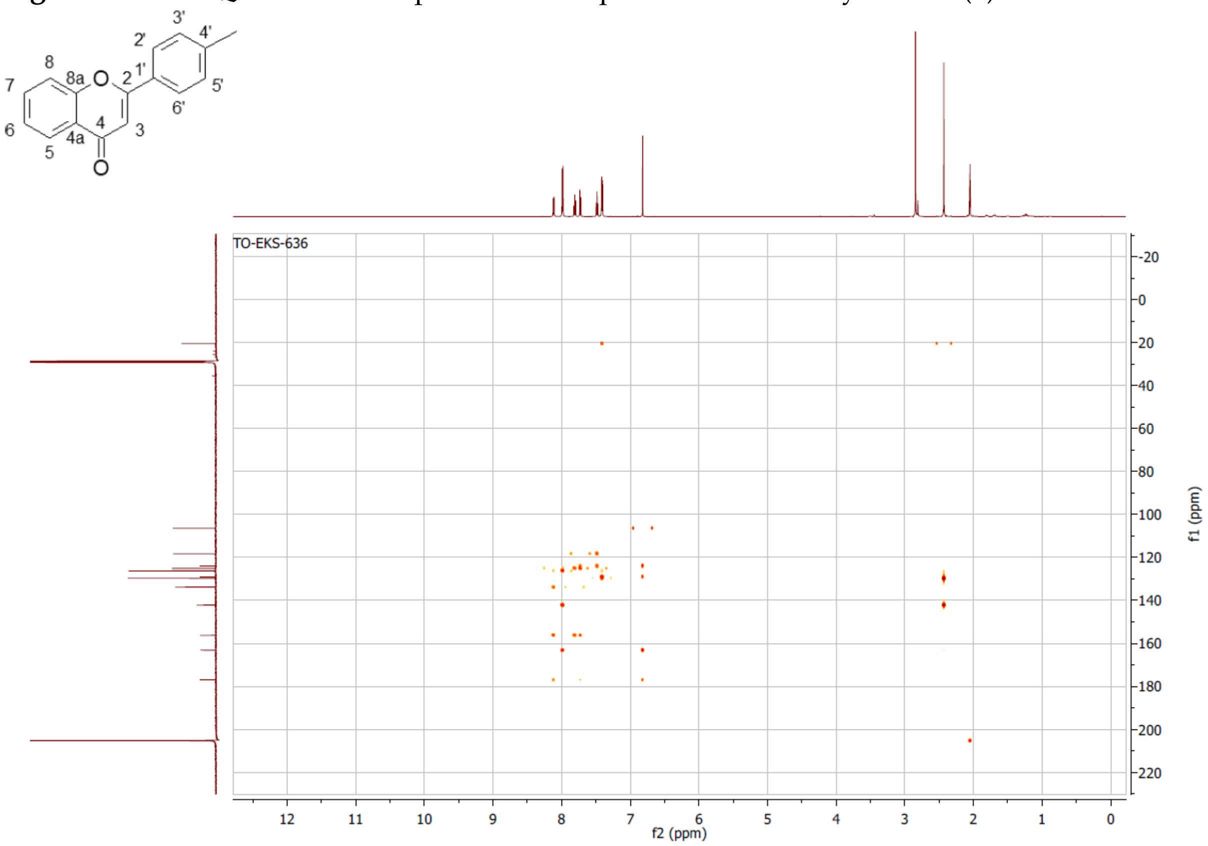

Figure S104. HMBC contour map – ^{1}H x ^{13}C of 4′-methyflavone (**5**)

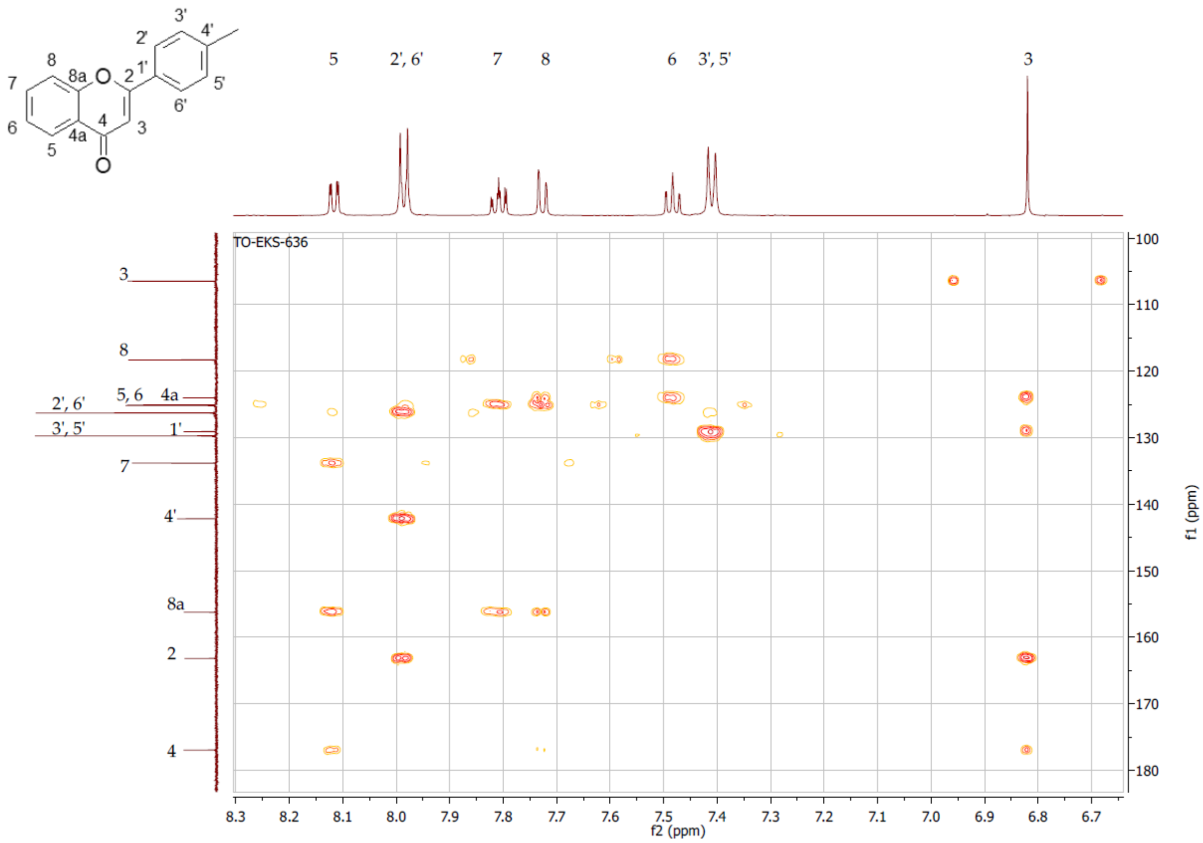

Figure S105. HMBC contour map – ^{1}H x ^{13}C expansion of 4′-methyflavone (**5**)

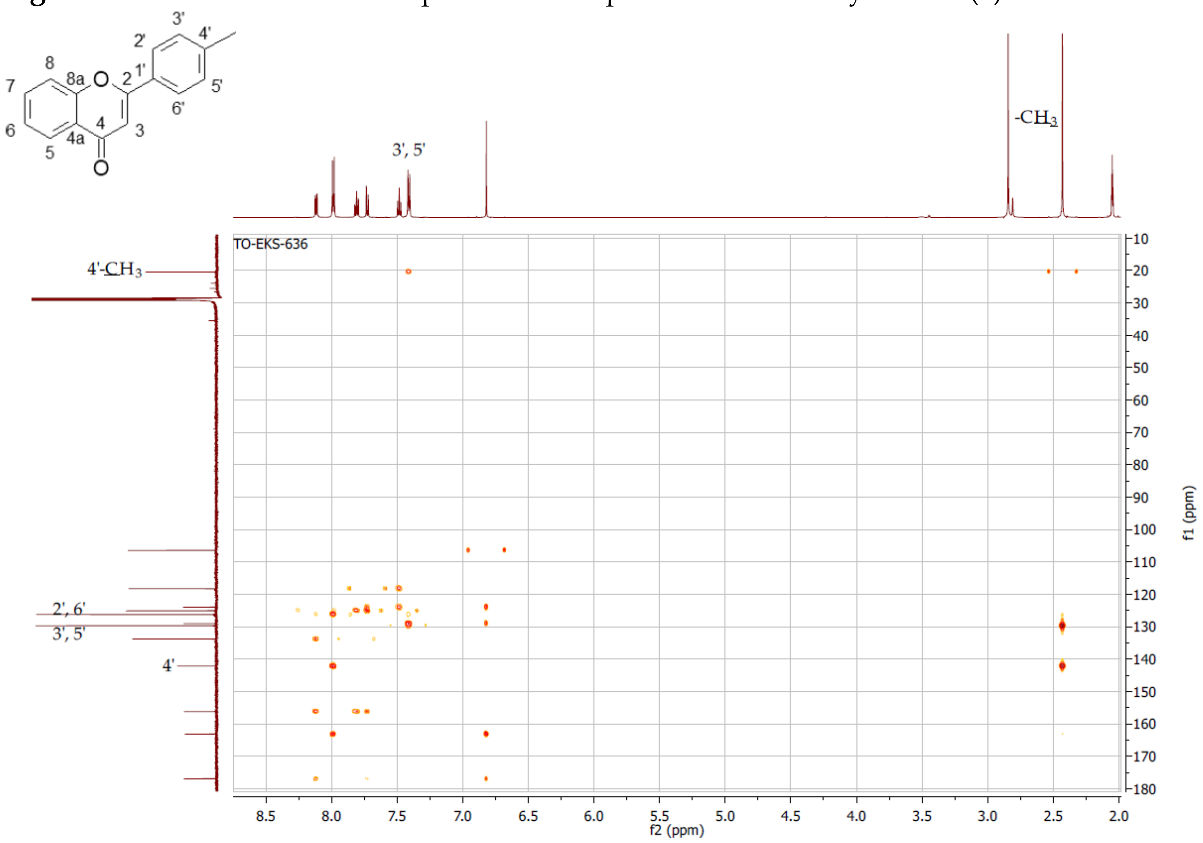

Figure S106. HMBC contour map – ^{1}H x ^{13}C expansion of 4′-methyflavone (**5**)

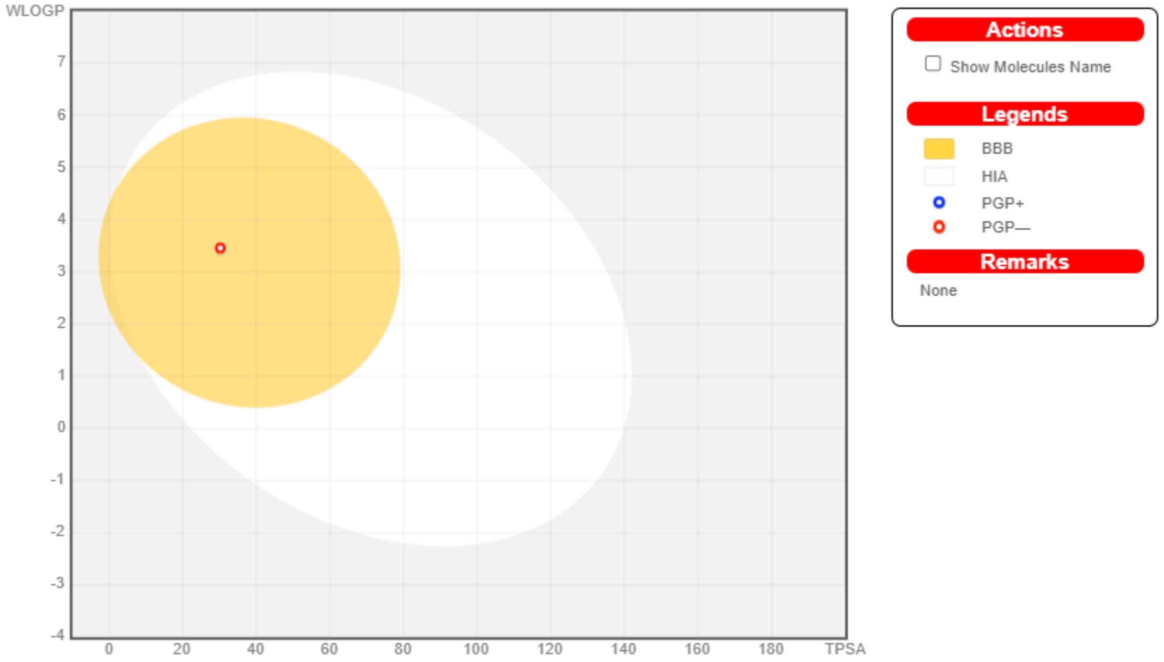

Molecule 1

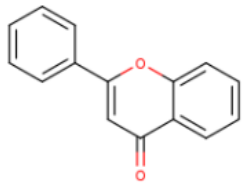

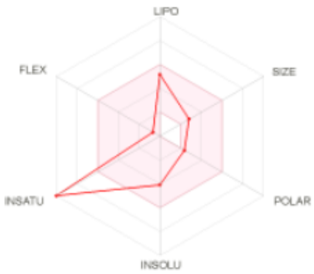

SMILES O=c1cc(oc2c1cccc2)c1ccccc1

Physicochemical Properties	
Formula	C15H10O2
Molecular weight	222.24 g/mol
Num. heavy atoms	17
Num. arom. heavy atoms	16
Fraction Csp3	0.00
Num. rotatable bonds	1
Num. H-bond acceptors	2
Num. H-bond donors	0
Molar Refractivity	67.92
TPSA	30.21 Å²

Lipophilicity	
Log $P_{o/w}$ (iLOGP)	2.55
Log $P_{o/w}$ (XLOGP3)	3.56
Log $P_{o/w}$ (WLOGP)	3.46
Log $P_{o/w}$ (MLOGP)	2.27
Log $P_{o/w}$ (SILICOS-IT)	4.04
Consensus Log $P_{o/w}$	3.18

Water Solubility	
Log *S* (ESOL)	-4.09
Solubility	1.80e-02 mg/ml ; 8.11e-05 mol/l
Class	Moderately soluble
Log *S* (Ali)	-3.88
Solubility	2.93e-02 mg/ml ; 1.32e-04 mol/l
Class	Soluble
Log *S* (SILICOS-IT)	-6.13
Solubility	1.63e-04 mg/ml ; 7.33e-07 mol/l
Class	Poorly soluble

Pharmacokinetics	
GI absorption	High
BBB permeant	Yes
P-gp substrate	No
CYP1A2 inhibitor	Yes
CYP2C19 inhibitor	Yes
CYP2C9 inhibitor	No
CYP2D6 inhibitor	No
CYP3A4 inhibitor	No
Log K_p (skin permeation)	-5.13 cm/s

Druglikeness	
Lipinski	Yes; 0 violation
Ghose	Yes
Veber	Yes
Egan	Yes
Muegge	Yes
Bioavailability Score	0.55

Medicinal Chemistry	
PAINS	0 alert
Brenk	0 alert
Leadlikeness	No; 2 violations: MW<250, XLOGP3>3.5
Synthetic accessibility	2.88

Figure S107. 4′-Methyflavone (**5**) physicochemical and ADME parameters prediction using the SwissADME modelling

Pa	Pi	Activity
0,952	0,001	4-Nitrophenol 2-monooxygenase inhibitor
0,952	0,003	HIF1A expression inhibitor
0,947	0,004	Membrane integrity agonist
0,943	0,002	27-Hydroxycholesterol 7alpha-monooxygenase inhibitor
0,938	0,002	Kinase inhibitor
0,933	0,002	Cholestanetriol 26-monooxygenase inhibitor
0,929	0,003	Anaphylatoxin receptor antagonist
0,914	0,003	Membrane permeability inhibitor
0,913	0,004	Chlordecone reductase inhibitor
0,913	0,009	CYP2C12 substrate

Figure S108. 4′-Methyflavone (**5**) physicochemical biological activity prediction using the Way2Drug Pass online modelling

Name	Confidence	ChEMBL ID
Yersinia pestis	0.5183	CHEMBL614597
Pseudomonas fluorescens	0.3605	CHEMBL612500
Streptococcus pneumoniae R6	0.2974	CHEMBL2366794
RESISTANT Burkholderia pseudomallei	0.2655	CHEMBL3140323
Salmonella enterica subsp. enterica	0.2594	CHEMBL613044
Bacillus subtilis subsp. subtilis str. 168	0.2582	CHEMBL613315
RESISTANT Staphylococcus aureus subsp. aureus MW2	0.2508	CHEMBL612531
Bacillus thuringiensis	0.2000	CHEMBL614959
Mycobacterium	0.1962	CHEMBL614981
Mycobacterium aurum	0.1864	CHEMBL612952
Bacillus subtilis	0.1846	CHEMBL359
RESISTANT Bacillus subtilis	0.1758	CHEMBL359
RESISTANT Helicobacter pylori	0.1413	CHEMBL612600
Bacillus anthracis	0.1376	CHEMBL613904
Nocardia nova	0.1339	CHEMBL612977

Figure S109. 4′-Methyflavone (**5**) antibacterial activity prediction using the Way2Drug AntiBac-Pred modelling

Name	Confidence	ChEMBL ID
Epidermophyton floccosum	0.3025	CHEMBL612386
Arthroderma benhamiae	0.0147	CHEMBL346

Figure S110. 4′-Methyflavone (**5**) antifungal activity prediction using the Way2Drug AntiFun-Pred modelling

Virus	Protein target	Confidence
Human immunodeficiency virus 2	Human immunodeficiency virus type 2 integrase	0.8508
Severe acute respiratory syndrome coronavirus 2	Replicase polyprotein 1ab	0.6456
Dengue virus type 2	Genome polyprotein	0.4865
Vaccinia virus (strain Western Reserve) (VACV) (Vaccinia virus (strainWR))	DNA polymerase	0.4125
SARS coronavirus	SARS coronavirus 3C-like proteinase	0.2286
Varicella-zoster virus (strain Dumas) (HHV-3) (Human herpesvirus 3)	DNA polymerase	0.2173
Herpes simplex virus (type 1 / strain 17)	Human herpesvirus 1 DNA polymerase	0.2173
Infectious bronchitis virus	3C-like protease	0.1764
SARS coronavirus	Replicase polyprotein 1ab	0.1489

Figure S111. 4′-Methyflavone (**5**) antiviral activity prediction using the Way2Drug AntiVir-Pred modelling

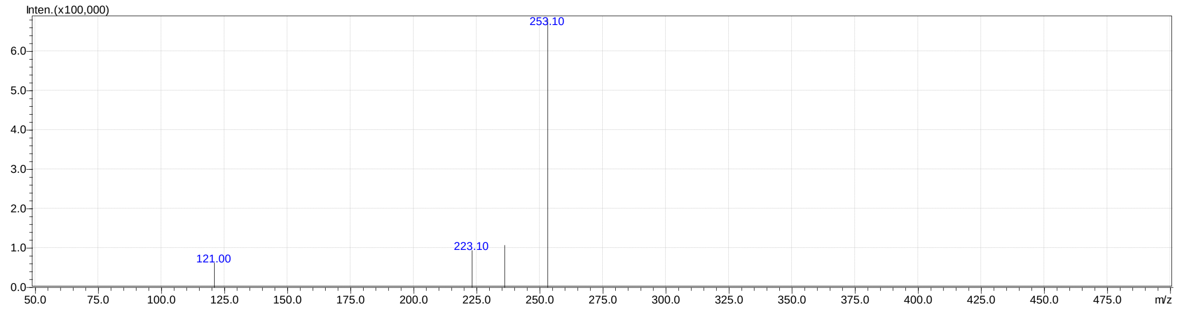

Figure S112. MS analysis 4′-hydroxymethylflavone (**5a**)

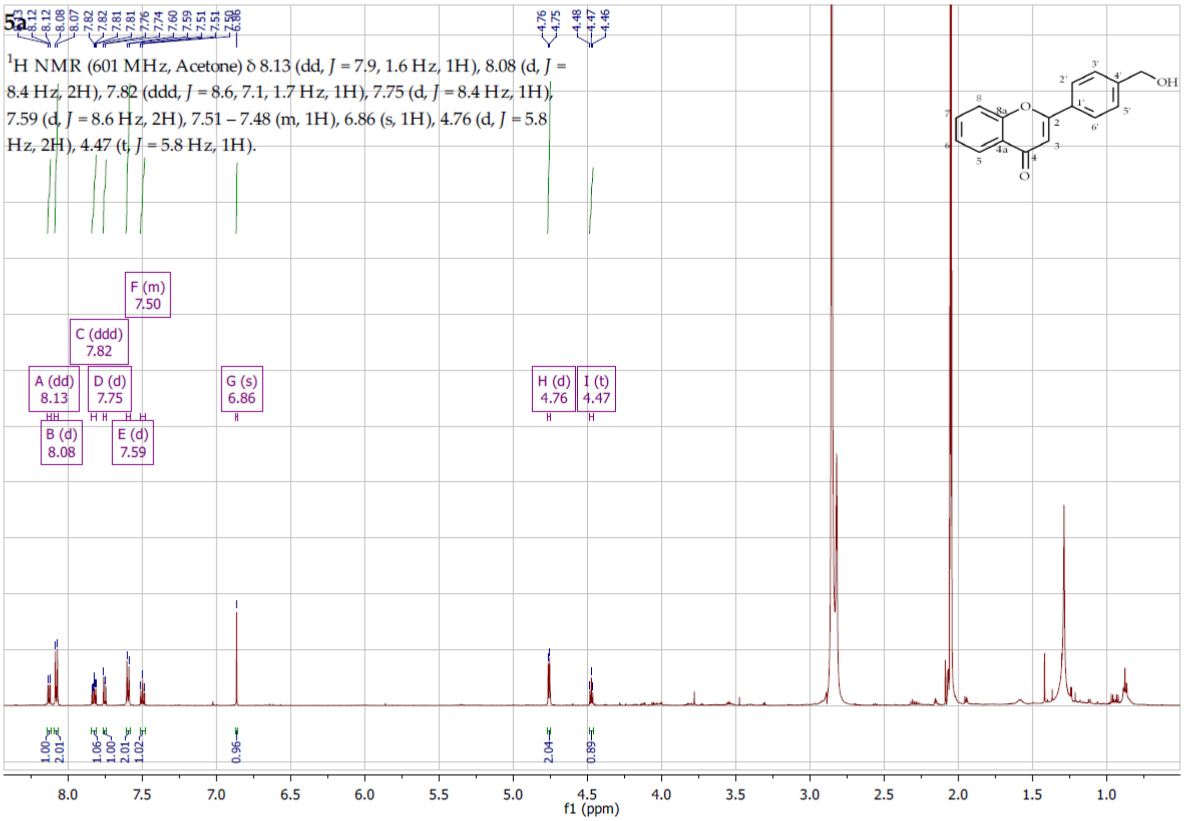

Figure S113. ^{1}H NMR spectrum (*δ*, acetone-d6, 600 MHz) of 4′-hydroxymethylflavone (**5a**)

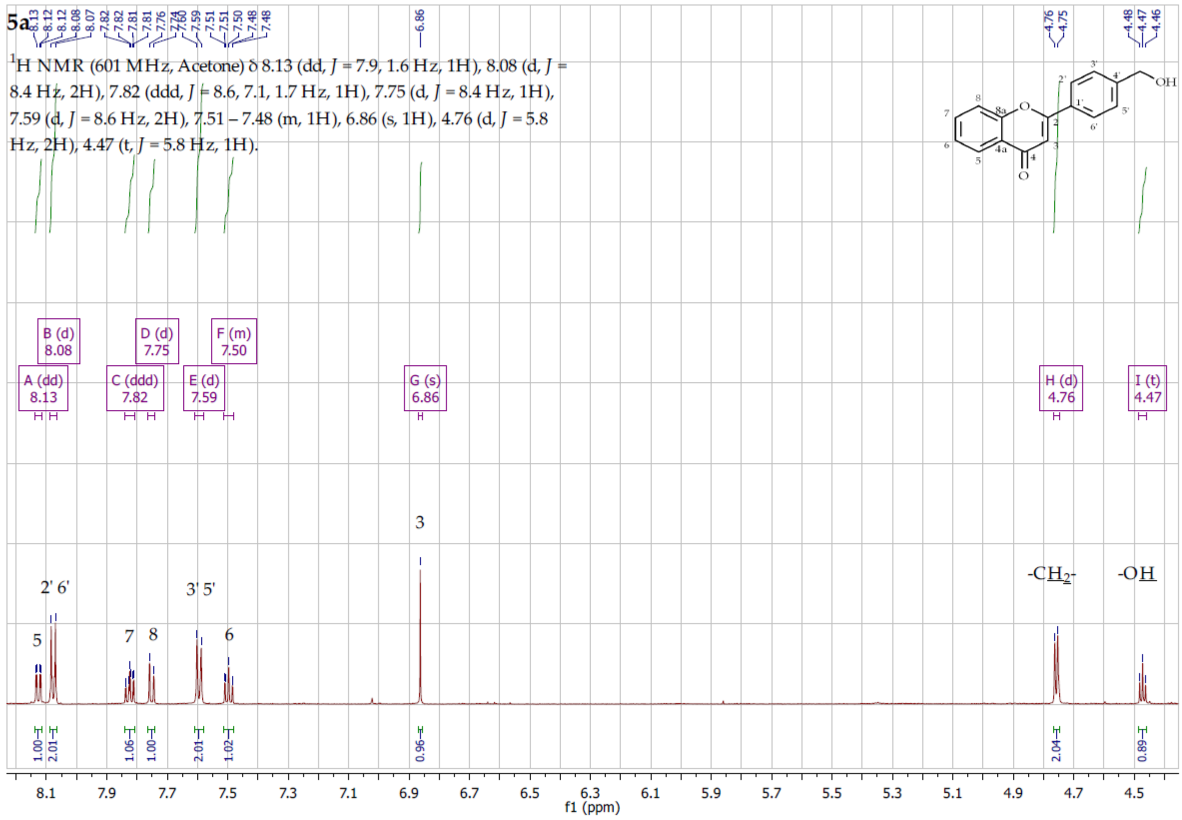

Figure S114. ^{1}H NMR spectrum expansion (δ, acetone-d6, 600 MHz) of 4′-hydroxymethylflavone (**5a**)

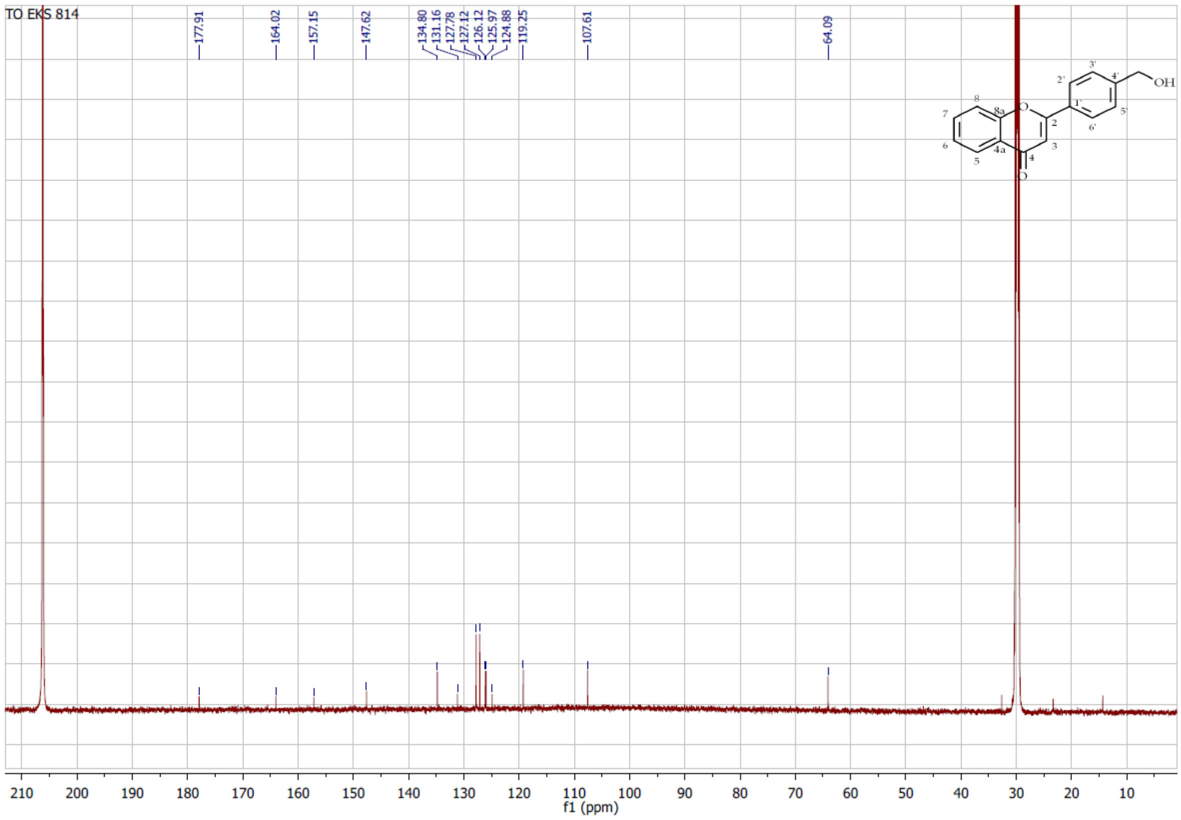

Figure S115. ^{13}C NMR spectrum expansion (δ, acetone-d6, 151 MHz) of 4′-hydroxymethylflavone (**5a**)

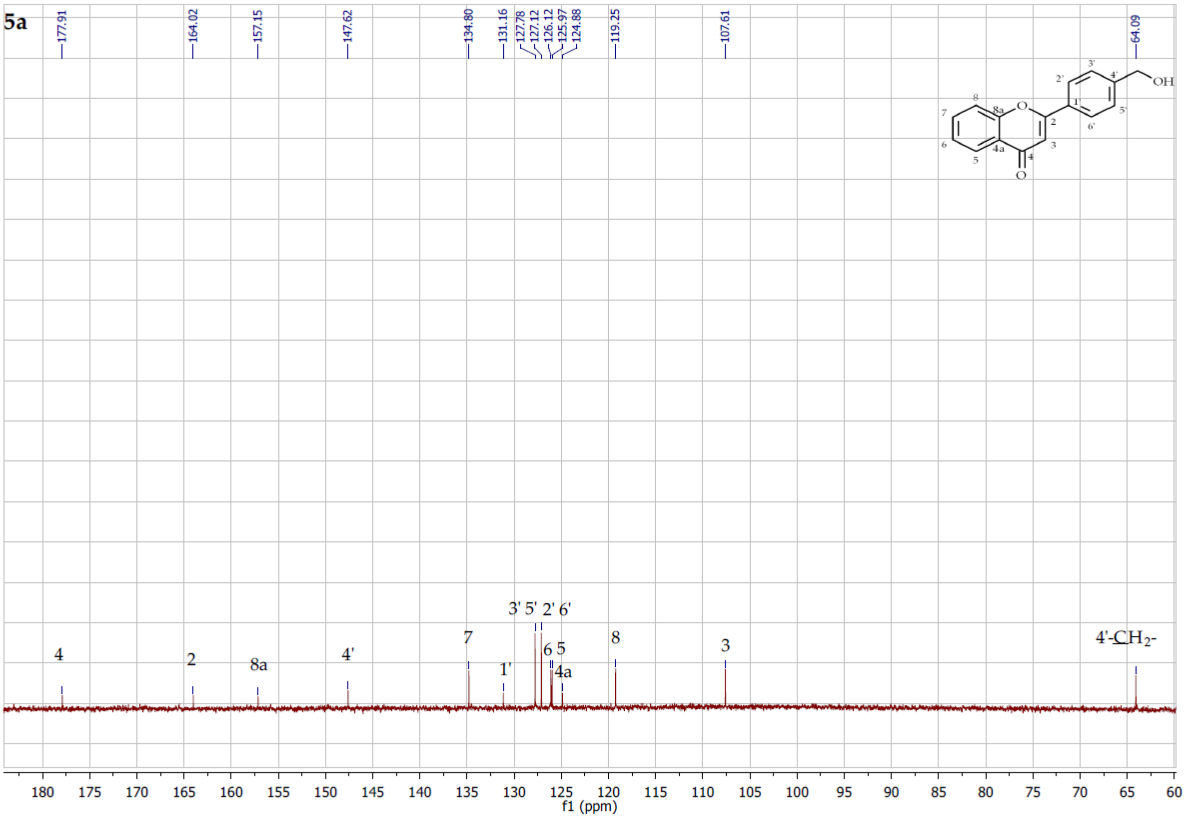

Figure S116. ^{13}C NMR spectrum expansion (δ, acetone-d6, 151 MHz) of 4'-hydroxymethylflavone (**5a**)

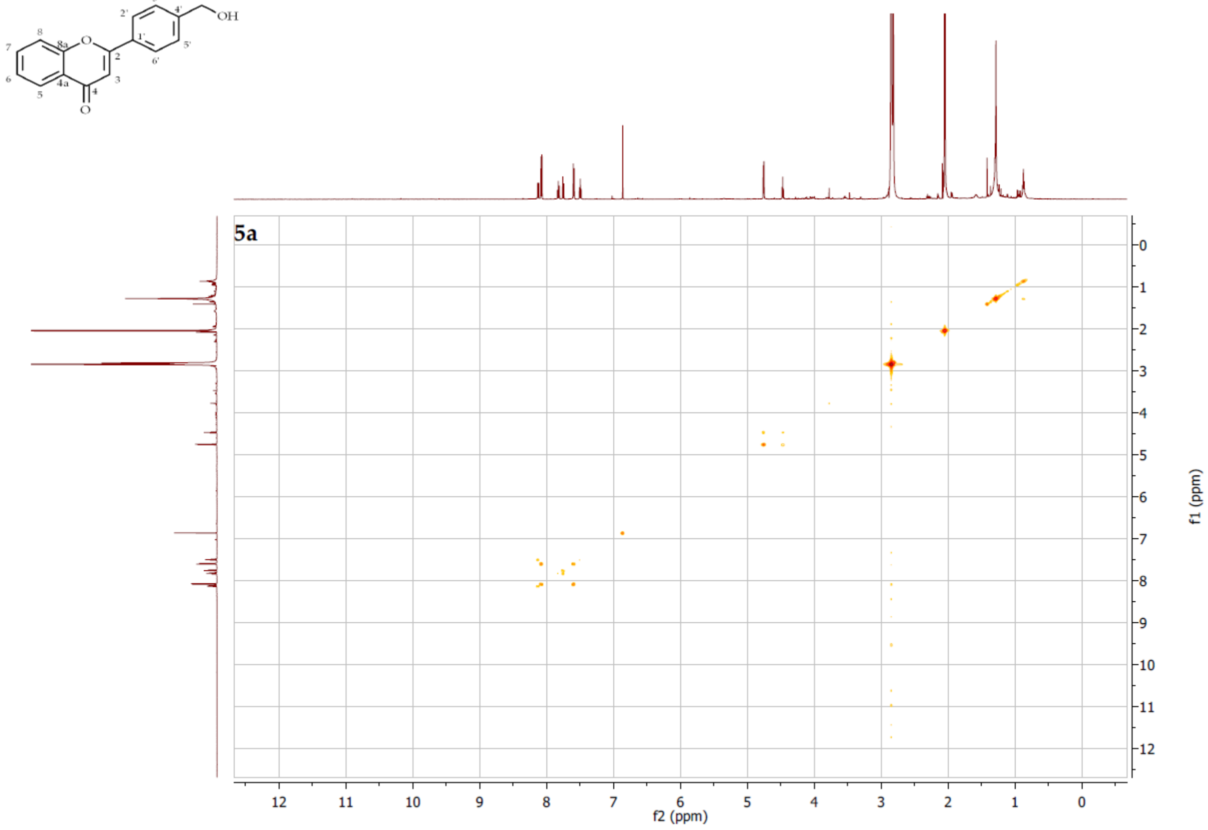

Figure S117. COSY contour map – ^{1}H x ^{1}H of 4'-hydroxymethylflavone (**5a**)

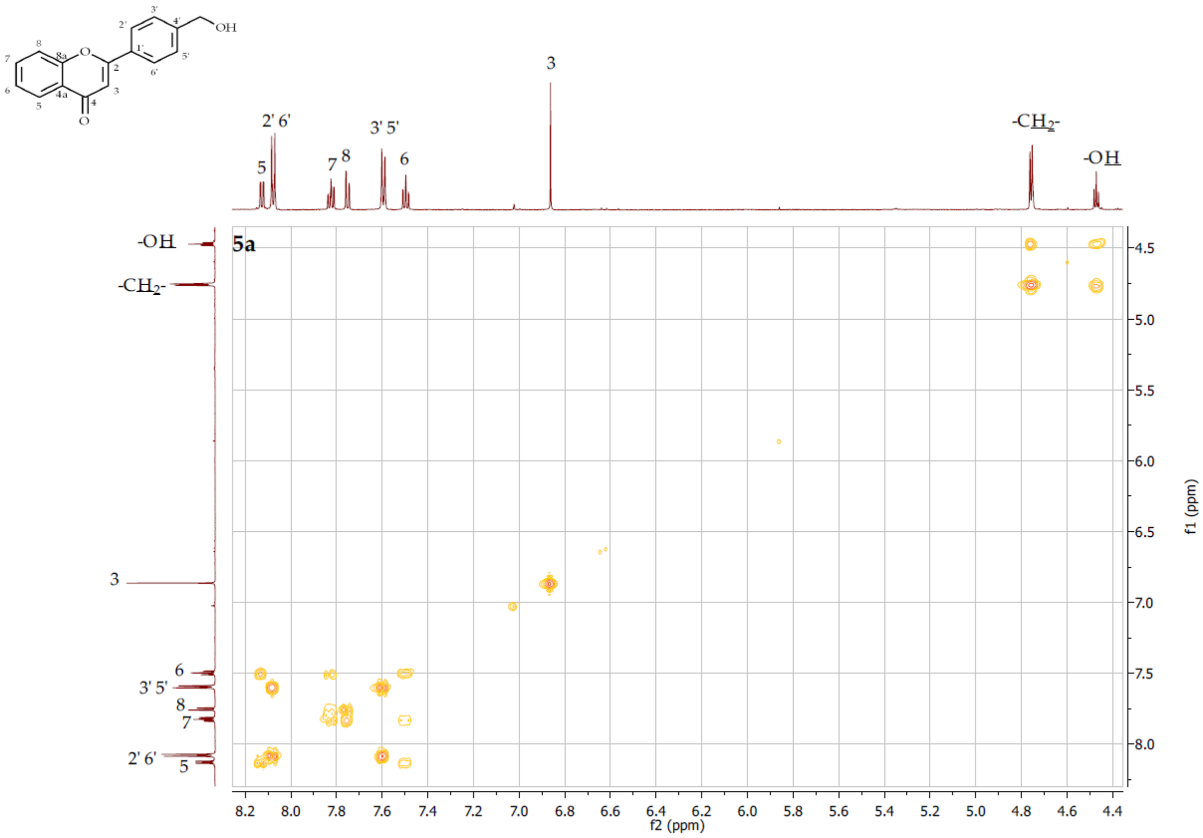

Figure S118. COSY contour map – ^{1}H x ^{1}H expansion of 4′-hydroxymethylflavone (**5a**)

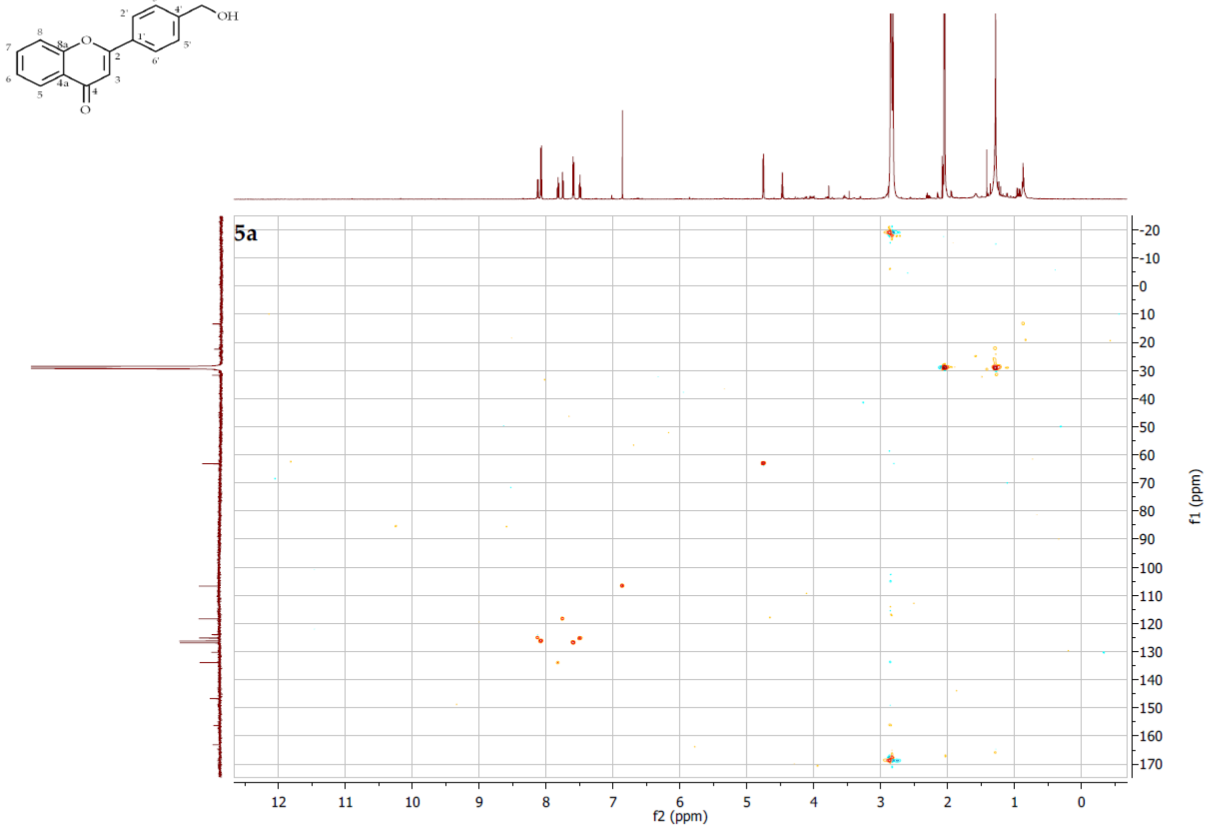

Figure S119. HSQC contour map – ^{1}H x ^{13}C of 4′-hydroxymethylflavone (**5a**)

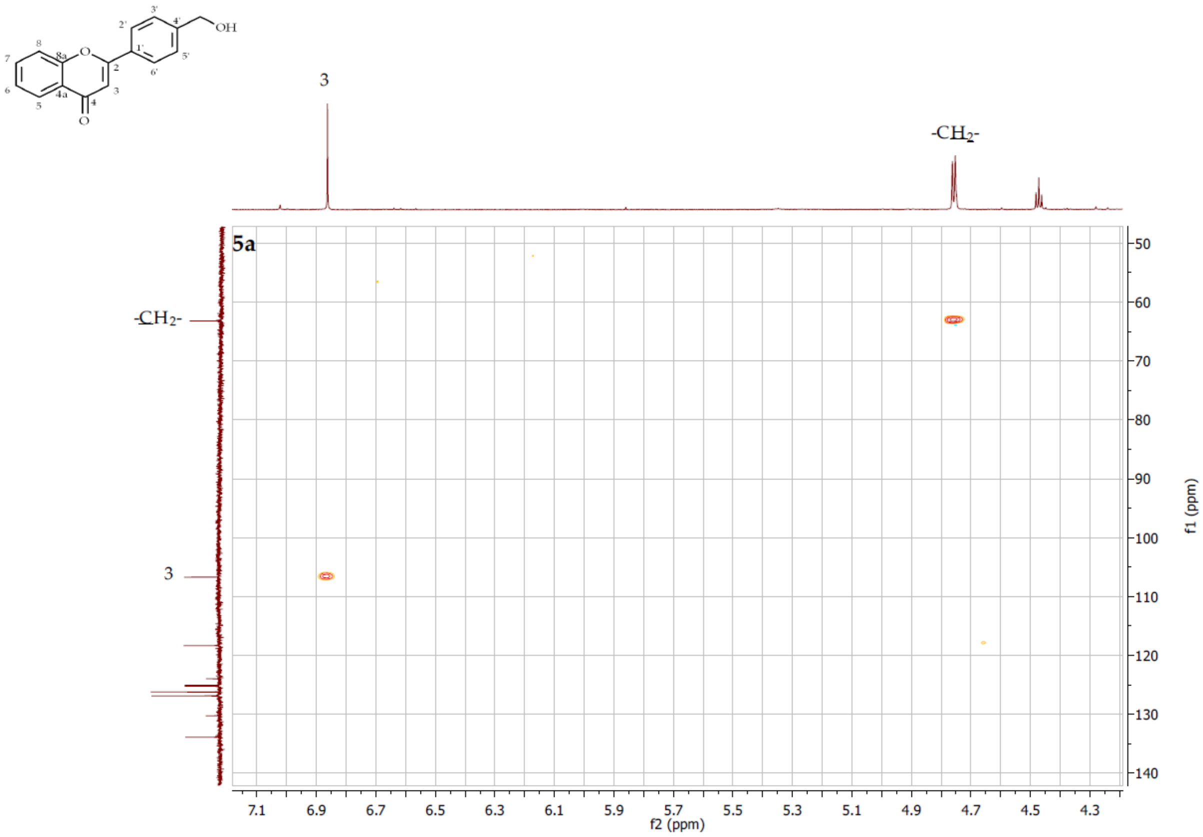

Figure S120. HSQC contour map – ^{1}H x ^{13}C expansion of 4′-hydroxymethylflavone (**5a**)

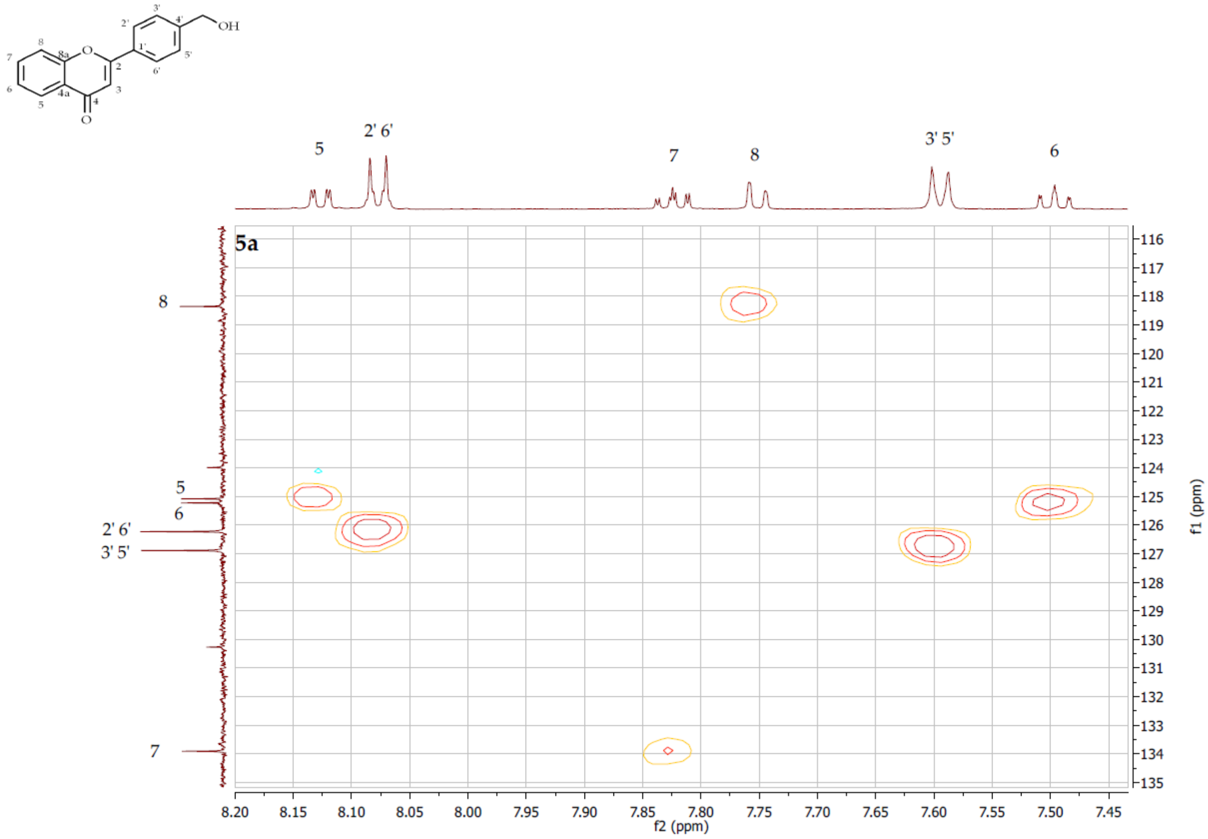

Figure S121. HSQC contour map – ^{1}H x ^{13}C expansion of 4′-hydroxymethylflavone (**5a**)

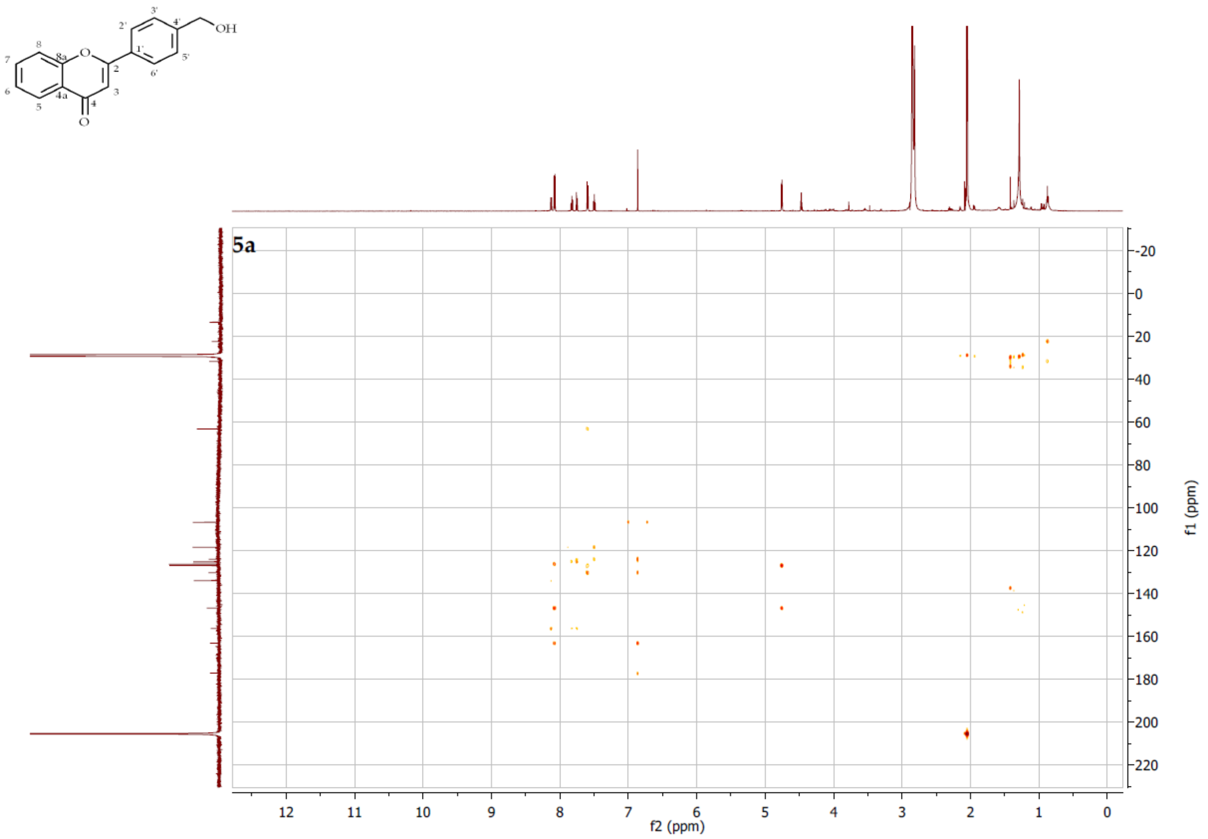

Figure S122. HMBC contour map – ^{1}H x ^{13}C of 4′-hydroxymethylflavone (**5a**)

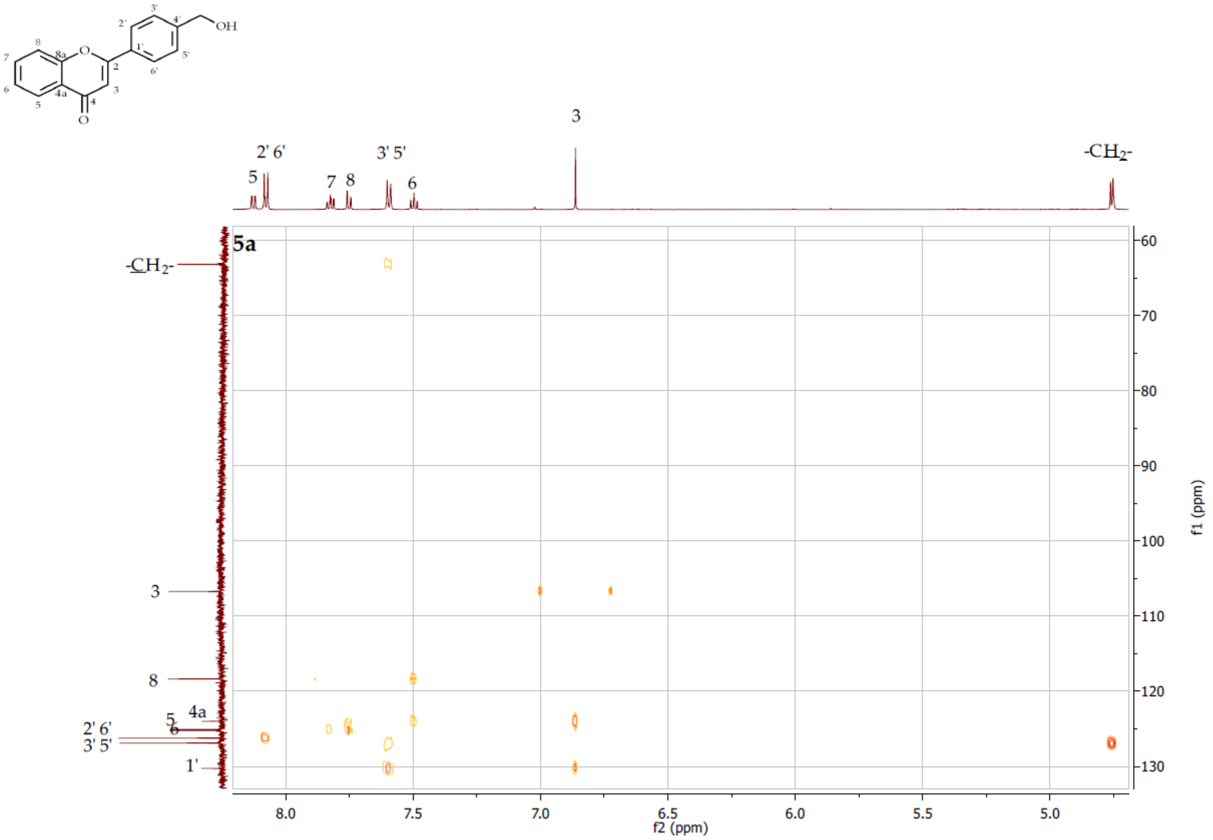

Figure S123. HMBC contour map – ^{1}H x ^{13}C expansion of 4′-hydroxymethylflavone (**5a**)

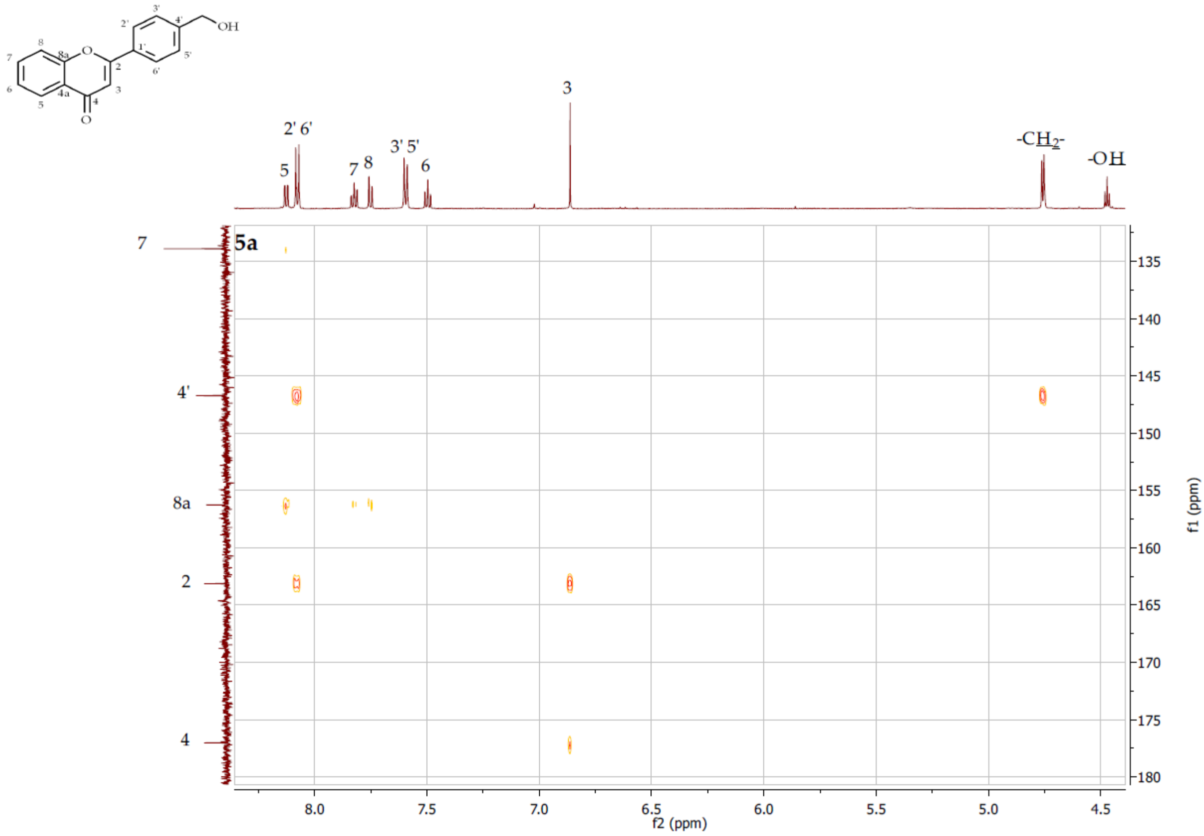

Figure S124. HMBC contour map – ^{1}H x ^{13}C expansion of 4′-hydroxymethylflavone (**5a**)

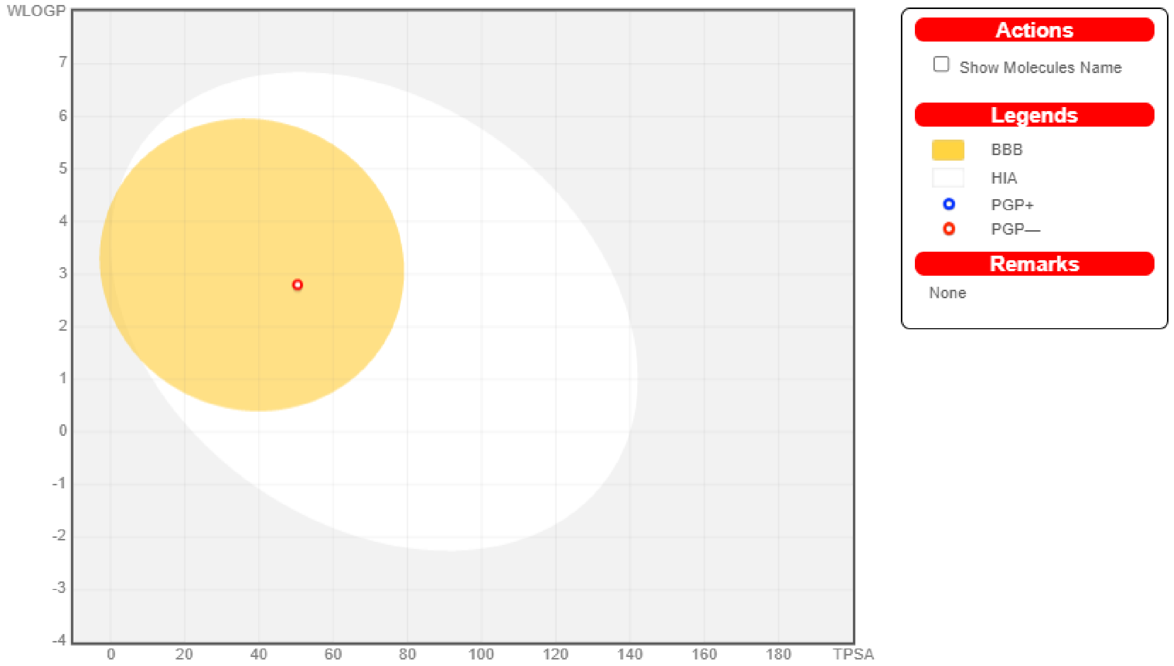

Molecule 1

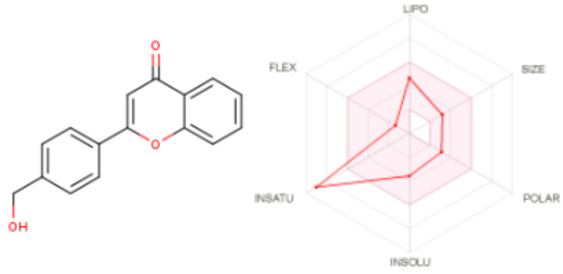

SMILES OCc1ccc(cc1)c1cc(=O)c2c(o1)cccc2

Physicochemical Properties	
Formula	C16H12O3
Molecular weight	252.26 g/mol
Num. heavy atoms	19
Num. arom. heavy atoms	16
Fraction Csp3	0.06
Num. rotatable bonds	2
Num. H-bond acceptors	3
Num. H-bond donors	1
Molar Refractivity	74.05
TPSA	50.44 Å²

Lipophilicity	
Log $P_{o/w}$ (iLOGP)	2.59
Log $P_{o/w}$ (XLOGP3)	2.67
Log $P_{o/w}$ (WLOGP)	2.80
Log $P_{o/w}$ (MLOGP)	1.64
Log $P_{o/w}$ (SILICOS-IT)	3.88
Consensus Log $P_{o/w}$	2.72

Water Solubility	
Log *S* (ESOL)	-3.58
Solubility	6.68e-02 mg/ml ; 2.65e-04 mol/l
Class	Soluble
Log *S* (Ali)	-3.38
Solubility	1.05e-01 mg/ml ; 4.16e-04 mol/l
Class	Soluble
Log *S* (SILICOS-IT)	-5.96
Solubility	2.75e-04 mg/ml ; 1.09e-06 mol/l
Class	Moderately soluble

Pharmacokinetics	
GI absorption	High
BBB permeant	Yes
P-gp substrate	No
CYP1A2 inhibitor	Yes
CYP2C19 inhibitor	Yes
CYP2C9 inhibitor	No
CYP2D6 inhibitor	Yes
CYP3A4 inhibitor	Yes
Log K_p (skin permeation)	-5.94 cm/s

Druglikeness	
Lipinski	Yes; 0 violation
Ghose	Yes
Veber	Yes
Egan	Yes
Muegge	Yes
Bioavailability Score	0.55

Medicinal Chemistry	
PAINS	0 alert
Brenk	0 alert
Leadlikeness	Yes
Synthetic accessibility	3.04

Figure S125. 4′-Hydroxymethylflavone (**5a**) physicochemical and ADME parameters prediction using the SwissADME modelling

Pa	Pi	Activity
0,935	0,005	Membrane integrity agonist
0,915	0,002	4-Nitrophenol 2-monooxygenase inhibitor
0,911	0,005	HIF1A expression inhibitor
0,892	0,003	27-Hydroxycholesterol 7alpha-monooxygenase inhibitor
0,893	0,005	Chlordecone reductase inhibitor
0,882	0,003	Cholestanetriol 26-monooxygenase inhibitor
0,881	0,004	Membrane permeability inhibitor
0,879	0,005	Anaphylatoxin receptor antagonist
0,871	0,004	Kinase inhibitor
0,867	0,003	CYP2B5 substrate

Figure S126. 4′-Hydroxymethylflavone (**5a**) biological activity prediction using the Way2Drug Pass online modelling

Name	Confidence	ChEMBL ID
Yersinia pestis	0.4722	CHEMBL614597
Pseudomonas fluorescens	0.3556	CHEMBL612500
Streptococcus pneumoniae R6	0.2843	CHEMBL2366794
Bacillus thuringiensis	0.2651	CHEMBL614959
RESISTANT Burkholderia pseudomallei	0.2610	CHEMBL3140323
Bacillus subtilis subsp. subtilis str. 168	0.2315	CHEMBL613315
Salmonella enterica subsp. enterica	0.2286	CHEMBL613044
Mycobacterium mageritense	0.2285	CHEMBL612959
RESISTANT Acinetobacter pittii	0.1785	CHEMBL3140321
Mycobacterium	0.1509	CHEMBL614981
Kocuria rhizophila	0.1496	CHEMBL1075349
RESISTANT Bacillus anthracis	0.1463	CHEMBL613904
Mycobacterium aurum	0.1449	CHEMBL612952
Helicobacter pylori SS1	0.1227	CHEMBL613197
RESISTANT Enterobacter	0.1212	CHEMBL614439

Figure S127. 4′-Hydroxymethylflavone (**5a**) antibacterial activity prediction using the Way2Drug AntiBac-Pred modelling

Name	Confidence	ChEMBL ID
Yarrowia lipolytica	0.1471	CHEMBL612844
Epidermophyton floccosum	0.1392	CHEMBL612386
Microsporum	0.0682	CHEMBL612278

Figure S128. 4′-Hydroxymethylflavone (**5a**) antifungal activity prediction using the Way2Drug AntiFun-Pred modelling

Virus	Protein target	Confidence
Human immunodeficiency virus 2	Human immunodeficiency virus type 2 integrase	0.7413
Severe acute respiratory syndrome coronavirus 2	Replicase polyprotein 1ab	0.5088
Dengue virus type 2	Genome polyprotein	0.4718
Vaccinia virus<br>(strain Western Reserve)<br>(VACV)<br>(Vaccinia virus<br>(strainWR))	DNA polymerase	0.2440
Varicella-zoster virus<br>(strain Dumas)<br>(HHV-3)<br>(Human herpesvirus 3)	DNA polymerase	0.2199
Herpes simplex virus<br>(type 1 / strain 17)	Human herpesvirus 1 DNA polymerase	0.2199
SARS coronavirus	Replicase polyprotein 1ab	0.1268
SARS coronavirus	SARS coronavirus 3C-like proteinase	0.0858
Macacine herpesvirus 1	Thymidine kinase	0.0623

Figure S129. 4′-Hydroxymethylflavone (**5a**) antiviral activity prediction using the Way2Drug AntiVir-Pred modelling

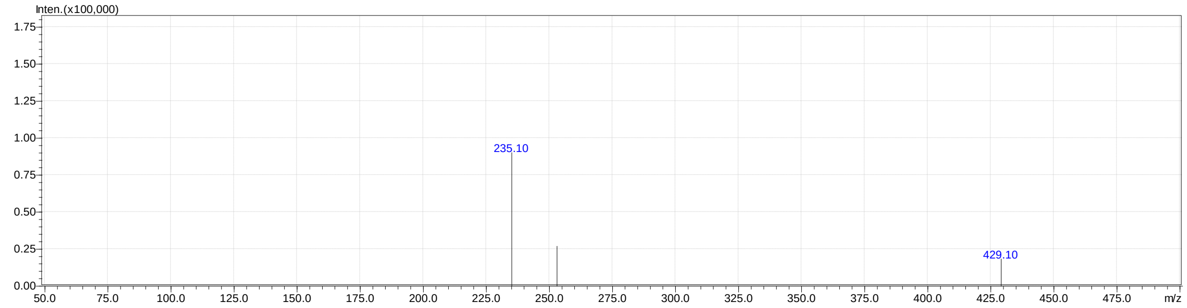

Figure S130. MS analysis flavone 4′-methylene-*O*-*β*-D-(4′′-*O*-methyl)-glucopyranoside (**5b**)

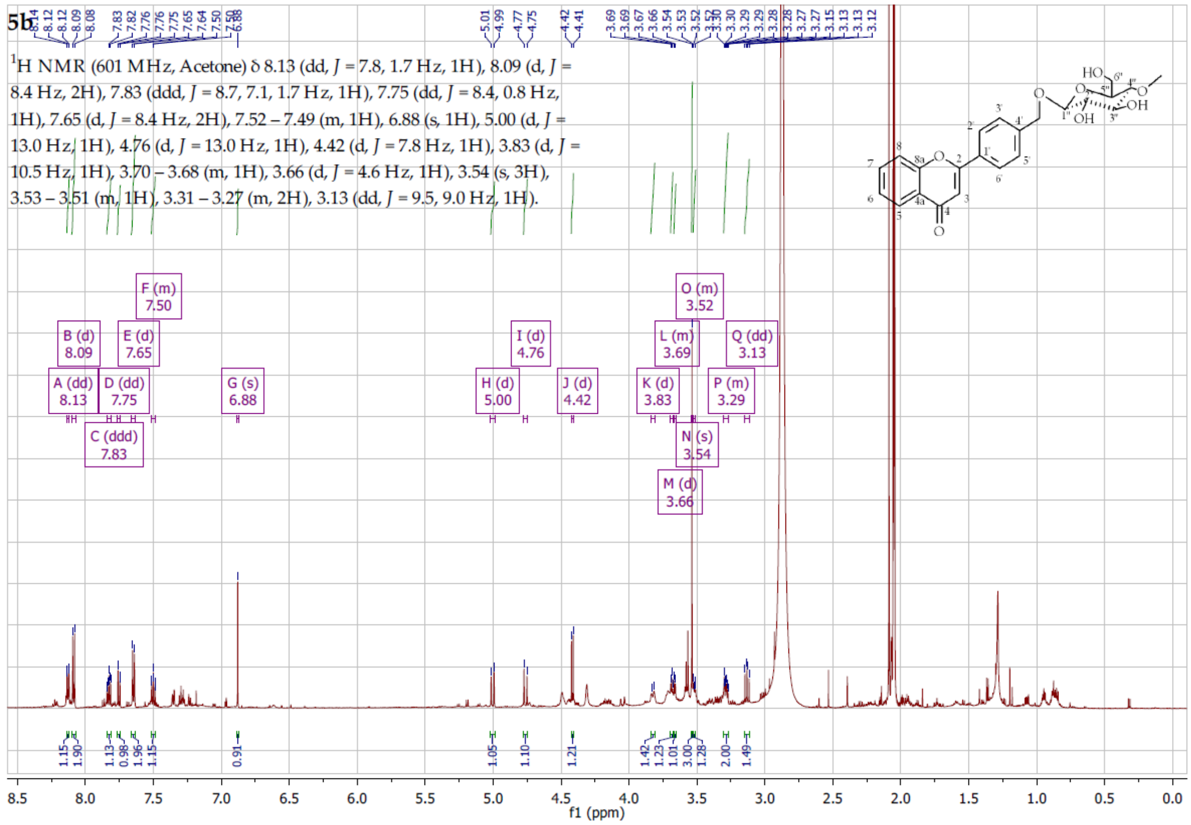

Figure S131. ^{1}H NMR spectrum (*δ*, acetone-d6, 600 MHz) of flavone 4′-methylene-*O*-*β*-D-(4′′-*O*-methyl)-glucopyranoside (**5b**)

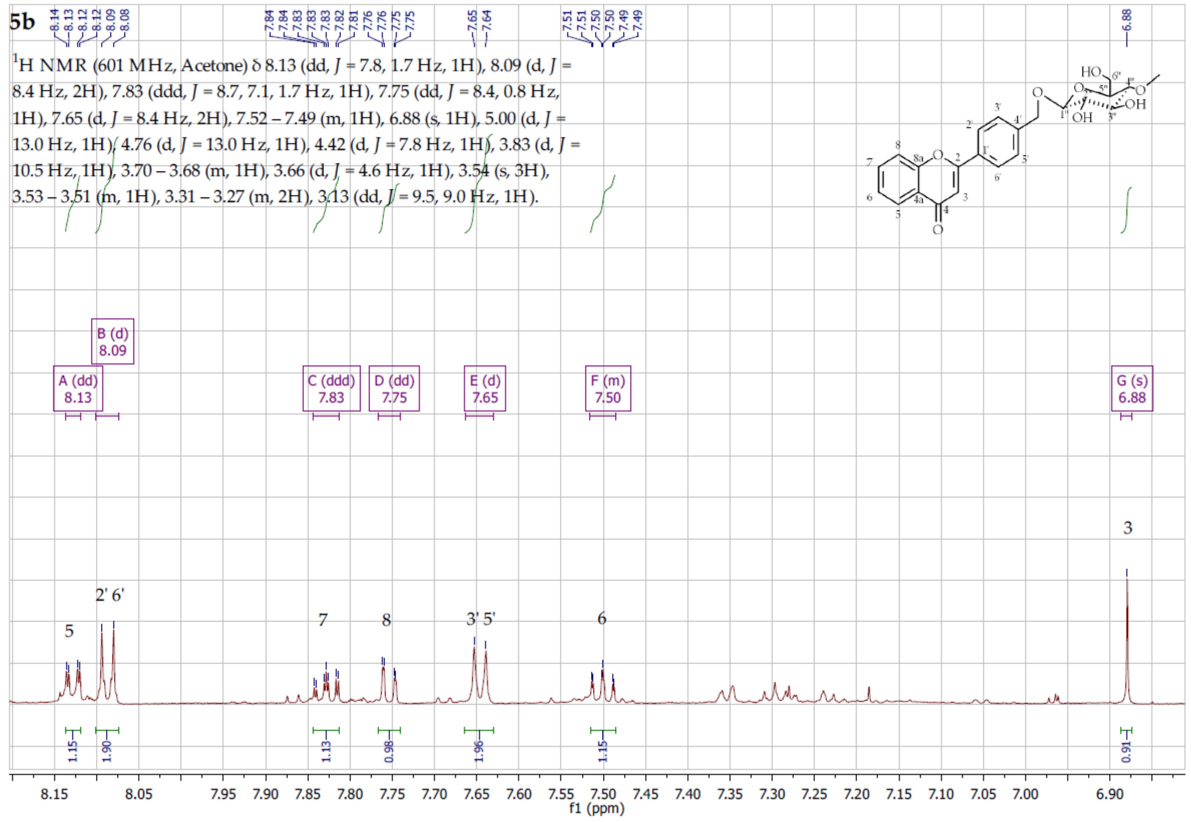

Figure S132. ^{1}H NMR spectrum expansion (*δ*, acetone-d6, 600 MHz) of flavone 4′-methylene-*O*-*β*-D-(4′′-*O*-methyl)-glucopyranoside (**5b**)

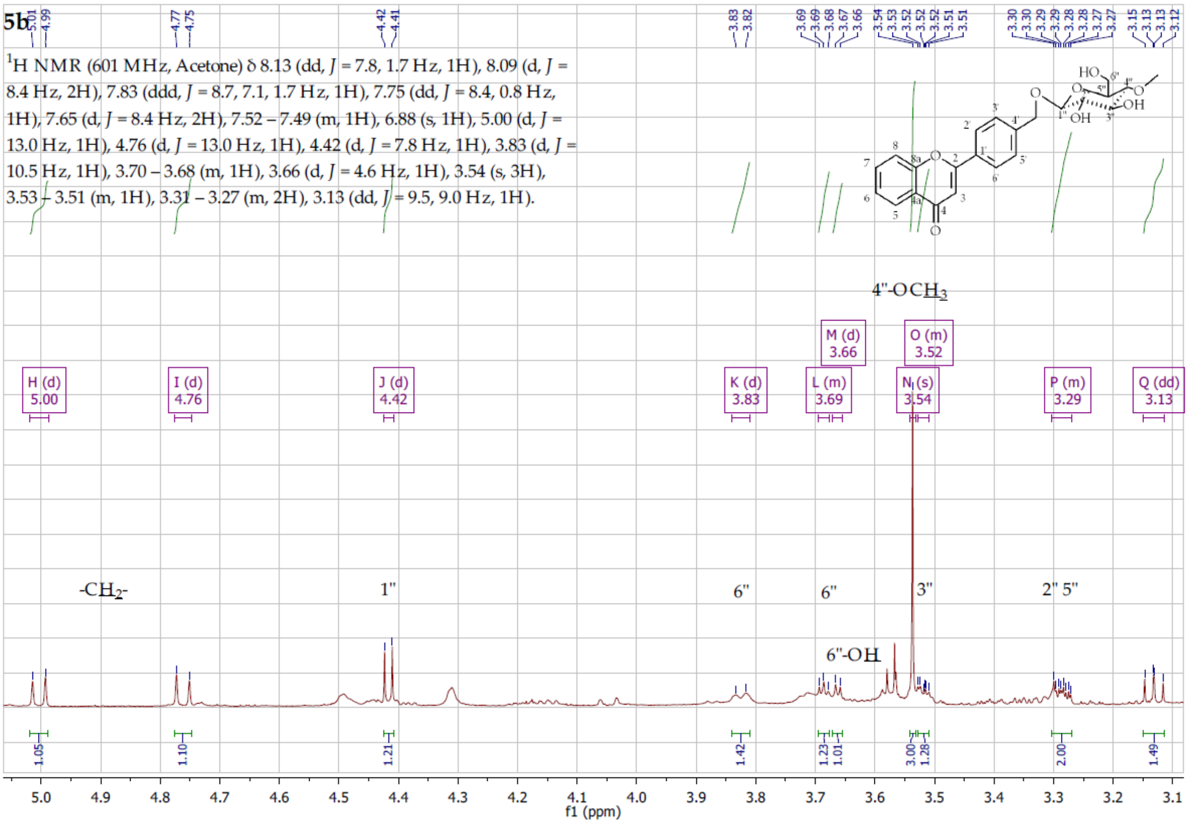

Figure S133. ¹H NMR spectrum expansion (*δ*, acetone-d6, 600 MHz) of flavone 4′-methylene-*O*-*β*-D-(4′′-*O*-methyl)-glucopyranoside (**5b**)

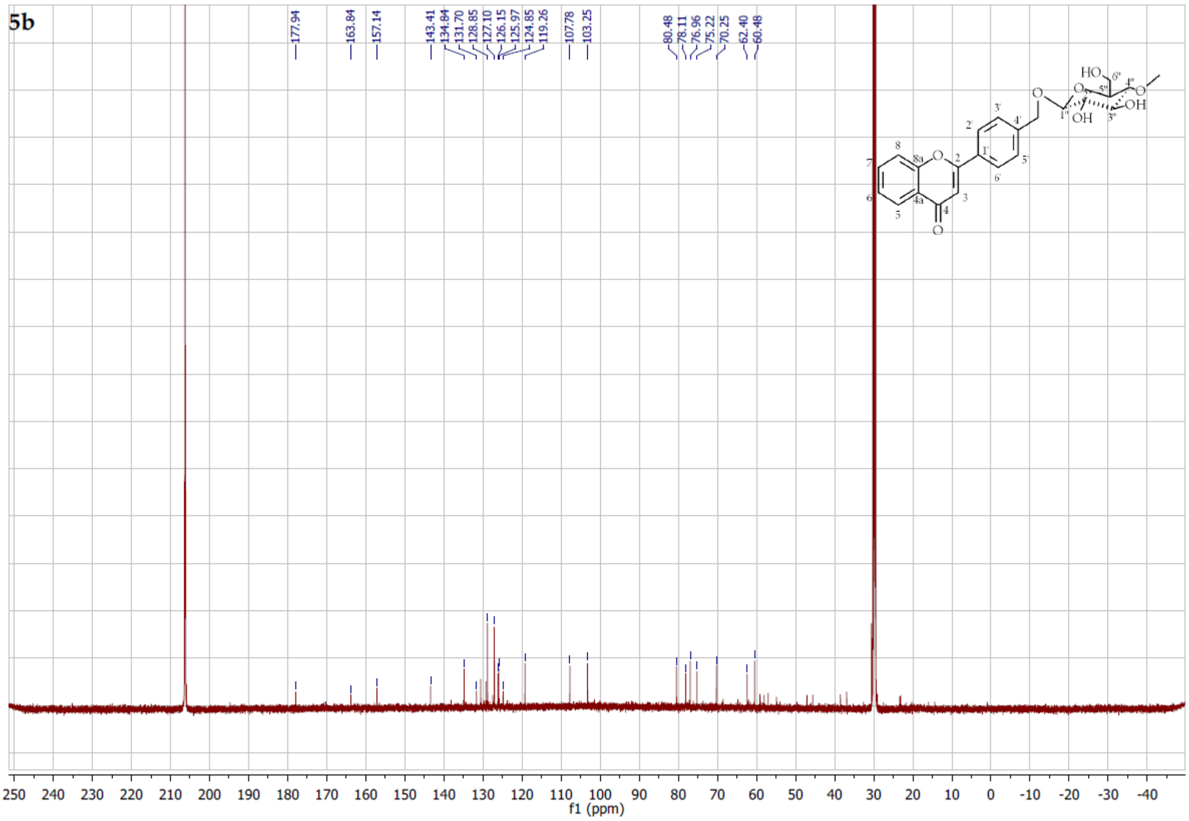

Figure S134. ¹³C NMR spectrum expansion (*δ*, acetone-d6, 151 MHz) of flavone 4′-methylene-*O*-*β*-D-(4′′-*O*-methyl)-glucopyranoside (**5b**)

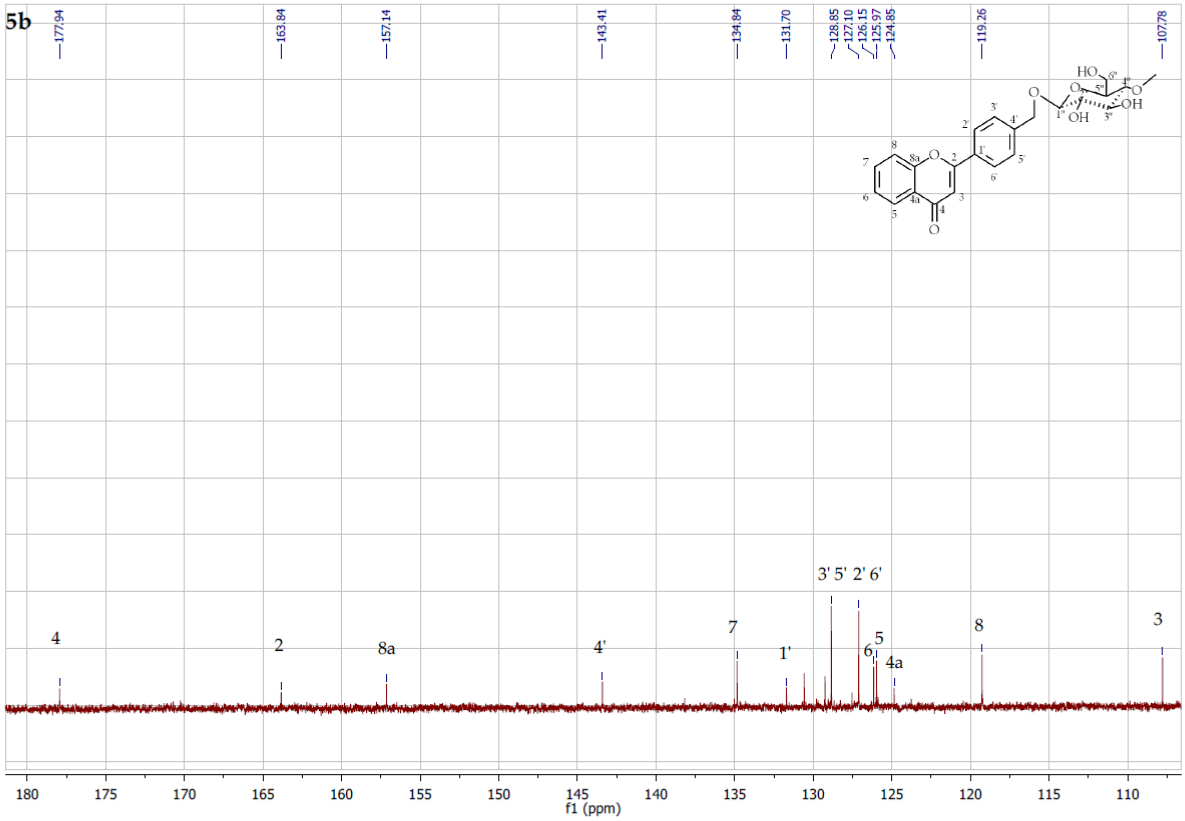

Figure S135. ^{13}C NMR spectrum expansion (*δ*, acetone-d6, 151 MHz) of flavone 4′-methylene-*O*-*β*-D-(4′′-*O*-methyl)-glucopyranoside (**5b**)

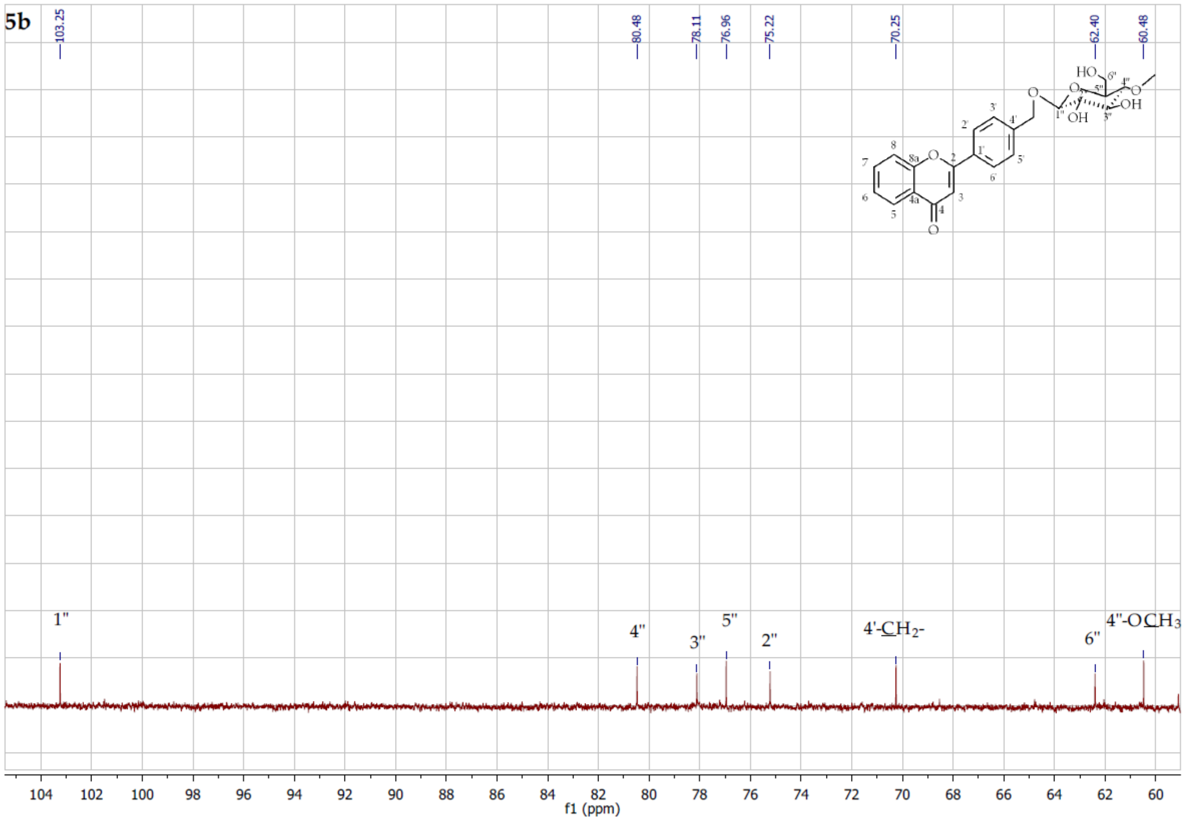

Figure S136. ^{13}C NMR spectrum expansion (*δ*, acetone-d6, 151 MHz) of flavone 4′-methylene-*O*-*β*-D-(4′′-*O*-methyl)-glucopyranoside (**5b**)

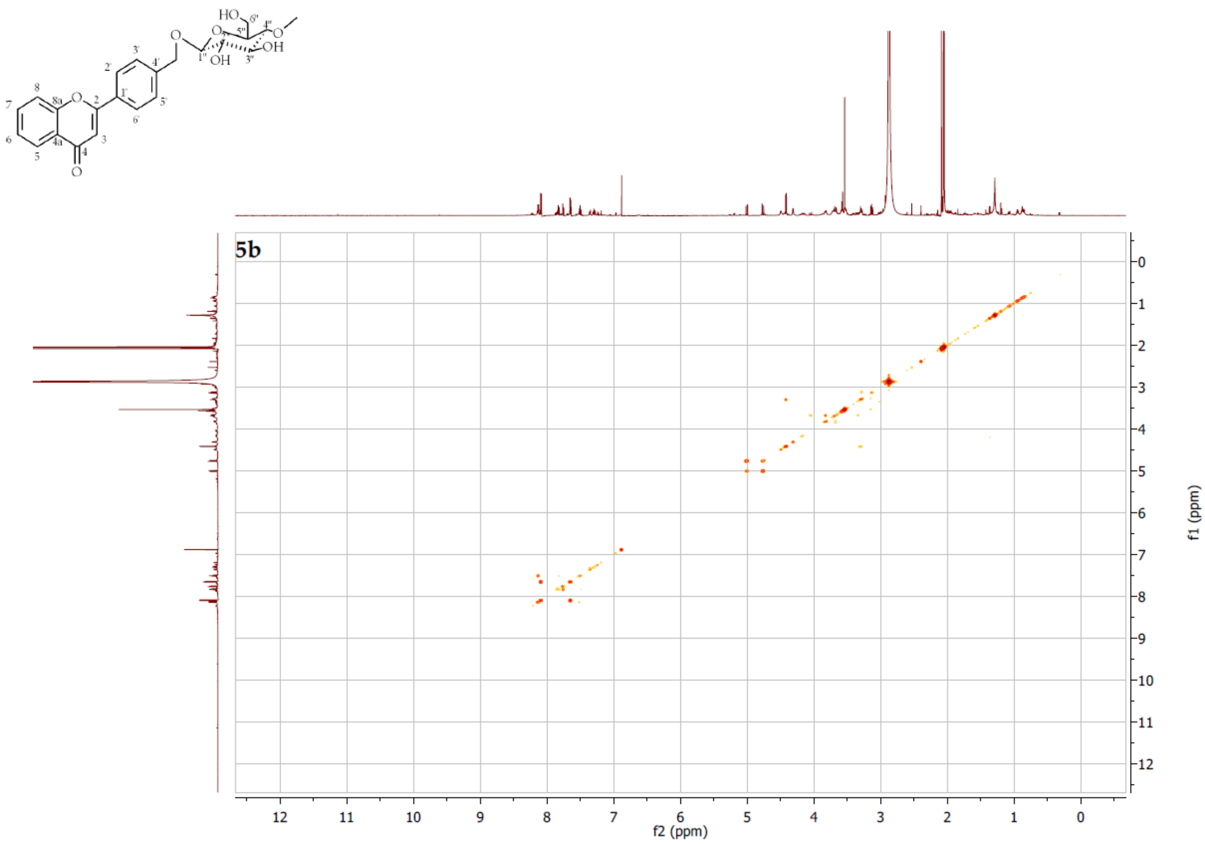

Figure S137. COSY contour map – $^{1}H \times {}^{1}H$ of flavone 4′-methylene-*O*-*β*-D-(4′′-*O*-methyl)-glucopyranoside (**5b**)

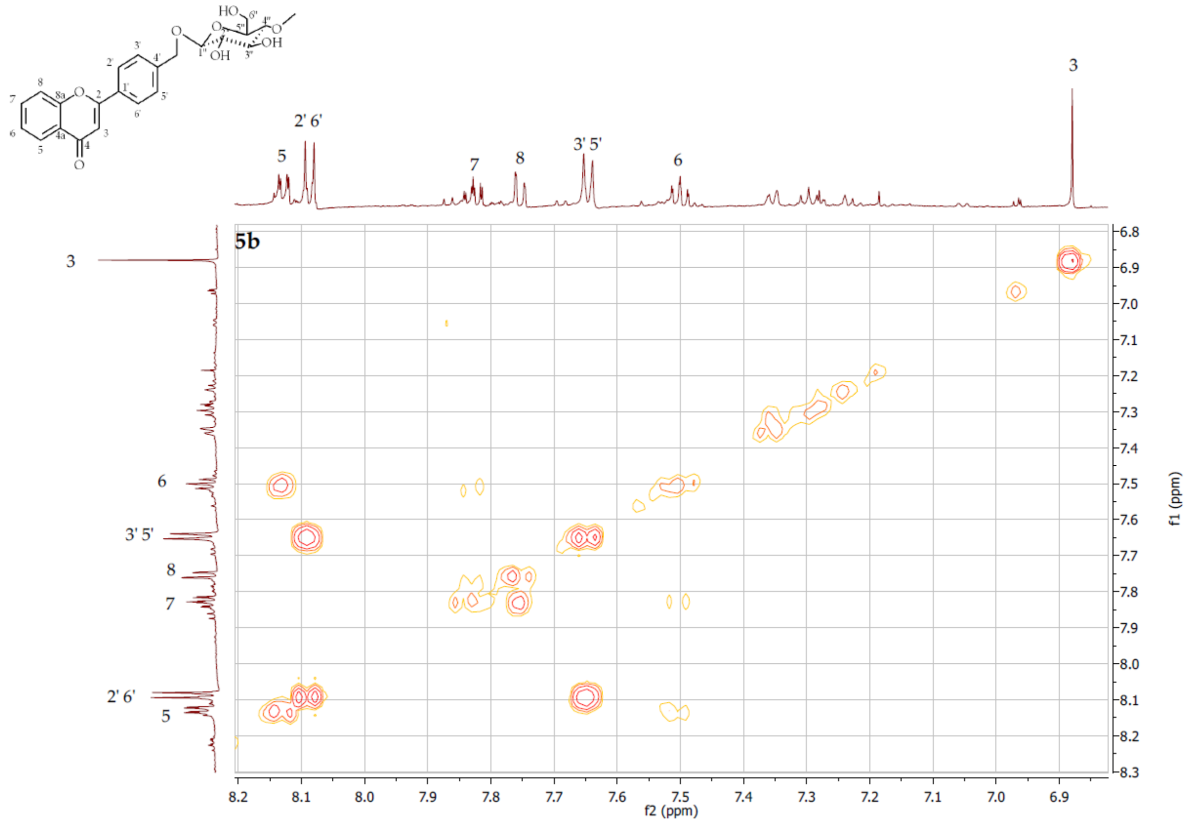

Figure S138. COSY contour map – $^{1}H \times {}^{1}H$ expansion of flavone 4′-methylene-*O*-*β*-D-(4′′-*O*-methyl)-glucopyranoside (**5b**)

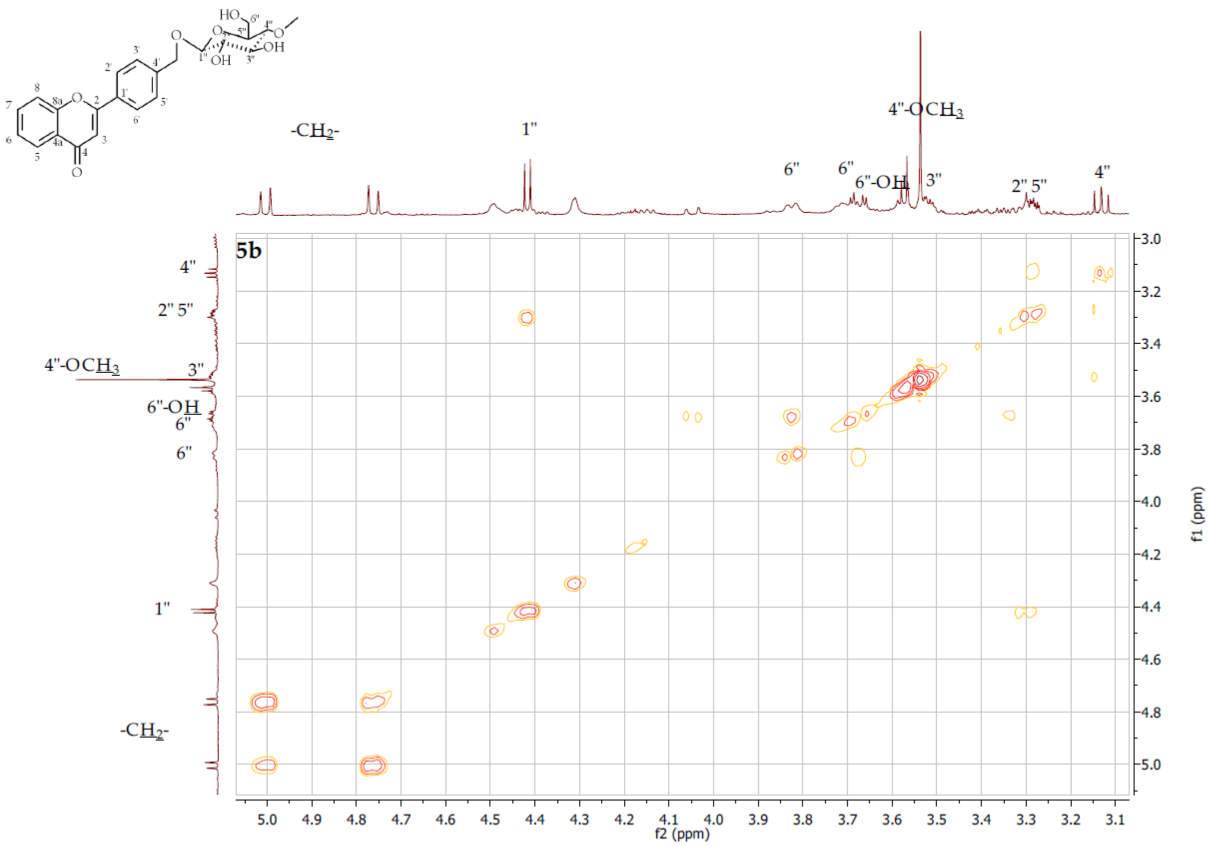

Figure S139. COSY contour map – $^{1}H \times {}^{1}H$ expansion of flavone 4′-methylene-*O*-*β*-D-(4′′-*O*-methyl)-glucopyranoside (**5b**)

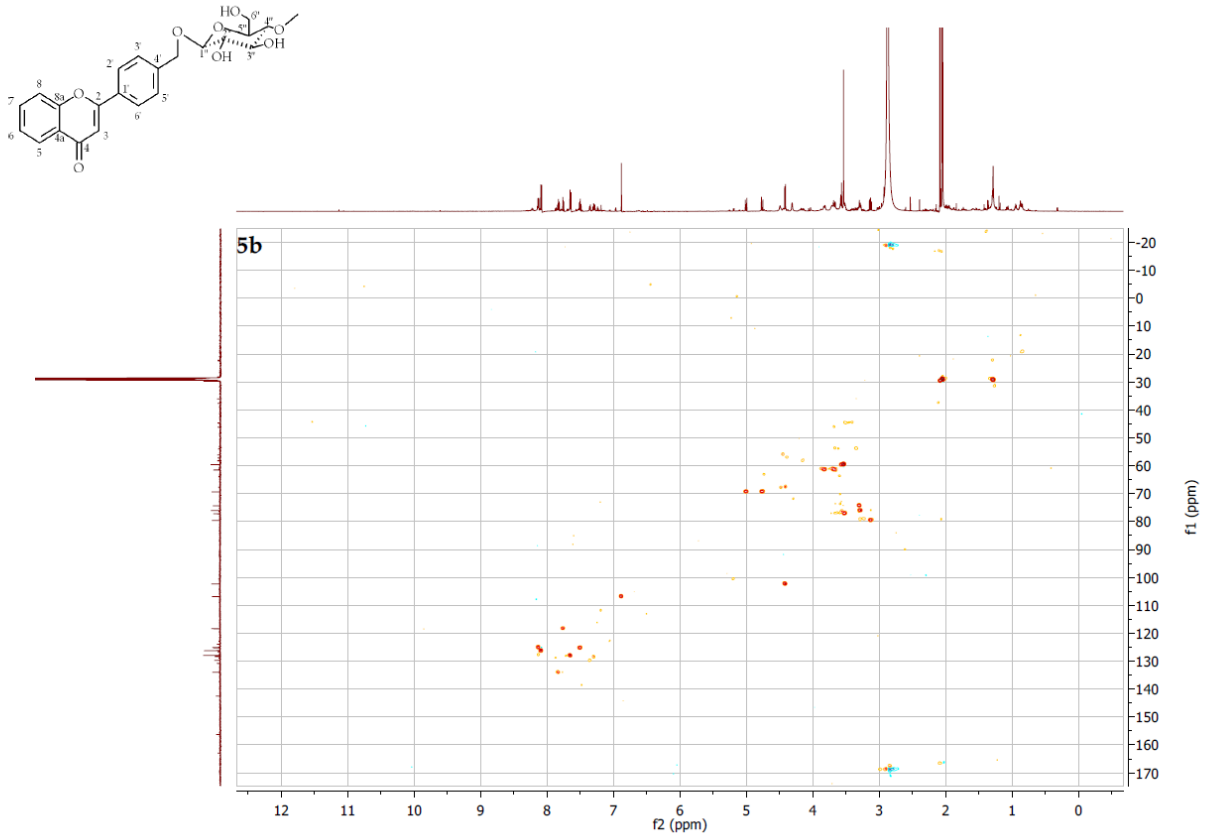

Figure S140. HSQC contour map – $^{1}H \times {}^{13}C$ of flavone 4′-methylene-*O*-*β*-D-(4′′-*O*-methyl)-glucopyranoside (**5b**)

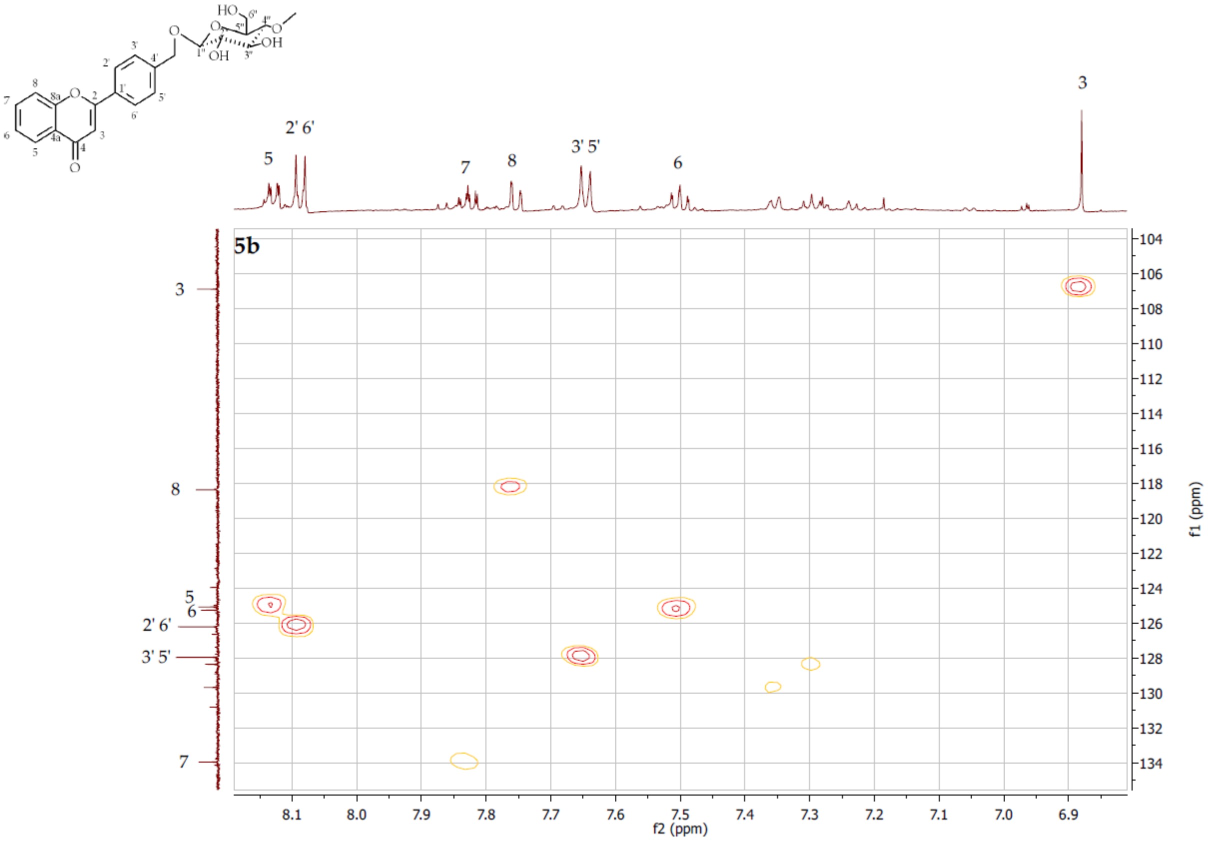

Figure S141. HSQC contour map – ^{1}H x ^{13}C expansion of flavone 4′-methylene-*O*-*β*-D-(4′′-*O*-methyl)-glucopyranoside (**5b**)

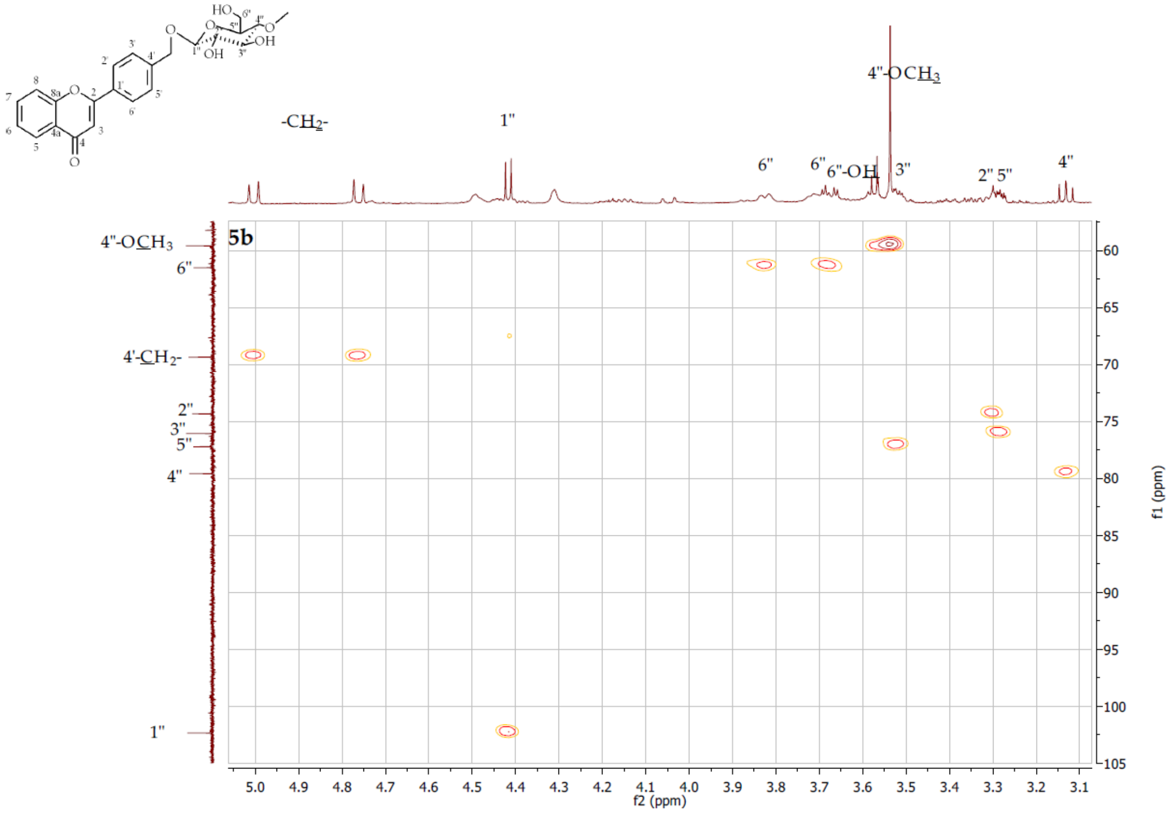

Figure S142. HSQC contour map – ^{1}H x ^{13}C expansion of flavone 4′-methylene-*O*-*β*-D-(4′′-*O*-methyl)-glucopyranoside (**5b**)

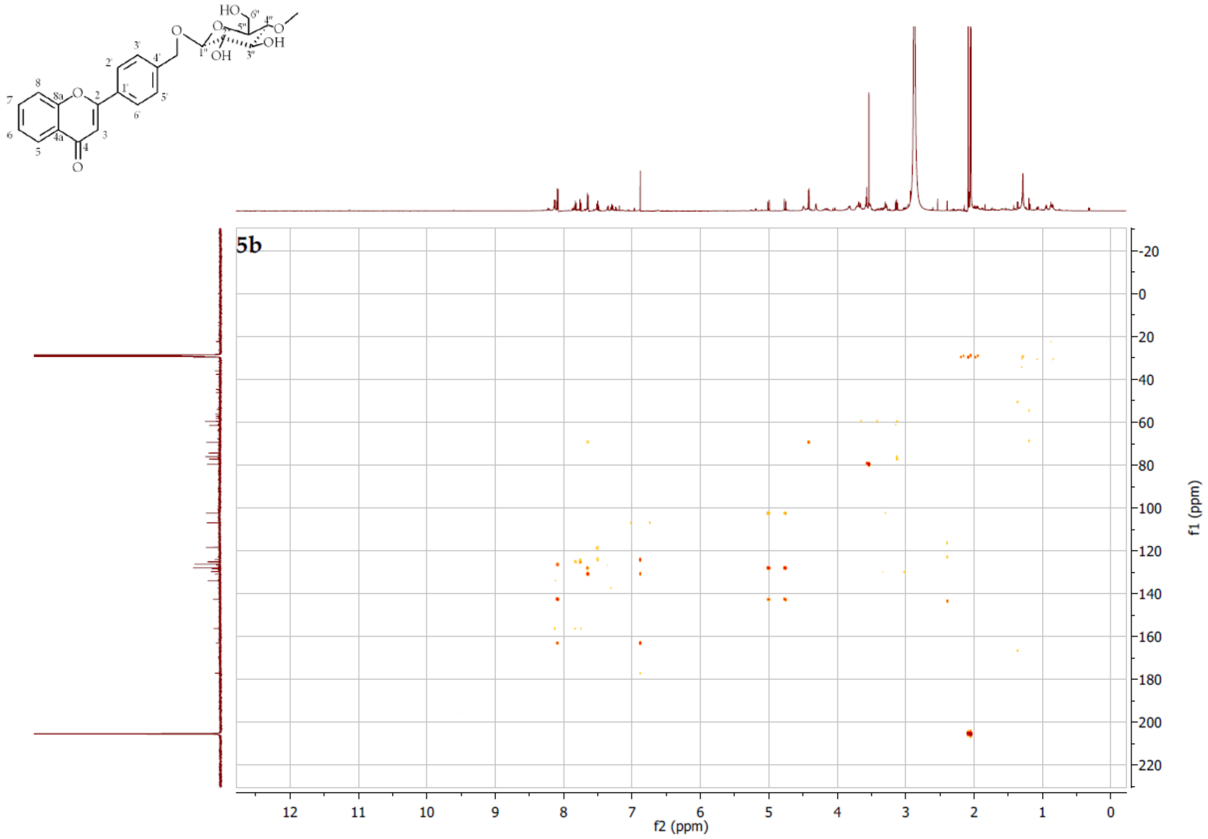

Figure S143. HMBC contour map – ^{1}H x ^{13}C of flavone 4′-methylene-*O*-*β*-D-(4′′-*O*-methyl)-glucopyranoside (**5b**)

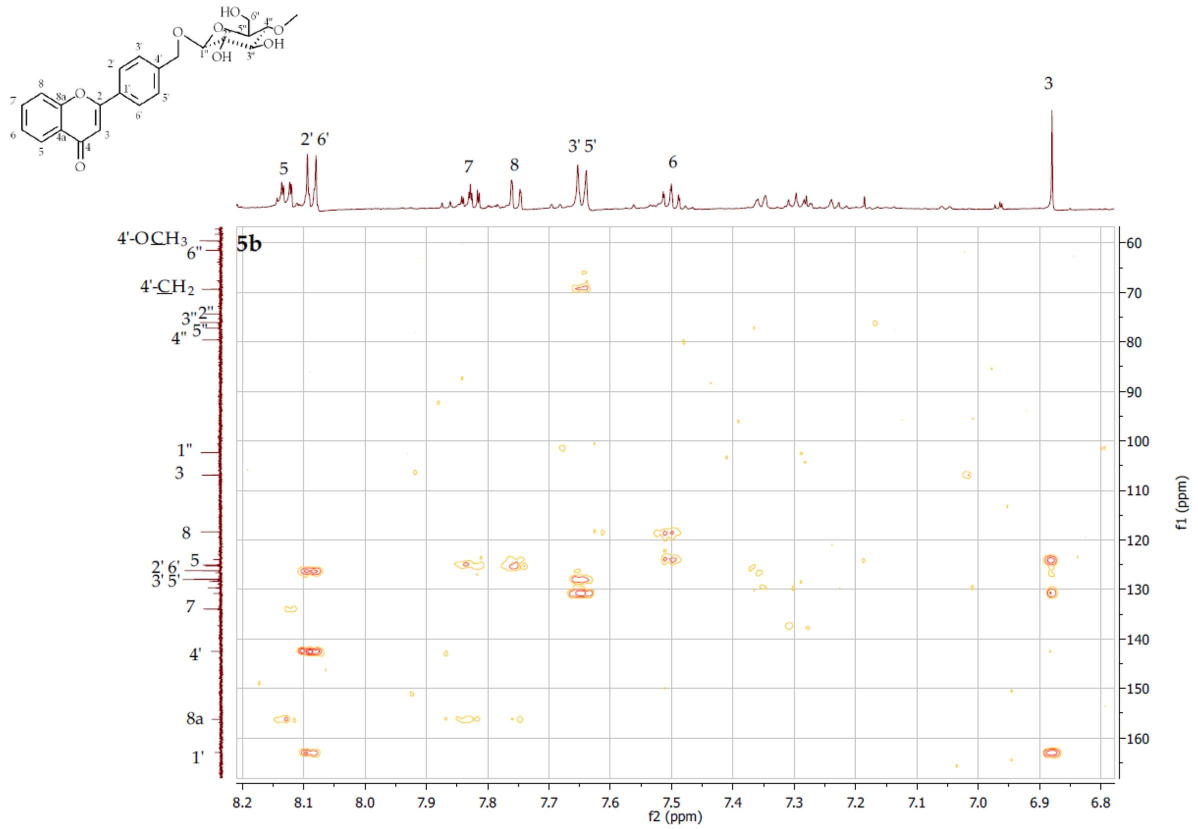

Figure S144. HMBC contour map – ^{1}H x ^{13}C expansion of flavone 4′-methylene-*O*-*β*-D-(4′′-*O*-methyl)-glucopyranoside (**5b**)

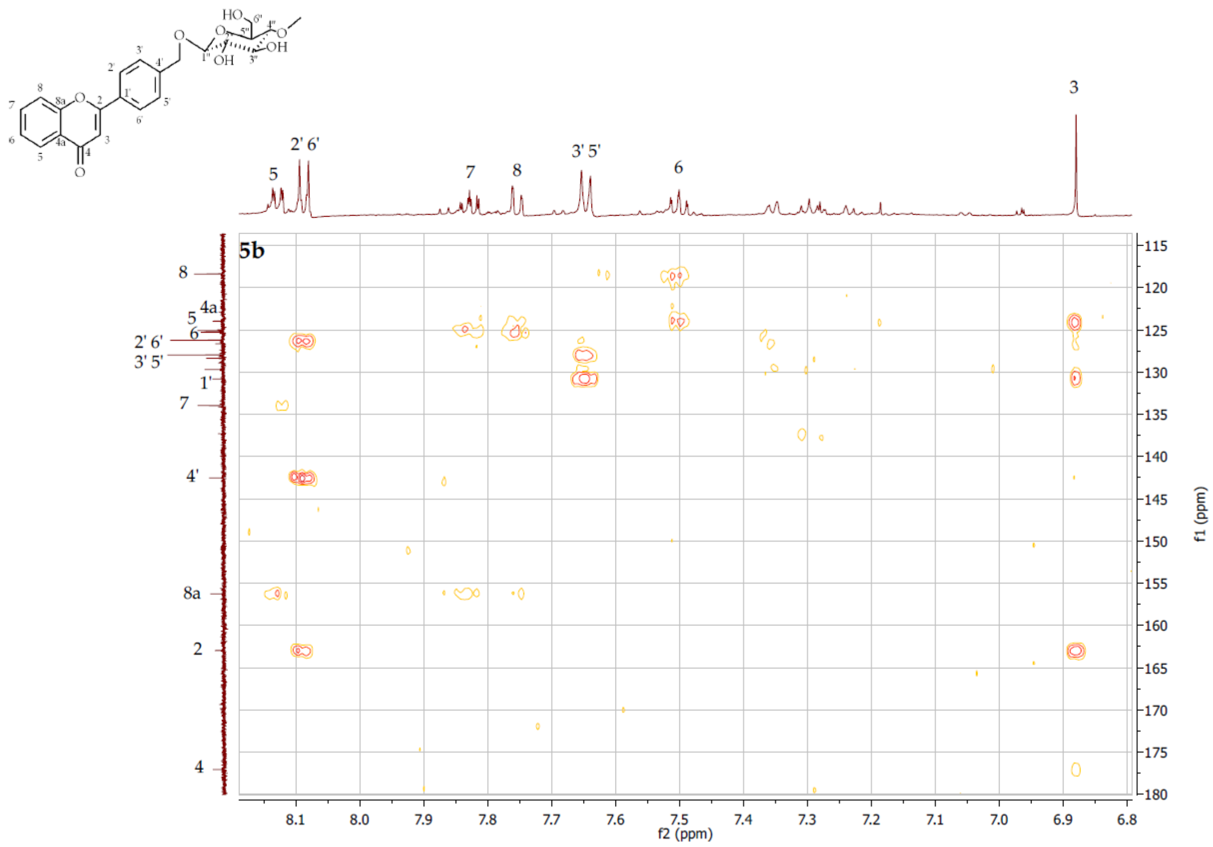

Figure S145. HMBC contour map – ^{1}H x ^{13}C expansion of flavone 4′-methylene-*O*-*β*-D-(4′′-*O*-methyl)-glucopyranoside (**5b**)

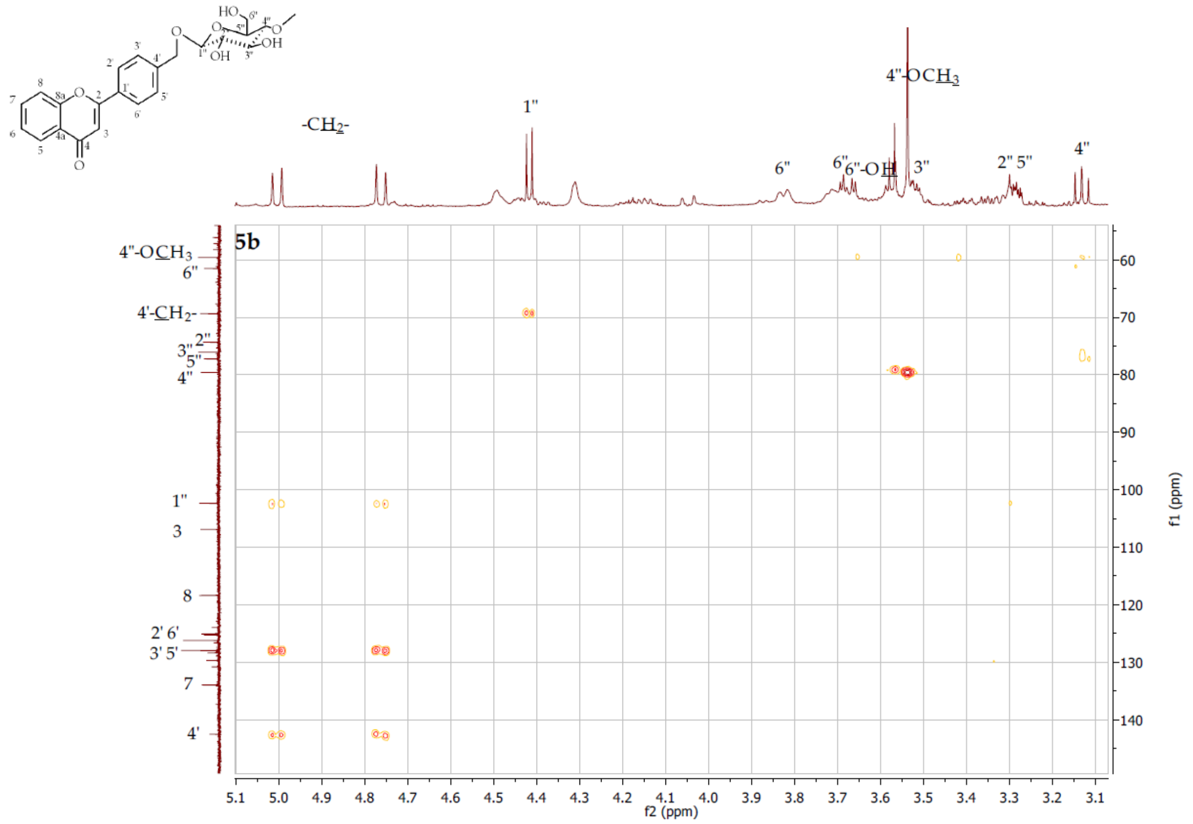

Figure S146. HMBC contour map – ^{1}H x ^{13}C expansion of flavone 4′-methylene-*O*-*β*-D-(4′′-*O*-methyl)-glucopyranoside (**5b**)

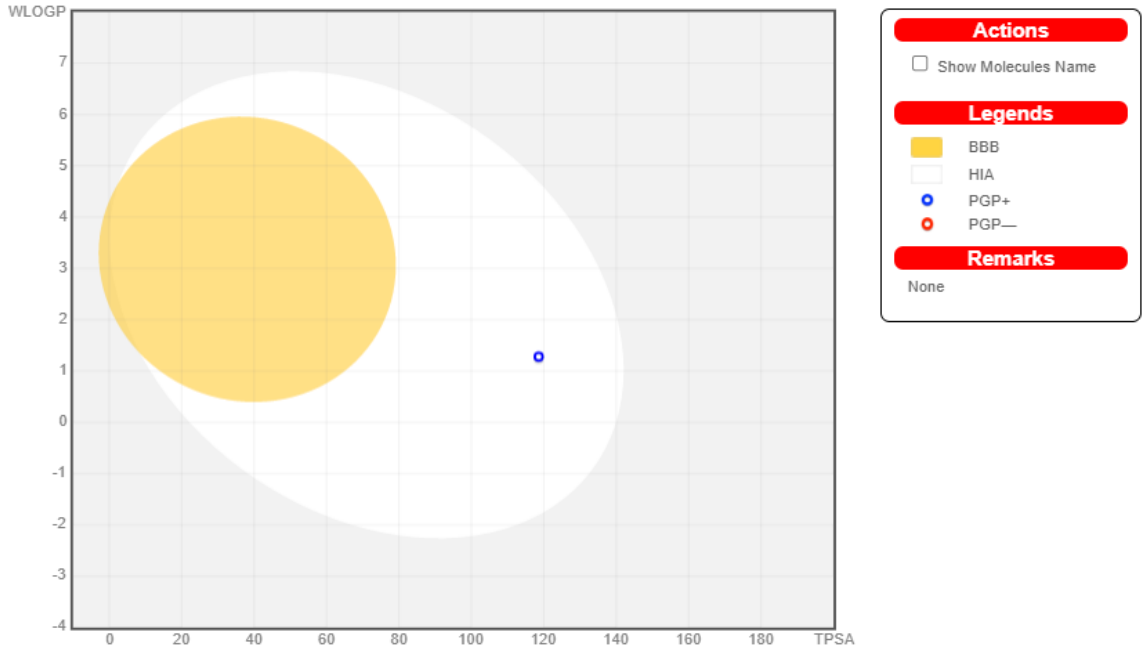

Molecule 1

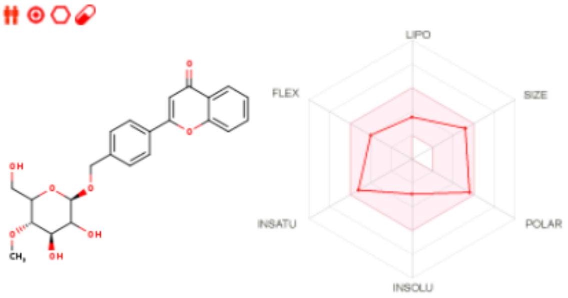

SMILES OCC1O[C@@H](OCc2ccc(cc2)c2cc(=O)c3c(o2)cccc3)C([C@H]([C@@H]1OC)O)O

Physicochemical Properties	
Formula	C23H24O8
Molecular weight	428.43 g/mol
Num. heavy atoms	31
Num. arom. heavy atoms	16
Fraction Csp3	0.35
Num. rotatable bonds	6
Num. H-bond acceptors	8
Num. H-bond donors	3
Molar Refractivity	111.16
TPSA	118.59 Å²

Lipophilicity	
Log $P_{o/w}$ (iLOGP)	2.93
Log $P_{o/w}$ (XLOGP3)	0.69
Log $P_{o/w}$ (WLOGP)	1.28
Log $P_{o/w}$ (MLOGP)	-0.43
Log $P_{o/w}$ (SILICOS-IT)	2.24
Consensus Log $P_{o/w}$	1.34

Water Solubility	
Log *S* (ESOL)	-2.92
Solubility	5.19e-01 mg/ml ; 1.21e-03 mol/l
Class	Soluble
Log *S* (Ali)	-2.76
Solubility	7.49e-01 mg/ml ; 1.75e-03 mol/l
Class	Soluble
Log *S* (SILICOS-IT)	-4.95
Solubility	4.77e-03 mg/ml ; 1.11e-05 mol/l
Class	Moderately soluble

Pharmacokinetics	
GI absorption	High
BBB permeant	No
P-gp substrate	Yes
CYP1A2 inhibitor	No
CYP2C19 inhibitor	No
CYP2C9 inhibitor	No
CYP2D6 inhibitor	No
CYP3A4 inhibitor	Yes
Log K_p (skin permeation)	-8.42 cm/s

Druglikeness	
Lipinski	Yes; 0 violation
Ghose	Yes
Veber	Yes
Egan	Yes
Muegge	Yes
Bioavailability Score	0.55

Medicinal Chemistry	
PAINS	0 alert
Brenk	0 alert
Leadlikeness	No; 1 violation: MW>350
Synthetic accessibility	5.33

Figure S147. Flavone 4′-methylene-*O*-*β*-D-(4′′-*O*-methyl)-glucopyranoside (**5b**) physicochemical and ADME parameters prediction using the SwissADME modelling

Pa	Pi	Activity
0,957	0,002	Membrane permeability inhibitor
0,942	0,002	Vasoprotector
0,920	0,007	Membrane integrity agonist
0,913	0,008	CDP-glycerol glycerophosphotransferase inhibitor
0,899	0,005	Benzoate-CoA ligase inhibitor
0,894	0,004	Anaphylatoxin receptor antagonist
0,892	0,003	Hepatoprotectant
0,878	0,008	Alkenylglycerophosphocholine hydrolase inhibitor
0,871	0,003	Cardioprotectant
0,863	0,008	Sugar-phosphatase inhibitor

Figure S148. Flavone 4′-methylene-*O*-*β*-D-(4′′-*O*-methyl)-glucopyranoside (**5b**) biological activity prediction using the Way2Drug Pass online modelling

Name	Confidence	ChEMBL ID
Clostridium ramosum	0.6319	CHEMBL614971
RESISTANT Acinetobacter pittii	0.5844	CHEMBL3140321
Actinomyces meyeri	0.5835	CHEMBL612289
Mycobacterium mageritense	0.5678	CHEMBL612959
RESISTANT Mycobacterium ulcerans	0.5614	CHEMBL612965
Clostridium cadaveris	0.5549	CHEMBL614970
Yersinia pestis	0.4399	CHEMBL614597
Nocardia transvalensis	0.4242	CHEMBL613234
Lactobacillus plantarum	0.4199	CHEMBL614973
Staphylococcus lugdunensis	0.4047	CHEMBL613303
Pseudomonas fluorescens	0.4042	CHEMBL612500
Clostridium sordellii	0.3944	CHEMBL613072
Streptococcus oralis	0.3940	CHEMBL613305
RESISTANT Staphylococcus aureus subsp. aureus RN4220	0.3887	CHEMBL2366906
Fusobacterium sp.	0.3442	CHEMBL612690

Figure S149. Flavone 4′-methylene-*O*-*β*-D-(4′′-*O*-methyl)-glucopyranoside (**5b**) antibacterial activity prediction using the Way2Drug AntiBac-Pred modelling

Name	Confidence	ChEMBL ID
Rhizopus oryzae	0.3257	CHEMBL612306
Absidia corymbifera	0.3067	CHEMBL612369
Trichophyton mentagrophytes	0.1971	CHEMBL613162
Aspergillus niger	0.1703	CHEMBL358
Saccharomyces cerevisiae	0.1416	CHEMBL361
Mucor	0.0665	CHEMBL612521
Penicillium marneffei	0.0131	CHEMBL612994
Epidermophyton floccosum	0.0073	CHEMBL612386

Figure S150. Flavone 4′-methylene-*O*-*β*-D-(4′′-*O*-methyl)-glucopyranoside (**5b**) antifungal activity prediction using the Way2Drug AntiFun-Pred modelling

Virus	Protein target	Confidence
Severe acute respiratory syndrome coronavirus 2	Replicase polyprotein 1ab	0.7858
Human immunodeficiency virus 2	Human immunodeficiency virus type 2 integrase	0.4219
Varicella-zoster virus (strain Dumas) (HHV-3) (Human herpesvirus 3)	Thymidine kinase	0.0180

Figure S151. Flavone 4′-methylene-*O*-*β*-D-(4′′-*O*-methyl)-glucopyranoside (**5b**) antiviral activity prediction using the Way2Drug AntiVir-Pred modelling

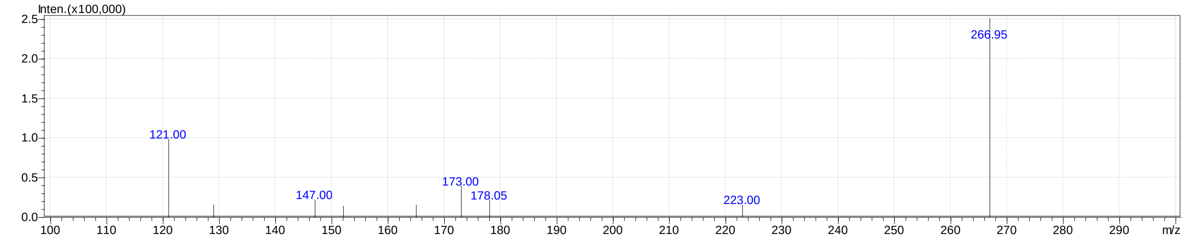

Figure S152. MS analysis flavone 4′-carboxylic acid (**5c**)

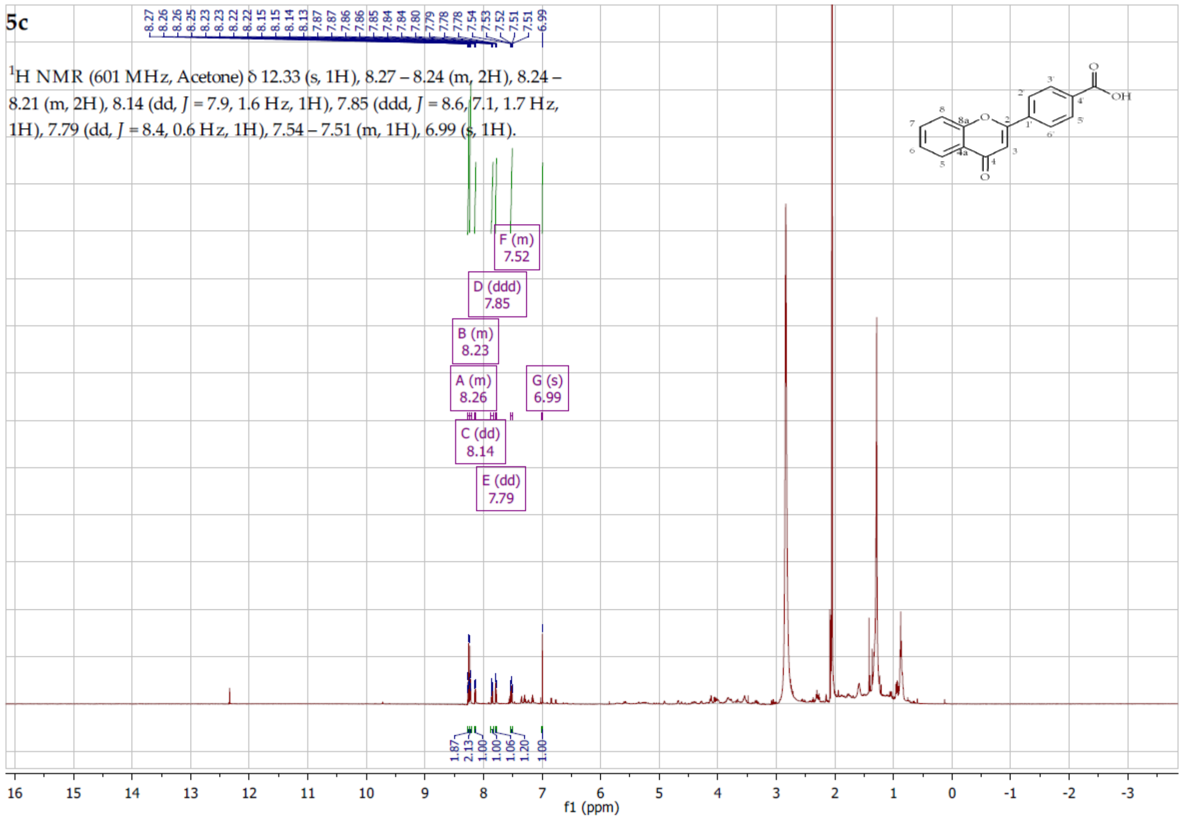

Figure S153. ^{1}H NMR spectrum (*δ*, acetone-d6, 600 MHz) of flavone 4′-carboxylic acid (**5c**)

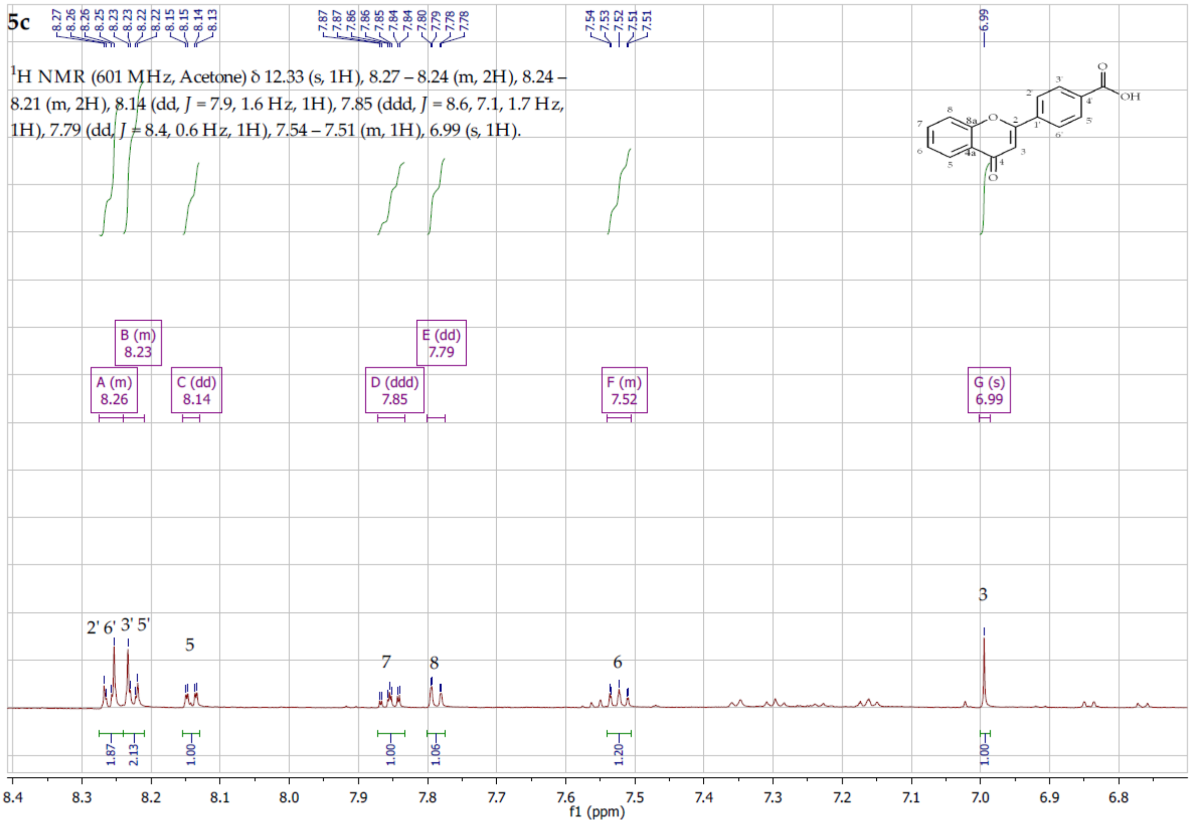

Figure S154. ^{1}H NMR spectrum expansion (*δ*, acetone-d6, 600 MHz) of flavone 4′-carboxylic acid (**5c**)

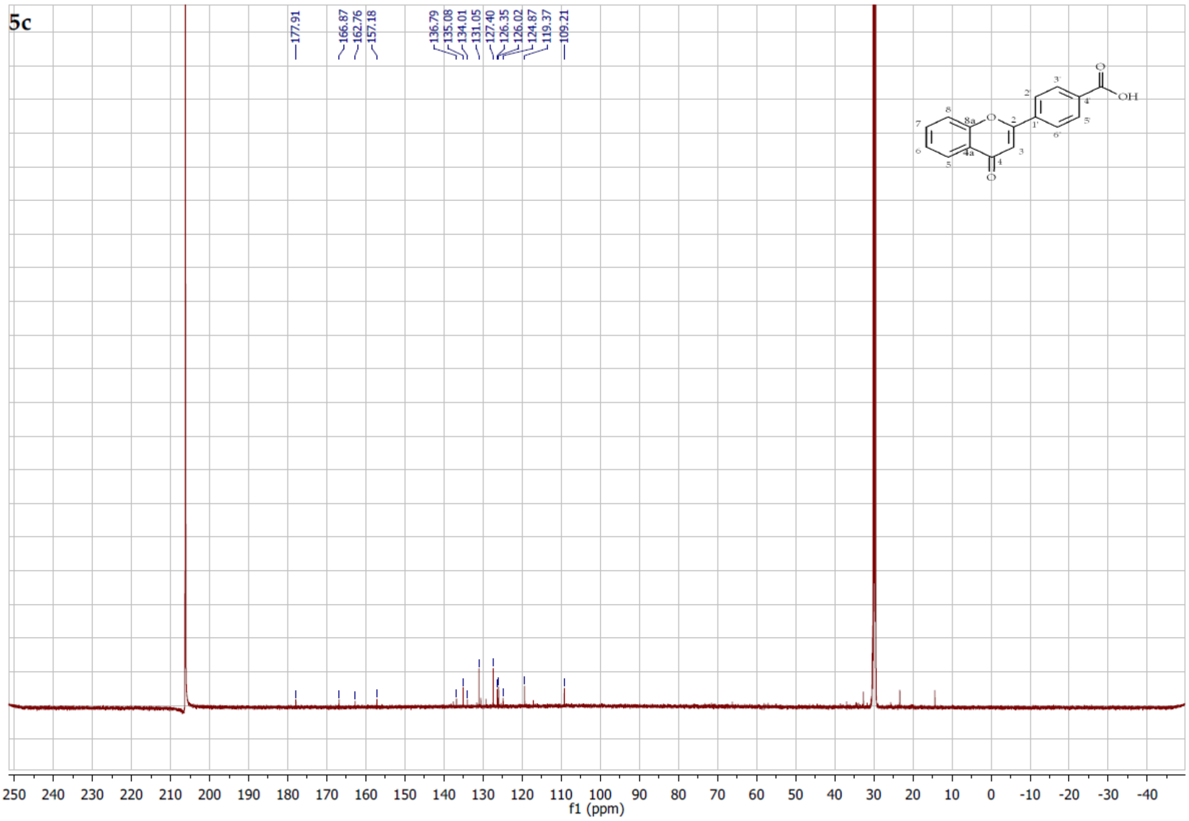

Figure S155. ^{13}C NMR spectrum expansion (*δ*, acetone-d6, 151 MHz) of flavone 4′-carboxylic acid (**5c**)

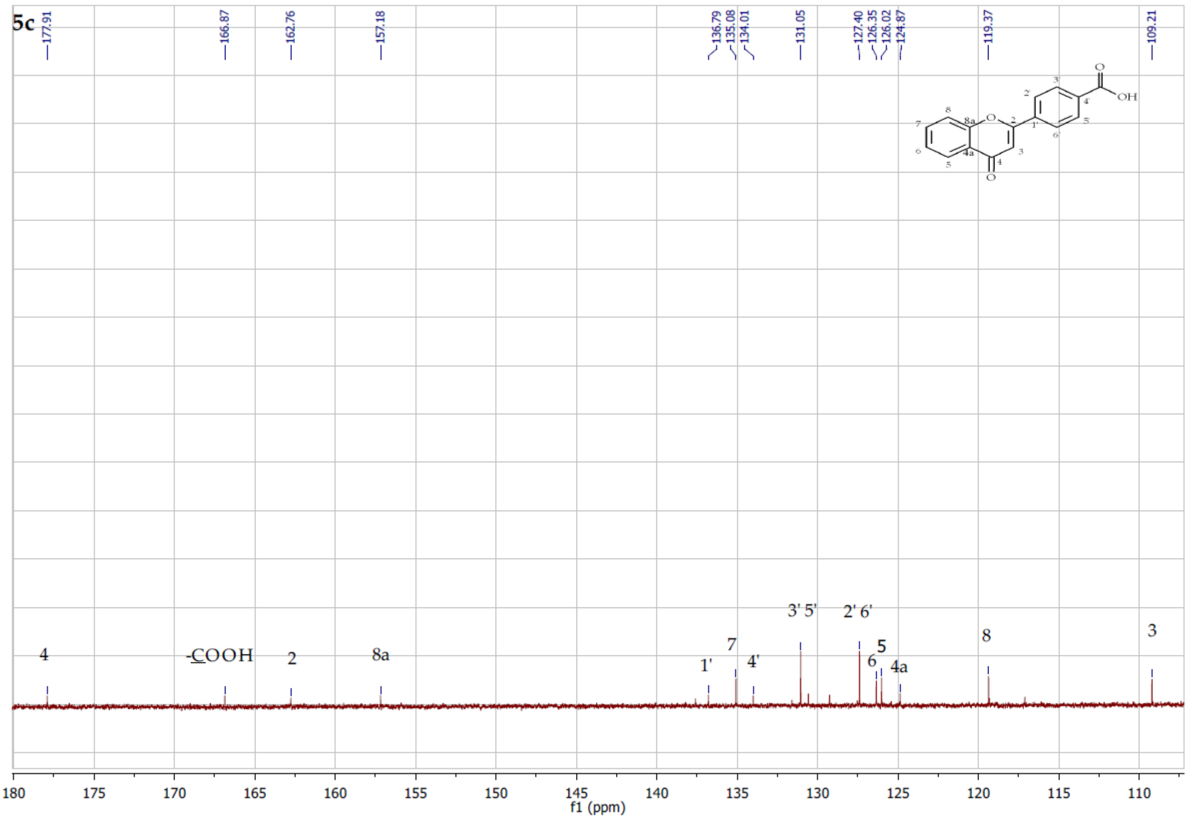

Figure S156. ^{13}C NMR spectrum expansion (δ, acetone-d6, 151 MHz) of flavone 4′-carboxylic acid (**5c**)

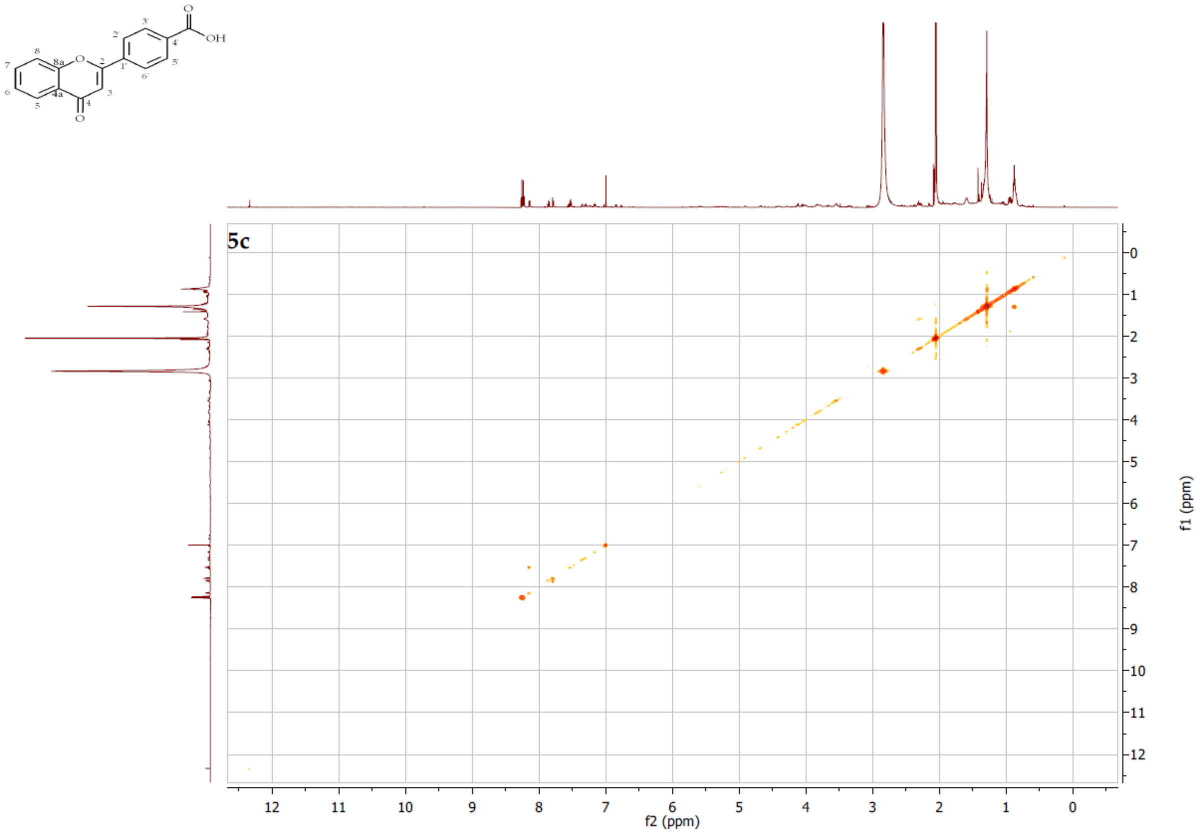

Figure S157. COSY contour map – ^{1}H x ^{1}H of flavone 4′-carboxylic acid (**5c**)

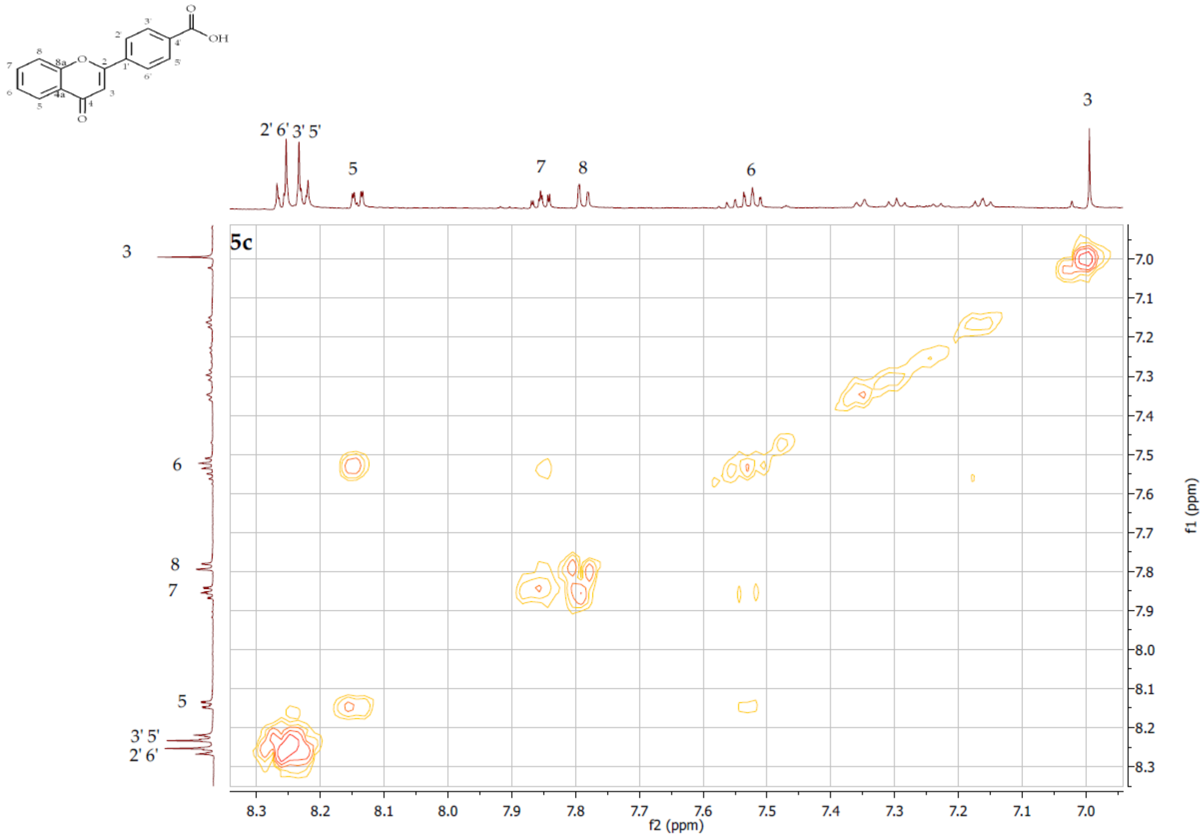

Figure S158. COSY contour map – ^{1}H x ^{1}H expansion of flavone 4′-carboxylic acid (**5c**)

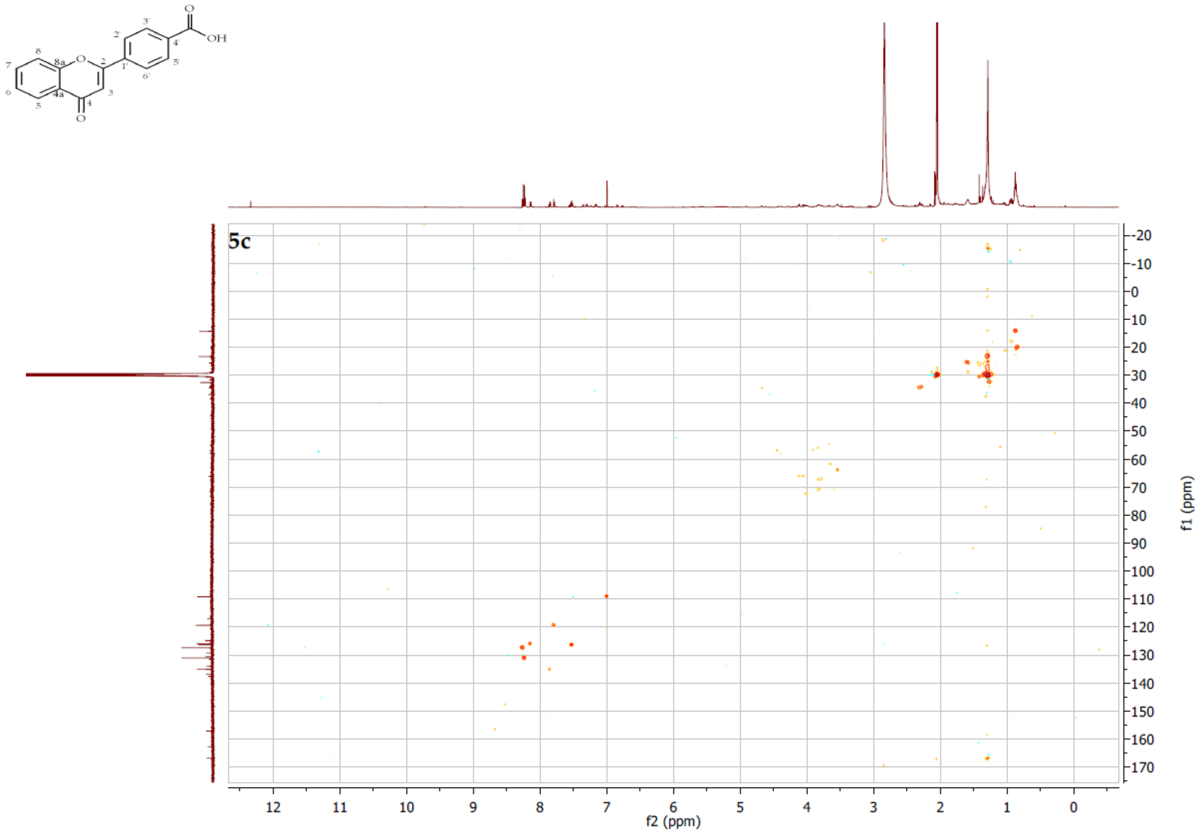

Figure S159. HSQC contour map – ^{1}H x ^{13}C of flavone 4′-carboxylic acid (**5c**)

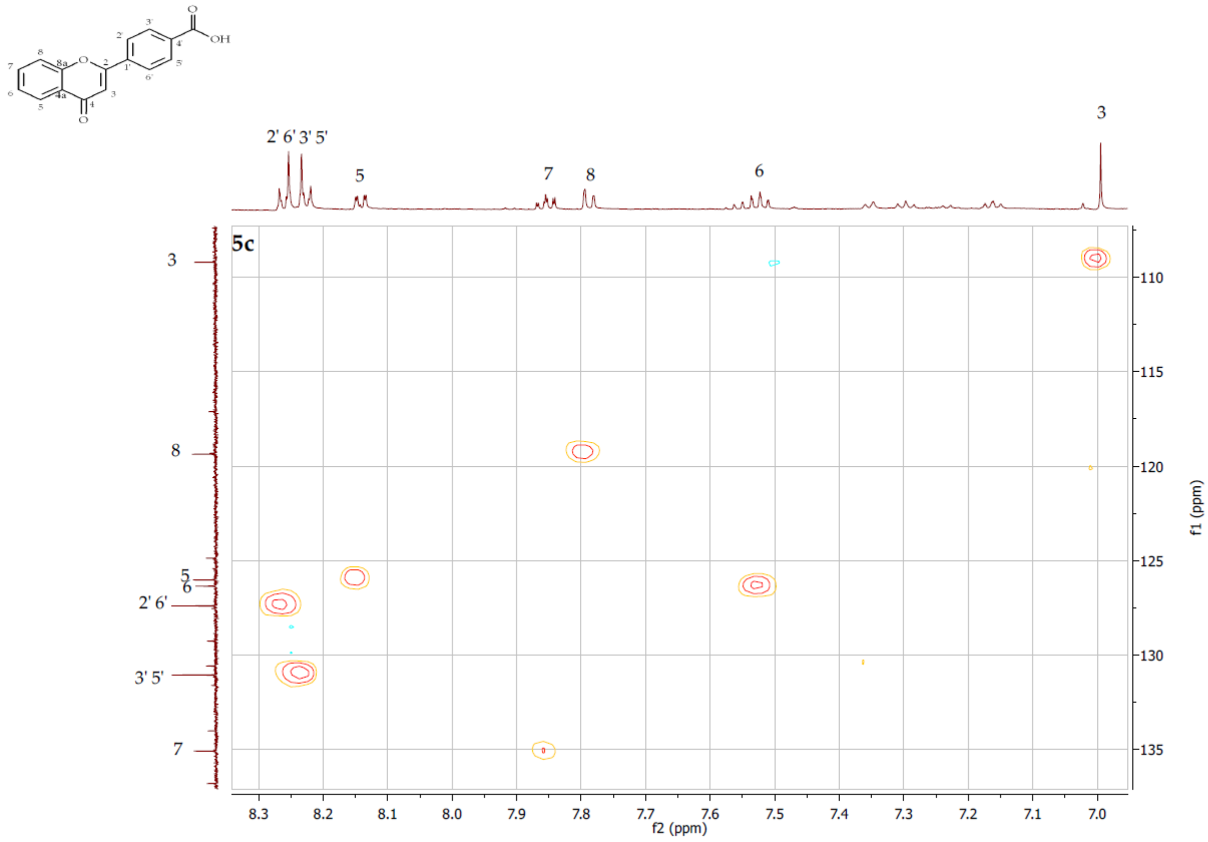

Figure S160. HSQC contour map – ^{1}H x ^{13}C expansion of flavone 4′-carboxylic acid (**5c**)

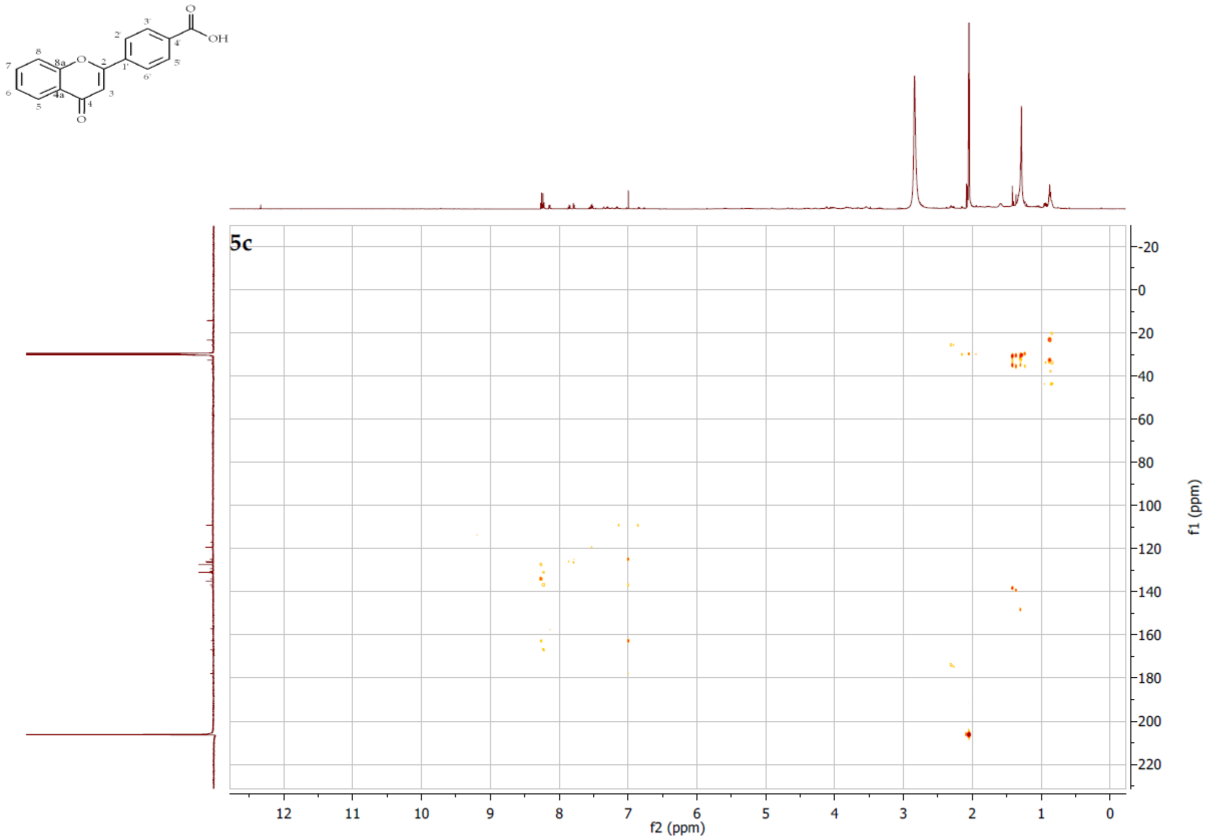

Figure S161. HMBC contour map – ^{1}H x ^{13}C of flavone 4′-carboxylic acid (**5c**)

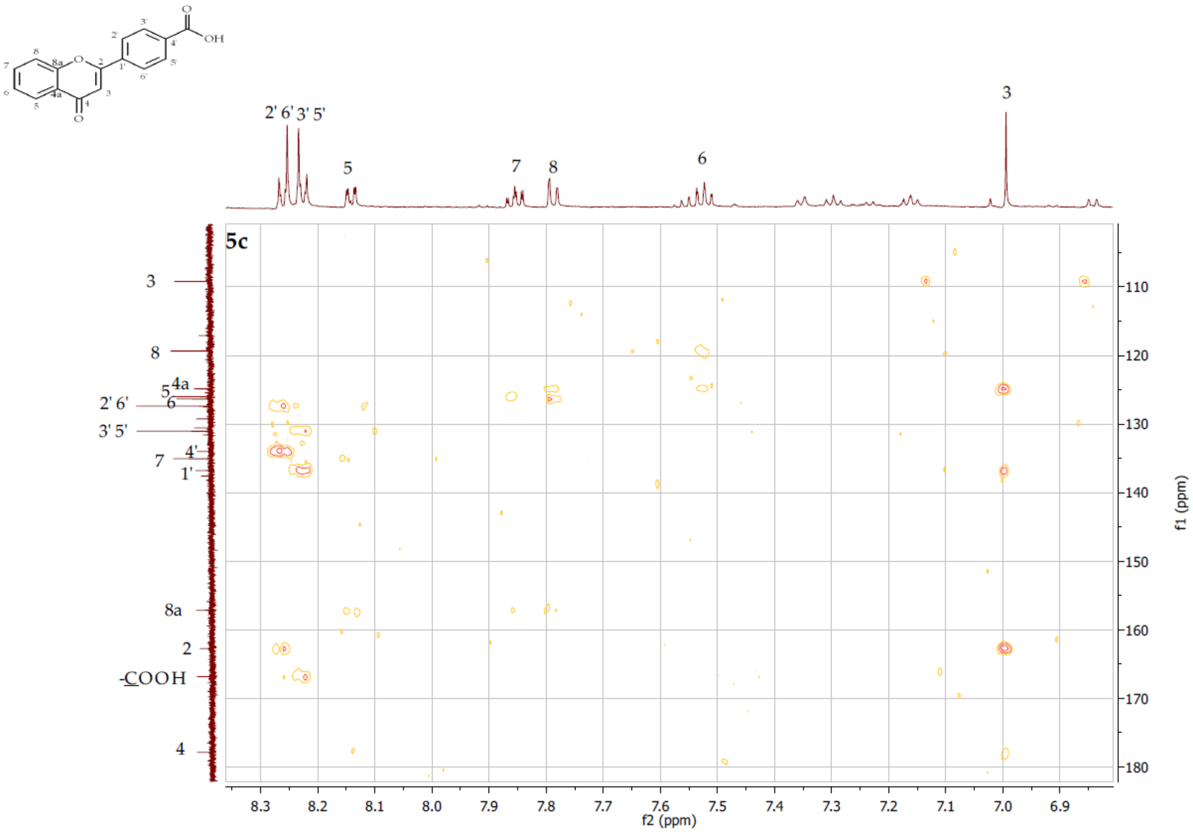

Figure S162. HMBC contour map – ^{1}H x ^{13}C expansion of flavone 4′-carboxylic acid (**5c**)

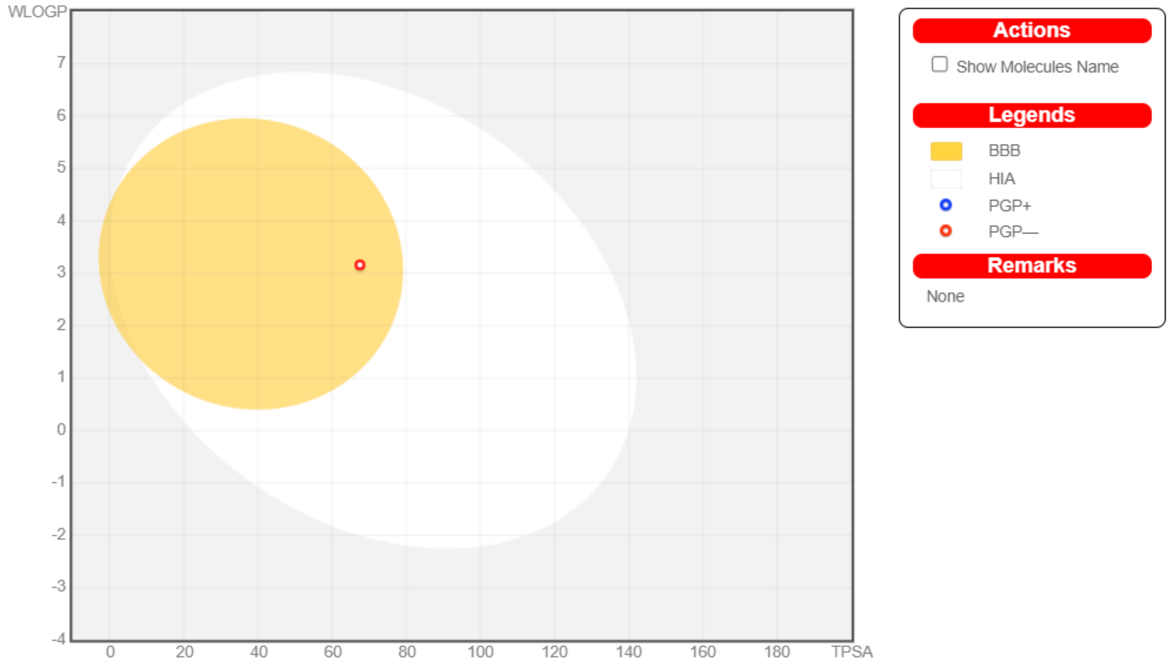

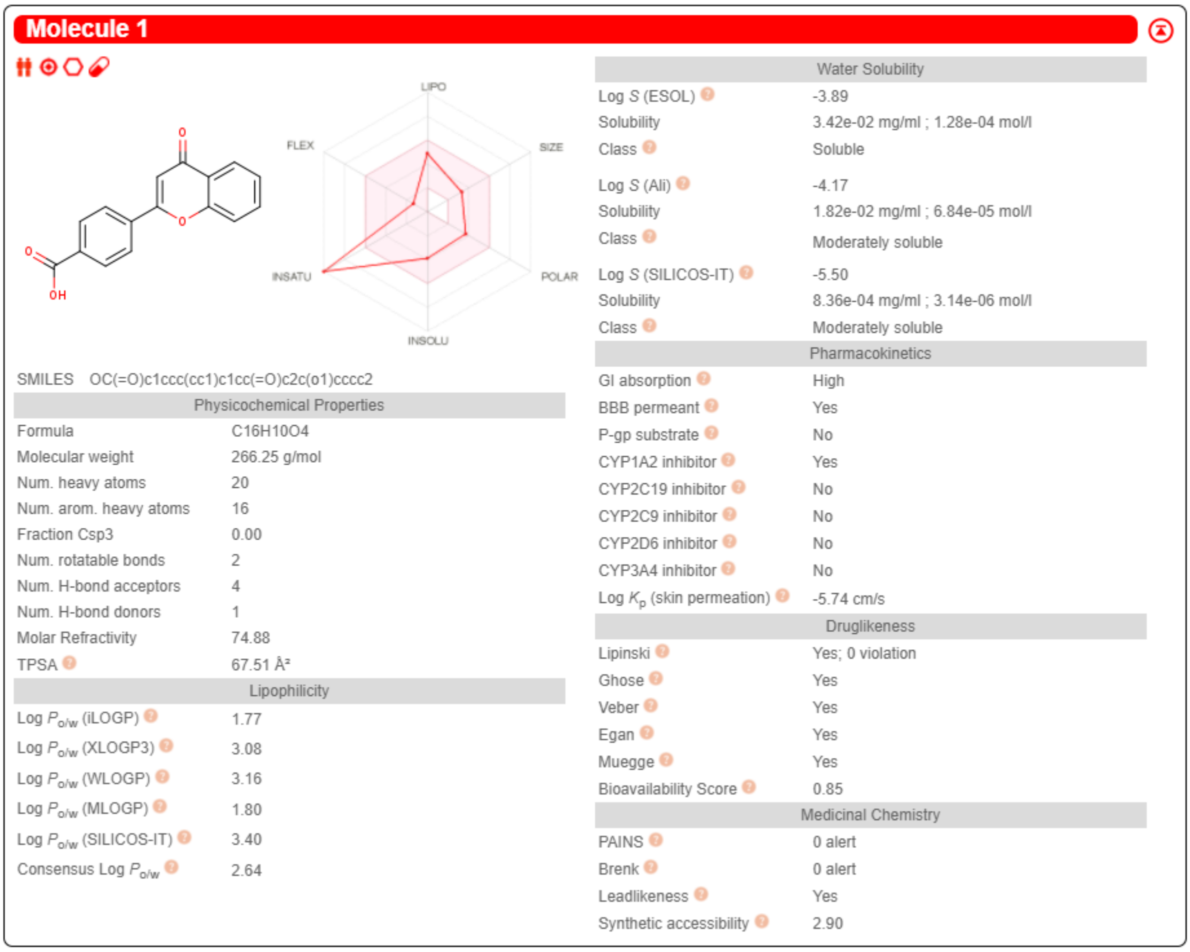

SMILES OC(=O)c1ccc(cc1)c1cc(=O)c2c(o1)cccc2

Physicochemical Properties	
Formula	C16H10O4
Molecular weight	266.25 g/mol
Num. heavy atoms	20
Num. arom. heavy atoms	16
Fraction Csp3	0.00
Num. rotatable bonds	2
Num. H-bond acceptors	4
Num. H-bond donors	1
Molar Refractivity	74.88
TPSA	67.51 Å²

Lipophilicity	
Log $P_{o/w}$ (iLOGP)	1.77
Log $P_{o/w}$ (XLOGP3)	3.08
Log $P_{o/w}$ (WLOGP)	3.16
Log $P_{o/w}$ (MLOGP)	1.80
Log $P_{o/w}$ (SILICOS-IT)	3.40
Consensus Log $P_{o/w}$	2.64

Water Solubility	
Log *S* (ESOL)	-3.89
Solubility	3.42e-02 mg/ml ; 1.28e-04 mol/l
Class	Soluble
Log *S* (Ali)	-4.17
Solubility	1.82e-02 mg/ml ; 6.84e-05 mol/l
Class	Moderately soluble
Log *S* (SILICOS-IT)	-5.50
Solubility	8.36e-04 mg/ml ; 3.14e-06 mol/l
Class	Moderately soluble

Pharmacokinetics	
GI absorption	High
BBB permeant	Yes
P-gp substrate	No
CYP1A2 inhibitor	Yes
CYP2C19 inhibitor	No
CYP2C9 inhibitor	No
CYP2D6 inhibitor	No
CYP3A4 inhibitor	No
Log K_p (skin permeation)	-5.74 cm/s

Druglikeness	
Lipinski	Yes; 0 violation
Ghose	Yes
Veber	Yes
Egan	Yes
Muegge	Yes
Bioavailability Score	0.85

Medicinal Chemistry	
PAINS	0 alert
Brenk	0 alert
Leadlikeness	Yes
Synthetic accessibility	2.90

Figure S163. Flavone 4′-carboxylic acid (**5c**) physicochemical and ADME parameters prediction using the SwissADME modelling

Pa	Pi	Activity
0,972	0,001	4-Nitrophenol 2-monooxygenase inhibitor
0,959	0,002	Chlordecone reductase inhibitor
0,957	0,001	Cholestanetriol 26-monooxygenase inhibitor
0,949	0,001	Aryl-alcohol dehydrogenase (NADP+) inhibitor
0,932	0,002	Alcohol dehydrogenase (NADP+) inhibitor
0,929	0,005	Membrane integrity agonist
0,923	0,002	2-Dehydropantoate 2-reductase inhibitor
0,919	0,005	Methylenetetrahydrofolate reductase (NADPH) inhibitor
0,916	0,003	Membrane permeability inhibitor

Figure S164. Flavone 4′-carboxylic acid (**5c**) biological activity prediction using the Way2Drug Pass online modelling

Name	Confidence	ChEMBL ID
Yersinia pestis	0.5582	CHEMBL614597
RESISTANT Helicobacter pylori	0.4367	CHEMBL612600
Dialister pneumosintes	0.4087	CHEMBL615039
Dialister micraerophilus	0.4087	CHEMBL615038
Dialister propionicifaciens	0.3798	CHEMBL615040
Pseudomonas fluorescens	0.3701	CHEMBL612500
RESISTANT Staphylococcus simulans	0.3328	CHEMBL612425
Parabacteroides merdae	0.3194	CHEMBL615057
Bacteroides uniformis	0.3156	CHEMBL612622
Salmonella enterica subsp. enterica	0.3008	CHEMBL613044
Streptococcus pneumoniae R6	0.2996	CHEMBL2366794
RESISTANT Burkholderia pseudomallei	0.2984	CHEMBL3140323
Shigella sp.	0.2935	CHEMBL614397
Prevotella disiens	0.2823	CHEMBL612235
RESISTANT Clostridium paraputrificum	0.2786	CHEMBL615027

Figure S165. Flavone 4′-carboxylic acid (**5c**) antibacterial activity prediction using the Way2Drug AntiBac-Pred modelling

Name	Confidence	ChEMBL ID
Epidermophyton floccosum	0.3059	CHEMBL612386

Figure S166. Flavone 4′-carboxylic acid (**5c**) antifungal activity prediction using the Way2Drug AntiFun-Pred modelling

Virus	Protein target	Confidence
Human immunodeficiency virus 2	Human immunodeficiency virus type 2 integrase	0.8526
Dengue virus type 2	Genome polyprotein	0.6407
Severe acute respiratory syndrome coronavirus 2	Replicase polyprotein 1ab	0.5298
Vaccinia virus (strain Western Reserve) (VACV) (Vaccinia virus (strainWR))	DNA polymerase	0.3180
SARS coronavirus	Replicase polyprotein 1ab	0.1753
SARS coronavirus	SARS coronavirus 3C-like proteinase	0.1053
Infectious bronchitis virus	3C-like protease	0.0937
Varicella-zoster virus (strain Dumas) (HHV-3) (Human herpesvirus 3)	DNA polymerase	0.0914
Herpes simplex virus (type 1 / strain 17)	Human herpesvirus 1 DNA polymerase	0.0914
Middle East respiratory syndrome-related coronavirus (isolate UnitedKingdom/H123990006/2012) (Betacoronavirus England 1) [illegible]	Replicase polyprotein 1ab	0.0821

Figure S167. Flavone 4′-carboxylic acid (**5c**) antiviral activity prediction using the Way2Drug AntiVir-Pred modelling

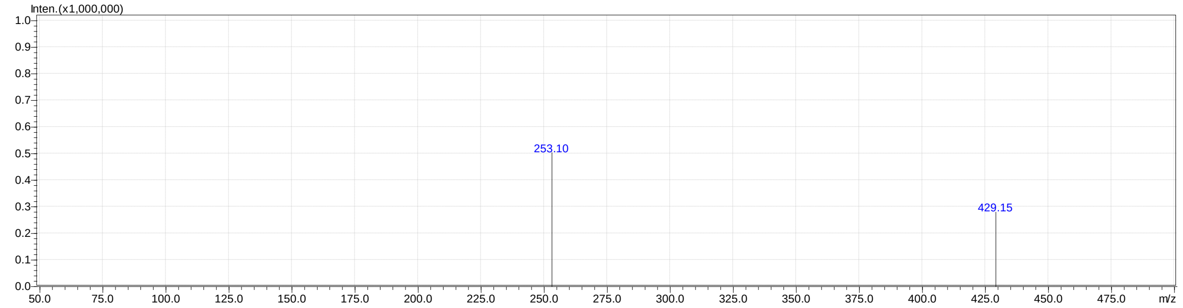

Figure S168. MS analysis 4′-methylflavone 3-*O*-*β*-D-(4′′-*O*-methyl)-glucopyranoside (**5d**)

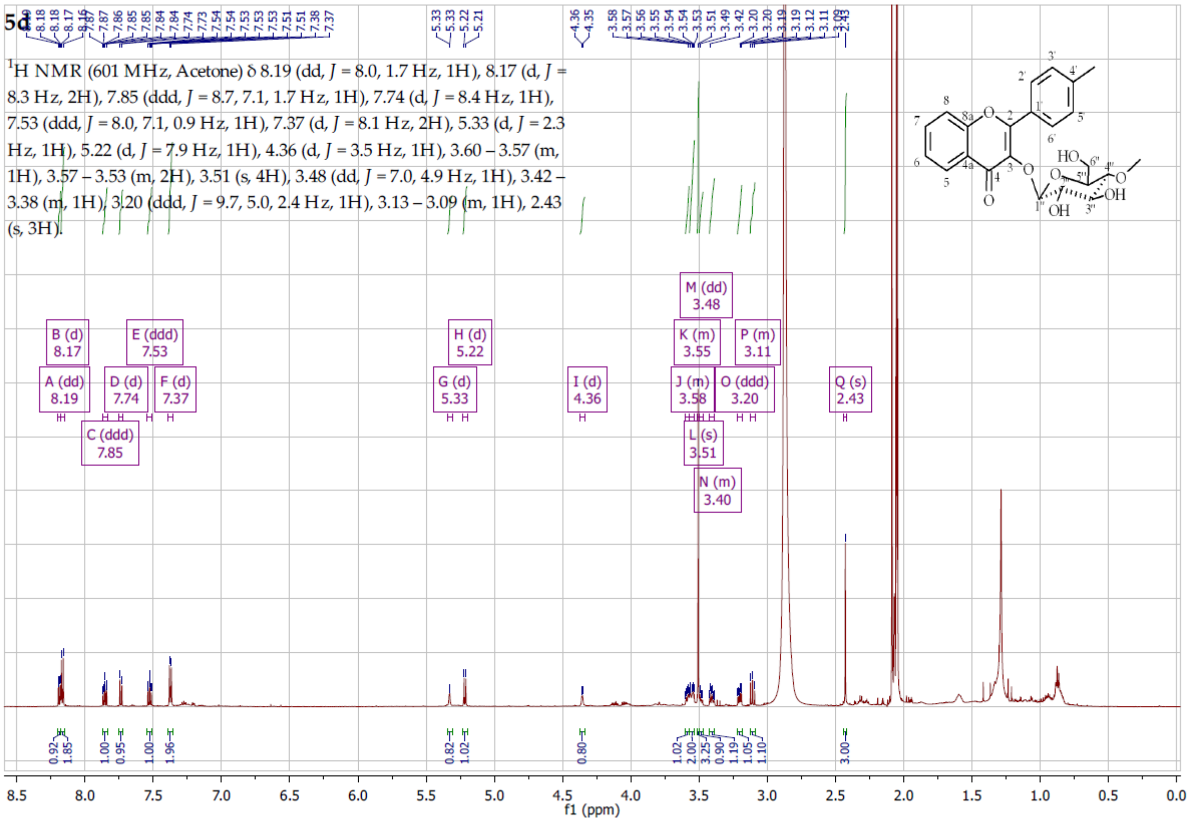

Figure S169. ^{1}H NMR spectrum (*δ*, acetone-d6, 600 MHz) of 4′-methylflavone 3-*O*-*β*-D-(4′′-*O*-methyl)-glucopyranoside (**5d**)

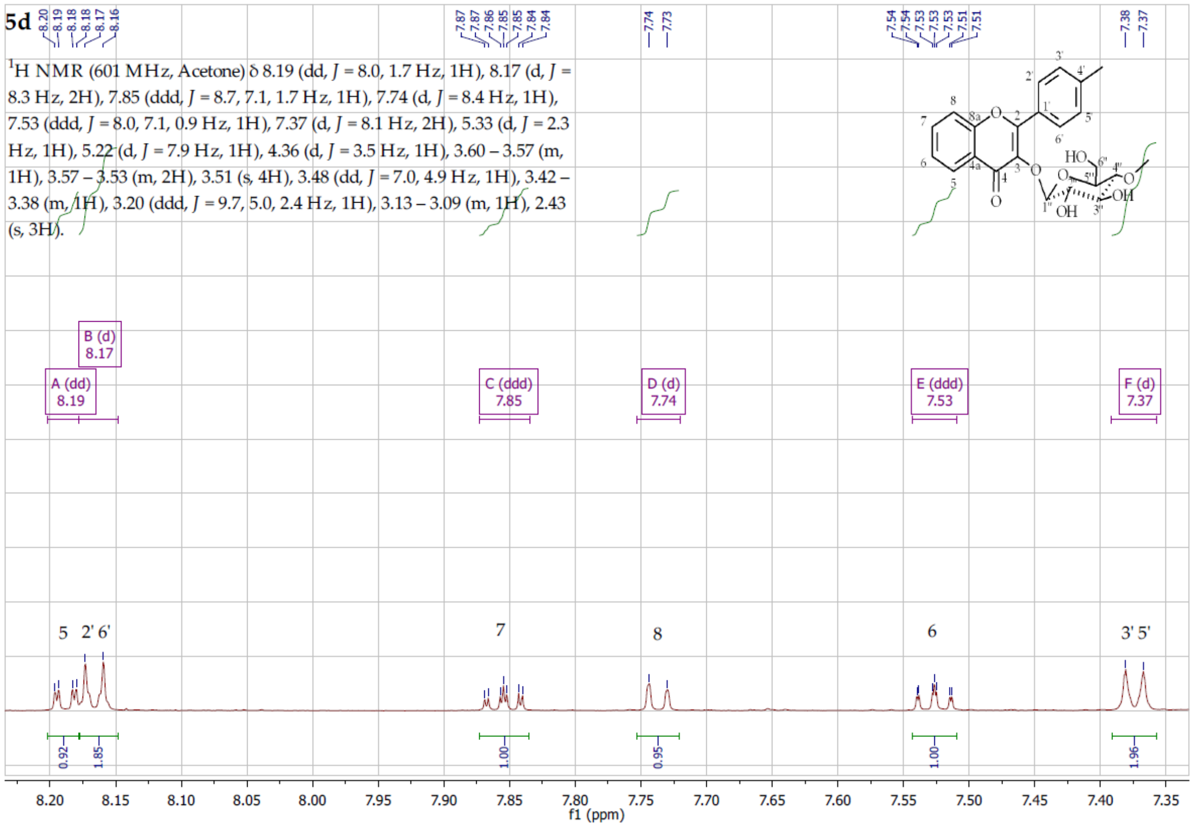

Figure S170. ^{1}H NMR spectrum expansion (*δ*, acetone-d6, 600 MHz) of 4′-methylflavone 3-*O*-*β*-D-(4′′-*O*-methyl)-glucopyranoside (**5d**)

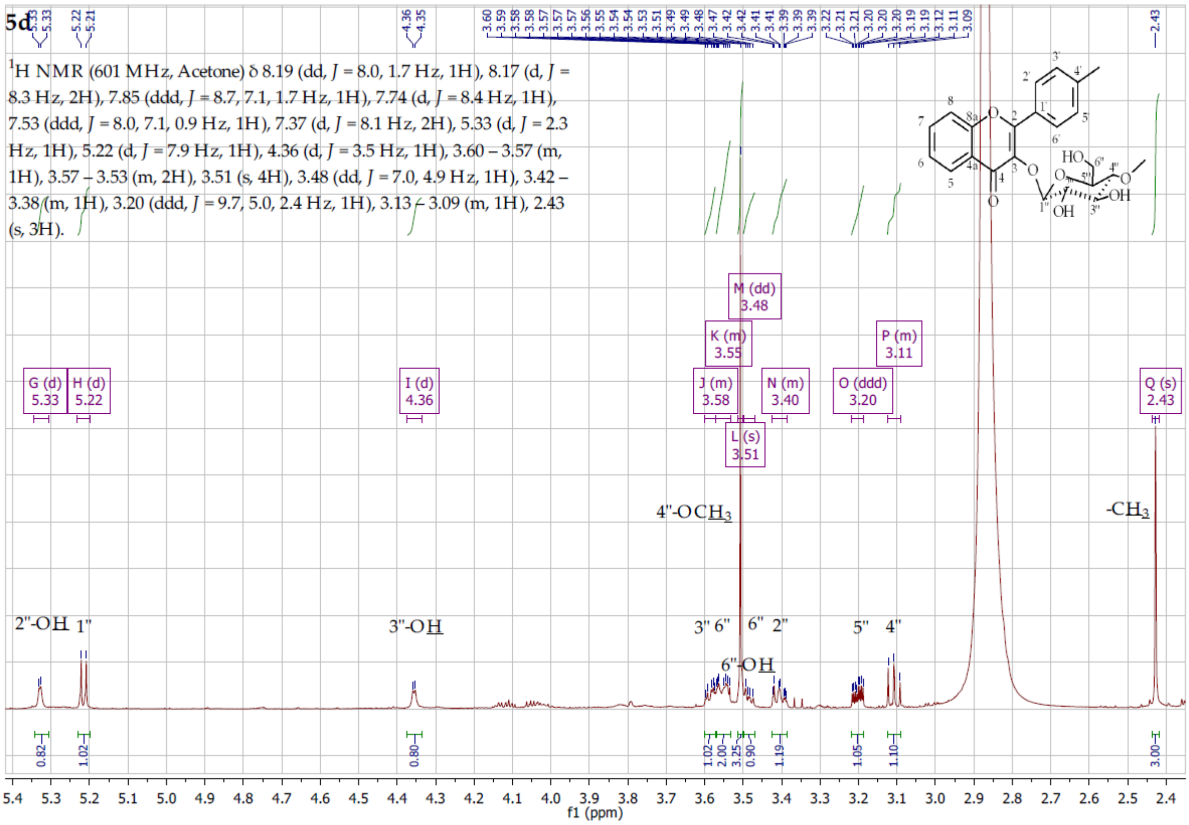

Figure S171. ^{1}H NMR spectrum expansion (*δ*, acetone-d6, 600 MHz) of 4′-methylflavone 3-*O*-*β*-D-(4′′-*O*-methyl)-glucopyranoside (**5d**)

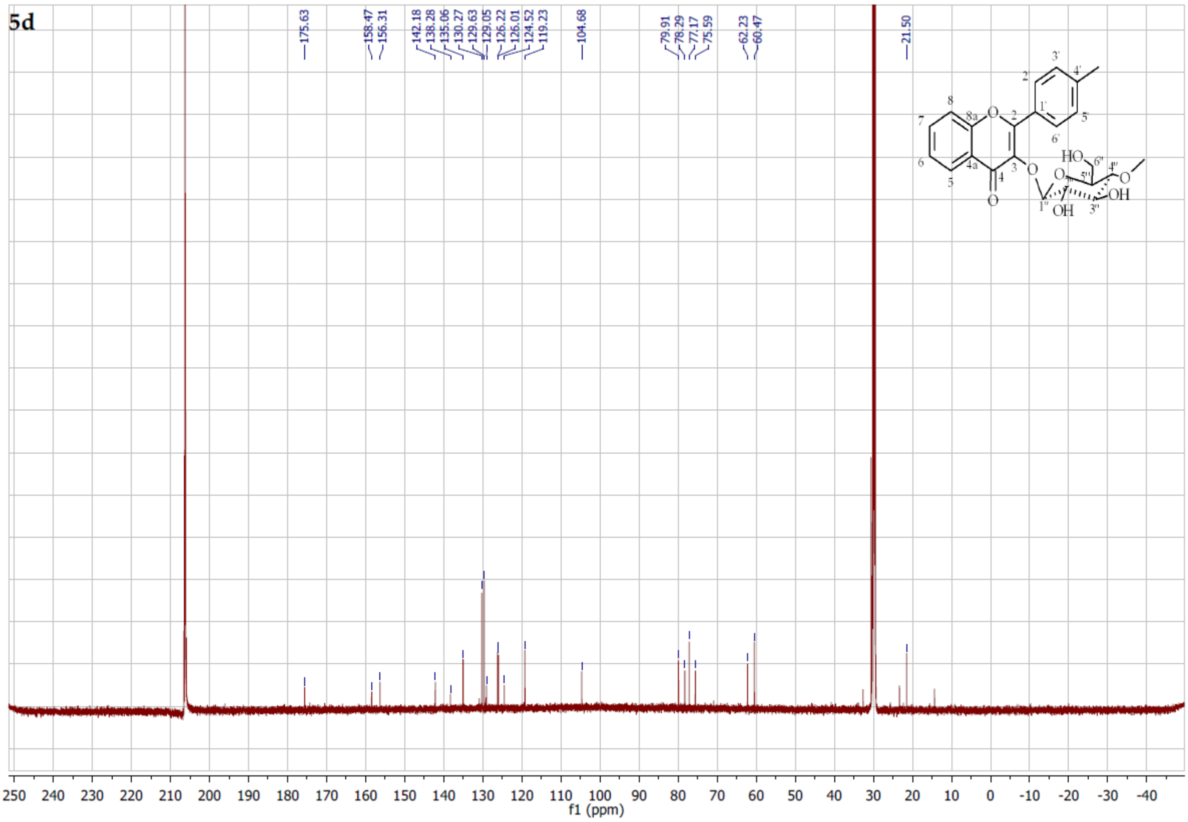

Figure S172. ^{13}C NMR spectrum expansion (*δ*, acetone-d6, 151 MHz) of 4′-methylflavone 3-*O*-*β*-D-(4′′-*O*-methyl)-glucopyranoside (**5d**)

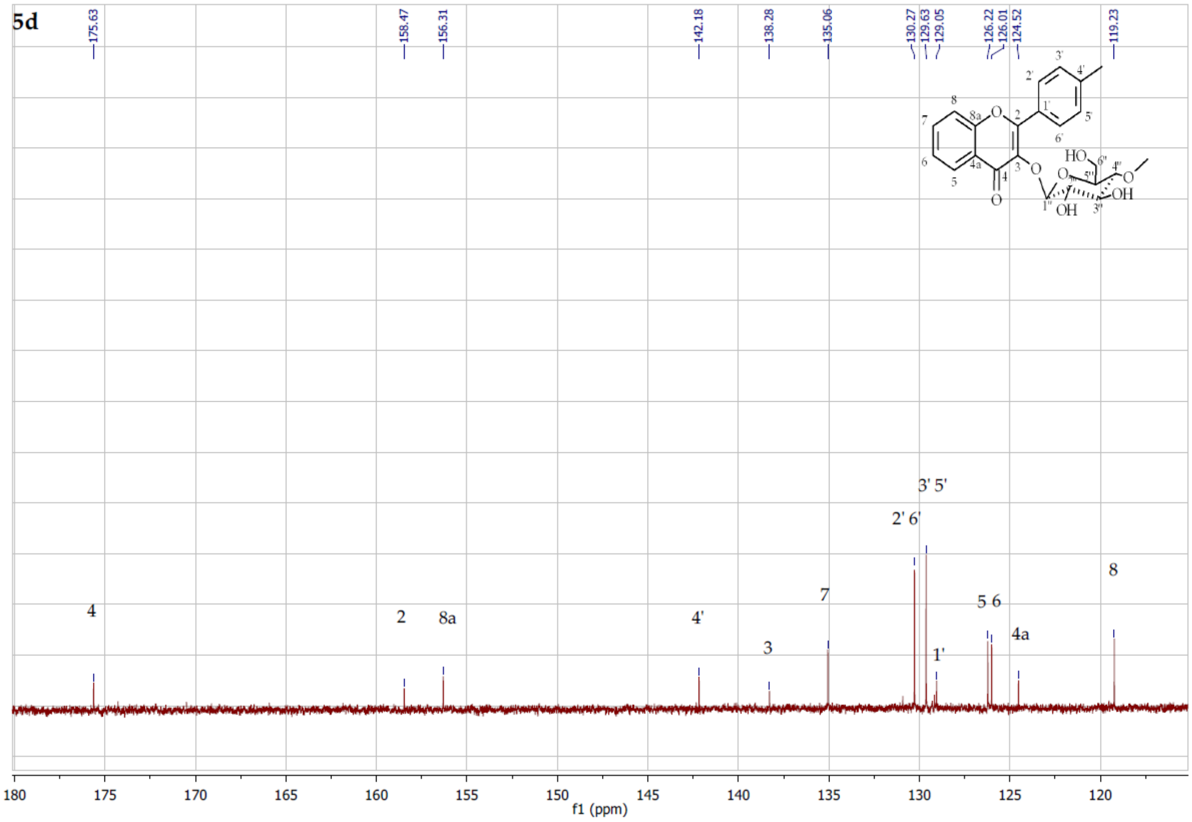

Figure S173. ^{13}C NMR spectrum expansion (δ, acetone-d6, 151 MHz) of 4′-methylflavone 3-*O*-*β*-D-(4′′-*O*-methyl)-glucopyranoside (**5d**)

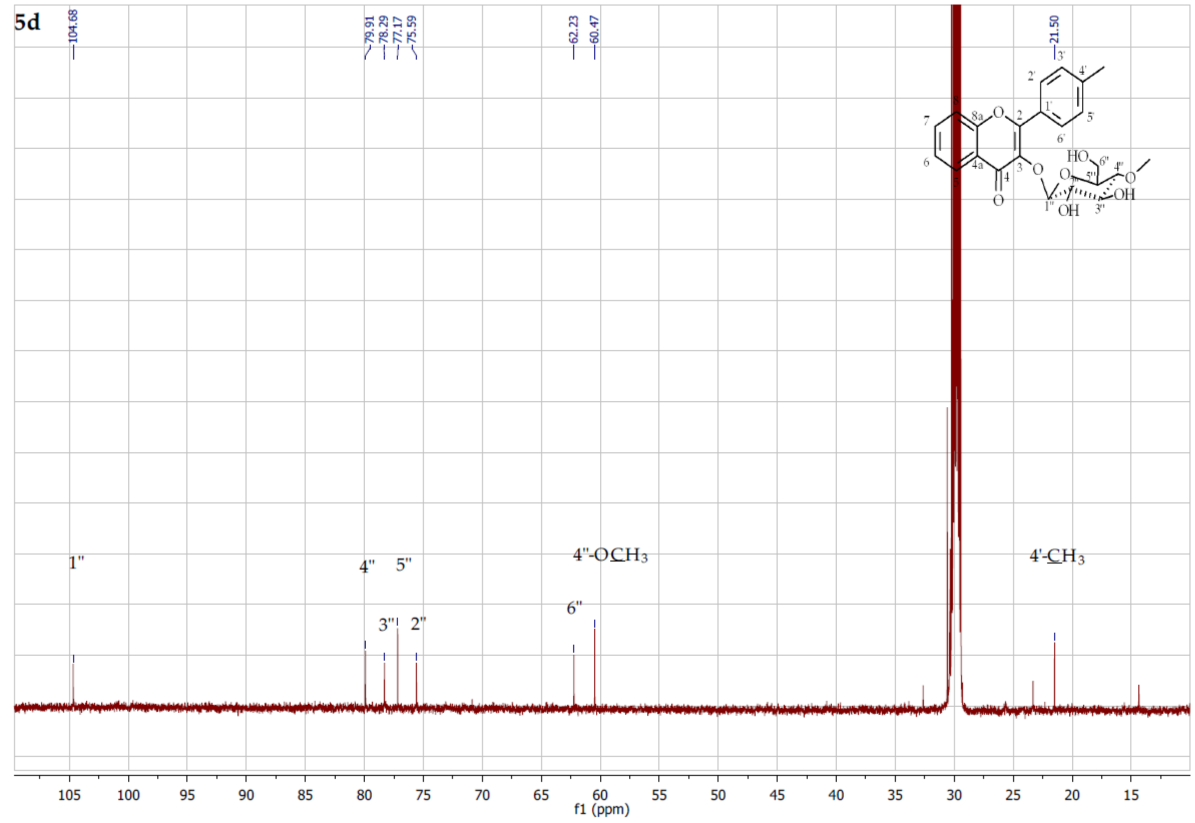

Figure S174. ^{13}C NMR spectrum expansion (δ, acetone-d6, 151 MHz) of 4′-methylflavone 3-*O*-*β*-D-(4′′-*O*-methyl)-glucopyranoside (**5d**)

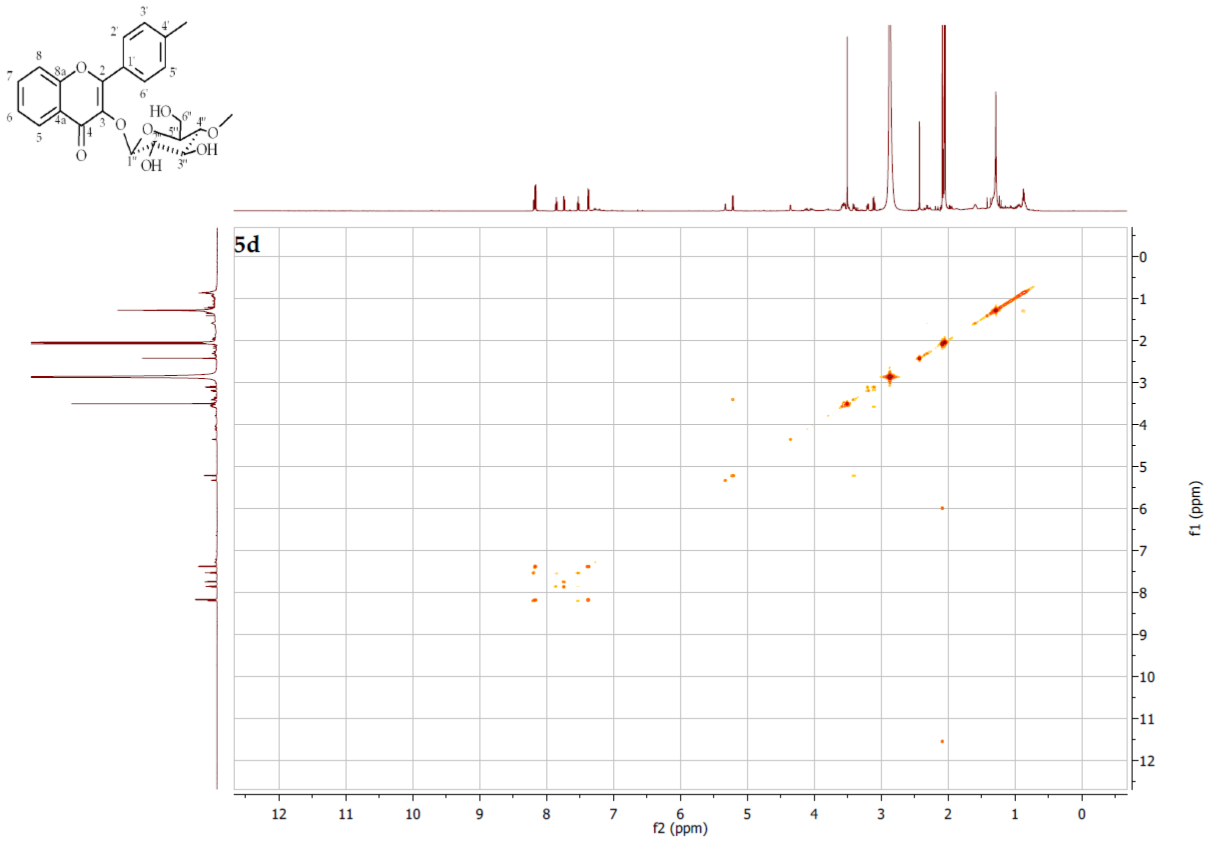

Figure S175. COSY contour map – $^{1}H \times {}^{1}H$ of 4′-methylflavone 3-*O*-*β*-D-(4′′-*O*-methyl)-glucopyranoside (**5d**)

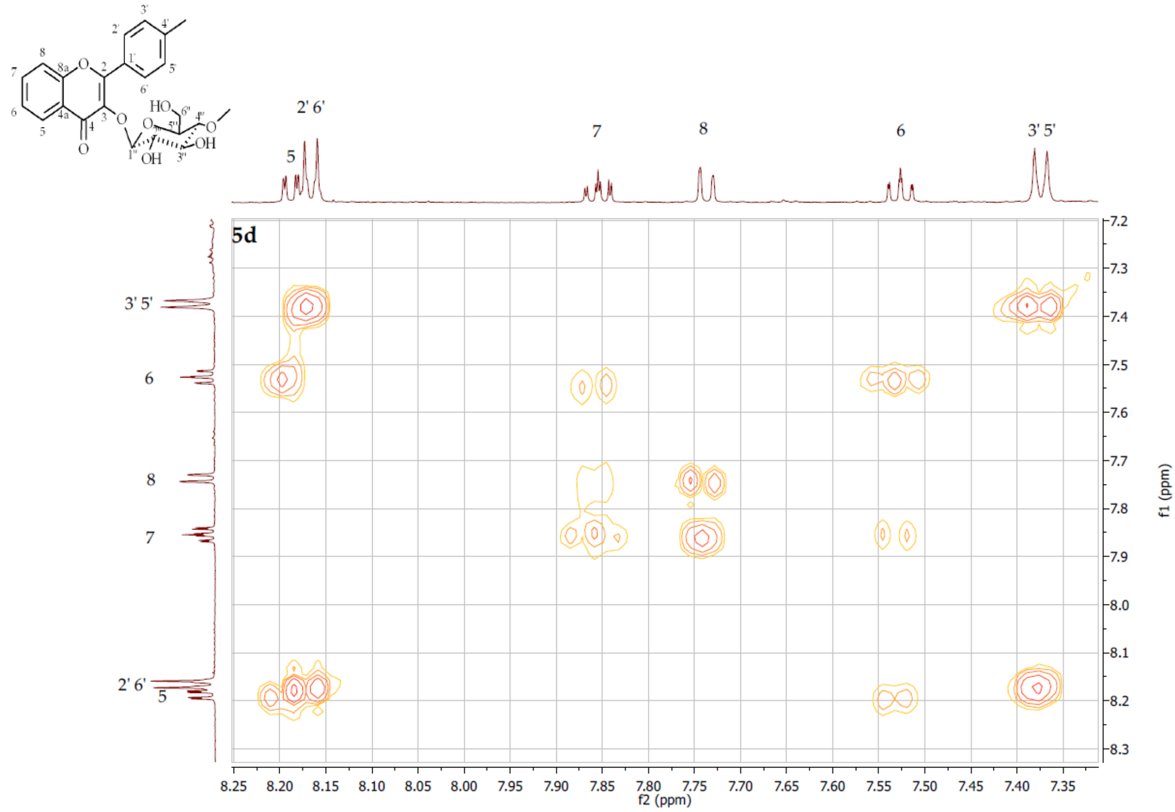

Figure S176. COSY contour map – $^{1}H \times {}^{1}H$ expansion of 4′-methylflavone 3-*O*-*β*-D-(4′′-*O*-methyl)-glucopyranoside (**5d**)

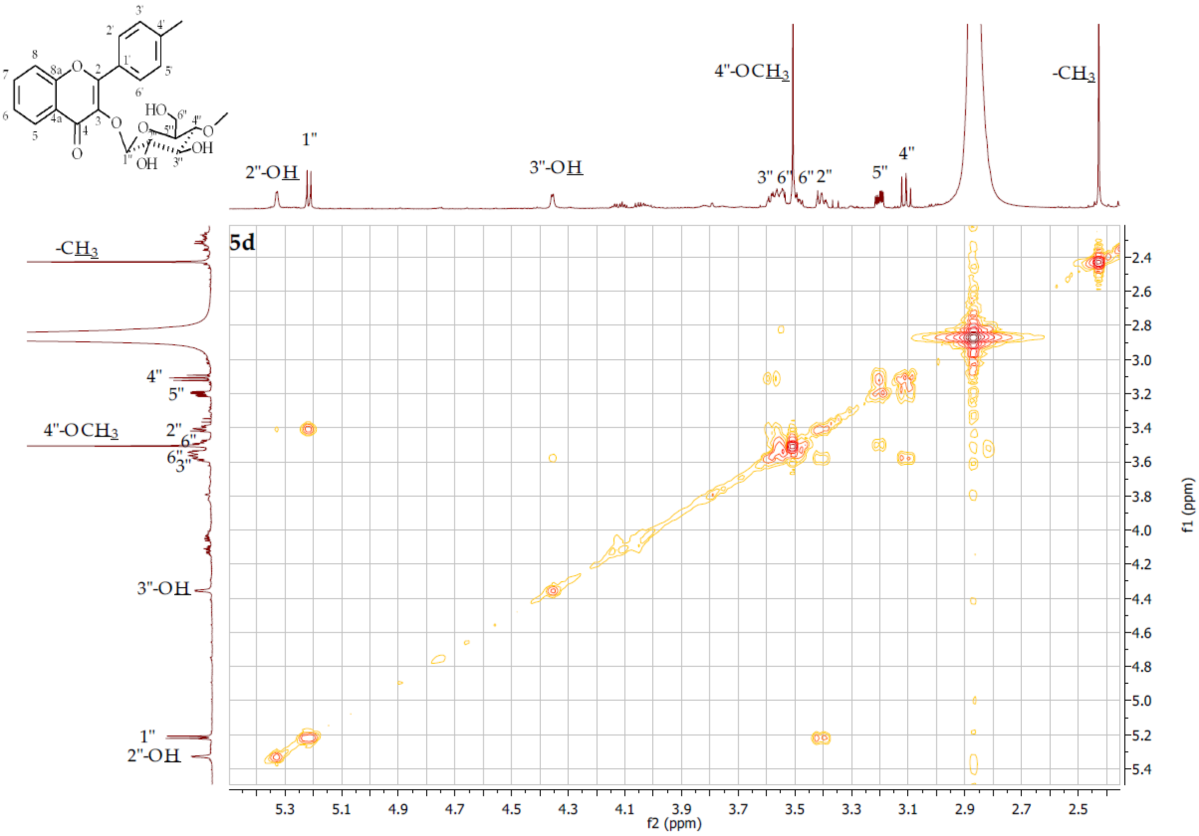

Figure S177. COSY contour map – ^{1}H x ^{1}H expansion of 4′-methylflavone 3-*O*-*β*-D-(4′′-*O*-methyl)-glucopyranoside (**5d**)

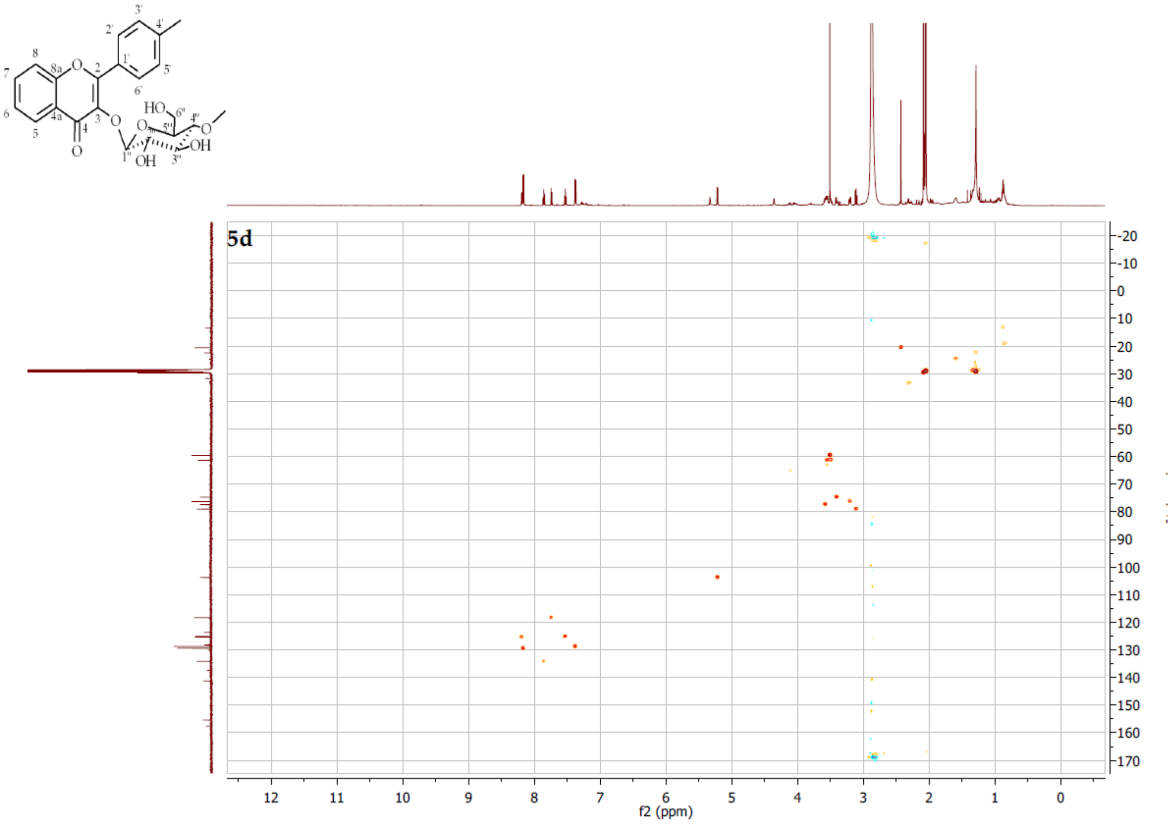

Figure S178. HSQC contour map – ^{1}H x ^{13}C of 4′-methylflavone 3-*O*-*β*-D-(4′′-*O*-methyl)-glucopyranoside (**5d**)

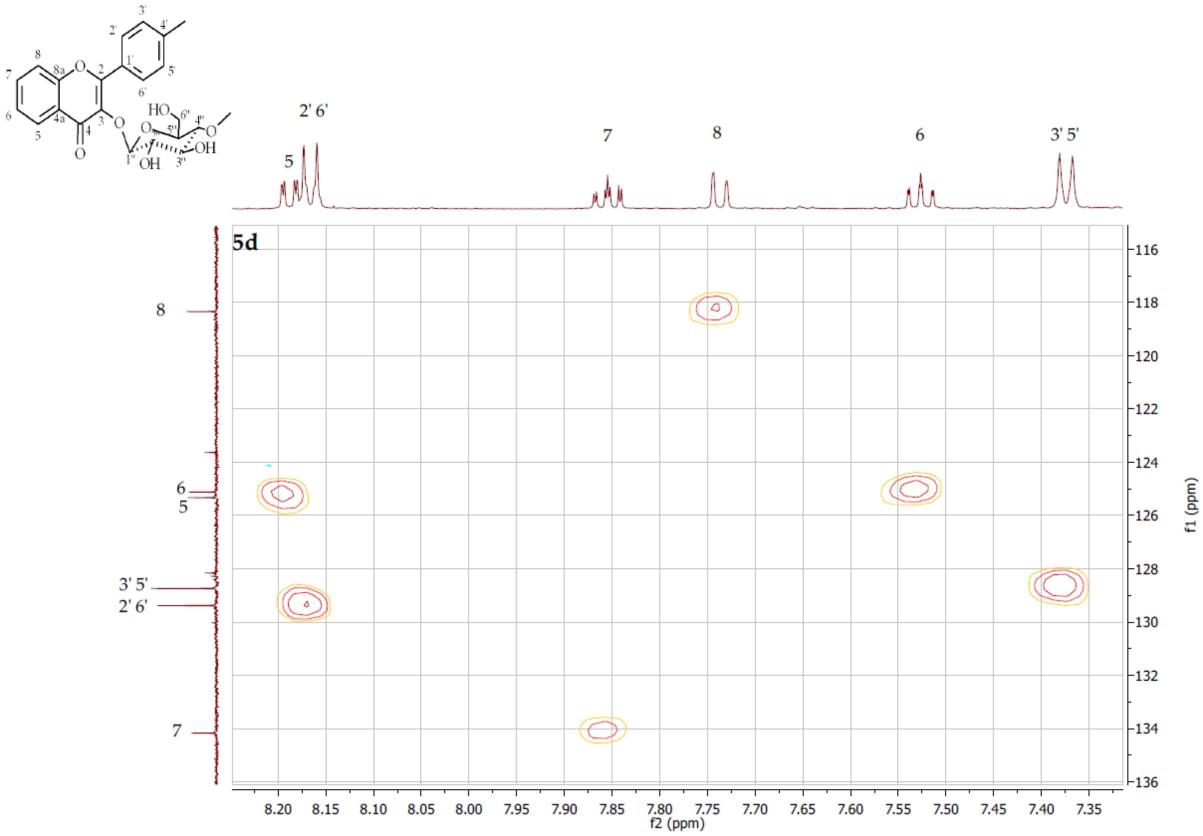

Figure S179. HSQC contour map – ^{1}H x ^{13}C expansion of 4′-methylflavone 3-*O*-*β*-D-(4′′-*O*-methyl)-glucopyranoside (**5d**)

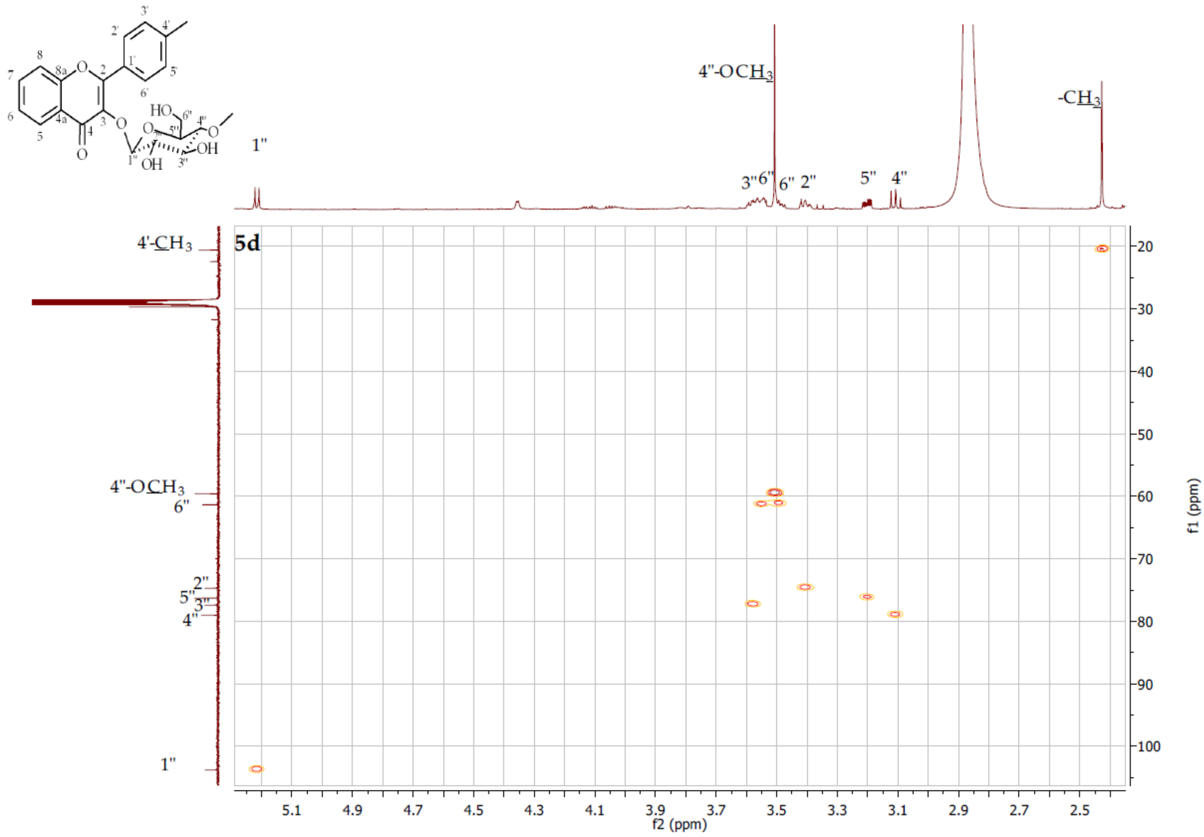

Figure S180. HSQC contour map – ^{1}H x ^{13}C expansion of 4′-methylflavone 3-*O*-*β*-D-(4′′-*O*-methyl)-glucopyranoside (**5d**)

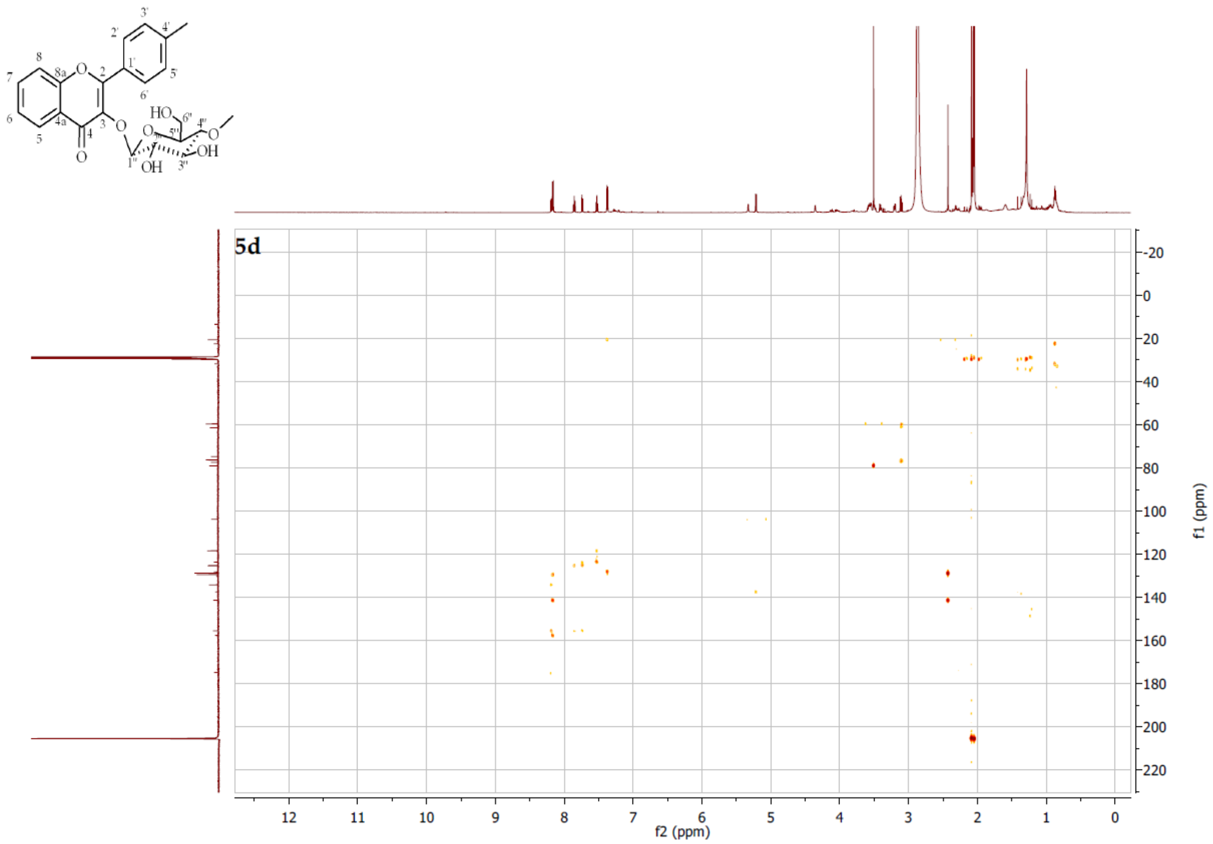

Figure S181. HMBC contour map – ^{1}H x ^{13}C of 4′-methylflavone 3-*O*-*β*-D-(4′′-*O*-methyl)-glucopyranoside (**5d**)

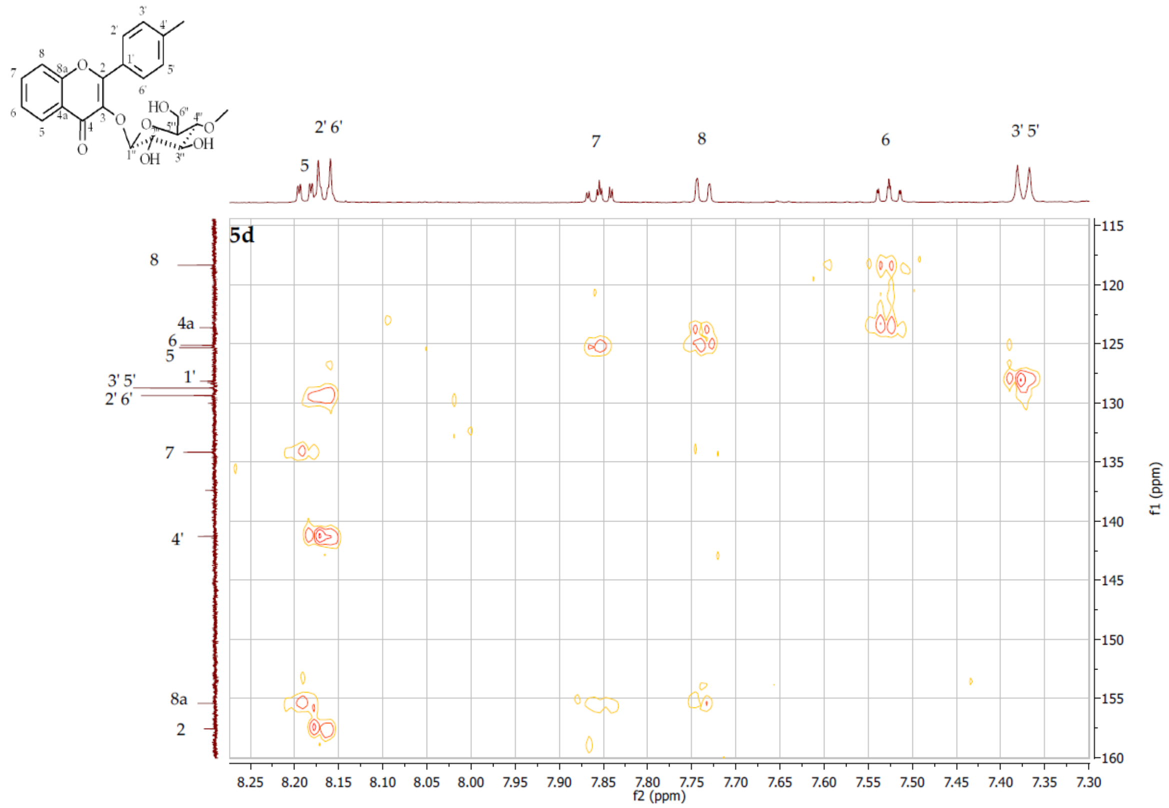

Figure S182. HMBC contour map – ^{1}H x ^{13}C expansion of 4′-methylflavone 3-*O*-*β*-D-(4′′-*O*-methyl)-glucopyranoside (**5d**)

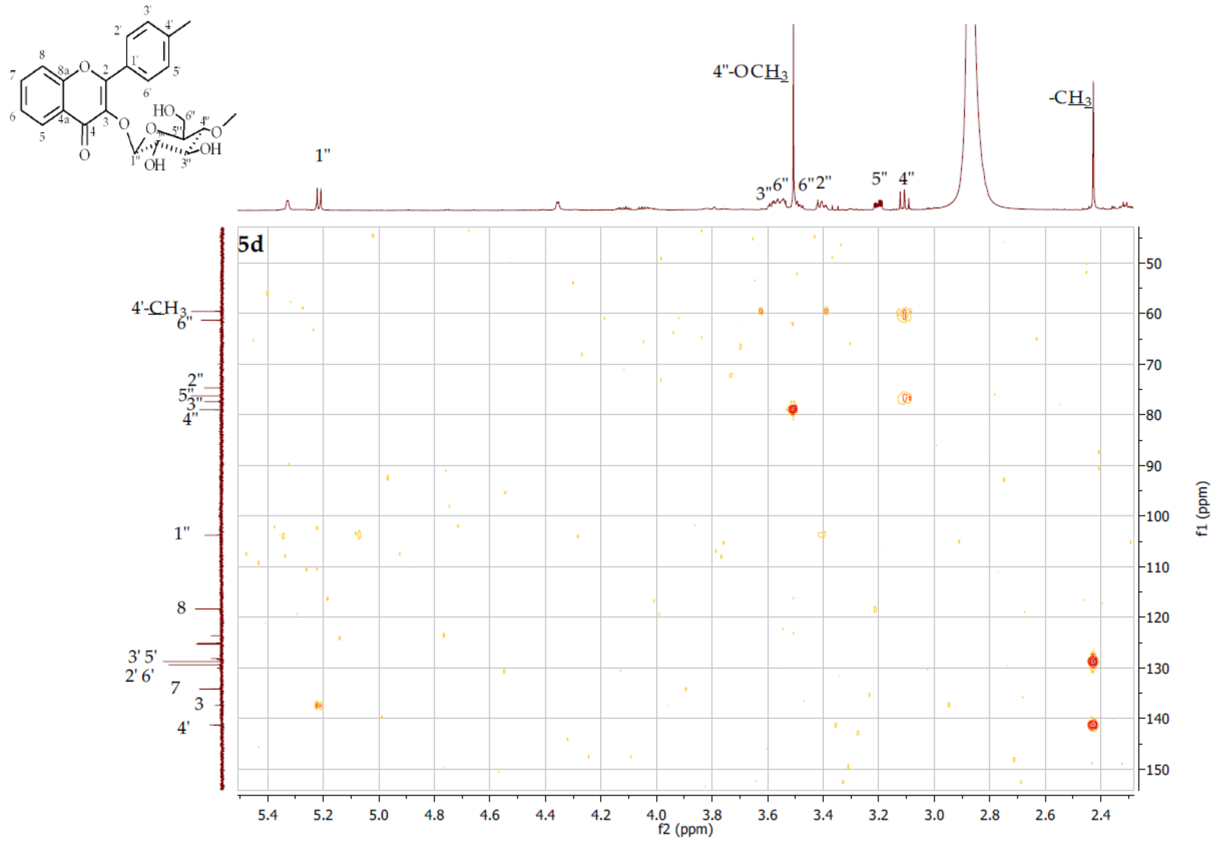

Figure S183. HMBC contour map – $^{1}H \times {}^{13}C$ expansion of 4′-methylflavone 3-*O*-*β*-D-(4′′-*O*-methyl)-glucopyranoside (**5d**)

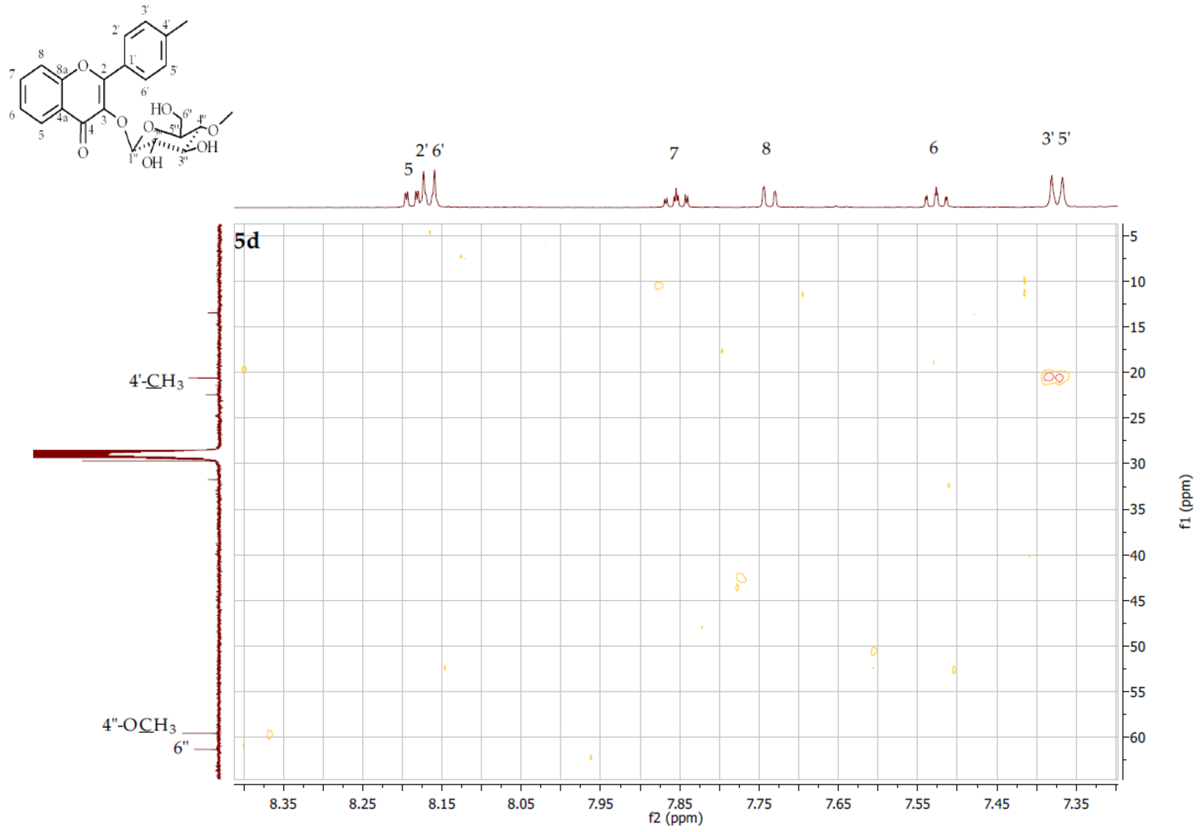

Figure S184. HMBC contour map – $^{1}H \times {}^{13}C$ expansion of 4′-methylflavone 3-*O*-*β*-D-(4′′-*O*-methyl)-glucopyranoside (**5d**)

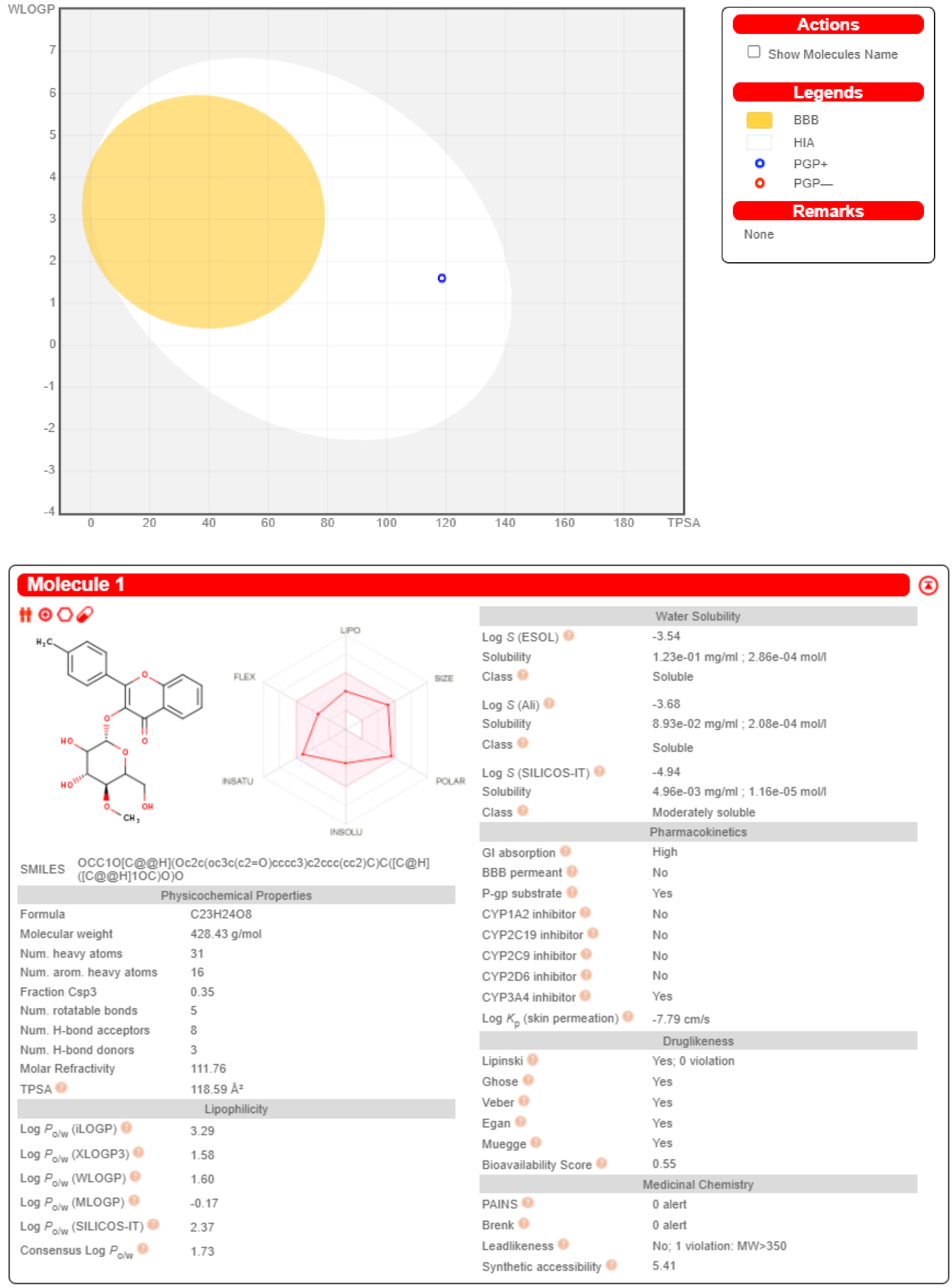

Figure S185. 4′-Methylflavone 3-*O*-*β*-D-(4′′-*O*-methyl)-glucopyranoside (**5d**) physicochemical and ADME parameters prediction using the SwissADME modelling

Pa	Pi	Activity
0,971	0,002	Membrane integrity agonist
0,967	0,001	Membrane permeability inhibitor
0,939	0,002	Cardioprotectant
0,937	0,005	CDP-glycerol glycerophosphotransferase inhibitor
0,914	0,003	Vasoprotector
0,912	0,002	Monophenol monooxygenase inhibitor
0,904	0,004	Anaphylatoxin receptor antagonist
0,895	0,003	Hepatoprotectant
0,889	0,003	Anticarcinogenic
0,887	0,002	Free radical scavenger

Figure S186. 4′-Methylflavone 3-*O*-*β*-D-(4′′-*O*-methyl)-glucopyranoside (**5d**) biological activity prediction using the Way2Drug Pass online modelling

Name	Confidence	ChEMBL ID
Clostridium ramosum	0.6641	CHEMBL614971
Yersinia pestis	0.6518	CHEMBL614597
Actinomyces meyeri	0.6127	CHEMBL612289
RESISTANT Acinetobacter pittii	0.5964	CHEMBL3140321
RESISTANT Mycobacterium ulcerans	0.5889	CHEMBL612965
Mycobacterium mageritense	0.5780	CHEMBL612959
Clostridium cadaveris	0.5715	CHEMBL614970
RESISTANT Staphylococcus aureus subsp. aureus RN4220	0.4894	CHEMBL2366906
Lactobacillus plantarum	0.4445	CHEMBL614973
Staphylococcus lugdunensis	0.4419	CHEMBL613303
Streptococcus pneumoniae R6	0.4406	CHEMBL2366794
Nocardia transvalensis	0.4250	CHEMBL613234
Streptococcus oralis	0.4241	CHEMBL613305
Clostridium sordellii	0.4177	CHEMBL613072
Fusobacterium sp.	0.3864	CHEMBL612690

Figure S187. 4′-Methylflavone 3-*O*-*β*-D-(4′′-*O*-methyl)-glucopyranoside (**5d**) antibacterial activity prediction using the Way2Drug AntiBac-Pred modelling

Name	Confidence	ChEMBL ID
Rhizopus oryzae	0.4609	CHEMBL612306
Absidia corymbifera	0.4043	CHEMBL612369
Trichophyton mentagrophytes	0.2885	CHEMBL613162
Candida dubliniensis	0.2241	CHEMBL613334
Aspergillus niger	0.1790	CHEMBL358
Mucor	0.1328	CHEMBL612521
Epidermophyton floccosum	0.1019	CHEMBL612386
Penicillium marneffei	0.0950	CHEMBL612994
Saccharomyces cerevisiae	0.0845	CHEMBL361
Galactomyces geotrichum	0.0495	CHEMBL613775
Mucor hiemalis	0.0354	CHEMBL612949
Candida rugosa	0.0279	CHEMBL612869

Figure S188. 4′-Methylflavone 3-*O*-*β*-D-(4′′-*O*-methyl)-glucopyranoside (**5d**) antifungal activity prediction using the Way2Drug AntiFun-Pred modelling

Virus	Protein target	Confidence
Severe acute respiratory syndrome coronavirus 2	Replicase polyprotein 1ab	0.9419
Human immunodeficiency virus 2	Human immunodeficiency virus type 2 integrase	0.3592
Varicella-zoster virus<br>(strain Dumas)<br>(HHV-3)<br>(Human herpesvirus 3)	Thymidine kinase	0.0130

Figure S189. 4′-Methylflavone 3-*O*-*β*-D-(4′′-*O*-methyl)-glucopyranoside (**5d**) antiviral activity prediction using the Way2Drug AntiVir-Pred modelling
